# A Novel Two-Component System Involved in the Transition to Secondary Metabolism in *Streptomyces coelicolor*

**DOI:** 10.1371/journal.pone.0031760

**Published:** 2012-02-09

**Authors:** Daniel Rozas, Sonia Gullón, Rafael P. Mellado

**Affiliations:** Centro Nacional de Biotecnología (CSIC), Madrid, Spain; Texas A&M University, United States of America

## Abstract

**Background:**

Bacterial two-component signal transduction regulatory systems are the major set of signalling proteins frequently mediating responses to changes in the environment. They typically consist of a sensor, a membrane-associated histidine kinase and a cytoplasmic response regulator. The membrane-associated sensor detects the environmental signal or stress, whereas the cytoplasmic regulatory protein controls the cellular response usually by gene transcription modulation.

**Methodology/PrincipalFindings:**

The *Streptomyces coelicolor* two genes operon *SCO5784*-*SCO5785* encodes a two-component system, where *SCO5784* encodes a histidine-kinase sensor and *SCO5785* encodes a response regulator protein. When the expression level of the regulator gene decreases, the antibiotic synthesis and sporulation is delayed temporarily in addition to some ribosomal genes became up regulated, whereas the propagation of the regulatory gene in high copy number results in the earlier synthesis of antibiotics and sporulation, as well as the down regulation of some ribosomal genes and, moreover, in the overproduction of several extracellular proteins. Therefore, this two-component system in *S. coelicolor* seems to influence various processes characterised by the transition from primary to secondary metabolism, as determined by proteomic and transcriptomic analyses.

**Conclusions/Significance:**

Propagation of *SCO5785* in multicopy enhances the production of antibiotics as well as secretory proteins. In particular, the increase in the expression level of secretory protein encoding genes, either as an artefactual or real effect of the regulator, could be of potential usefulness when using *Streptomyces* strains as hosts for homologous or heterologous extracellular protein production.

## Introduction

Bacteria frequently use two-component signal transduction regulatory systems to sense changes in the environment. These two-component systems usually are composed of a membrane-associated histidine kinase, the sensor, and a response regulator, which acts in the cytoplasm. The sensor detects the environmental signal or stress, and the regulatory protein triggers the cellular response via gene transcription modulation.

Streptomycetes are among the more numerous and ubiquitous soil bacteria [1] that undergo a complex biochemical and morphological differentiation [2], including the formation of a substrate mycelium from which a filamentous vegetative growth gives rise to an aerial mycelium containing long chains of reproductive spores. In order to adapt to their natural environment, largely formed by insoluble polymers, streptomycetes need to produce and secrete large quantities of proteins [3], such as suitable hydrolytic enzymes, which are more prevalent, together with antibiotics and signalling molecules. The capacity to accumulate hydrolytic enzymes and antibiotics extracellularly makes bacterial species from the *Streptomyces* genus attractive for industrial and medical application.

The best-known representative of the *Streptomyces* genus is the *Streptomyces coelicolor* A3 (2) bacterium. The complete sequence of its genome revealed the existence of eighty-five sensor kinases and seventy-nine response regulators, including fifty-three sensor-regulator pairs [4]. More recently, the number of sensor kinase genes has been revised to a total of eighty-four and the response regulators to eighty. Sixty-seven of the eighty-four sensor kinase genes are adjacent to the response regulator genes in the genome, therefore constituting potential sensor-regulator pairs. The remaining seventeen sensor genes are unpaired, in addition to the remaining thirteen response regulators [5]. *S. coelicolor* is a soil-dwelling microorganism that needs to respond to a large number of variable conditions within its natural environment. The amount of environmental stimuli to which a bacterium can respond seems to be linked to that of the sensor kinase genes contained in its genome and is also proportional to its size [5]. The *S. coelicolor* number of paired sensor kinase-response regulator genes suggests that this bacterium would have to deal with a wide range of environmental signals and stimuli [4].

Although the two-component sensor kinase-response regulator pairs have been classified in different groups [5], and despite the knowledge accumulated over many years on *S. coelicolor* metabolism and its regulatory networks, very little is known about the functions and targets of the *S. coelicolor* sixty-seven two-component systems. We report the identification and functional characterisation by proteomic and transcriptomic analyses of one of the sixty-seven two-component systems of *S. coelicolor* (*SCO5784*-*SCO5785*). This system was in principle selected as being one of the two-component systems that apparently constitute a single two-genes operon, sharing some degree of homology with the *B. subtilis degS-degU* operon, and whose activity, similarly to the *B. subtilis* one, apparently influences the time and level of antibiotic production, and stimulates the synthesis of some secretory proteins [6], [7].

## Results

### Expression of genes modulated by SCO5785 overproduction or its deficiency

It was not possible to obtain mutations in the regulator and sensor genes. Therefore, the operon was interrupted by the insertion of the plasmid pAC301 between the sensor and the regulator genes, as described in Materials and Methods, and hence the regulator gene was no longer under the control of the native promotor operon. The cellular response to the absence of the response regulator *SCO5785* in the strain carrying a *SCO5784*-*SCO5785* disrupted operon was measured by analysing the overall gene expression of the bacterial cell using genome-wide microarrays (Table S1). It has been shown that propagation in high copy number of some regulator genes has helped to reveal the effects that the regulator produces in the cell [6], [7], [8]. Therefore, the cellular response to the presence in high copy number of the response regulator *SCO5785* was also measured by analysing overall bacterial gene expression using genome-wide microarrays (Table S2).

Total RNA was extracted from the cell cultures in minimal medium at the late exponential phase of growth. All microarray analyses were performed with RNA samples obtained from three independent cultures grown under identical conditions. The cDNA obtained from each RNA preparation of the strain *S. coelicolor* M28 (overproducer strain), carrying the *SCO5785* gene in the multicopy plasmid pIJ487, was hybridised with the cDNA obtained from the equivalent RNA preparation of its wild type isogenic strain (*S. coelicolor* M145 [pIJ487]). The strain *S. coelicolor* I32 (deficient strain), carrying the disrupted operon, where the regulator gene is no longer under the control of the operon promoter, was hybridised with the cDNA obtained from the equivalent RNA preparation of the wild type isogenic strain *S. coelicolor* M145. Thresholds of probability values (p values) below 0.05 and fold change above 2 (two fold or higher positively induced expression) or below −2 (two fold or lower negatively induced expression) were used to select differential hybridisation spot results. The results obtained for the deficient and overproducer strains at the late exponential phase of growth are summarised in Tables S1 and S2, respectively. Hybridisation data at later phases of growth were very dispersed (not shown), probably due to bacterial heterogeneity, as observed in other bacterial strains [9], and therefore, transcriptional analyses were centred on the late exponential phase of growth. A discrete number of genes were affected in their respective levels of expression in each case. Thus, the regulator deficiency seemed to cause the activation of 40 genes and the number of down regulated ones only rose to 16. Sixty genes were down regulated in the bacterial cells oversynthesising the response regulator, while only 15 genes resulted up regulated in the same strain, from which more than 50% are annotated to encode possible secretory proteins.

When the transcriptome profiles of the overproducer and deficient strains were compared, 15 down regulated genes in the overproducer strain were up regulated in the regulator deficient strain (Table 1). This figure increased to 40 genes when overall transcriptional organisation (potential operons and clusters) of the different genes was considered, clearly showing the contrary effect of the regulator deficiency versus its overproduction. In the overproducer strain a significant number of down regulated genes encode ribosomal proteins or proteins related to potential stress responses. Interestingly, most of these genes resulted up regulated when *SCO5785* expression was impaired in the deficient strain.

**Table 1:** Genes down regulated in *S. coelicolor* M28 and up regulated in *S. coelicolor* I32.

Gene	Ratio M28/wt	Ratio I32/wt	Operon/cluster	Annotated function
**Primary metabolism**				
SCO2076	−2.07	2.13	SCO2076	Possible isoleucyl-tRNA synthetase
SCO2618	−2.41	2.75	SCO2619-2617*	ClpP2, ATP dependent Clp protease
				proteolytic subunit 2
SCO4704	−2.03	3.77	SCO4701-4721*	RplW, 50S ribosomal protein L23
SCO4706	−2.25	4.09	SCO4701-4721*	RpsS, 30S ribosomal protein S19
SCO4707	−2.22	2.75	SCO4701-4721*	RplV, 50S ribosomal protein L22
SCO4710	−2.32	5.30	SCO4701-4721*	RpmC, 50S ribosomal protein L29
SCO4713	−2.09	3.42	SCO4701-4721*	RplX, 50S ribosomal protein L24
SCO4714	−2.15	4.28	SCO4701-4721*	RplE, 50S ribosomal protein L5
SCO4716	−2.13	3.30	SCO4701-4721*	RpsH, 30S ribosomal protein S8
SCO4719	−2.04	2.34	SCO4701-4721*	RpsE, 30S ribosomal protein S5
SCO4726	−2.04	2.14	SCO4724-4731*	RpmJ, 50S ribosomal protein L36
SCO4956	−2.02	2.31	SCO4956	Possible peptide methionine
				sulfoxide reductase
SCO5999	−2.63	2.75	SCO5999	Hypothetical protein
**Secretory proteins**				
SCO6109	−2.18	2.19	SCO6108-6109	Possible secreted hydrolase
**Other genes**				
SCO4253	−2.27	2.36	SCO4253-4251	Hypothetical protein

*Transcriptional units potentially ppGpp regulated [10]. M28/wt indicates the ratio between the regulator overproducer strain (*S. coelicolor* M28) and its isogenic strain (*S. coelicolor* M145 [pIJ487]). I32/wt indicates the ratio between the regulator deficient strain *(S. coelicolor* I32) and its isogenic strain (*S. coelicolor* M145).

Down regulation of ribosomal genes has been reported to form part of a stringent response in *S. coelicolor* [10]. In *S. coelicolor*, RelA appears to be the only source of ppGpp synthesis [11], [12]. The expression of *relA* was measured by qRT-PCR analysis and found to be up regulated (fold change 3.86) in the strain overproducing SCO5785, as expected, while it was slightly down regulated in the *SCO5785* deficient strain (fold change −2.11), where the expression of ribosomal genes was restored. The expression of a total of twenty-two ppGpp-dependent genes [10] appears to be regulated by SCO5785, as determined by microarray hybridisation analysis, a figure that increased to fifty-five genes, when overall transcriptional organisation (potential operons and clusters) of the different genes was taken into account (Tables S1 and S2).

No change in the expression level of the *SCO5785* gene was detected by microarray hybridisation analyses, although *SCO5785* transcription was up regulated in the strain carrying *SCO5785* in high copy number when analysed by qRT-PCR (fold change 30.97), and the *SCO5785* transcriptional level appeared to be reduced in the deficient strain when estimated by qRT-PCR (fold change −32.11).

### Effect of the response regulator on antibiotic production and sporulation

**Figure 1:**
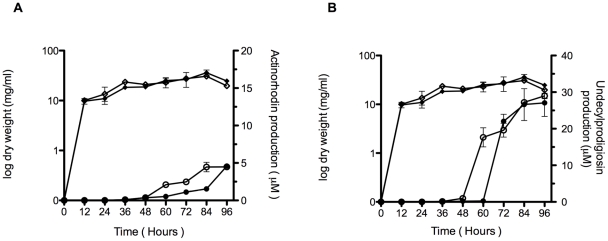
Antibiotic production in the deficient strain. (A) Growing cultures and actinorhodin production of *S. coelicolor* I32 (black circles) or its isogenic *S. coelicolor* M145 (white circles) in R5 liquid medium at 30°C. (B) Growing cultures and undecylprodigiosin production of *S. coelicolor* I32 (black circles) or its isogenic *S. coelicolor* M145 (white circles) in R5 liquid medium at 30°C. The data presented are the average of at least three independent determinations; the bars show standard error.

**Figure 2:**
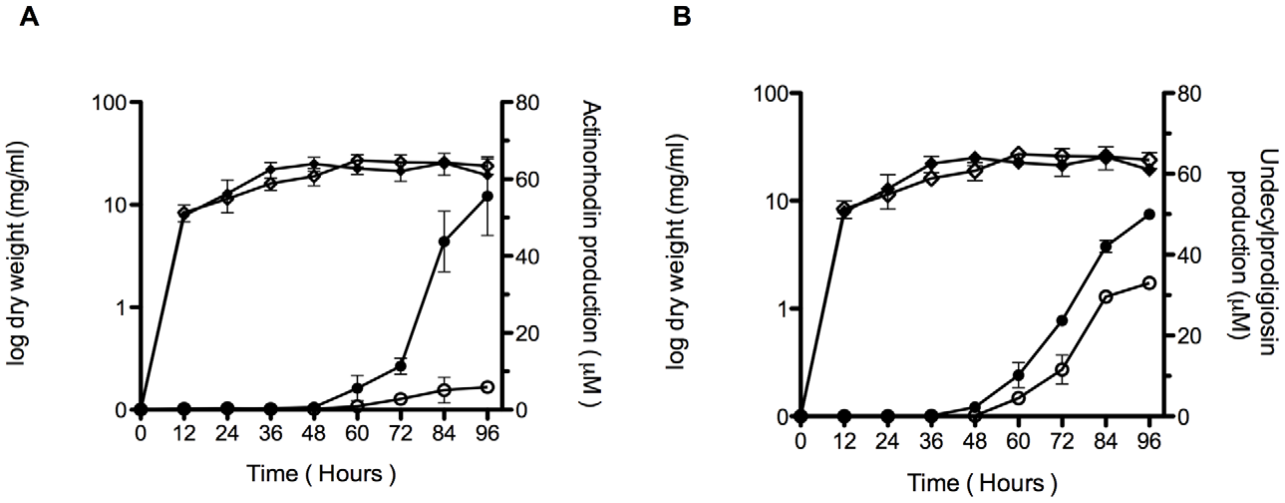
Antibiotic production in the SCO5785 overproducer strain. (A) Growing cultures and actinorhodin production of *S. coelicolor* M28 (black circles) or its isogenic *S. coelicolor* M145 [pIJ487] (white circles) in R5 liquid medium at 30°C. (B) Growing cultures and undecylprodigiosin production of *S. coelicolor* M28 (black circles) or its isogenic *S. coelicolor* M145 [pIJ487] (white circles) in R5 liquid medium at 30°C. The data presented are the average of at least three independent determinations; the bars show standard error.

The synthesis of the blue-pigmented antibiotic actinorhodin occurred later in the deficient strain than in the wild type strain when incubated in solid medium, and propagation of the *SCO5785* gene in multicopy apparently resulted in a higher production of actinorhodin at the earlier stages of growth relative to the isogenic strain carrying a single copy of the regulator gene (not shown). Actinorhodin production has been reported to increase when *S. coelicolor* cells were grown in liquid R5 medium [13]; therefore, the relative production of this antibiotic during growth of the cultured cells was determined. Figure 1A shows the difference in the relative production of actinorhodin between the deficient and the wild type strains. Actinorhodin production accumulates earlier in the wild type, although the final amount seems to be almost the same in both strains. Figure 2A shows the different level of actinorhodin synthesis in cells carrying the regulator gene in high copy number versus the isogenic strain. Actinorhodin synthesis started earlier in the regulator overproducer strain and accumulated to higher levels than in the strain carrying the regulator gene in single copy. In this respect, the *actII-4* gene encoding the actinorhodin cluster activator was found to be up regulated in the regulator overproducer strain when its transcription was analysed by qRT-PCR (fold change 2.28), as well as the *act* cluster gene *SCO5087* encoding the actinorhodin polyketide beta-ketoacyl synthase (fold-change 31.77). Both genes were down regulated in their relative level of expression in the deficient strain (fold change −2.34 and −11.34, respectively). Synthesis of the antibiotic produced by *S. coelicolor*, undecylprodigiosin, was also measured in the deficient and regulator overproducer strains. An equivalent result to that of acrtinorhodin was obtained for undecylprodigiosin production in both the deficient and overproducer strains when compared to their respective isogenic wild type strains, both in solid (not shown) and liquid media (Fig. 1B and 2B, respectively). The undecylprodigiosin cluster activator gene *redZ* was up regulated in the overproducer strain and down regulated in the deficient strain when analysed by qRT-PCR (fold change 2.10 and −4.62, respectively), as well as the gene *redX* encoding the poliketide synthase (fold change 10.41 and −13.13, respectively).

**Figure 3:**
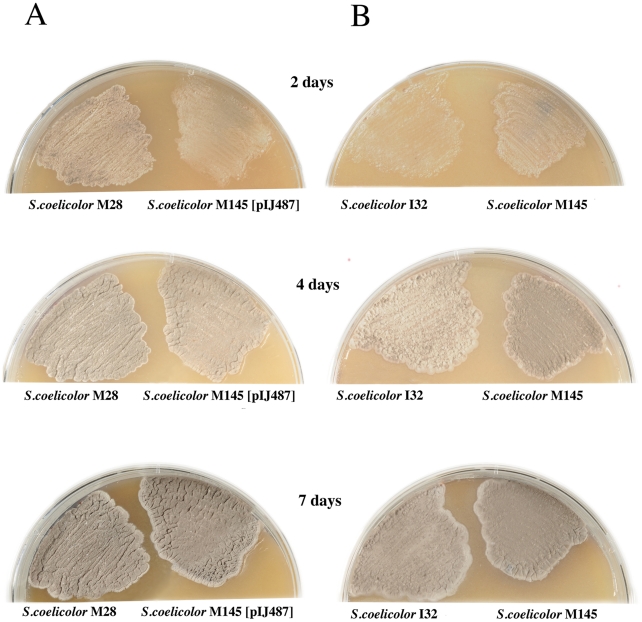
Sporulation of the SCO5785 deficient and overproducer strains. (A) The strain carrying the regulator in multicopy (*S. coelicolor* M28) showed a premature sporulation phenotype when compared to the isogenic strain, while the deficient strain (*S. coelicolor* I32) appeared to have delayed sporulation when compared to that of the wild type strain (B). Plates were incubated in MS medium at 30°C at the indicated times.

The strain carrying the regulator in multicopy showed a premature sporulation phenotype, while the deficient strain appeared to have delayed sporulation when propagated in MS solid medium (Fig. 3). Propagation of the muticopy plasmid carrying the regulator gene in the strain containing the disrupted operon restored the observed deficiencies, both in sporulation and antibiotic production, acquiring phenotypes analogous to those of the wild type strain carrying the regulator gene in multicopy (not shown).

### Effect of the response regulator overproduction on secretory proteins

Genes encoding secretory proteins were up regulated in the overproducer strain, among these, the gene encoding the subtilisin inhibitor (*SCO0762*; *sti*, up regulated 5.88 fold; Table S1, qRT-PCR fold change, 189.9). The subtilisin inhibitor is a major protein present in *S. lividans* supernatants [14] and, in good agreement with the transcriptomic and proteomic analyses, its activity was found to be higher in *S. coelicolor* cells carrying the regulator in high copy number than in the isogenic strain, as expected (Fig. S1A). Therefore, extracellular proteins from exponentially growing cell cultures of the overproducer strain and its isogenic strain were precipitated and subjected to 2D-PAGE gel electrophoresis, where the amount of protein loaded onto the 2D gels was corrected by the dry weight of the bacterial cultures. The overall 2D-PAGE extracellular protein patterns of both strains showed the overproduction of Sti, being clearly distinguishable from the extracellular proteins in the overproducer strain, in good agreement with the transcriptional analysis (Fig. S1B). No differences were apparently observed when the overall extracellular pattern of the wild type or the deficient strains were analysed in 2D gels (Fig. S1C).

To gain further insight into the major secretome components of the overproducer strain and its isogenic strain, samples from exponentially growing cell cultures from both strains were subjected to isotope coded protein labelling (ICPL), followed by high-performance liquid chromatography/electrospray ionization tandem mass spectrometry (HPLC/ESI-MS/MS). Among the differential proteomics methods based on stable isotopic labelling, ICPL is a non-isobaric technique devised to efficiently label primary amines found in proteins. Recent studies have shown ICPL to be a reliable technique for relative quantification at the protein [15] and peptide level [16].

The amount of non-secretory proteins present in the different supernatants (Tables S3 and S4) was significant and increased at later growth; the level of lyses increases with the stage of growth, and therefore, the extracellular presence of intracellular protein increases as well, as already described [17], [18]. Lipoproteins exported and translocated outside the bacterial membrane, together with membrane proteins, have been reported to form part of bacterial secretomes [14], due to their occasional release outside of the bacterial cell. However, it is difficult to assess whether their differential presence in the overproducer supernatant with respect to the isogenic supernatant is due to a specific regulation of their respective genes, or simply to a higher or lower accidental release from the membrane. Therefore, on analysing the secretomes, special attention has been paid to the polypeptides already reported to be secretory proteins or to those carrying potential type I leader peptides. SignalP 3.0, TatP 1.0 and LipoP 1.0 algorithms were used to predict the presence and location of type I, type I (twin-arginine) or type II signal peptides, respectively, in the identified polypeptides.

Seven polypeptides reproducibly showed a score above the cut-off value (Table 2), from which six have been annotated as encoding potential secretory proteins, one of them having a predicted type I (twin-arginine) leader peptide, and the remaining one was predicted to have a cytoplasmic location but experimentally found to be extracellular (Table 3) [18]. Among the polypeptides with a reproducible score below the cut-off value, only two could be considered secretory proteins. The transcriptional regulation of the genes encoding the differentially secreted proteins was assessed by qRT-PCR analyses. Table 2 summarises the results of these analyses. Some secretory proteins, whose genes could appear up regulated by microarray analysis, might not be synthesised and secreted in such an amount that tandem mass spectrometry would be able to detect them above the cut-off point for protein differential abundance (*SCO3222, SCO3286, SCO3607, SCO7657*). In addition, genes from secretory proteins, which are normally largely synthesised by the cell, may not be detected as up regulated by microarray analysis, therefore, their relative level of expression would need to be revealed by qRT-PCR analysis (*SCO0297, SCO1860*). The same rationale applies to genes encoding secretory proteins that are extracellularly present below the cut-off point for protein differential abundance (*SCO6109, SCO1230, SCO4561*). Combining the transcriptional and proteomic analyses, the number of up regulated genes encoding secretory proteins amounted to eleven. The transcriptional analysis of the regulator deficient strain basically showed no regulatory effects on genes encoding the secretory proteins, and mass spectrometry of the ICPL extracellular polypeptides confirmed that no secretory proteins were found to reproducibly score above or below the cut-off values, as expected (not shown).

**Table 2:** Genes encoding secretory proteins modulated by SCO5785.

Gene	Ratio arrays	Ratio ICPL	Ratio qRTPCR	Operon/Cluster	Annotated function
**Proteins present above the cut-off value (log2 (Avg(H/L)) ≥1.5**					
SCO0297^2^		3.64	20.68	SCO0297	Possible secreted protein
SCO0762^2^	5.88	4.32		SCO0762	Sti1, secreted subtilisin
					inhibitor
SCO1860		2.35	79.89	SCO1860	Possible secreted protein
SCO5074^3^		2.40	79.71	SCO5074	Possible dehydratase
SCO6197^2^	2.75	2.06		SCO6197	Possible secreted protein
SCO6198^1^	2.15	2.13		SCO6198	Possible secreted protein
SCO6199^2^		1.74	143.01	SCO6199	Possible secreted esterase
**Proteins present below the cut-off value (log2 (Avg(H/L)) ≤−1.5**					
SCO1230		−2.60	−1.47	SCO1230*	Possible secreted
					tripeptidylaminopeptidase
SCO4561		−2.55	−2.88	SCO4561	Possible secreted NLP/P60
					family protein

The results correspond to the mean of at least two independent cultures.

1 Protein predicted to contain a Tat leader peptide and confirmed to use the Tat secretion route instead of the Sec route [18].

2 Proteins previously described as forming part of the *S. lividans* secretome [14].

3 It has been described as equivalent to one forming part of the *act*VI region of the *S. coelicolor* A3(2) actinorhodin biosynthetic cluster [35], [36], although it has been found to be extracellular [18].

*Transcriptional units potentially regulated by ppGpp [10]. qRT-PCR analyses were performed only when microarray hybridisation analyses were not conclusive.

**Table 3:** Leader peptides of the secretory proteins modulated by SCO5785.

Protein	Leader peptide
**Proteins above the cut-off**	
SCO0297	MRSTGPVSRIGRSLAAVTATAAAGAVAALGLAASP**AAAA**
SCO0762	MRNTARWAATLGLTATAVCGPLAGA**SLAS**
SCO1860	MSLMRAPHACLTGGPTTLNGNTFRMPARRLATVAAATALAAGPATLAGAGS**AHAT**
SCO5074	MTSSLHHAIRLTTASAIALGGLVTLGTS**AHAA**
SCO6197	MSTTSVNGSRSKRRVLLRRALPVCVAAGVASIVVFGSGSS**PDAA**
SCO6198^1^	MDTTYWS*RRRVL*TVLGAATAATSIPLAAPSR**ALAA**
SCO6199	MHRTRPGGHPRSIRFATLAISLTAGSALMTASP**AVAV**
**Proteins below the cut-off**	
SCO1230	LRKSSIRRRATAFGTAGALVTATLIAGAVSAPA**ASAA**
SCO4561	MSHTAHIRSHRKPRRSASTIAMRTGVAGGVLSTLAVAGASGA**AHAA**

Putative signal peptidase type I predicted signal peptides are shown. The residues at positions −3 to +1 relative.

to the predicted signal peptidase type I cleavage site are indicated in bold.

1 The conserved Tat motif is indicated in italics in the protein predicted to contain a Tat leader peptide and confirmed to use the Tat secretion route instead of the Sec route [18].

## Discussion

The analysis of the global gene expression profile of the strain carrying the *SCO5785* gene in multicopy (*S. coelicolor* M28), as well as that of the deficient strain (*S. coelicolor* I32) by a combination of proteomic and transcriptomic approaches, has enabled us to characterise the potential regulation exerted by the *S. coelicolor SCO57854-SCO5785* operon.

Different bacteria may react differently to environmental changes, or use different metabolic circuits to reach their final objective, consequently triggering an appropriate adaptive response to those changes. The results obtained suggest that the *S. coelicolor* SCO5784-SCO5785 two-component system may also respond to environmental changes by means of a temporal modulation of the secondary metabolism. This was judged by the moderate down regulation of genes involved in antibiotic biosynthesis and sporulation observed by solid medium and activity analyses in the deficient strain, showing a potential role of the operon in secondary metabolism regulation. This regulation may be transient in the deficient strain, as this effect seems to recover at later phases of growth (Fig. 1, 2 and 3), whereas exactly the contrary appears to occur in the regulator overproducer strain.

The most important system for sensing nutrient starvation and triggering adaptive responses in bacteria involves the phosphorylated nucleotide ppGpp, also known as the “stringent factor”. The SCO5784–5785 two-component system seems to exert its function, at least partially, via the stringent factor, modulating the expression of ribosomal and antibiotic biosynthetic genes, among other ppGpp modulated genes. The *S. coelicolor* stringent response regulates genes involved in central carbon metabolism and purine/pyrimidine biosynthesis, whose regulation is *relA* independent in *B. subtilis* [10], [19]. Hence, this suggests that the synthesis of ppGpp in *S. coelicolor* may have a broader regulatory effect in metabolic processes than in other bacteria. The obtained results certainly help to support this.

The *S. coelicolor* SCO5784–SCO5785 two-component system does share some similarity in its regulatory mode of action with that of the *B. subtilis* DegS-DegU system, in particular, eliciting the synthesis of a discrete number of secretory proteins. Among all possible *S. coelicolor* two-component sensor kinase or response regulator genes, the SCO5784–SCO5785 pair was selected as it does have some degree of homology to its potential *B. subtilis* counterpart and has a similar chromosomal organisation. Experiments are under way to further characterise the mode of action of the *S, coelicolor* SCO5785 response regulator in order to test its potential equivalence to that of the *B. subtilis* DegS-DegU two-component system, and especially, whether the *S. coelicolor* regulator is able to modulate its own operon expression. Propagation of *SCO5785* in multicopy enhances the production of antibiotics as well as secretory proteins. In particular, the increase in the expression level of secretory protein encoding genes, either as an artefactual or real effect of the regulator, could be of potential usefulness when using *Streptomyces* strains as hosts for homologous or heterologous extracellular protein production. More experimental evidence is needed to confirm this potential usefulness in addition to getting further insight on the transient function of this system.

## Materials and Methods

### Bacterial strains, plasmids and media

The *S. coelicolor*A3(2) strain M145 [20], used as the wild-type strain, and its derivatives were cultured in liquid NMMP medium using mannitol as carbon source or in solid R5 medium, as indicated [20]. Thiostrepton (10 µg/ml) or kanamycin (10 µg/ml) was added to the liquid medium, when required. Both antibiotics were added to the solid medium at a concentration of 50 µg/ml, when required. For the sporulation studies, the different strains were set to sporulate in solid MS medium where sporulation takes place more efficiently [21]. *S. coelicolor* M28 is an *S. coelicolor*A3(2) derivative that has been used to propagate the multicopy plasmid pIJ487 carrying the *SCO5785* gene under the control of its own operon promoter. *S. coelicolor* I32 is a *S. coelicolor*A3(2) derivative carrying the disrupted *SCO5784–SCO5785* two genes operon in such a way that the *SCO5785* gene is no longer expressed under the control of the operon promoter. Operon disruption resulted from the insertion of the non-replicative plasmid pAC301 [22], conferring thiostrepton resistance between the sensor and regulator genes.

To construct the *S. coelicolor* M28 strain, oligonucleotides S/U 3.4 d (5′GGAGAATTCGTGCTTTCCCCTCACTCGT 3′) and PR3.4r (5′GGGGGATCCACCGTACGTGCG3′) were used to amplify a 114 nt long DNA fragment from the *S. coelicolor* M145 genome containing the regulatory sequence of the *SCO5784–SCO5785* two genes operon. Oligonucleotides S/U 3.4 r (5′GCGTCTAGAGACCAGCAGTGGCTCACC3′) and DEGU3.4 d (5′GGAGGATCCATGTCCGCACGGCCC3′) were used to amplify a 849 nt long DNA fragment from the *S. coelicolor* M145 genome containing the regulator coding sequence of the *SCO5785* gene. Both fragments were ligated to plasmid pIJ487 linearised with endonucleases *Eco*RI and *Xba*I, and the ligation mixture was used to transform *S. coelicolor* M145 protoplasts. A bacterial strain containing the multicopy plasmid pIJ487 carrying the operon promoter fused upstream of the regulator coding sequences was selected. Another bacterial strain was similarly constructed carrying the regulatory sequence of the *SCO5784–SCO5785* two genes operon in multicopy. This strain was used to control the lack of possible artefactual transcriptional effects potentially due to the propagation of the operon promoter sequences in such a high copy number (results not shown).

To construct the *S. coelicolor* I32 strain, oligonucleotides MD34d (5′GAATTCCCTACAACACGATGCTGGAC3′) and MD34r (5′CCGTCTAGATTGAGGGCCTCGAGA3′) were used to amplify a 1008 nt long DNA fragment from the *S. coelicolor* M145 genome, spanning the *SCO5784* 3′ moiety (the last 695 nt of the coding sequence), followed by the *SCO5784*5 5′ moiety (the first 296 nt of the coding sequence). The amplified fragment was inserted into plasmid pAC301 and the recombinant plasmid used to transform the *S. coelicolor* M145 protoplasts. A thiostrepton resistant strain, *S. coelicolor* I32, with its regulator gene no longer under the control of the operon promoter, as verified by PCR amplification and Southern blot hybridisation analysis, was selected and used as the SCO5785-deficient strain. To avoid *S. coelicolor* methyl-specific restriction mechanisms, recombinant pAC301 derivatives were first propagated in the methylation deficient *E. coli* strain ET12567 (*supE44 hsdS20 ara-14 proA2 lacY galK2 rpsL20 xyl-5 mtl-1* D*dam* D*dcm* D*hsd*M Cm^r^, [23]) before being transferred to *S. coelicolor* M145. *S. coelicolor* M145 [pIJ487] was used as the isogenic strain of *S. coelicolor* M28 and *S. coelicolor* M145 was the *S. coelicolor* I32 isogenic strain. The recombinant plasmid contained in *S. coelicolor* M28 was propagated in *S. coelicolor* I32 to complement its regulator deficiency.

Bacterial cell cultures used for the transcriptional or proteomic analyses were depleted of thiostrepton to avoid possible interferences in gene expression patterns potentially caused by the presence of the antibiotic. The presence of the plasmid carrying the regulator gene was monitored to ensure the maintenance of its relative copy number per cell throughout bacterial growth. The presence of the thiostrepton resistance gene in the deficient strain was monitored by PCR analysis.

### RNA extraction, labelling and hybridisation

Total RNA was extracted from the bacterial growing cultures using the RNeasy mid kit (Qiagen). Bacterial cell lysates were extracted twice with phenol-chloroform before being loaded onto the RNeasy midi columns for RNA purification. Fluorescently labelled cDNA for microarray hybridization was obtained using the SuperScript Indirect cDNA Labelling System (Invitrogen), following the supplier's instructions. Twenty micrograms of RNA were transformed to cDNA with Superscript III reverse transcriptase using random hexamers as primers, including aminoallyl-modified nucleotides in the reaction mixture. After cDNA purification, the Cy3 or Cy5 fluorescent dyes (Invitrogen) were coupled to the amino-modified first-strand cDNA. Labelling efficiency was assessed using a NanoDrop ND1000 spectrophotometer (NanoDrop Technologies). Prior to the hybridisation process, the *S. coelicolor* genome-wide DNA microarrays (Eurogentec, Belgium) were blocked by immersion into a 50 ml Falcon tube containing 5×SSC, 0.1% (w/v) SDS and 1% (w/v) bovine serum albumin, preheated to 42°C. After 45 min at 42°C, the microarrays were washed by being briefly immersed in a Falcon tube containing sterile water at room temperature, followed, when necessary, by another immersion in isopropanol, before being allowed to dry.

Equal amounts of Cy3- or Cy5-labelled cDNAs (about 50 pmoles each), one sample corresponding to the control and the other to the problem under analysis, were mixed and dried in a Speed-Vac. Each sample was dissolved in 45 µl of a solution containing 50% (v/v) deionised formamide, 5× Denhardt's solution, 6× SSC, 0.5% (w/v) SDS, 5% (w/v) dextran sulphate, pre-filtered and pre-heated at 42°C. After 2 min at 90°C to denature the DNA, the solution was applied to the microarray slide and covered with a 24×60 mm cover glass. The slide was introduced into a hybridisation chamber and incubated for 18 h away from the light, following the microarray supplier's instructions. The microarray was then transferred to a Falcon tube containing 0.5× SSPE (1× SSPE contains 150 mM NaCl, 1 mM EDTA, 11.5 mM NaH_2_PO_4_, PH 7.4), 0.5% (w/v) SDA and pre-heated to 37°C. After removing the cover glass, the microarray was washed by gentle shaking for 5 min. The slide was subsequently transferred to a new tube containing 0.5× SSPE and 0.5% (w/v) SDS and washed again by gentle shaking for 5 min at room temperature. Similar washes with 0.5× SSPE were conducted three more times, followed by a final wash with 0.1× SSPE at room temperature. The microarray was allowed to dry and scanned in a microarray scanner with green and red lasers operating at 532 and 635 nm, respectively, to excite the Cy3 and Cy5. Images were taken at 10 µm resolution and spot intensity was determined using the Genepix Pro 5.0 (Axon) software package.

Hybridisation data were statistically analysed using LIMMA [24] software. Three independent RNA extractions were made for each experiment at two different bacterial cell culture growing times, the corresponding microarray analyses were performed and the information provided by three biological replicas combined in each case. The results for each replica (median intensity for each channel) were normalised and statistically analysed using the LIMMA software package [24]. Background subtraction was performed using a method implemented in LIMMA designed to yield positive corrected intensities (i.e. to avoid negative intensity values). A convolution of normal and exponential distributions was fitted to the foreground intensities using the background intensities as covariate. This results in a smooth monotonic transformation of the background subtracted intensities in such a way that all the corrected ones are positive. Differential hybridisation was calculated using linear models and empirical Bayes moderated *t*-statistics [24], [25]. The resulting log-ratios were normalised for each array through print-tip loess [25] and differential hybridisation values were scaled to achieve consistency among arrays. Each probe was tested for changes in differential hybridisation over replicates by using moderated *t*-statistics [24]. The p-values were adjusted for multiple testing, as described [26], to control the false discovery rate. The output file provides the fold-change and p-values for each spot, among other data. Comparisons were performed using the Venn algorithm (http://www.pangloss.com/seidel/Protocols/venn.cgi). Operon prediction was initially carried out using the Microbesonline website (1; http://microbesonline.org).

### Microarray data accession number

The microarray data presented in this paper have been registered in the NCBIGEO data bank (www.ncbi.nlm.nih.gov/projectsgeo: accession numbers GSM570831–GSM570836).

### Quantitative real time PCR

DNA potentially contaminating the RNA preparations was removed by incubation with RNase-free DNAse (Ambion) and its absence was tested by quantitative real time PCR amplification in the absence of reverse transcriptase. Complementary DNA was synthesised using the High Capacity Archive kit (Applied Biosystems). Quantitative real time PCR (qRT-PCR) was performed using SYBR Green technology in an ABI Prism 700 Sequence Detection System (Applied Biosystems). Samples were initially denatured by heating at 95°C for 10 min. A 40-cycle amplification and quantification program was then followed (95°C for 15 sec and 60°C for 1 min) by a single fluorescence measurement per cycle, according to the manufacturer's recommendations. A final extension cycle (72°C, 1 min) was performed. Three biological samples from the different bacterial cultures were amplified in triplicate in separate PCR reactions. All PCR products were between 50 and 150 bp in length.

A melting curve analysis was conducted after amplification to distinguish the targeted PCR products from the non-targeted ones. The melting curves were obtained by slow heating at temperatures ranging from 60°C to 95°C at a rate of 0.2°C per sec, with continuous fluorescence scanning. Oligonucleotides HRDBD (5′-CGCGGCATGCTCTTCCT-3′) and HRDBR (5′-AGGTGGCGTACGTGGAGAAC-3′) were used for the amplification of the *hrdB* transcript carried out as an internal control to quantify the relative expression of target genes [27]. The oligonucleotides used as primers to amplify other transcripts are indicated in Table S5.

The gene encoding the major vegetative sigma factor (*hrdB*) has been used as a reference gene for qRT-PCR analyses. No differences in the transcriptional level of this gene were detected by microarray hybridisation analysis after induction of ppGpp synthesis in *S. coelicolor*, although the gene appeared to be slightly repressed when its level of expression was analysed by qRT-PCR [10]. We have not seen any differences in the expression level of *hrdB* in the *S. coelicolor* M28, *S. coelicolor* I32 or wild type cultures when analysed by microarray hybridisation or qRT-PCR (not shown), and *hrdB* has been used as a reference gene.

### Extracellular protein analysis

Standard extracellular protein analyses were essentially carried out, as described [14]. Supernatants from cells grown in NMMP medium were collected by centrifugation at 1400× g for 10 minutes. One volume of 10% TCA was added to the supernatant and the mixture was incubated at −20°C for one hour to precipitate the extracellular proteins. The proteins were then separated by centrifugation at 15000× g for 20 minutes at 4°C. Protein pellets were washed twice with ice cold acetone and any residual acetone was removed by air-drying. Protein pellets were resuspended in 10 mM Tris-HCl pH 8, 1 mM EDTA, 1% SDS and total extracellular proteins were visualised by Coomasie blue stained sodium dodecyl sulphate-polyacrylamide gel electrophoresis (SDS-PAGE) on a 12% polyacrylamide gel [28]. The protein concentration in the different samples was determined using the BCA protein assay kit (Pierce), as indicated by the supplier.

The 2D electrophoresis was performed as described [29], [30], using gradient Immobiline Dry Strips pH 4–7 (11 cm; Amersham Biosciences) for the first dimension (IEF). Purified proteins (30 µg) were carried to a 200 µl dehydration buffer (7 M urea, 2 M thiourea, 4% CHAPS, 0,5% IPG buffer pH 4–7 and 50 mM DTT and bromophenol blue traces) and loaded onto the IPG strip. Isoelectric focusing was performed in IPGPhor-I (Amersham Biosciences) to reach 8000 vh in total.

After the separation, the first dimension strip was equilibrated twice with equilibration buffer (50 mM Tris-HCl pH 8, 8.6 M urea, 30% glycerol, 2% SDS and bromophenol blue traces) in the presence of 1% DTT in the first equilibration and 4% iodoacetamide in the second one. SDS-PAGE in the second dimension was performed in 12.5% polyacrylamide gels. After electrophoresis the gels were stained with MALDI TOF compatible silver nitrate, as described [31].

### Tandem mass spectrometry

Extracellular proteins were submitted to in-solution digestion with trypsin, as described [32]. Tryptic peptides were purified with 100 µl C18 tips (Varian) in order to eliminate urea, and mainly NH_4_HCO_3_, which would compete with peptides for the labelling reagent, and then isotopically labelled with ICPL reagent, according to the manufacturer's instructions (Serva). To ensure reproducibility, technical replicates were carried out interchanging the light and heavy isotopes in the labelling procedure.

In order to increase peak capacity and resolution, the labelled peptide mixture was analysed by 2D-HPLC-MS/MS. The first dimension consisted of a separation by reverse-phase chromatography at basic pH on a Smartline HPLC (Knauer), using a Fortis C18 column 100×2.1 mm, 5 µm particle size (Fortis Technologies) at a flow-rate of 150 µl/min. Mobile phase A was 10 mM NH_4_OH in water (pH 9.4), and B was 20% water, 10 mM NH_4_OH in methanol (pH 9.4). The gradient elution conditions were as follows: 0–5 min: 2% B; 5–15 min: 25% B; 15–55 min: 70% B; 55–60 min: 100% B; 60–65 min: 100%; 65–67 min: 2%; 67–85 min: 2% B. Seven fractions were collected, taken to dryness in a speed-vacuum system and off-line injected in the tandem mass spectrometry system.

HPLC-ESI-MS/MS analysis was performed on an Ultimate 3000 nano HPLC (Dionex, Sunnyvale, California) coupled to an HCT Ultra ion-trap mass spectrometer (Bruker Daltonics, Bremen, Germany), using a silica-based reverse-phase column C18 PepMap 75 µm×15 cm, 3 µm particle size and 100 Å pore size (Dionex), and a trapping column C18 PepMap 5 mm×300 µm, 5 µm, 100 Å (Dionex) at a flow-rate of 300 nl/min. Mobile phase A was 0.1% formic acid in water, and B was 20% water, 0.1% formic acid in acetonitrile. The gradient elution conditions were as follows: 0–5 min: 4% B; 5–95 min: 40% B; 95–96 min: 90% B; 100–101 min: 4% B; 101–120 min: 4% B.

The LC system was coupled via a nanospray source to the ion trap mass spectrometer operating in positive ion mode. An automatic data-dependent acquisition method was used, selecting the four most abundant ions for isolation and fragmentation from the MS scan range of 350–1000 m/z. Dynamic exclusion was set to 1.0 min after 2 spectra in order to prevent the same ion from isolation. Next, all these single HPLC-MS/MS runs were merged using the Analysis Combiner tool before being processed as a single experiment. Processed files in the form of Mascot Generic Files were analyzed using WARP-LC 1.1 (Bruker Daltonics) for their qualitative and quantitative analysis. For protein identification, HPLC-ESI-MS/MS spectra were searched against the NCBI nonredundant protein sequence database 20090406 (http://www.ncbi.nlm.nih.gov/), using a licensed version v.2.2.04 of the Mascot search engine (Matrix Science). Search parameters were set as follows: taxonomy = *Streptomyces coelicolor*; carbamidomethylated cysteins = fixed modification; oxidized methionines = variable modification; ICPL-light (+105.02 Da) and -heavy (+111.04 Da) labelled lysine residues = variable modification; ICPL-light (+105.02 Da) and -heavy (+111.04 Da) labelled amino termini = variable modification. ICPL pairs were determined considering a mass tolerance of 0.5 Da and a retention time tolerance of 40 seconds. For quantitative analysis, extracted ion chromatograms of those peptides identified with Mascot scores ≥25 were considered and relative peptide quantification was assessed according to the intensity ratio of the monoisotopic signals. Only proteins quantified with at least two peptides were taken into account to assure the certainty of the quantitative data. For the statistical analysis, all the data were converted in the log space to maintain symmetry around zero. The cut-off point for protein differential abundance was set to log2 (Avg(H/L)) ≥1.5 or ≤−1.5. That is, proteins whose relative abundance deviated 1.5 fold above or below the comparative average value with the isogenic strain were selected. SignalP 3.0, LipoP 1.0, and TatP 1.0 (http://www.cbs.dtu.dk/services) were used to predict the presence and type of the extracellular protein leader peptides.

### Extracellular enzyme activities and antibiotic production

To determine the extracellular activities, supernatants from 20 ml aliquots of bacterial cell cultures at the indicated phases of growth were concentrated by precipitation with ammonium sulphate brought to 80% saturation. The precipitated protein was then collected by centrifugation at 13000× g for 30 min and dissolved in 0.01 M Tris-HCl pH 8. The total amount of protein present in the assay was determined using the BioRad kit (Pierce), as indicated by the supplier. To assay the extracellular presence of the subtilisin inhibitor, aliquots were brought to a 250 µl final volume of 0.01 M Tris- HCl pH 8.6 in the presence of 2.85×10^−4^ U of subtilisin (Sigma Chemical Co.) and 0.25 mM of the N-succinyl-L-Ala-L-Ala-L-Pro-L-Phe-p-nitroanilide (sAAPF-pNA) (Sigma Chemical Co.) as substrate, and the mixture was incubated at 25°C until the yellow colour developed, as described [33]. The presence of the subtilisin inhibitor was referred to as a percentage of subtilisin activity remaining after the incubation period.

To monitor antibiotic production, the bacterial strains were cultured in rich R5 liquid medium, their respective mycelia were harvested at the indicated times, and the production of actinorhodin and undecylprodigiosin was monitored throughout cellular growth, as described [34].

## Supporting Information

Figure S1 **Subtilisin inhibitor production and overall pattern of extracellular proteins.** (A) Subtilisin inhibitor activity in cultures of *S. coelicolor* M28 (dark blocks) and its isogenic wild type strain (light blocks) grown in minimal medium. Values are given as a percentage of residual subtilisin activity in the assay. Total extracellular protein from exponentially growing cultures of *S. coelicolor* M28 (B) or its isogenic wild type strain (C) in minimal medium was fractionated by 2D-PAGE. The amount of protein loaded onto the gels was corrected by the cultures' dry weight. Arrows indicate the presence of the subtilisin isoforms.
(TIF)

Table S1 **Genes modulated by** ***SCO5785*** **propagation in high copy number.** Transcriptional units potentially ppGpp regulated [10] are underlined. *Genes up regulated in the strain carrying the disrupted *SCO5784–SCO5785* operon.
(DOC)

Table S2 **Genes modulated by disruption of the** ***SCO5784–5785*** **operon.** Transcriptional units potentially ppGpp regulated [10] are underlined. *Genes down regulated in the strain carrying the *SCO5785* gene in multicopy.
(DOC)

Table S3 **ICPL analysis of extracellular proteins of** ***S. coelicolor*** **M28 labelled with C13.** Extracellular proteins of *S. coelicolor* M145 were labelled with C12 and those of *S. coelicolor* M28 were labelled with C13 at 24 h of growth.
(DOC)

Table S4 **ICPL analysis of extracellular proteins of** ***S. coelicolor*** **M28 labelled with C12.** Extracellular proteins of *S. coelicolor* M145 were labelled with C13 and those of *S. coelicolor* M28 were labelled with C12 at 24 h of growth.
(DOC)

Table S5 **Oligonucleotide primers used for gene transcript amplification.** Oligonucleotide sequences start and terminate at their 5′ and 3′ ends, respectively.
(DOC)
