# Supplementary figures and images for: A Novel Two-Component System Involved in the Transition to Secondary Metabolism in Streptomyces coelicolor
Source: PLoS One. 2012 Feb 9;7(2):e31760. doi: 10.1371/journal.pone.0031760 (PMC3276577; doi:10.1371/journal.pone.0031760)

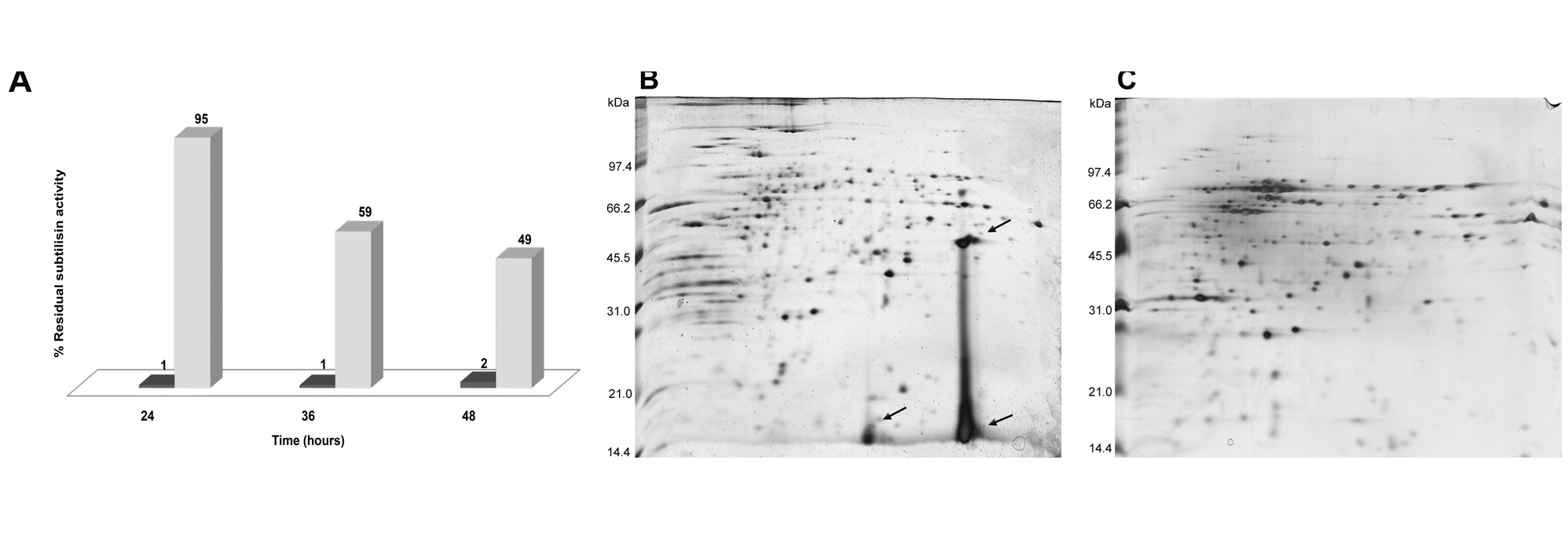

Supplement: Figure S1 — Subtilisin inhibitor production and overall pattern of extracellular proteins. (A) Subtilisin inhibitor activity in cultures of S. coelicolor M28 (dark blocks) and its isogenic wild type strain (light blocks) grown in minimal medium. Values are given as a percentage of residual subtilisin activity in the assay. Total extracellular protein from exponentially growing cultures of S. coelicolor M28 (B) or its isogenic wild type strain (C) in minimal medium was fractionated by 2D-PAGE. The amount of protein loaded onto the gels was corrected by the cultures' dry weight. Arrows indicate the presence of the subtilisin isoforms. (TIF) [file pone.0031760.s001.tif]
