# Supplementary material for: A Novel Two-Component System Involved in the Transition to Secondary Metabolism in Streptomyces coelicolor
Source: PLoS One. 2012 Feb 9;7(2):e31760. doi: 10.1371/journal.pone.0031760 (PMC3276577; doi:10.1371/journal.pone.0031760)
Supplement: Table S1 — Genes modulated by SCO5785 propagation in high copy number. Transcriptional units potentially ppGpp regulated [10] are underlined. *Genes up regulated in the strain carrying the disrupted SCO5784–SCO5785 operon. (DOC) [file pone.0031760.s002.doc]

**Table S1.**  **Genes modulated by *SCO5785* propagation in high copy number** _______________________________________________________________________________

**Gene** **Ratio**  **Transcriptional Annotated function**

**M28/wt organisation**

_______________________________________________________________________________

**Up regulated genes**

Secondary metabolism

SCO3218 2.69 SCO3218 Hypothetical protein, similar to others located within antibiotic synthesis clusters. MbtH-like protein

SCO7659 2.34 SCO7659 Possible oxidoreductase, similar to *Bacteroides fragilis* tetracycline resistance protein

TetX

Secretory proteins

SCO0762 5.78 SCO0762 Sti1, secreted subtilisin inhibitor

SCO2412 2.12 SCO2422-2412 Possible membrane protein. Last gene of a large operon containing seven genes encoding possible secreted proteins

SCO3222 2.16 SCO3222 Possible secreted protein (CDA cluster)

SCO3286 2.0 SCO3286-3285 Possible secreted protein

SCO3607 2.01 SCO3607 Possible secreted protein

SCO6197 2.75 SCO6197 Possible secreted protein

SCO6198 2.16 SCO6198 Possible secreted protein

SCO7657 9.70 SCO7657 Possible secreted protein

Transcriptional regulators

SCO0712 2.73 SCO0712 LipR, Transcriptional activator

Other genes

SCO1181 2.04 SCO1181-1182 Hypothetical protein

SCO3635 2.10 SCO3635 Hypothetical protein

SCO4036 3.19 SCO4037-4036 Hypothetical protein

SCO7738 2.0 SCO7738 Hypothetical protein

**Down regulated genes**

Primary metabolism

SCO0179 -2.79 SCO0179 Zinc-containing dehydrogenase

SCO0212 -3.20 SCO0213-0212 Hypothetical protein

SCO0213 -3.07 SCO0213-0212 Possible nitrate/nitrite transporter protein

SCO0216 -2.18 SCO0216-0219 NarG2, nitrate reductase alpha chain

SCO0217 -2.31 SCO0216-0219 NarH2, nitrate reductase beta chain

SCO0218 -4.05 SCO0216-0219 NarJ2, nitrate reductase delta chain

SCO0219 -3.63 SCO0216-0219 NarI2, nitrate reductase gamma

Chain

SCO0922 -2.37 SCO0924-0922 Possible reductase iron-sulphur protein

SCO0923 -2.03 SCO0924-0922 Reductase flavoprotein subunit

SCO0924 -2.02 SCO0924-0922 Cytochrome B subunit

SCO0999 -4.35 SCO0999 SodF2, superoxide dismutase

SCO1898 -2.20 SCO1898-1901 Sugar binding lipoprotein

SCO2076* -2.07 SCO2076 Possible isoleucyl-tRNA synthetase

SCO2150 -2.21 SCO2151-2148 QcrC, cytochrome C heme- binding subunit

SCO2154 -2.05 SCO2156-2153 Possible integral membrane protein

SCO2155 -2.12 SCO2156-2153 Cox1, cytochrome c oxidase subunit I

SCO2156 -2.08 SCO2156-2153 Cox2, cytochrome c oxidase subunit II

SCO2181 -2.34 SCO2181 SucB, possible dihydrolipoamide succinyltransferase

SCO2519 -3.13 SCO2519-2517 Possible membrane protein. SCO2518 and SCO2517 are a possible two-component system

SCO2618* -2.41 SCO2619-2617 ClpP2, ATP dependent Clp protease proteolytic subunit 2

SCO2633 -3.80 SCO2633 SodF, superoxide dismutase

SCO2643 -2.14 SCO2643 PepN, aminopeptidase N

SCO3428 -2.32 SCO3428-3426 RpmG, 50S ribosomal protein L33

SCO3906 -2.27 SCO3906 PsF, 30S ribosomal protein S6

SCO4704* -2.03 SCO4701-4721 RplW, 50S ribosomal protein L23

SCO4706* -2.25 SCO4701-4721 RpsS, 30S ribosomal protein S19

SCO4707* -2.22 SCO4701-4721 RplV, 50S ribosomal protein L22

SCO4710* -2.32 SCO4701-4721 RpmC, 50S ribosomal protein L29

SCO4713* -2.09 SCO4701-4721 RplX, 50S ribosomal protein L24

SCO4714* -2.15 SCO4701-4721 RplE, 50S ribosomal protein L5

SCO4716* -2.13 SCO4701-4721 RpsH, 30S ribosomal protein S8

SCO4719* -2.04 SCO4701-4721 RpsE, 30S ribosomal protein S5

SCO4726* -2.04 SCO4724-4731 RpmJ, 50S ribosomal protein L36

SCO4727 -2.04 SCO4724-4731 RpsM, 30S ribosomal protein S13

SCO4855 -2.20 SCO4858-4855 DhsB, succinate dehydrogenase iron-sulfur subunit

SCO4956* -2.02 SCO4956 Possible peptide methionine sulfoxide reductase

SCO4974 -2.40 SCO4974 Possible deaminase

SCO4979 -2.34 SCO4979 Possible phosphoenolpyruvate carboxykinase

SCO5178 -2.45 SCO5178 MoeB, possible sulphurylase

SCO5470 -2.31 SCO5472-5469 GlyA2, probable serine hydroxymethyltransferase

SCO5471 -2.73 SCO5472-5469 GcvH, glycine cleavage system H protein

SCO5805 -2.53 SCO5805 NrdJ, ribonucleotide reductase

SCO5897 -2.15 SCO5877-5898 RedG, probable oxidase

SCO5999* -2.63 SCO5999 Hypothetical protein

Secondary metabolism

SCO0107 -2.09 SCO0107 Possible aminoglycoside nucleotidyltransferase (drug resistance)

SCO3899 -2.35 SCO3899 Hypothetical protein (streptomycin biosynthesis)

SCO6764 -2.36 SCO6762-6769 Possible squalene-hopene cyclase

SCO6766 -2.11 SCO6762-6769 Hypothetical protein

Cellular stress

SCO0527 -2.11 SCO0527 ScoF, cold shock protein

Secretory proteins

SCO6109* -2.18 SCO6108-6109 Possible secreted hydrolase

Transcriptional regulators

SCO0168 -2.60 SCO0618 Possible regulator protein

Other genes

SCO0169 -2.49 SCO0169 Hypothetical protein

SCO0200 -3.13 SCO0200 Hypothetical protein

SCO4252 -2.09 SCO4253-4251 Hypothetical protein

SCO4253* -2.27 SCO4253-4251 Hypothetical protein

SCO4509 -2.08 SCO4509 Hypothetical protein

SCO4822 -2.53 SCO4822 Possible integral membrane protein

SCO5555 -2.27 SCO5555 Hypothetical protein

SCO5638 -2.07 SCO5639-5638 Possible integral membrane protein

SCO5650 -2.37 SCO5650 Possible membrane protein

_______________________________________________________________________________

Transcriptional units potentially ppGpp regulated [10] are underlined. *Genes up regulated in the strain carrying the disrupted *SCO5784-SCO5785* operon.
