# Supplementary material for: A Novel Two-Component System Involved in the Transition to Secondary Metabolism in Streptomyces coelicolor
Source: PLoS One. 2012 Feb 9;7(2):e31760. doi: 10.1371/journal.pone.0031760 (PMC3276577; doi:10.1371/journal.pone.0031760)
Supplement: Table S2 — Genes modulated by disruption of the SCO5784–5785 operon. Transcriptional units potentially ppGpp regulated [10] are underlined. *Genes down regulated in the strain carrying the SCO5785 gene in multicopy. (DOC) [file pone.0031760.s003.doc]

**Table S2. Genes modulated by disruption of the *SCO5784-5785* operon** _________________________________________________________________________________

**Gene Ratio Transcriptional Annotated function**

**I32/wt organisation**

_________________________________________________________________________________

**Up regulated genes**

Primary metabolism

SCO1088 2.29 SCO1089-1087 Possible oxidoreductase

SCO1491 2.32 SCO1491-1490 Efp, elongation factor P

SCO1814 2.10 SCO1815-1814 InhA, possible enoyl-(acyl-carrier protein) reductase

SCO1864 2.70 SCO1864-1866 Possible acetyltransferase

SCO2076* 2.13 SCO2076 Possible isoleucyl-tRNA synthetase

SCO2618* 2.45 SCO2619-2617 ClpP2, ATP dependent Clp protease proteolytic subunit 2

SCO2619 2.41 SCO2619-2617 ClpP1, ATP dependent Clp protease proteolytic subunit 1

SCO4660 2.32 SCO4659-4662 RpsJ, 30S ribosomal protein S7

SCO4701 2.36 SCO4701-4721 RpsJ, 30S ribosomal protein S10

SCO4702 2.24 SCO4701-4721 RplC, 50S ribosomal protein L3

SCO4704* 3.77 SCO4701-4721 RplW, 50S ribosomal protein L23

SCO4705 2.20 SCO4701-4721 RplB, 50S ribosomal protein L2

SCO4706* 4.09 SCO4701-4721 RpsS, 30S ribosomal protein S19

SCO4707* 2.75 SCO4701-4721 RplV, 50S ribosomal protein L22

SCO4708 2.59 SCO4701-4721 RpsC, 30S ribosomal protein S3

SCO4709 3.73 SCO4701-4721 RplP, 50S ribosomal protein L16

SCO4710* 5.30 SCO4701-4721 RpmC, 50S ribosomal protein L29

SCO4711 2.71 SCO4701-4721 RpsQ, 30S ribosomal protein S17

SCO4712 2.80 SCO4701-4721 RplN, 50S ribosomal protein L14

SCO4713* 3.42 SCO4701-4721 RplX, 50S ribosomal protein L24

SCO4714* 4.28 SCO4701-4721 RplE, 50S ribosomal protein L5

SCO4716* 3.30 SCO4701-4721 RpsH, 30S ribosomal protein S8

SCO4717 2.32 SCO4701-4721 RplF, 50S ribosomal protein L6

SCO4718 2.64 SCO4701-4721 RplR, 50S ribosomal protein L18

SCO4719* 2.34 SCO4701-4721 RpsE, 30S ribosomal protein S5

SCO4720 2.68 SCO4701-4721 RpmD, 50S ribosomal protein L30

SCO4726* 2.14 SCO4724-4731 RpmJ, 50S ribosomal protein L36

SCO4729 2.16 SCO4724-4731 RpoA, DNA-directed RNA polymerase alpha chain

SCO4730 2.36 SCO4724-4731 RplQ, 50S ribosomal protein L17

SCO4956* 2.31 SCO4956 Possible peptide methionine sulfoxide reductase

SCO5032 2.11 SCO5032-5031 AhpC, alkyl hydroperoxide reductase

SCO5370 2.11 SCO5367-5374 AtpH, ATP synthase delta chain

SCO5699 2.13 SCO5699 ProS, prolyl tRNA synthetase

SCO5999* 2.75 SCO5999 SacA, aconitase

SCO7268 2.29 SCO7268 Add, probable adenosine deaminase

Secretory proteins

SCO6108 2.41 SCO6108-6109 FusH, secreted esterase

SCO6109* 2.19 SCO6108-6109 Possible secreted hydrolase

Transcriptional regulators

SCO5778 2.33 SCO5778-5779 Two-component system transcriptional regulator

Other genes

SCO2634 2.58 SCO2634 Hypothetical protein

SCO4253* 2.36 SCO4253-4251 Hypothetical protein

**Down regulated genes**

Primary metabolism

SCO0499 -2.42 SCO0499 Possible formyltransferase

SCO1773 -2.13 SCO1773 Possible L-alanine dehydrogenase

SCO5028 -2.65 SCO5028 Possible ATP-binding protein

SCO6282 -5.99 SCO6282 Possible 3-oxoacyl-[acyl-carrier protein] reductase

SCO7399 -3.97 SCO7400-7398 Possible binding-protein- dependent transport lipoprotein

SCO7400 -3.02 SCO7400-7398 Possible ABC-transport protein, ATP-binding component

Secondary metabolism

SCO3774 -2.13 SCO3774 Possible beta-lactamase related protein

SCO6717 -2.16 SCO6717 Possible acyl-[acyl-carrier protein] desaturase. Regulated by bldA.

Transcriptional regulators

SCO1839 -3.17 SCO1839 Possible transcriptional regulator

Other genes

SCO0177 -2.77 SCO0177 Possible membrane protein

SCO0459 -2.06 SCO0459 Hypothetical protein

SCO0865 -2.16 SCO0865 Possible integral-membrane protein

SCO2780 -2.15 SCO2780 Possible lipoprotein

SCO4175 -2.13 SCO4175-4174 Hypothetical protein

SCO6040 -2.44 SCO6040 Possible lipoprotein

SCO6624 -2.51 SCO6624 Possible membrane protein

_________________________________________________________________________________

Transcriptional units potentially ppGpp regulated [10] are underlined. *Genes down regulated in the strain carrying the *SCO5785* gene in multicopy.
