# Supplementary material for: A Novel Two-Component System Involved in the Transition to Secondary Metabolism in Streptomyces coelicolor
Source: PLoS One. 2012 Feb 9;7(2):e31760. doi: 10.1371/journal.pone.0031760 (PMC3276577; doi:10.1371/journal.pone.0031760)
Supplement: Table S3 — ICPL analysis of extracellular proteins of S. coelicolor M28 labelled with C13. Extracellular proteins of S. coelicolor M145 were labelled with C12 and those of S. coelicolor M28 were labelled with C13 at 24 h of growth. (DOC) [file pone.0031760.s004.doc]

**Table S3. ICPL analysis of extracellular proteins of *S. coelicolor* M28 labelled with C13**

**________________________________________________________________________________________________________________________________________________________________________________________________________________________________________________**

Protein List: D:\Daniel Rozas ICPL_baf.d\BTDataExchange_1\ProteinList.WARPResult

WARP-LC Method: D:\Methods\WarpLCMethods\ICPL_PME5_C12C13.WarpLCMethod

BioTools Method: ICPL_NCBI_trypsin_streptomyces_C12C13

Computer Name: MSMSANALYSIS

Rel. Protein Name and Species Accession Score SC [%] # Pept. # (L/H) Avg. Avg. (SD (L/H)) # (H/L) Avg. Avg. (SD (H/L)) Abs. Inten. S/N

TRUE nucleotidase [S. coelicolor A3(2)] gi|21220497 1653,419626 50,83056478 45 27 5,49 0,46 27 0,22 0,02 245925931 2592,9

TRUE secreted protein [S. coelicolor A3(2)] gi|21224522 1541,309394 23,35640138 37 22 0,41 0,02 22 2,59 0,14 139700053 1441,3

TRUE esterase [S. coelicolor A3(2)] gi|21224437 1321,490352 38,07692308 34 18 4,98 0,3 18 0,21 0,01 184524834 1823,7

TRUE chaperonin GroEL [S. coelicolor A3(2)] gi|21222689 834,8396258 26,43253235 30 16 0,96 0,08 16 1,15 0,09 88978755 1327,8

TRUE hypothetical protein SCO6593

[S coelicolor A3(2)] gi|21224893 833,7744388 15,51874455 11 9 4,19 0,51 9 0,27 0,03 37794879 261,2

TRUE succinyl-CoA synthetase subunit beta

[S. coelicolor A3(2)] gi|21223186 749,9238177 32,74111675 21 11 0,68 0,05 11 1,55 0,11 48246424 766,7

TRUE oligopeptide-binding lipoprotein

[S. coelicolor A3(2)] gi|21223834 721,7899309 26,83333333 24 13 1,1 0,07 13 0,96 0,06 82208660 554,4

TRUE molecular chaperone DnaK

[S. coelicolor A3(2)] gi|32141213 678,9747073 20,22653722 16 12 0,48 0,03 12 2,17 0,12 41886772 578,8

TRUE secreted protein [S. coelicolor A3(2)] gi|21218846 675,9629855 30,82352941 15 11 0,15 0,04 11 10,94 2,65 44374628 471,6

TRUE dihydrolipoamide dehydrogenase

[S. coelicolor A3(2)] gi|21220654 633,4837121 28,60082305 17 11 1,15 0,08 11 0,92 0,06 89110736 613,5

TRUE secreted protein [S. coelicolor A3(2)] gi|21220594 548,3958081 11,2407211 11 8 2,19 0,11 8 0,47 0,02 42182839 306,8

TRUE secreted hydrolase [S. coelicolor A3(2)] gi|21224438 535,5038177 29,38856016 10 9 2,13 0,51 9 0,71 0,17 40797256 310,5

TRUE isocitrate dehydrogenase

[S. coelicolor A3(2)] gi|21225286 512,3576355 14,61434371 17 8 1,27 0,05 8 0,8 0,03 52249559 405,8

TRUE secreted esterase [S. coelicolor A3(2)] gi|21224523 484,9309952 11,44010767 11 7 0,3 0,05 7 4,06 0,72 24688939 466,5

TRUE tellurium resistance protein

[S. oelicolor A3(2)] gi|32141118 480,9869089 48,16753927 14 8 0,98 0,06 8 1,05 0,07 76563038 750,4

TRUE tellurium resistance protein

[S. coelicolor A3(2)] gi|21222670 479,14 59,16230366 23 10 0,74 0,06 10 1,45 0,12 103231282 812,6

TRUE secreted 5'-nucleotidase

[S. coelicolor A3(2)] gi|21222550 479,1083671 21,89542484 13 7 1,59 0,14 7 0,66 0,06 49810658 313,8

TRUE elongation factor gi|1091582 455,75 18,63979849 12 9 1,54 0,16 9 0,72 0,08 46630547 393,5

TRUE secreted protein [S. coelicolor A3(2)] gi|21224521 455,16 22,77777778 14 6 0,24 0,04 6 4,92 0,82 49827831 593,2

TRUE secreted tripeptidylaminopeptidase

[S. coelicolor A3(2)] gi|21219739 448,7109952 13,4935305 7 5 6,08 0,33 5 0,167 0,009 32680618 284,8

TRUE SLPI=protease inhibitor

[S. lividans, 66, Peptide, 107 aa] gi|257239 448,5569089 78,5046729 24 4 0,05 0,02 4 29,12 11,04 307639867 5442

TRUE aconitate hydratase [S. coelicolor A3(2)] gi|21224335 431,457904 9,402654867 10 8 1,78 0,09 8 0,57 0,03 23342858 260,3

TRUE GroEL1 [S. coelicolor] gi|406598 426,8969089 14,44444444 8 6 1,09 0,22 6 1,14 0,23 11957463 184,4

TRUE substrate binding protein

[S. coelicolor A3(2)] gi|21220385 420,3788992 15,57017544 9 7 2,96 0,39 7 0,38 0,05 31873127 226,6

TRUE hypothetical protein SCO2368

[S. coelicolor A3(2)] gi|21220836 375,6369089 31,41361257 19 8 0,89 0,03 8 1,14 0,04 72759200 741,8

TRUE secreted protein [S. coelicolor A3(2)] gi|21220348 361,1969089 22,68656716 10 5 0,196 0,009 5 5,17 0,23 47903242 279

TRUE membrane protein [S. coelicolor A3(2)] gi|21220739 346,0769089 9,647495362 5 5 9,94 2,78 5 0,14 0,04 37729571 189,1

TRUE secreted esterase [S. coelicolor A3(2)] gi|21221495 336,7969089 10,90651558 11 5 0,46 0,07 5 2,4 0,36 39293116 418,4

TRUE hypothetical protein SCO3767

[S.coelicolor A3(2)] gi|21222178 321,1307266 37,08609272 10 6 0,53 0,07 6 2,07 0,27 40844752 387

TRUE secreted protein [S. coelicolor A3(2)] gi|21220483 313,9519903 7,370184255 7 4 2,33 0,23 4 0,45 0,04 40648753 266,9

TRUE co-chaperonin GroES [S. coelicolor A3(2)] gi|21223140 312,25 74,50980392 9 6 0,39 0,04 6 2,73 0,27 38935452 334,4

TRUE secreted protein [S. coelicolor A3(2)] gi|21221339 302,1338177 13,87665198 7 5 1,44 0,06 5 0,7 0,03 5298632 188,9

TRUE BldKB [S. coelicolor] gi|1532202 296,2694679 8,637873754 4 4 1,56 0,24 4 0,7 0,11 13093334 136,6

TRUE ribosome recycling factor

[S. coelicolor A3(2)] gi|21223982 255,2884727 19,45945946 9 5 0,45 0,11 5 2,97 0,76 21096732 241,2

TRUE glycine betaine transport system permease

protein [S. coelicolor A3(2)] gi|21220116 254,8 4,47761194 4 4 4,09 0,23 4 0,25 0,01 19062626 97,7

TRUE metallopeptidase [S. coelicolor A3(2)] gi|21225030 247,85 8,102766798 4 4 3,04 0,74 4 0,41 0,1 4196424 106,9

TRUE hypothetical protein SCO3324

[S. oelicolor A3(2)] gi|21221755 242,9509952 9,307875895 5 4 0,15 0,01 4 7,16 0,71 10259622 111

TRUE cytochrome c oxidase subunit II

[S. coelicolor A3(2)] gi|21220633 241,7438177 15,04702194 13 6 3,89 0,49 6 0,28 0,04 32347892 422,4

TRUE type II citrate synthase [S. coelicolor A3(2)] gi|21221189 240,1309952 10,25641026 6 4 2,05 0,29 4 0,53 0,07 28482205 205,3

TRUE 30S ribosomal protein S1

[S. coelicolor A3(2)] gi|21220480 237,3609952 8,366533865 5 4 0,53 0,07 4 2,02 0,27 18297283 584,2

TRUE ORF3 [S. coelicolor A3(2)] gi|565055 221,69 21,4953271 3 3 0,19 0,02 3 5,54 0,57 19754800 103,4

TRUE fructose-bisphosphate aldolase

[S. coelicolor A3(2)] gi|21222064 219,06 13,11953353 4 4 0,95 0,12 4 1,11 0,14 14416890 99,6

TRUE trigger factor [S. coelicolor A3(2)] gi|21221077 215,7188992 11,96581197 5 4 1,8 0,85 4 1,05 0,49 11595509 150,9

TRUE secreted esterase [S. coelicolor A3(2)] gi|21224890 209,95 3,80952381 8 5 0,6 0,15 5 2,16 0,53 19910502 461

TRUE superoxide dismutase [S. coelicolor A3(2)] gi|21219516 203,39 20,46511628 4 3 1,48 0,12 3 0,69 0,05 21608956 176,7

TRUE superoxide dismutase [Fe-Zn] [

S. coelicolor A3(2)] gi|21221090 199,6369089 19,71830986 3 3 1,73 0,18 3 0,6 0,06 15410359 136,9

TRUE aminopeptidase N [S. coelicolor A3(2)] gi|21221100 198,967904 4,317386231 5 3 0,99 0,19 3 1,13 0,22 15970922 183,6

TRUE Clp-family ATP-binding protease

[S. coelicolor A3(2)] gi|21221802 197,7176355 4,637336504 3 2 1,33 0,3 2 0,83 0,19 9813707 109,8

TRUE hypothetical protein SCO2271

[S. coelicolor A3(2)] gi|21220740 197,32 8,385744235 3 3 4,15 0,45 3 0,25 0,03 18210814 89,6

TRUE DNA-directed RNA polymerase subunit

alpha [S. coelicolor A3(2)] gi|21223108 195,8669089 14,11764706 5 3 1,08 0,23 3 1,04 0,22 14578944 169,5

TRUE hypothetical protein SCO3967

[S. coelicolor A3(2)] gi|21222371 191,64 22,13438735 5 4 1,61 0,15 4 0,64 0,06 11567001 74

TRUE alpha-ketoglutarate decarboxylase

[S. coelicolor A3(2)] gi|21223647 184,4586307 4,402515723 5 3 2,16 0,32 3 0,49 0,07 12627413 93,6

TRUE secreted protein [S. coelicolor A3(2)] gi|21222858 183,11 8,333333333 4 3 4,14 0,9 3 0,28 0,06 2590118 115,9

TRUE Rieske iron-sulfur protein

[S. coelicolor A3(2)] gi|21220626 179,74 7,932011331 11 3 1,14 0,44 3 1,27 0,49 25493982 431,7

TRUE triosephosphate isomerase

[S. coelicolor A3(2)] gi|21220430 178,9169089 9,302325581 4 3 0,68 0,08 3 1,54 0,18 13798053 122,5

TRUE 50S ribosomal protein L1

[S. coelicolor A3(2)] gi|21223031 175,6238177 10,37344398 2 2 1,99 0,28 2 0,52 0,07 2863273 27,4

TRUE hypothetical protein SCO4584

[S. oelicolor A3(2)] gi|21222967 174,6 6,904761905 4 3 2,41 0,44 3 0,45 0,08 7457544 128,4

TRUE lipoprotein [S. coelicolor A3(2)] gi|21223259 171,457904 14,94252874 4 3 2,55 0,67 3 0,47 0,13 7655301 84,3

TRUE transcriptional regulator

[S. coelicolor A3(2)] gi|21221840 169,8409952 7,90513834 6 2 0,42 0,02 2 2,37 0,13 31261433 339,4

TRUE secreted protein [S. coelicolor A3(2)] gi|21223053 169,2569089 7,300509338 6 4 1,62 0,21 4 0,66 0,09 21643123 181,4

TRUE secreted protein [S. coelicolor A3(2)] gi|21224891 168,74 16,07142857 6 4 0,99 0,04 4 1,01 0,04 17893998 190,8

TRUE carboxyl transferase [S. coelicolor A3(2)] gi|21223890 166,93 6,451612903 4 3 5,55 0,27 3 0,181 0,009 5643630 161,6

TRUE phosphoglycerate kinase

[S. coelicolor A3(2)] gi|21220431 164,07 7,444168734 3 2 0,49 0,04 2 2,08 0,15 4090107 87

TRUE substrate binding protein

[S. coelicolor A3(2)] gi|21224757 162,88 5,904059041 3 3 4,37 0,91 3 0,26 0,05 6061620 63

TRUE regulatory protein [S. coelicolor A3(2)] gi|21225529 156,62 6,374501992 3 3 5,88 2,55 3 0,27 0,12 6500887 75,4

TRUE malate dehydrogenase [S. coelicolor A3(2)] gi|21223204 153,19 9,118541033 3 3 0,7 0,07 3 1,48 0,14 5578430 98,8

TRUE 30S ribosomal protein S3

[S. coelicolor A3(2)] gi|21223088 153,0469089 16,24548736 3 3 2,47 0,23 3 0,42 0,04 4174504 58,4

TRUE glutamine synthetase I [S. coelicolor A3(2)] gi|21220671 149,57 7,249466951 2 2 2,03 0,06 2 0,49 0,01 3056727 147,2

TRUE F0F1 ATP synthase subunit beta

[S. coelicolor A3(2)] gi|21223733 147,8069089 4,811715481 2 2 1,57 0,22 2 0,66 0,09 8707943 49,9

TRUE NLP/P60 family protein [S. coelicolor A3(2)] gi|21222944 144,83 11,19133574 9 3 8,58 0,39 3 0,117 0,005 30438074 1523,8

TRUE 50S ribosomal protein L7/L12

[S. coelicolor A3(2)] gi|21223035 141,4069089 20,47244094 5 2 0,85 0,08 2 1,19 0,11 26698390 172,6

TRUE F0F1 ATP synthase subunit alpha

[S. coelicolor A3(2)] gi|21223731 139,8069089 7,75047259 4 3 1,45 0,22 3 0,74 0,11 5803013 83,6

TRUE aldehyde dehydrogenase

[S. coelicolor A3(2)] gi|21223286 138,2707266 9,556313993 2 2 1,88 0,68 2 0,67 0,24 5443588 38,6

TRUE secreted protein [S. coelicolor A3(2)] gi|21220861 137,07 10,07194245 3 3 2,97 0,52 3 0,37 0,07 4366938 67,1

TRUE gamma-glutamyltranspeptidase (putative

secreted protein) [S. coelicolor A3(2)] gi|21224715 136,687904 3,648424544 2 2 1,93 0,2 2 0,53 0,06 5076351 33,4

TRUE lipoprotein [S. coelicolor A3(2)] gi|21222720 134,87 19,48051948 5 3 1,38 0,39 3 0,89 0,25 7164441 192,6

TRUE succinyl-CoA synthetase subunit alpha

[S. coelicolor A3(2)] gi|21223187 134,27 9,523809524 3 3 0,76 0,11 3 1,41 0,21 2299277 41,3

TRUE aldehyde dehydrogenase

[S. coelicolor A3(2)] gi|21223287 131,9169089 3,958333333 2 2 1,83 0,02 2 0,546 0,006 2742823 39,5

TRUE phosphomannomutase

[S. coelicolor A3(2)] gi|21223290 129,95 6,921675774 3 3 0,49 0,04 3 2,1 0,17 2004449 35,6

TRUE phosphocarrier protein HPr

[S. coelicolor A3(2)] gi|21224185 128,67 27,95698925 3 2 0,42 0,16 2 3,08 1,18 18456974 54,9

TRUE peptidyl-prolyl cis-trans isomerase

[S. coelicolor A3(2)] gi|21225776 126,16 18,18181818 3 2 0,7676 0,0003 2 1,3028 0,0005 15911496 93,2

TRUE oxidoreductase (secreted protein)

[S. coelicolor A3(2)] gi|21225017 125,6 6,034482759 2 2 2,57 0,17 2 0,39 0,03 3419769 95,9

TRUE elongation factor G [S. coelicolor A3(2)] gi|21223042 123,37 2,824858757 2 2 0,84 0,24 2 1,4 0,4 2048588 53,4

TRUE hypothetical protein SCO2999

[S. coelicolor A3(2)] gi|32141188 119,7748129 0,907441016 2 2 2,26 0,72 2 0,53 0,17 1799955 71,6

TRUE ketol-acid reductoisomerase

[S. coelicolor A3(2)] gi|21223870 119,6 7,831325301 2 2 0,685 0,007 2 1,46 0,02 3668295 25,1

TRUE aspartate aminotransferase

[S. coelicolor A3(2)] gi|21223027 115,84 8,578431373 3 2 0,87 0,06 2 1,16 0,08 9645711 42,2

TRUE RecName: Full=30S ribosomal protein S2 gi|6226896 112,3469089 6,451612903 2 2 3,98 0,1 2 0,252 0,006 780553 24,8

TRUE secreted protein [S. coelicolor A3(2)] gi|21222370 111,61 14,35185185 2 2 1,26 0,02 2 0,8 0,01 6422491 25,7

TRUE spore-associated protein precursor

[S. coelicolor A3(2)] gi|21218951 110,33 16,88311688 2 2 0,41 0,12 2 2,9 0,88 3750017 30,7

TRUE carboxypeptidase [S. coelicolor A3(2)] gi|21224458 108,2 3,32594235 2 1 5,26 0 1 0,19 0 2118255 36

TRUE hypothetical protein SCO3899

[S. coelicolor A3(2)] gi|21222306 105,4309952 6,666666667 3 2 1,45 0,14 2 0,7 0,07 6439976 46,4

TRUE aminopeptidase [S. coelicolor A3(2)] gi|21221092 101,54 2,761104442 2 2 0,92 0,04 2 1,09 0,04 1457242 52,3

TRUE secreted protein [S. coelicolor A3(2)] gi|21223402 101,067904 12,60504202 3 2 1,02 0,07 2 0,99 0,07 5610316 63,5

TRUE serine hydroxymethyltransferase

[S. coelicolor A3(2)] gi|21223213 95,08 4,365904366 2 2 0,73 0,1 2 1,42 0,2 2934893 43,6

TRUE glutamate binding protein

[S. coelicolor A3(2)] gi|21224122 93,17099517 7,194244604 2 2 1,05 0,02 2 0,96 0,02 8270703 26,2

TRUE branched chain amino acid binding protein

[S. coelicolor A3(2)] gi|21220490 92,84 2,870813397 2 1 0,98 0 1 1,02 0 14501712 73,2

TRUE hydrolytic protein [S. coelicolor A3(2)] gi|21222651 92,35 3,966597077 2 2 2,28 0,15 2 0,44 0,03 7003307 46

TRUE zinc protease [S. coelicolor A3(2)] gi|21224181 90,95 2,444444444 2 1 0,83 0 1 1,21 0 2195481 24,6

TRUE secreted protease [S. coelicolor A3(2)] gi|21221367 89,92690887 1,408450704 3 2 1,61 0,06 2 0,62 0,03 10659063 87,1

TRUE superoxide dismutase [S. coelicolor A3(2)] gi|21223621 88,62 12,21374046 2 2 0,56 0,23 2 2,38 0,97 8927390 163

TRUE hypothetical protein SCO1074

[S. coelicolor A3(2)] gi|21219589 87,27 4,4345898 2 2 2 1,5 1,54 2 2,05 2,09 9690334 30,7

TRUE hypothetical protein SCO4253

[S. coelicolor A3(2)] gi|21222648 86,38 2,434456929 2 2 2,86 1,01 2 0,44 0,15 1595763 59,9

TRUE nucleoside diphosphate kinase

[S. coelicolor A3(2)] gi|2125898 85,67 17,51824818 2 2 2,68 0,84 2 0,44 0,14 10367040 32,3

TRUE hypothetical protein SCO4175

[S. coelicolor A3(2)] gi|21222572 83,31690887 20 2 2 0,3 0,16 2 5,23 2,8 966140 45,7

TRUE electron transfer flavoprotein, alpha

subunit [S. coelicolor A3(2)] gi|21219596 75,64 9,375 2 2 3,88 1,2 2 0,31 0,1 1910647 34,1

TRUE acyl carrier protein [S. coelicolor A3(2)] gi|21220856 72,66 17,07317073 3 2 0,683 0,004 2 1,464 0,008 1772240 109,4

TRUE purine nucleoside phosphorylase

[S. coelicolor A3(2)] gi|21223291 70,29 6,934306569 4 2 1,44 0,08 2 0,7 0,04 3458571 86

Protein List: D:\Daniel Rozas ICPL_baf.d\BTDataExchange_1\ProteinList.WARPResult

WARP-LC Method: D:\Methods\WarpLCMethods\ICPL_PME5_C12C13.WarpLCMethod

BioTools Method: ICPL_NCBI_trypsin_streptomyces_C12C13

Computer Name: MSMSANALYSIS

Proteins: Peptides:

Rel. Protein Name and

Species Accession Score SC [%] # Pept. # (L/H) Avg. Avg. (SD (L/H))# (H/L) Avg. Avg. (SD (H/L)) Abs. Inten. S/N Rel. Cmpd MH+ (calc) [Da] m/z (calc) Δ m [Da] z RT [min] Ions Sc. Rank S/N L/H H/L Miss Sequence Variable Modif.

TRUE nucleotidase

[S. coelicolor A3(2)] gi|21220497 1653,419626 50,83056478 45 27 5,49 0,46 27 0,22 0,02 245925931 2592,9 TRUE 1849 838,409376 838,409376 0,015824 1 188,8983552 34,4 1 34,6 5,957809545 0,167846923 0 GVLDPK ICPL (K); ICPL

(N-term)

gi|21220497 TRUE 4806 976,557416 488,782346 0,055708 2 429,7783079 51,89 1 107,1 7,602570782 0,131534454 0 LLTGAQLR ICPL (N-term)

gi|21220497 TRUE 221 1008,474485 504,7408805 0,097439 2 41,50759093 48,18 1 81,8 11,21695008 0,089150793 0 VVNEETGR ICPL (N-term)

gi|21220497 TRUE 1791 1026,489088 1026,489088 0,038512 1 184,0650885 29,8 1 21,8 3,609320279 0,277060478 0 VQEDVVK ICPL (K); ICPL (N-term)

gi|21220497 TRUE 1787 1026,489088 513,748182 0,050436 2 183,8482384 43,48 1 65,9 3,609320279 0,277060478 0 VQEDVVK ICPL (K); ICPL (N-term)

gi|21220497 TRUE 4482 1029,605115 515,3061955 0,134009 2 403,608741 46,21 1 40,5 0 ISTLVNQVR

gi|21220497 TRUE 3343 1094,505399 547,7563375 0,108125 2 306,4339136 46,44 1 27 4,179214981 0,239279387 0 WHVESVK ICPL (K); ICPL (N-term)

gi|21220497 TRUE 4888 1134,626569 567,8169225 0,101755 2 435,6304162 65,23 1 109,5 7,269921866 0,13755306 0 ISTLVNQVR ICPL (N-term)

gi|21220497 TRUE 1774 1164,53198 582,769628 0,134544 2 182,7444885 53,88 1 72,2 6,753666032 0,14806773 0 LLADEHEK ICPL (K); ICPL (N-term)

gi|21220497 TRUE 1780 1253,579694 627,293485 0,10123 2 183,3944554 74,23 1 47,2 5,787246774 0,172793738 0 TAAGAPVDVDK ICPL (K); ICPL (N-term)

gi|21220497 TRUE 1599 1258,663755 629,8355155 0,168169 2 170,6083386 33,57 1 11,5 0 TSEIPAGDVTIR

gi|21220497 TRUE 4053 1320,723676 660,865476 0,066648 2 350,7958554 71,62 1 58,8 4,815044572 0,207682397 0 DAPIIDLITK ICPL:13C(6) (K); ICPL:13C(6) (N-term)

gi|21220497 TRUE 1910 1354,627332 677,817304 0,137392 2 193,1196053 64,03 1 47,9 4,625068469 0,216213015 0 ELWSESTEIR ICPL (N-term)

gi|21220497 TRUE 3867 1483,72157 742,364423 -0,002646 2 339,1075802 82,68 1 513 7,339693747 0,136245467 0 LAFPGLEEQAAK ICPL (K); ICPL (N-term)

gi|21220497 TRUE 3909 1483,72157 742,364423 0,133554 2 342,0302138 45,6 1 15,8 1,98326742 0,504218438 0 LAFPGLEEQAAK ICPL (K); ICPL (N-term)

gi|21220497 TRUE 3226 1493,692029 747,3496525 0,072095 2 299,4416138 78,84 1 19,9 3,358487426 0,297753087 0 ANGGGAFPHVASAK ICPL (K); ICPL (N-term)

gi|21220497 TRUE 3870 1495,761838 748,384557 -0,005514 2 339,2205717 66,25 1 119,3 7,339693747 0,136245467 0 LAFPGLEEQAAK ICPL:13C(6) (K); ICPL:13C(6) (N-term)

gi|21220497 TRUE 2109 1625,799192 813,403234 -0,094068 2 207,8190805 53,04 1 10,9 2,300443199 0,434698844 0 TVVLSEPLCFAER ICPL (N-term)

gi|21220497 TRUE 5049 1625,799192 813,403234 0,017532 2 447,507941 62,54 1 22,5 2,262822549 0,441925948 0 TVVLSEPLCFAER ICPL (N-term)

gi|21220497 TRUE 3699 1625,799192 813,403234 0,030532 2 327,6795552 71,35 1 140 7,085498347 0,141133333 0 TVVLSEPLCFAER ICPL (N-term)

gi|21220497 TRUE 5368 1704,863157 852,9352165 -0,001433 2 471,1177746 77,42 1 59,6 8,556294076 0,116873028 1 YKDAPIIDLITK 2 ICPL (K); ICPL (N-term)

gi|21220497 TRUE 1096 1704,863157 852,9352165 0,027767 2 110,8785744 77,51 1 24,8 4,823686537 0,20731032 1 YKDAPIIDLITK 2 ICPL (K); ICPL (N-term)

gi|21220497 TRUE 1098 1704,863157 568,9592363 0,027791 3 110,9095579 49,4 1 8,9 4,823686537 0,20731032 1 YKDAPIIDLITK 2 ICPL (K); ICPL (N-term)

gi|21220497 TRUE 5369 1704,863157 568,9592363 0,170591 3 470,8023495 59,87 1 74,4 8,556294076 0,116873028 1 YKDAPIIDLITK 2 ICPL (K); ICPL (N-term)

gi|21220497 TRUE 1661 1713,74629 857,376783 -0,026166 2 174,7259472 120,92 1 69,9 6,093738539 0,164102873 0 DAEYTDAAGNAQGLGR ICPL (N-term)

gi|21220497 TRUE 6681 1876,995588 939,001432 -0,098864 2 594,1828304 115,04 1 72,2 6,828863962 0,146437241 0 VAVLGLTNPGIAIWDK ICPL (K); ICPL

(N-term)

gi|21220497 TRUE 5343 1910,917051 955,9621635 -0,071127 2 468,9149159 85,75 1 39,5 4,645947556 0,215241345 0 DLSSLYVYDNTLVAK ICPL (K); ICPL

(N-term)

gi|21220497 TRUE 5306 1910,917051 955,9621635 -0,044327 2 466,5782914 71,53 1 52,5 7,247592809 0,137976846 0 DLSSLYVYDNTLVAK ICPL (K); ICPL

(N-term)

gi|21220497 TRUE 1016 1910,917051 955,9621635 -0,036127 2 106,2554325 88,68 1 14,5 5,311156337 0,188282916 0 DLSSLYVYDNTLVAK ICPL (K); ICPL

(N-term)

gi|21220497 TRUE 5299 1910,917051 637,6438677 0,007297 3 466,4822247 28,1 1 26,6 7,247592809 0,137976846 0 DLSSLYVYDNTLVAK ICPL (K); ICPL

(N-term)

gi|21220497 TRUE 5307 1922,957319 961,9822975 -0,036795 2 466,5356743 71,19 1 11,5 7,247592809 0,137976846 0 DLSSLYVYDNTLVAK ICPL:13C(6) (K); ICPL:13C(6) (N-term)

gi|21220497 TRUE 3698 2390,086988 797,36718 0,10266 3 327,8492298 61,75 1 55,8 5,939986271 0,168350557 1 RFEEQCDFPLLGANAVDAK ICPL (K); ICPL (N-term)

gi|21220497 TRUE 6656 2426,256193 809,4235817 0,020755 3 592,7118886 58,35 1 33,1 3,291293691 0,303831895 0 VVAYVNQVVGTATETLTTVEAR ICPL

(N-term)

gi|21220497 TRUE 1069 2627,266401 876,4269843 -0,107953 3 109,4479749 102,16 1 30,2 3,156824719 0,316774002 0 NTLLVDAGDTIQGTPLTYYYAK ICPL (K); ICPL (N-term)

gi|21220497 TRUE 931 2644,27902 882,0978573 -0,089272 3 100,3464992 63,39 1 26,2 5,272054856 0,189679362 0 NLTYGGAPLDDAQQFVLAVNNYR ICPL

(N-term)

TRUE secreted protein

[S. coelicolor A3(2)] gi|21224522 1541,309394 23,35640138 37 22 0,41 0,02 22 2,59 0,14 139700053 1441,3 TRUE 3122 882,525129 441,7662025 0,088995 2 292,7899301 31,05 1 74,1 0,298103372 3,354541054 1 RVDILR ICPL:13C(6) (N-term)

gi|21224522 TRUE 1769 1003,492216 502,249746 0,062308 2 182,2742971 29,66 1 23,6 0,278888958 3,585656487 0 QIYTEK ICPL:13C(6) (K); ICPL:13C(6) (N-term)

gi|21224522 TRUE 3439 1035,529676 518,268476 0,031448 2 312,4100384 35,31 1 23,7 0,536200615 1,864973615 0 ILGDNPGK ICPL:13C(6) (K); ICPL:13C(6) (N-term)

gi|21224522 TRUE 1785 1050,521421 525,7643485 0,123503 2 183,5731552 49,29 1 38,6 0,372298732 2,686015056 0 TAELQDLR ICPL (N-term)

gi|21224522 TRUE 1784 1056,541562 528,774419 0,082762 2 183,5405973 37,85 1 115,7 0,372298732 2,686015056 0 TAELQDLR ICPL:13C(6) (N-term)

gi|21224522 TRUE 3450 1122,588514 561,797895 0,09401 2 312,9849386 24,57 1 59,2 0,325793731 3,069426768 0 LPELPDTAR ICPL:13C(6) (N-term) gi|21224522 TRUE 4745 1196,609205 598,8082405 0,165519 2 425,2984157 49,59 1 23,3 0,479683028 2,084709989 0 TVLGAMALSGR ICPL (N-term); Oxidation (M)

gi|21224522 TRUE 4739 1202,629346 601,818311 0,102978 2 424,8475906 99,59 1 33,5 0,479683028 2,084709989 0 TVLGAMALSGR ICPL:13C(6) (N- term); Oxidation (M)

gi|21224522 TRUE 4797 1412,691678 706,849477 0,142046 2 429,2272407 108,02 1 53,7 0,348355034 2,870634564 0 FNTLSIQNSAGR ICPL (N-term)

gi|21224522 TRUE 4793 1418,71182 709,859548 0,070904 2 429,0640242 82,56 1 90,1 0,348355034 2,870634564 0 FNTLSIQNSAGR ICPL:13C(6) (N- term) gi|21224522 TRUE 1654 1545,729169 773,3682225 0,075555 2 174,2662138 67,25 1 30,6 0,346542817 2,885646309 0 LAHDTEQSLAEAR ICPL (N-term)

gi|21224522 TRUE 4864 1617,807493 809,4073845 -0,005769 2 433,7679239 93,3 1 43,7 0,66316203 1,507927106 0 SAFAATAAAQAAATAGR ICPL:13C(6) (N-term)

gi|21224522 TRUE 6140 1617,807493 809,4073845 0,054831 2 555,3424054 68,75 1 27,3 0,351027087 2,848783003 0 SAFAATAAAQAAATAGR ICPL:13C(6) (N-term)

gi|21224522 TRUE 5293 1654,807111 827,9071935 0,015213 2 465,7223911 98,18 1 24,9 0,439075064 2,277514897 0 GVSQILEDASQGLK ICPL (K); ICPL (N-term)

gi|21224522 TRUE 1009 1666,847379 833,9273275 -0,055055 2 105,7877664 67,32 1 6,8 0,346723522 2,884142372 0 GVSQILEDASQGLK ICPL:13C(6) (K); ICPL:13C(6) (N-term)

gi|21224522 TRUE 1012 1666,847379 833,9273275 -0,000855 2 105,7421083 76,79 1 1,2 0,346723522 2,884142372 0 GVSQILEDASQGLK ICPL:13C(6) (K); ICPL:13C(6) (N-term)

gi|21224522

TRUE 5291 1666,847379 833,9273275 0,016545 2 465,5780162 91,04 1 52,2 0,439075064 2,277514897 0 GVSQILEDASQGLK ICPL:13C(6) (K); ICPL:13C(6) (N-term)

gi|21224522 TRUE 4923 1733,860532 867,433904 0,029392 2 438,1730413 100,61 1 39,4 0,34298882 2,915546924 0 NFLTTGIHEAAALDR ICPL (N-term)

gi|21224522 TRUE 642 1739,880674 870,443975 0,00585 2 79,05203253 67,06 1 13,7 0,237648363 4,207897696 0 NFLTTGIHEAAALDR ICPL:13C(6) (N-term)

gi|21224522 TRUE 4919 1739,880674 870,443975 0,09585 2 438,0285831 90,79 1 86 0,34298882 2,915546924 0 NFLTTGIHEAAALDR ICPL:13C(6) (N-term)

gi|21224522 TRUE 4921 1739,880674 580,631742 0,095874 3 438,0119079 60,36 1 81,8 0,34298882 2,915546924 0 NFLTTGIHEAAALDR ICPL:13C(6) (N-term)

gi|21224522 TRUE 3263 1806,834821 903,9210485 0,017703 2 301,2538218 132,11 1 21,2 0,536747006 1,863075135 0 AEAAYASGDTASALANGR ICPL:13C(6) (N-term)

gi|21224522 TRUE 5397 1910,009687 955,5084815 -0,112763 2 472,4346658 84,46 1 45,8 0,429427664 2,3286809 0 VEVFTILANASPEVAK ICPL:13C(6) (K); ICPL:13C(6) (N-term)

gi|21224522 TRUE 1126 1910,009687 955,5084815 -0,072163 2 112,5089744 60,06 1 9,6 0,564824521 1,770461377 0 VEVFTILANASPEVAK ICPL:13C(6) (K); ICPL:13C(6) (N-term)

gi|21224522 TRUE 5364 2029,986524 1015,4969 0,1014 2 470,4857826 115,66 1 26,8 0,388473276 2,574179644 0 ALAGSATDIDGFLAEGLAK ICPL (K); ICPL (N-term)

gi|21224522 TRUE 5362 2029,986524 677,333692 0,140424 3 470,4111079 76,2 1 14,2 0,388473276 2,574179644 0 ALAGSATDIDGFLAEGLAK ICPL (K); ICPL (N-term)

gi|21224522 TRUE 1090 2042,026792 681,3471147 0,086356 3 110,4650912 95,9 1 25,2 0,34008869 2,940409457 0 ALAGSATDIDGFLAEGLAK ICPL:13C(6) (K); ICPL:13C(6) (N-term)

gi|21224522 TRUE 5359 2042,026792 681,3471147 0,463456 3 470,2864498 92,99 1 78,9 0,388473276 2,574179644 0 ALAGSATDIDGFLAEGLAK ICPL:13C(6) (K); ICPL:13C(6) (N-term)

gi|21224522 TRUE 5360 2042,026792 1021,517034 0,463532 2 470,2999079 139,59 1 68,5 0,388473276 2,574179644 0 ALAGSATDIDGFLAEGLAK ICPL:13C(6) (K); ICPL:13C(6) (N-term)

gi|21224522 TRUE 376 2055,894559 685,9697037 -0,028811 3 55,10109119 65,94 1 40,2 0,493564179 2,026078964 0 ESGVDPADNADNDVVNSAR ICPL:13C(6) (N-term)

gi|21224522 TRUE 377 2055,894559 1028,450918 0,004365 2 55,05422453 82,38 1 23,5 0,493564179 2,026078964 0 ESGVDPADNADNDVVNSAR ICPL:13C(6) (N-term)

gi|21224522 TRUE 6493 2719,431241 907,1485977 0,017707 3 581,8397467 120,66 1 23 0,529218625 1,889578242 1 KAAQALAAAQTATQAAAAAGISAAATAR ICPL:13C(6) (K); ICPL:13C(6) (N-term)

gi|21224522 TRUE 4985 2852,405744 951,473432 -0,088896 3 442,9093661 105,44 1 26,8 0,39713737 2,518020404 0 DFITTVQHSAAQLDYDNAAHIAAIR ICPL:13C(6) (N-term)

TRUE esterase

[S. coelicolor A3(2)] gi|21224437 1321,490352 38,07692308 34 18 4,98 0,3 18 0,21 0,01 184524834 1823,7 TRUE 3366 791,440992 396,224134 0,086932 2 307,798472 31,87 1 43,6 4,200605728 0,238060905 0 ADLLVR ICPL (N-term)

gi|21224437 TRUE 4857 890,509412 445,758344 0,028912 2 433,2801831 41,07 1 6,5 5,336280473 0,187396447 0 ADLVVLR ICPL (N-term)

gi|21224437 TRUE 3159 944,447217 472,7272465 0,032707 2 295,2631802 47,44 1 21,8 2,621488153 0,38146272 0 STATLGGK ICPL (K); ICPL (N-term)

gi|21224437 TRUE 1462 948,453367 474,7303215 0,089157 2 161,2637717 58,59 1 35 3,775994059 0,264830925 0 GDTGGPALR ICPL (N-term)

gi|21224437 TRUE 4692 1082,656827 541,8320515 0,118297 2 420,763341 66,34 1 38,2 0 TLGIVEVVPR

gi|21224437 TRUE 3418 1084,505796 542,756536 0,056928 2 310,9912304 45,74 1 35 4,654525913 0,214844652 0 FIDGSDLGR ICPL (N-term)

gi|21224437 TRUE 1407 1087,527936 544,267606 0,143988 2 156,458464 81,49 1 23,7 3,547016358 0,28192709 0 SNGDVVVHR ICPL (N-term)

gi|21224437 TRUE 4961 1187,678281 594,3427785 0,061243 2 440,955941 83,01 1 381,4 5,512657164 0,181400724 0 TLGIVEVVPR ICPL (N-term)

gi|21224437 TRUE 674 1187,678281 594,3427785 0,123043 2 81,55930826 65,88 1 43,3 7,249341578 0,137943562 0 TLGIVEVVPR ICPL (N-term)

gi|21224437 TRUE 3230 1215,611646 405,8753993 0,036602 3 299,5558554 65,84 1 3,7 5,839053898 0,171260622 0 LHIGEGDTLR ICPL (N-term)

gi|21224437 TRUE 3224 1215,611646 608,309461 0,153678 2 299,1653973 51,37 1 116,5 5,839053898 0,171260622 0 LHIGEGDTLR ICPL (N-term)

gi|21224437 TRUE 549 1415,752897 708,3800865 0,019027 2 70,62126612 37,95 1 13,3 3,148580255 0,317603465 0 LAEPVTTVEPVR ICPL (N-term)

gi|21224437 TRUE 3396 1415,752897 708,3800865 0,092227 2 309,6567637 68,42 1 68,6 5,935680477 0,16847268 0 LAEPVTTVEPVR ICPL (N-term)

gi|21224437 TRUE 3689 1422,664823 711,8360495 0,161101 2 327,551897 100,43 1 144,1 5,183932362 0,192903751 0 ADGLADWVTDVR ICPL (N-term)

gi|21224437 TRUE 1605 1487,723722 744,365499 0,084402 2 170,8822635 112,17 1 155,8 5,620512328 0,177919724 0 GTGGEVELAAVHSR ICPL (N-term)

gi|21224437 TRUE 1606 1487,723722 496,5794247 0,084526 3 170,8976218 81,36 1 51,5 5,620512328 0,177919724 0 GTGGEVELAAVHSR ICPL (N-term)

gi|21224437 TRUE 3628 1508,680463 754,8438695 0,117661 2 323,6170469 64,61 1 42,5 5,180639456 0,193026365 1 LYFADVDGDRK ICPL (K); ICPL (N-term)

gi|21224437 TRUE 3140 1733,791166 867,399221 0,048758 2 293,7991221 79,77 1 31,1 5,02144712 0,199145779 0 ADMIVHTGDGNVEVR ICPL (N-term); Oxidation (M)

gi|21224437 TRUE 3141 1733,791166 578,601906 0,152382 3 293,9115968 84,46 1 27,1 5,02144712 0,199145779 0 ADMIVHTGDGNVEVR ICPL (N-term); Oxidation (M)

gi|21224437 TRUE 5267 1904,986463 952,9968695 -0,102539 2 463,6347495 151,59 1 133,2 4,682444406 0,213563667 0 LAADAPVAAETLLGAGFGR ICPL (N-term)

gi|21224437 TRUE 978 1904,986463 952,9968695 0,005861 2 103,5873243 158,21 1 60,6 5,108688574 0,195744952 0 LAADAPVAAETLLGAGFGR ICPL (N-term) gi|21224437 TRUE 3955 2196,935667 1098,971472 -0,117143 2 344,6725552 132,36 1 32,6 6,842103131 0,146153892 0 FVDGSDLGSLEFGDATGDGK ICPL (K); ICPL (N-term)

gi|21224437 TRUE 3962 2208,975934 1104,991605 -0,15941 2 345,2559136 99,47 1 11 6,842103131 0,146153892 0 FVDGSDLGSLEFGDATGDGK ICPL:13C(6) (K); ICPL:13C(6) (N-term)

TRUE chaperonin GroEL

[S. coelicolor A3(2)] gi|21222689 834,8396258 26,43253235 30 16 0,96 0,08 16 1,15 0,09 88978755 1327,8 TRUE 3647 939,493421 470,2503485 0,062103 2 324,9126634 40,7 1 36,9 1,177397657 0,84933072 0 IGAELVK ICPL (K); ICPL (N-term)

gi|21222689 TRUE 3642 951,533689 476,2704825 0,046635 2 324,7245968 44,27 1 37,2 1,177397657 0,84933072 0 IGAELVK ICPL:13C(6) (K); ICPL:13C(6) (N-term)

gi|21222689 TRUE 5063 1049,618101 525,3126885 0,049023 2 448,2973826 54,95 1 34,7 0,741937988 1,347821538 0 LAGGVAVIK ICPL:13C(6) (K); ICPL:13C(6) (N-term)

gi|21222689 TRUE 3851 1064,577466 532,792371 0,058258 2 337,7432469 52,85 1 96,3 1,222824789 0,817778646 0 LALEAPLK ICPL (K); ICPL (N-term)

gi|21222689 TRUE 3854 1064,577466 1064,577466 0,086534 1 337,9057301 45,24 1 31,2 1,222824789 0,817778646 0 LALEAPLK ICPL (K); ICPL (N-term)

gi|21222689 TRUE 3849 1076,617734 538,812505 0,08399 2 337,5277386 55,65 1 81 1,222824789 0,817778646 0 LALEAPLK ICPL:13C(6) (K); ICPL:13C(6) (N-term)

gi|21222689 TRUE 4072 1150,614255 1150,614255 0,023345 1 351,6028138 42,69 1 24,4 0,86515419 1,155863327 0 DLLPLLEK ICPL (K); ICPL (N-term)

gi|21222689 TRUE 4068 1150,614255 575,8107655 0,077069 2 351,4718554 71,9 1 112,6 0,86515419 1,155863327 0 DLLPLLEK ICPL (K); ICPL (N-term)

gi|21222689 TRUE 2521 1162,654523 581,8308995 -0,071399 2 231,7061221 52,27 1 15 1,005966463 0,994068925 0 DLLPLLEK ICPL:13C(6) (K); ICPL:13C(6) (N-term)

gi|21222689 TRUE 4071 1162,654523 1162,654523 0,029577 1 351,5909802 46,67 1 48,1 0,86515419 1,155863327 0 DLLPLLEK ICPL:13C(6) (K); ICPL:13C(6) (N-term)

gi|21222689 TRUE 4064 1162,654523 581,8308995 0,061601 2 351,3169722 55,29 1 176,9 0,86515419 1,155863327 0 DLLPLLEK ICPL:13C(6) (K); ICPL:13C(6) (N-term)

gi|21222689 TRUE 1986 1168,563276 584,785276 0,156648 2 198,6734304 74,19 1 55,1 1,402564439 0,712979719 0 IIAFDEEAR ICPL (N-term)

gi|21222689 TRUE 1879 1210,614118 605,810697 0,098606 2 190,7947803 70,62 1 45,8 0,860740445 1,161790416 0 AGAATEVELK ICPL:13C(6) (K); ICPL:13C(6) (N-term)

gi|21222689 TRUE 805 1477,764493 739,3858845 0,104831 2 91,65299092 51,52 1 24 0,497253394 2,011047109 0 LENATLDLLGSAR ICPL (N-term)

gi|21222689 TRUE 5099 1477,764493 739,3858845 0,136831 2 451,5156994 57,97 1 99,3 0,63651113 1,571064436 0 LENATLDLLGSAR ICPL (N-term)

gi|21222689 TRUE 804 1483,784635 742,3959555 0,096889 2 91,5469744 77,98 1 27,5 0,497253394 2,011047109 0 LENATLDLLGSAR ICPL:13C(6) (N- term)

gi|21222689 TRUE 2075 1486,677488 743,842382 0,019036 2 205,8351552 58,18 1 23,4 1,527136601 0,654820269 0 EIELEDPYEK ICPL:13C(6) (K); ICPL:13C(6) (N-term)

gi|21222689 TRUE 5344 1609,822004 805,41464 0,06352 2 469,1782413 76,68 1 37,1 1,030575545 0,970331583 0 AVEAVSAALLEQAK ICPL (K); ICPL (N-term)

gi|21222689 TRUE 5312 1609,822004 805,41464 0,09992 2 466,8866578 76,84 1 53,9 0,975788807 1,024811919 0 AVEAVSAALLEQAK ICPL (K); ICPL (N-term)

gi|21222689 TRUE 5311 1621,862272 811,434774 0,100852 2 466,8723325 72,33 1 68,6 0,975788807 1,024811919 0 AVEAVSAALLEQAK ICPL:13C(6) (K); ICPL:13C(6) (N-term)

gi|21222689 TRUE 2052 1698,796934 849,902105 -0,04161 2 203,8667056 113,68 1 44 1,167449327 0,856568227 0 LELTGDEATGANAVK ICPL (K); ICPL (N-term)

gi|21222689 TRUE 2081 1698,796934 849,902105 0,56459 2 205,7706474 56,16 1 11,3 0,691618335 1,445884166 0 LELTGDEATGANAVK ICPL (K); IC PL (N-term)

gi|21222689 TRUE 2051 1710,837202 855,922239 -0,046278 2 203,8127557 111,84 1 11,8 1,167449327 0,856568227 0 LELTGDEATGANAVK ICPL:13C(6) (K); ICPL:13C(6) (N-term)

gi|21222689 TRUE 1702 1923,867902 962,437589 -0,158778 2 177,7519802 104,26 1 12,8 0,591849665 1,689618258 0 DETTIVDGAGSADQVQGR ICPL (N- term)

gi|21222689 TRUE 5257 2490,214734 830,7430953 0,158714 3 462,948733 50,41 1 18,7 0,923341971 1,08302236 1 KTDDVAGDGTTTATVLAQALVK 2 ICPL (K); ICPL (N-term)

TRUE hypothetical protein SCO6593

[S. coelicolor A3(2)] gi|21224893 833,7744388 15,51874455 11 9 4,19 0,51 9 0,27 0,03 37794879 261,2 TRUE 1972 1307,626646 654,316961 0,165278 2 197,8312555 58,75 1 40,9 3,052274502 0,32762453 0 VVGDEGPLSPK ICPL (K); ICPL (N- term)

gi|21224893 TRUE 5363 1446,726346 723,866811 0,120978 2 470,5827079 79,48 1 24,3 4,36948231 0,228860064 0 FTLTEALVSAGK ICPL (K); ICPL (N- term)

gi|21224893 TRUE 3232 1539,7339 770,370588 -0,031376 2 299,7564053 71,46 1 11,9 3,785392744 0,264173381 0 QFIGTTQHEFAR ICPL (N-term)

gi|21224893 TRUE 3438 1672,781278 836,894277 -0,011354 2 312,5568384 62,99 1 29 4,209242278 0,23757245 0 ESSEALATGTDFALR ICPL (N-term)

gi|21224893 TRUE 2125 1684,770017 842,8886465 -0,059093 2 208,9948469 42,72 1 14,9 4,530797863 0,220711678 0 LNEETEEAIEVAK ICPL (K); ICPL (N-term)

gi|21224893 TRUE 2048 1797,828947 899,4181115 -0,031423 2 203,1389467 106,37 1 29,9 4,967277159 0,201317536 0 EAAEFALAGSDEDVLR ICPL (N-term)

gi|21224893 TRUE 5273 2110,056333 704,0236283 0,209915 3 463,9325826 102,73 1 43,4 7,017861747 0,142493545 0 TAAAAALVGNADSVATFLGER ICPL (N-term)

gi|21224893 TRUE 5146 2362,146207 788,0535863 0,178241 3 455,0538242 34,48 1 13,2 1,786117485 0,559873585 0 AAEAALTDGSAEALHTFLHVK ICPL (K); ICPL (N-term)

gi|21224893 TRUE 5274 2503,217084 835,077212 0,126564 3 464,032141 153,33 1 17,4 3,815053896 0,262119495 0 LAALASDAAEAAGNAAAGAAVDSAAAAR ICPL (N-term)

TRUE succinyl-CoA synthetase subunit beta

[S. coelicolor A3(2)] gi|21223186 749,9238177 32,74111675 21 11 0,68 0,05 11 1,55 0,11 48246424 766,7 TRUE 3681 815,412519 815,412519 0,035781 1 326,7831717 28,52 1 6,7 1,025424584 0,975205798 0 DLFAK ICPL:13C(6) (K); ICPL:13C(6) (N-term)

gi|21223186 TRUE 3685 815,412519 408,2098975 0,078005 2 326,8359632 28,48 1 18,8 1,025424584 0,975205798 0 DLFAK ICPL:13C(6) (K); ICPL:13C(6) (N-term)

gi|21223186 TRUE 2043 1086,489072 543,748174 0,119852 2 202,6652224 45,85 1 53 0,82727503 1,208787844 0 FPAEVADK ICPL (K); ICPL (N-term)

gi|21223186 TRUE 2036 1098,52934 549,768308 0,069784 2 202,5841637 39,92 1 54,1 0,82727503 1,208787844 0 FPAEVADK ICPL:13C(6) (K); ICPL:13C(6) (N-term)

gi|21223186 TRUE 1669 1163,543943 582,2756095 0,177781 2 175,3313221 36,41 1 31,6 0,480594273 2,080757214 0 LDGNNAELGR ICPL (N-term)

gi|21223186 TRUE 1698 1184,600126 592,803701 0,116198 2 177,5108971 81,45 1 56,7 0,692193848 1,44468201 0 LAASADEAVAR ICPL:13C(6) (N-term)

gi|21223186 TRUE 1828 1270,569837 635,7885565 0,145487 2 187,2639472 34,91 1 36,4 0,670241778 1,491998907 0 VSLDDNAEFR ICPL (N-term)

gi|21223186 TRUE 1831 1270,569837 635,7885565 0,171087 2 187,4243803 52,43 1 30 0,670241778 1,491998907 0 VSLDDNAEFR ICPL (N-term)

gi|21223186 TRUE 3691 1314,64372 657,825498 0,144804 2 327,286472 47,31 1 31,4 0,598783072 1,670053894 0 ATDILGMDIK ICPL:13C(6) (K); ICPL:13C(6) (N-term); Oxidation (M)

gi|21223186 TRUE 3745 1382,695066 691,851171 0,125258 2 330,9769381 123,92 1 44,2 0,643818961 1,553231669 0 VVSGDVIALDGK ICPL (K); ICPL (N-term)

gi|21223186 TRUE 4701 1481,785917 741,3965965 0,079407 2 421,4261991 66,15 1 49 0,670540719 1,491333743 0 ILTDANHPLVQR ICPL (N-term)

gi|21223186 TRUE 4700 1487,806058 496,60687 0,06879 3 421,5537746 35,14 1 37,9 0,670540719 1,491333743 0 ILTDANHPLVQR ICPL:13C(6) (N-term)

gi|21223186 TRUE 2095 1565,748212 783,377744 0,008312 2 206,9944635 79,04 1 40,4 0,768031358 1,302030171 0 TPIDAIDGVTPEK ICPL (K); ICPL (N-term)

gi|21223186 TRUE 2094 1577,78848 789,397878 -0,006356 2 206,8353888 92,67 1 41,4 0,768031358 1,302030171 0 TPIDAIDGVTPEK ICPL:13C(6) (K); ICPL:13C(6) (N-term)

gi|21223186 TRUE 2098 1577,78848 526,6010107 0,012568 3 207,1624053 60,04 1 6 0,768031358 1,302030171 0 TPIDAIDGVTPEK ICPL:13C(6) (K); ICPL:13C(6) (N-term)

gi|21223186 TRUE 3997 1632,867032 816,937154 0,060692 2 347,7390634 92,12 1 33,9 0,415175682 2,40861891 0 EDALLVEVNPLAK ICPL:13C(6) (K); ICPL:13C(6) (N-term)

gi|21223186 TRUE 1886 1993,99777 665,3374407 0,228478 3 191,446672 40,2 1 10,2 0,701195799 1,426135184 0 HDVPVLAGEVIDTPEAAR ICPL (N-term)

TRUE oligopeptide-binding lipoprotein

[S. coelicolor A3(2)] gi|21223834 721,7899309 26,83333333 24 13 1,1 0,07 13 0,96 0,06 82208660 554,4 TRUE 3314 821,451566 411,229421 0,021558 2 304,3947301 38,38 1 20,8 0,808309538 1,237149821 0 DLTLVR ICPL (N-term)

gi|21223834 TRUE 3311 827,471708 414,239492 0,021216 2 304,2603136 35,29 1 34,8 0,808309538 1,237149821 0 DLTLVR ICPL:13C(6) (N-term)

gi|21223834 TRUE 3182 1022,494153 511,7507145 0,057771 2 296,5228384 26,9 2 26,1 1,61385996 0,619632449 0 TYTYTLR ICPL (N-term)

gi|21223834 TRUE 1734 1263,611631 632,3094535 0,148693 2 179,868697 77,76 1 13,6 0,652266868 1,533114817 0 QIATEINHK ICPL (K); ICPL (N-term)

gi|21223834 TRUE 4827 1423,696424 712,35185 -0,0001 2 431,1827165 57,34 1 22,8 1,194135627 0,837425815 0 ANLDNPVSGYIR ICPL (N-term)

gi|21223834 TRUE 4826 1423,696424 712,35185 0,0019 2 430,9471661 44,24 1 15,2 1,194135627 0,837425815 0 ANLDNPVSGYIR ICPL (N-term)

gi|21223834 TRUE 3420 1423,696424 712,35185 0,1013 2 311,1292469 33,11 1 49,1 0,863275759 1,158378409 0 ANLDNPVSGYIR ICPL (N-term)

gi|21223834 TRUE 5388 1536,736906 768,872091 -0,022982 2 471,8018077 97,69 1 60,9 1,233894952 0,810441763 0 IDGLFADYLTAK ICPL (K); ICPL (N-term)

gi|21223834 TRUE 1115 1536,736906 768,872091 0,031818 2 112,0210411 103,16 1 10,7 1,0423319 0,959387312 0 IDGLFADYLTAK ICPL (K); ICPL (N-term)

gi|21223834 TRUE 5387 1548,777173 774,8922245 0,050551 2 471,6732994 101,84 1 28,2 1,233894952 0,810441763 0 IDGLFADYLTAK ICPL:13C(6) (K); ICPL:13C(6) (N-term)

gi|21223834 TRUE 1842 1705,708875 853,3580755 -0,044351 2 188,23928 77,06 1 19 1,116525846 0,895635335 0 FASTQDADSWDTTR ICPL (N-term)

gi|21223834 TRUE 4758 1705,865629 853,4364525 -0,022905 2 426,3078247 95,92 1 32,2 0,992292992 1,007766867 0 AVLYGADHVSLQTAR ICPL (N-term)

gi|21223834 TRUE 4755 1705,865629 853,4364525 -0,019105 2 426,1439581 71,01 1 1,3 0,992292992 1,007766867 0 AVLYGADHVSLQTAR ICPL (N-term)

gi|21223834 TRUE 5335 1843,901365 922,4543205 -0,049641 2 468,7730077 50,38 1 9,4 1,384806073 0,722122772 0 VWAQDVLSGGPTYLK ICPL (K); ICPL (N-term)

gi|21223834 TRUE 1922 2124,972013 708,9955217 -0,330965 3 194,215289 64,57 1 18,6 1,109721021 0,901127384 0 TEPGAAGAETTPDLATDVAK ICPL (K); ICPL (N-term)

gi|21223834 TRUE 1921 2137,012281 713,0089443 0,075167 3 194,3189888 40,33 1 22,2 1,109721021 0,901127384 0 TEPGAAGAETTPDLATDVAK ICPL:13C(6) (K); ICPL:13C(6) (N-term)

gi|21223834 TRUE 838 2208,093122 736,702558 -0,003474 3 93,91723252 50,59 1 21,6 1,329354635 0,752244716 0 LIAGDYDIDLAQTGLSPQGR ICPL (N-term)

gi|21223834 TRUE 5133 2208,093122 736,702558 0,300126 3 453,8955661 60,05 1 34,2 1,004158692 0,995858531 0 LIAGDYDIDLAQTGLSPQGR ICPL (N-term)

TRUE molecular chaperone DnaK

[S. coelicolor A3(2)] gi|32141213 678,9747073 20,22653722 16 12 0,48 0,03 12 2,17 0,12 41886772 578,8 TRUE 3843 1045,535306 523,271291 0,037618 2 337,2889136 46,81 1 44,7 0,580398557 1,722953972 0 VVDYLVK ICPL (K); ICPL (N-term)

gi|32141213 TRUE 3842 1057,575574 1057,575574 -0,028074 1 337,1637888 29,33 1 27,6 0,580398557 1,722953972 0 VVDYLVK ICPL:13C(6) (K); ICPL:13C(6) (N-term)

gi|32141213 TRUE 3839 1057,575574 529,291425 0,11555 2 336,9957381 49,55 1 61,1 0,580398557 1,722953972 0 VVDYLVK ICPL:13C(6) (K); ICPL:13C(6) (N-term)

gi|32141213 TRUE 4790 1177,619169 589,3132225 0,108955 2 428,7862493 26,6 1 30,5 0,406224891 2,461690609 0 TPFHNVIK ICPL:13C(6) (K); ICPL:13C(6) (N-term)

gi|32141213 TRUE 4981 1230,615354 615,811315 0,08157 2 442,5071746 74,5 1 25,1 0,385609797 2,593295109 0 TTPSVVAFAK ICPL (K); ICPL (N-term)

gi|32141213 TRUE 1878 1233,546094 617,276685 0,14903 2 190,6377717 67,47 1 28 0,396685109 2,520891197 0 DAESYLGEK ICPL:13C(6) (K); ICPL:13C(6) (N-term)

gi|32141213 TRUE 1973 1336,693448 668,850362 0,155676 2 197,8153968 82,21 1 29,2 0,47871057 2,088944892 0 NGEVLVGEVAK ICPL:13C(6) (K); ICPL:13C(6) (N-term)

gi|32141213 TRUE 3525 1352,726392 676,866834 0,127732 2 317,4517557 87,17 1 25,9 0,432769084 2,310701106 0 EAGEIAGLNVLR ICPL:13C(6) (N-term)

gi|32141213 TRUE 1760 1353,683591 677,3454335 0,050533 2 181,8043723 54,25 1 10,9 0,307906179 3,247742557 1 LKGEDTAEIR ICPL:13C(6) (K); ICPL:13C(6) (N-term)

gi|32141213 TRUE 2140 1385,658307 693,3327915 0,102017 2 210,2274805 69,27 1 18,5 0,539550265 1,853395439 0 TEVESAVAELK ICPL (K); ICPL (N-term)

gi|32141213 TRUE 2139 1397,698575 699,3529255 0,063949 2 210,2099968 68,62 1 58,6 0,539550265 1,853395439 0 TEVESAVAELK ICPL:13C(6) (K); ICPL:13C(6) (N-term)

gi|32141213 TRUE 5120 1653,823087 827,4151815 0,090437 2 452,7091661 53,99 1 107,9 0,556439434 1,797140782 0 AQFQQLTSDLLER ICPL (N-term)

gi|32141213 TRUE 826 1653,823087 827,4151815 0,108837 2 92,73114132 72,96 1 25,1 0,580207799 1,723520437 0 AQFQQLTSDLLER ICPL (N-term)

gi|32141213 TRUE 5355 1856,936872 928,972074 -0,008148 2 470,057333 60,91 1 18,5 0,502758623 1,989026055 0 DFNPQQISAFVLQK ICPL:13C(6) (K); ICPL:13C(6) (N-term)

gi|32141213 TRUE 693 2177,12927 726,381274 0,066978 3 83,09993279 57,65 1 43 0,582019514 1,718155449 0 DAGIQLSEIDHVVLVGGSTR ICPL:13C(6) (N-term)

TRUE secreted protein

[S. coelicolor A3(2)] gi|21218846 675,9629855 30,82352941 15 11 0,15 0,04 11 10,94 2,65 44374628 471,6 TRUE 4875 872,439664 436,72347 0,06186 2 434,8479997 25,32 1 28,2 0,071837225 13,92035958 0 FYIYR ICPL:13C(6) (N-term)

gi|21218846 TRUE 3468 1024,550092 512,778684 0,051832 2 313,8955221 58,58 1 30,2 0,063473754 15,75454322 0 ATVGLDVK ICPL:13C(6) (K); ICPL:13C(6) (N-term)

gi|21218846 TRUE 1783 1081,504453 541,2558645 0,111871 2 183,7401973 50,48 1 38,5 0,037523388 26,65004525 0 YADGEFIR ICPL:13C(6) (N-term)

gi|21218846 TRUE 3377 1150,545377 575,7763265 0,136947 2 308,6067386 48,25 1 21 0,241013172 4,149150822 0 LEYTSTSK ICPL:13C(6) (K); ICPL:13C(6) (N-term)

gi|21218846 TRUE 3828 1319,620984 660,31413 0,06694 2 336,290105 78,89 1 65,8 0,056222508 17,78647079 0 QDGSGTFDLIGV ICPL:13C(6) (N-term)

gi|21218846 TRUE 1667 1414,636289 707,8217825 0,070835 2 175,2154891 50,84 1 63,2 0,133384225 7,497138448 0 TVPCAEDDLQR ICPL:13C(6) (N-term)

gi|21218846 TRUE 3151 1452,663175 726,8352255 -0,012051 2 294,7238138 51,7 1 26,2 0,317430444 3,15029645 0 VTGTYGSLNCNR ICPL:13C(6) (N-term)

gi|21218846 TRUE 3570 1497,719327 749,3633015 -0,001203 2 319,8065968 100,65 1 60,1 0,129053678 7,748713656 0 AQALLDCGSAAAK ICPL:13C(6) (K); ICPL:13C(6) (N-term)

gi|21218846 TRUE 4934 1542,762601 514,9257177 0,120847 3 438,850333 49 1 5,2 0,101723474 9,830572669 0 TTEVSSAITHFK ICPL:13C(6) (K); ICPL:13C(6) (N-term)

gi|21218846 TRUE 1524 1603,80142 802,404348 -0,143696 2 165,1707136 57,18 1 9,1 0,11309586 8,842056664 0 AGAAAPTAQQKPDR ICPL:13C(6) (K); ICPL:13C(6) (N-term)

gi|21218846 TRUE 1773 1659,71803 830,362653 -0,050906 2 182,6217808 31,38 1 33,8 0,390802423 2,558837766 0 CQDSVPADSIMAK ICPL:13C(6) (K); ICPL:13C(6) (N-term); Oxidation (M)

TRUE dihydrolipoamide dehydrogenase

[S. coelicolor A3(2)] gi|21220654 633,4837121 28,60082305 17 11 1,15 0,08 11 0,92 0,06 89110736 613,5 TRUE 4732 1005,54759 503,277433 -0,073266 2 424,1914823 65,06 1 92 1,748340812 0,571970861 0 GLQGLVASR ICPL (N-term)

gi|21220654 TRUE 1788 1133,489805 567,2485405 0,058719 2 183,8841717 38,42 1 24 0,995117534 1,004906421 0 ESEQFGVK ICPL (K); ICPL (N-term)

gi|21220654 TRUE 1714 1220,521833 610,7645545 0,169691 2 178,5442885 33,51 1 14,9 0,969302545 1,031669633 0 AEYTQDGVK ICPL (K); ICPL (N-term)

gi|21220654 TRUE 6659 1311,61569 656,311483 0,131234 2 592,7795216 65,5 1 110,3 1,280704457 0,78082027 0 FNLGTFFQK ICPL (K); ICPL (N-term)

gi|21220654 TRUE 1804 1322,637556 661,822416 0,123568 2 184,9608474 69,16 1 46,9 1,347829302 0,741933714 0 TVPVDYDGVPR ICPL (N-term)

gi|21220654 TRUE 6660 1323,655958 662,331617 0,074766 2 592,764247 55,05 1 90,7 1,280704457 0,78082027 0 FNLGTFFQK ICPL:13C(6) (K); ICPL:13C(6) (N-term)

gi|21220654 TRUE 4816 1331,710645 666,3589605 0,088679 2 430,4944994 69,58 1 29,2 1,205384055 0,829611107 0 HVLLATGSVPK ICPL (K); ICPL (N-term)

gi|21220654 TRUE 4815 1343,750913 672,3790945 0,047211 2 430,3376823 67,85 1 29 1,205384055 0,829611107 0 HVLLATGSVPK ICPL:13C(6) (K); ICPL:13C(6) (N-term)

gi|21220654 TRUE 3356 1387,704328 694,355802 0,074596 2 307,4114714 52,89 1 5,6 1,033506077 0,967580184 1 KITYIEGEGR ICPL:13C(6) (K); ICPL:13C(6) (N-term)

gi|21220654 TRUE 3042 1438,683942 719,845609 0,247982 2 287,87868 28,17 1 16,7 0,67325607 1,485318951 0 DGAVVGVHMVGDR ICPL:13C(6)

(N-term); Oxidation (M)

gi|21220654 TRUE 3191 1471,728788 736,368032 0,039536 2 297,0377552 50,75 1 3,1 1,047197331 0,95492986 0 ALLHAGEVADQSR ICPL (N-term)

gi|21220654 TRUE 3192 1471,728788 736,368032 0,039536 2 297,0505888 72,52 1 32,2 1,047197331 0,95492986 0 ALLHAGEVADQSR ICPL (N-term)

gi|21220654 TRUE 1743 1563,707395 782,3573355 0,034529 2 180,6707141 49,23 1 22,7 1,07294406 0,932015039 0 HLVPVEDENSSK ICPL (K); ICPL (N-term)

gi|21220654 TRUE 3298 1993,943632 665,3193947 0,093916 3 303,4921802 50,13 1 15,3 1,216226407 0,822215333 0 VTYCHPEVASVGLTEAR ICPL (N-term)

TRUE secreted protein

[S. coelicolor A3(2)] gi|21220594 548,3958081 11,2407211 11 8 2,19 0,11 8 0,47 0,02 42182839 306,8 TRUE 3537 1045,510145 523,2587105 -0,001821 2 317,7497637 52,04 1 19,1 2,006631076 0,498347709 0 YGVLAGQK ICPL (K); ICPL (N-term)

gi|21220594 TRUE 3513 1045,510145 523,2587105 0,049379 2 316,565297 65,22 1 27,3 2,273511464 0,439848233 0 YGVLAGQK ICPL (K); ICPL (N-term)

gi|21220594 TRUE 3515 1057,550413 529,2788445 0,158911 2 316,6661221 48,95 1 15,6 2,273511464 0,439848233 0 YGVLAGQK ICPL:13C(6) (K); ICPL:13C(6) (N-term)

gi|21220594 TRUE 3489 1095,587202 548,297239 0,105922 2 315,114072 40,6 1 12 1,923620115 0,519853162 0 TADVQLVK ICPL:13C(6) (K); ICPL:13C(6) (N-term)

gi|21220594 TRUE 3491 1438,73248 719,869878 0,139244 2 315,212664 117,53 1 69,9 2,801258213 0,356982443 0 VLAGGEYVVAEAR ICPL (N-term)

gi|21220594 TRUE 5009 1520,793516 760,900396 0,099608 2 444,707741 34,46 1 33 2,23092778 0,448244004 0 VVPPFTTANQPK ICPL:13C(6) (K); ICPL:13C(6) (N-term)

gi|21220594 TRUE 3595 1814,870817 907,9390465 -0,012093 2 321,5446384 87,46 1 19,8 2,079297558 0,480931647 0 NGGFATAVGTPDVVTAK ICPL (K); ICPL (N-term)

gi|21220594 TRUE 1588 1824,741941 912,8746085 0,044383 2 169,5672304 84,4 1 14,3 1,747014562 0,572405074 0 GSYYNSGNIDGSGEGSR ICPL (N-term)

gi|21220594 TRUE 1750 1942,892972 971,950124 -0,111648 2 180,9853301 84,03 1 56 2,430247158 0,411480782 0 AYPEDGAYAGTGVPVQSR ICPL (N-term)

TRUE secreted hydrolase

[S. coelicolor A3(2)] gi|21224438 535,5038177 29,38856016 10 9 2,13 0,51 9 0,71 0,17 40797256 310,5 TRUE 4872 825,425352 413,216314 0,031372 2 434,2428578 40,78 1 73 1,210313064 0,826232509 0 LDFAVR ICPL (N-term)

gi|21224438 TRUE 3354 1043,515623 522,2614495 0,085101 2 307,4852138 51,11 1 42,5 1,716148144 0,582700278 0 LTIGTDYR ICPL (N-term)

gi|21224438 TRUE 1583 1213,559614 607,283445 0,15951 2 169,2960053 67,01 1 16,5 0,776485245 1,287854478 0 ADSGVANGYVR ICPL (N-term)

gi|21224438 TRUE 3187 1317,654584 659,33093 0,14654 2 297,0964469 56,61 1 31,7 1,6555341 0,604034674 0 DGAPQLVGVNSR ICPL (N-term)

gi|21224438 TRUE 3325 1393,722257 697,3647665 0,168067 2 305,1889973 28,17 1 42,8 1,015850929 0,984396403 0 EVVELVPHPNR ICPL (N-term)

gi|21224438 TRUE 3352 2093,0258 1047,016538 0,175724 2 306,7945552 51,11 1 6,7 4,09196628 0,24438129 1 LTGTTDVNADGRDDLVAVR ICPL (N-term)

gi|21224438 TRUE 2171 2213,010526 738,3416927 0,160822 3 212,7320634 28,94 1 15,2 2,43307992 0,411001707 0 DLGTGNLSDLVASADFNADGR ICPL

(N-term)

gi|21224438 TRUE 5249 2702,430067 901,4815397 -0,044819 3 462,2610658 88,37 1 6,1 0,887324047 1,126983996 0 AVPDIAPVPLAAGAPDPGAPLTAVGFGR ICPL (N-term)

gi|21224438 TRUE 5163 3135,469419 1045,82799 -0,176071 3 455,9762247 101,48 1 11,4 5,682633331 0,175974754 0 TDIAAVLDDGSLHAFYTKPDGTLEYGR ICPL (K); ICPL (N-term)

TRUE isocitrate dehydrogenase

[S. coelicolor A3(2)] gi|21225286 512,3576355 14,61434371 17 8 1,27 0,05 8 0,8 0,03 52249559 405,8 TRUE 2133 1195,599348 598,303312 0,080576 2 210,0055307 55,43 1 21,2 1,528586094 0,654199331 0 ILDPVEATK ICPL (K); ICPL (N-term)

gi|21225286 TRUE 2131 1207,639615 604,3234455 0,079709 2 209,5831557 50,11 1 26,8 1,528586094 0,654199331 0 ILDPVEATK ICPL:13C(6) (K); ICPL:13C(6) (N-term)

gi|21225286 TRUE 3763 1422,668836 711,838056 0,142888 2 332,4562053 95,68 1 23,8 1,144481091 0,873758429 0 TFAQYGDVLAK ICPL (K); ICPL (N-term)

gi|21225286 TRUE 3766 1434,709104 717,85819 0,12102 2 332,5066298 27,66 1 38,9 1,144481091 0,873758429 0 TFAQYGDVLAK ICPL:13C(6) (K); ICPL:13C(6) (N-term)

gi|21225286 TRUE 2063 1452,700498 726,853887 0,071226 2 204,8414218 38,7 1 19,6 1,356213857 0,737346839 0 GLESLPEGAEIK ICPL (K); ICPL (N-term)

gi|21225286 TRUE 2064 1464,740766 732,874021 0,064958 2 204,4616219 39,14 1 22,3 1,356213857 0,737346839 0 GLESLPEGAEIK ICPL:13C(6) (K); ICPL:13C(6) (N-term)

gi|21225286 TRUE 6550 1490,800153 745,9037145 0,005171 2 585,9871302 51,51 1 53,1 1,238510426 0,807421544 0 LPNISASIPQLK ICPL (K); ICPL (N-term)

gi|21225286 TRUE 6549 1502,840421 751,9238485 0,089303 2 585,8837056 45,54 1 6,7 1,238510426 0,807421544 0 LPNISASIPQLK ICPL:13C(6) (K); ICPL:13C(6) (N-term)

gi|21225286 TRUE 3692 1585,764524 793,3859 0,0676 2 327,3637802 49,49 1 14,8 1,408187174 0,71013287 0 AGLTPNDGLGGIYK ICPL (K); ICPL

(N-term)

gi|21225286 TRUE 3640 1593,811887 797,4095815 0,015437 2 324,3718885 64,94 1 20,6 1,077974569 0,927665669 0 IELVGDDGTTTVLR ICPL (N-term)

gi|21225286 TRUE 3641 1593,811887 797,4095815 0,044037 2 324,6070053 97,01 1 24,2 1,077974569 0,927665669 0 IELVGDDGTTTVLR ICPL (N-term)

gi|21225286 TRUE 3690 1597,804792 799,406034 0,042932 2 327,201097 44,02 1 3,3 1,408187174 0,71013287 0 AGLTPNDGLGGIYK ICPL:13C(6) (K); ICPL:13C(6) (N-term)

gi|21225286 TRUE 3958 1786,864606 893,935941 0,028318 2 344,9856549 91,58 1 43,8 1,286543056 0,777276746 0 AFAPLAETLTASEQK ICPL (K); ICPL

(N-term)

gi|21225286 TRUE 3957 1798,904874 899,956075 0,01385 2 345,0732133 104,38 1 33,2 1,286543056 0,777276746 0 AFAPLAETLTASEQK ICPL:13C(6) (K); ICPL:13C(6) (N-term)

gi|21225286 TRUE 5262 2257,155469 753,0566737 0,030979 3 463,6920077 29,81 1 22,1 1,087892237 0,919208692 0 LVDAAGNAVLEQTVSAGDIFR ICPL:13C(6) (N-term)

TRUE secreted esterase

[S. coelicolor A3(2)] gi|21224523 484,9309952 11,44010767 11 7 0,3 0,05 7 4,06 0,72 24688939 466,5 TRUE 4855 922,545209 461,7762425 0,022915 2 433,0013826 40,64 1 28,7 0,278811248 3,586655879 0 VVELVPR ICPL:13C(6) (N-term)

gi|21224523 TRUE 1609 1128,573957 564,7906165 0,117567 2 171,2324304 99,53 1 74,6 0,139250463 7,181304656 0 ADLTGTGGAVR ICPL:13C(6) (N-term)

gi|21224523 TRUE 1543 1149,601087 575,3041815 0,147437 2 166,7102384 32,03 1 23,3 0,296919058 3,367921236 1 TDRDVVLAR ICPL (N-term)

gi|21224523 TRUE 1539 1155,621229 578,3142525 0,042095 2 166,363464 39,04 1 87,9 0,296919058 3,367921236 1 TDRDVVLAR ICPL:13C(6) (N-term)

gi|21224523 TRUE 1544 1155,621229 385,8785937 0,078319 3 166,6341803 30,61 1 21,2 0,296919058 3,367921236 1 TDRDVVLAR ICPL:13C(6) (N-term)

gi|21224523 TRUE 4879 1161,68176 581,344518 0,122364 2 434,8505911 61,15 1 77,7 0,267030915 3,744884747 1 KVVELVPR ICPL:13C(6) (K); ICPL:13C(6) (N-term)

gi|21224523 TRUE 3874 1386,632459 693,8198675 0,038265 2 339,641872 65,51 1 6,3 0,207709468 4,814417037 0 VDDLGSWVASK ICPL (K); ICPL (N-term)

gi|21224523 TRUE 3872 1398,672727 699,8400015 0,108597 2 339,4745386 81,5 1 55,5 0,207709468 4,814417037 0 VDDLGSWVASK ICPL:13C(6) (K); ICPL:13C(6) (N-term)

gi|21224523 TRUE 1696 1758,74835 879,877813 -0,060026 2 177,3310634 98,64 1 56,7 0,689603151 1,450109384 0 SYQGGCYGIDAAETR ICPL:13C(6)

(N-term)

gi|21224523 TRUE 6396 3248,736906 1083,583819 -0,206158 3 574,3538054 68,43 1 16,4 0,25062858 3,989967947 0 LNRPVTNVTPLALATAAPTAGEELTLAGYGR ICPL:13C(6) (N-term)

TRUE tellurium resistance protein

[S. coelicolor A3(2)] gi|32141118 480,9869089 48,16753927 14 8 0,98 0,06 8 1,05 0,07 76563038 750,4 TRUE 3139 725,372907 725,372907 0,044593 1 293,9720384 24,75 1 28,9 0,989856308 1,010247641 0 NAFIR ICPL (N-term)

gi|32141118 TRUE 2990 983,469358 492,238317 -0,008034 2 284,6239717 59,95 1 40,4 0,975943761 1,024649206 0 GQSFGQVR ICPL (N-term)

gi|32141118 TRUE 3567 1386,737577 693,8724265 0,119947 2 319,6636554 51,87 1 76,1 0,837919475 1,193432102 0 VNLVAVPAEVDR ICPL (N-term)

gi|32141118 TRUE 3565 1386,737577 693,8724265 0,124747 2 319,5802224 26,76 1 115,4 0,837919475 1,193432102 0 VNLVAVPAEVDR ICPL (N-term)

gi|32141118 TRUE 1595 1460,724027 730,8656515 0,109697 2 169,9851888 67,17 1 67,1 1,169395403 0,855142749 0 VVNQANNQELAR ICPL (N-term)

gi|32141118 TRUE 1593 1460,724027 730,8656515 0,118297 2 170,1131802 47,29 1 97,5 1,169395403 0,855142749 0 VVNQANNQELAR ICPL (N-term)

gi|32141118 TRUE 4901 1601,80705 801,407163 0,106074 2 436,7477074 86,5 1 100,3 1,164889985 0,858450165 0 IVFPVSIHDAENR ICPL (N-term)

gi|32141118 TRUE 4902 1601,80705 801,407163 0,106074 2 436,7616157 93,49 1 77,7 1,164889985 0,858450165 0 IVFPVSIHDAENR ICPL (N-term)

gi|32141118 TRUE 6666 2041,997785 1021,502531 -0,340861 2 593,1414224 41,42 1 19,5 1,067769257 0,936531927 0 AVGQGYASGLAGIAADFGVNV

ICPL (N-term)

gi|32141118 TRUE 2337 2368,0827 790,0324173 -0,107152 3 222,6272224 27,77 1 7,6 1,011943991 0,988196984 0 TTTGTDYDLDASALLLDTSGK ICPL (K); ICPL (N-term)

gi|32141118 TRUE 3926 2368,0827 790,0324173 0,037748 3 343,1702298 53,68 1 51,2 0,648457433 1,542121271 0 TTTGTDYDLDASALLLDTSGK ICPL (K); ICPL (N-term)

gi|32141118 TRUE 3923 2368,0827 1184,544988 0,068624 2 342,75468 139,69 1 21,9 0,648457433 1,542121271 0 TTTGTDYDLDASALLLDTSGK ICPL (K); ICPL (N-term)

gi|32141118 TRUE 3922 2380,122968 1190,565122 -0,123244 2 342,6626389 144,86 1 26,7 0,648457433 1,542121271 0 TTTGTDYDLDASALLLDTSGK ICPL:13C(6) (K); ICPL:13C(6) (N-term)

TRUE tellurium resistance protein

[S. coelicolor A3(2)] gi|21222670 479,14 59,16230366 23 10 0,74 0,06 10 1,45 0,12 103231282 812,6 TRUE 2988 1040,490812 520,749044 0,016912 2 284,4845968 44,95 1 29,7 0,772710201 1,294146238 0 SQNFGQVR ICPL (N-term)

gi|21222670 TRUE 2987 1040,490812 520,749044 0,016912 2 284,4516634 25,13 1 58 0,772710201 1,294146238 0 SQNFGQVR ICPL (N-term)

gi|21222670 TRUE 3308 1317,690931 659,3491035 0,072593 2 304,4964549 57,79 1 116 0,563351039 1,775092136 0 ILNQAGGAEIAR ICPL (N-term)

gi|21222670 TRUE 3310 1317,690931 659,3491035 0,151793 2 304,2996138 88,56 1 63,3 0,563351039 1,775092136 0 ILNQAGGAEIAR ICPL (N-term)

gi|21222670 TRUE 4842 1522,790006 761,898641 0,019918 2 432,117741 57,02 1 18,9 0 IVFPVSIYDAENR

gi|21222670 TRUE 880 1627,81146 814,409368 0,022464 2 96,99779946 73,11 1 8,3 0,934719554 1,069839607 0 IVFPVSIYDAENR ICPL (N-term)

gi|21222670 TRUE 881 1627,81146 814,409368 0,054664 2 96,84890827 95,69 1 25 0,934719554 1,069839607 0 IVFPVSIYDAENR ICPL (N-term)

gi|21222670 TRUE 5173 1627,81146 814,409368 0,117864 2 456,6961495 71,27 1 135 0,788367055 1,268444684 0 IVFPVSIYDAENR ICPL (N-term)

gi|21222670 TRUE 5174 1627,81146 814,409368 0,117864 2 456,7119325 78,99 1 58,2 0,788367055 1,268444684 0 IVFPVSIYDAENR ICPL (N-term)

gi|21222670 TRUE 5230 1633,831602 817,419439 0,169722 2 460,7841079 44,28 1 16,3 1,006600992 0,993442295 0 IVFPVSIYDAENR ICPL:13C(6) (N-term)

gi|21222670 TRUE 1496 1672,767393 836,8873345 0,069731 2 163,4364555 67,63 1 42,4 0,583772293 1,712996681 0 QTPDNTIVHTGDNR ICPL (N-term)

gi|21222670 TRUE 1494 1672,767393 836,8873345 0,075531 2 163,3295467 46,37 1 61,5 0,583772293 1,712996681 0 QTPDNTIVHTGDNR ICPL (N-term)

gi|21222670 TRUE 5361 2129,029828 1065,018552 0,074096 2 470,2615581 43,4 1 17,5 0,524953147 1,90493191 0 AVGQGYASGLTGIAQDFGVNV

ICPL (N-term)

gi|21222670 TRUE 5357 2129,029828 710,3481267 0,20782 3 470,033541 37,98 1 36,9 0,524953147 1,90493191 0 AVGQGYASGLTGIAQDFGVNV ICPL (N-term)

gi|21222670 TRUE 3714 2336,067731 779,360761 0,107217 3 328,7707301 61,88 1 14,1 0,535870324 1,866123117 0 TTTGTDFDLDASAIAVNTQGK

ICPL (K); ICPL (N-term)

gi|21222670 TRUE 3710 2348,107999 783,3741837 0,119149 3 328,3741637 52,99 1 14,9 0,535870324 1,866123117 0 TTTGTDFDLDASAIAVNTQGK ICPL:13C(6) (K); ICPL:13C(6) (N-term)

gi|21222670 TRUE 2236 2591,229856 864,4148027 -0,128408 3 216,6833968 77,03 1 11,4 0,612705756 1,632104791 0 TGEGAGDDEAINVNLAGLPADIEK ICPL:13C(6) (K); ICPL:13C(6) (N-term)

gi|21222670 TRUE 2250 2591,229856 864,4148027 -0,094808 3 217,4828805 55,81 1 1,9 1,07077083 0,933906651 0 TGEGAGDDEAINVNLAGLPADIEK ICPL:13C(6) (K); ICPL:13C(6) (N-term)

TRUE secreted 5'-nucleotidase

[S. coelicolor A3(2)] gi|21222550 479,1083671 21,89542484 13 7 1,59 0,14 7 0,66 0,06 49810658 313,8 TRUE 2042 1433,621931 717,3146035 0,118593 2 202,6485637 57,43 1 46,8 1,496615797 0,668174158 0 FEDEVETINK ICPL (K); ICPL (N-term)

gi|21222550 TRUE 2041 1445,662198 723,334737 0,066926 2 202,5638304 55,98 1 25,4 1,496615797 0,668174158 0 FEDEVETINK ICPL:13C(6) (K); ICPL:13C(6) (N-term)

gi|21222550 TRUE 3441 1466,679792 733,843534 0,114332 2 312,3415882 51,71 1 22,9 1,353784715 0,738669885 0 LYTDTTLTYDR ICPL (N-term)

gi|21222550 TRUE 3419 1467,722639 734,3649575 0,088285 2 311,4156554 45,37 1 26,4 1,981425211 0,504687229 0 LNGAAIDPTATYR ICPL (N-term)

gi|21222550 TRUE 696 1720,865293 574,2932817 0,113155 3 83,22543307 31,68 1 6,5 2,078603867 0,481092148 0 TIDAGGVEYLATHLR ICPL (N-term)

gi|21222550 TRUE 3846 1848,84387 924,925573 0,062254 2 337,4038053 105,2 1 44,3 1,84466349 0,542104294 0 GADYPYLAANVLDEK ICPL (K); ICPL

(N-term)

gi|21222550 TRUE 3639 2462,121885 821,3788123 -0,093937 3 324,3149717 45,01 1 15 1,335194019 0,748954823 0 VASNSFLAGGGDGFTTLGEGTNER ICPL

(N-term)

gi|21222550 TRUE 6257 2476,27313 826,095894 0,016218 3 564,395472 54,61 1 21,1 1,051801477 0,950749758 0 WSTLAAPIGNRPIGYVSADINR ICPL

(N-term)

TRUE elongation factor gi|1091582 455,75 18,63979849 12 9 1,54 0,16 9 0,72 0,08 46630547 393,5 TRUE 3092 942,515546 471,761411 -0,089022 2 290,813705 33,36 1 47,8 1,676995032 0,596304688 0 EHVLLAR ICPL (N-term)

gi|1091582 TRUE 1679 1070,526548 535,766912 0,085176 2 176,0555221 60,94 1 16,4 1,112077389 0,899217995 0 TVGAGQVTK ICPL (K); ICPL (N-term)

gi|1091582 TRUE 3850 1410,726346 705,866811 0,104978 2 337,6232218 83,26 1 77,7 1,806050715 0,553694308 0 VNETVDIIGIK ICPL (K); ICPL (N-term)

gi|1091582 TRUE 3848 1422,766614 711,886945 0,10631 2 337,5394053 84,98 1 38,6 1,806050715 0,553694308 0 VNETVDIIGIK ICPL:13C(6) (K); ICPL:13C(6) (N-term)

gi|1091582 TRUE 4033 1777,92317 889,465223 0,021354 2 349,708897 56,81 1 12,7 0,821160325 1,217788987 0 VELIQPVAMEEGLK ICPL:13C(6) (K); ICPL:13C(6) (N-term)

gi|1091582 TRUE 3907 1781,87782 891,442548 -0,002496 2 341,6429968 67,84 1 29,1 1,017158435 0,983131011 0 VELIQPVAMEEGLK ICPL (K); ICPL

(N-term); Oxidation (M)

gi|1091582 TRUE 5126 1801,944257 901,4757665 -0,083733 2 453,0402909 102,08 1 35,6 1,829695052 0,546539162 0 LLDEGQAGENVGLLLR ICPL (N-term)

gi|1091582 TRUE 834 1801,944257 901,4757665 -0,005333 2 93,43307413 56,34 1 14,2 2,251530017 0,444142424 0 LLDEGQAGENVGLLLR ICPL (N-term)

gi|1091582 TRUE 819 1801,944257 901,4757665 0,029667 2 92,75555787 77,76 1 4,9 2,251530017 0,444142424 0 LLDEGQAGENVGLLLR ICPL (N-term)

gi|1091582 TRUE 3782 1801,944257 901,4757665 0,051067 2 333,5507968 105,72 1 45,7 1,607723309 0,621997575 0 LLDEGQAGENVGLLLR ICPL (N-term)

gi|1091582 TRUE 4021 2146,001523 1073,5044 -0,011599 2 349,1449221 102,91 1 45,9 1,650953299 0,605710653 0 ELLSEYEFPGDDVPVVK ICPL (K); ICPL

(N-term)

gi|1091582 TRUE 4023 2158,041791 1079,524534 -0,113267 2 349,1583888 94,11 1 24,9 1,650953299 0,605710653 0 ELLSEYEFPGDDVPVVK ICPL:13C(6) (K); ICPL:13C(6) (N-term)

TRUE secreted protein

[S. coelicolor A3(2)] gi|21224521 455,16 22,77777778 14 6 0,24 0,04 6 4,92 0,82 49827831 593,2 TRUE 1861 934,394117 467,7006965 0,006207 2 189,5043973 39,66 1 15,7 0,187606128 5,330316284 0 SFDVEK ICPL (K); ICPL (N-term)

gi|21224521 TRUE 1858 946,434385 946,434385 0,062115 1 189,3305717 31,11 1 44,3 0,187606128 5,330316284 0 SFDVEK ICPL:13C(6) (K); ICPL:13C(6) (N-term)

gi|21224521 TRUE 1855 946,434385 473,7208305 0,075139 2 189,1925051 41,87 1 118,4 0,187606128 5,330316284 0 SFDVEK ICPL:13C(6) (K); ICPL:13C(6) (N-term)

gi|21224521 TRUE 1755 1406,656034 469,556862 0,083214 3 181,5941557 55,77 1 28,1 0,149779046 6,676501335 0 GDGHIVLADCK ICPL:13C(6) (K); ICPL:13C(6) (N-term)

gi|21224521 TRUE 1753 1406,656034 703,831655 0,10629 2 181,2649221 48,77 1 90,5 0,149779046 6,676501335 0 GDGHIVLADCK ICPL:13C(6) (K); ICPL:13C(6) (N-term)

gi|21224521 TRUE 1640 1562,757139 781,8822075 -0,019415 2 173,4312805 69,2 1 41,2 0,26642385 3,753417724 1 RGDGHIVLADCK ICPL:13C(6) (K); ICPL:13C(6) (N-term)

gi|21224521 TRUE 3120 1562,757139 521,5905637 0,063709 3 292,6319136 54,3 1 32,5 0,126631302 7,896941639 1 RGDGHIVLADCK ICPL:13C(6) (K); ICPL:13C(6) (N-term)

gi|21224521 TRUE 3121 1562,757139 781,8822075 0,063785 2 292,64828 80,16 1 24,5 0,126631302 7,896941639 1 RGDGHIVLADCK ICPL:13C(6) (K); ICPL:13C(6) (N-term)

gi|21224521 TRUE 3550 1757,818479 879,4128775 -0,000755 2 318,7124053 107,17 1 55,7 0,31930302 3,131821303 0 NLWTAVGQTADEEGR

ICPL:13C(6) (N-term)

gi|21224521 TRUE 5211 2715,34683 905,7871273 0,048518 3 459,5326077 104,89 1 26,2 0,36765618 2,719932522 1 NLWTAVGQTADEEGRDFALLEIR ICPL:13C(6) (N-term)

TRUE secreted tripeptidylaminopeptidase

[S. coelicolor A3(2)] gi|21219739 448,7109952 13,4935305 7 5 6,08 0,33 5 0,167 0,009 32680618 284,8 TRUE 1794 835,430829 418,2190525 0,105295 2 184,3298805 41,84 1 60,1 4,951615546 0,20195429 0 DLDVIR ICPL (N-term)

gi|21219739 TRUE 3615 1291,574216 646,290746 0,158508 2 322,6608298 72,23 1 48,5 6,619320814 0,151072901 0 AYDFVGFDPR ICPL (N-term)

gi|21219739 TRUE 3538 1486,717252 743,862264 0,118072 2 317,8232554 89,72 1 29,8 6,624472599 0,150955413 0 VDTYLLTGGTDAR ICPL (N-term)

gi|21219739 TRUE 4776 1718,860883 859,9340795 0,007241 2 427,7635826 103,68 1 55,9 6,648954369 0,150399588 0 QGALIYNPGGPGGSGLR ICPL (N-term)

gi|21219739 TRUE 4777 1718,860883 573,625145 0,203565 3 427,9320743 97,19 1 34,5 6,648954369 0,150399588 0 QGALIYNPGGPGGSGLR ICPL (N-term)

gi|21219739 TRUE 1673 1761,819059 881,4131675 -0,006735 2 175,5923472 99,58 1 31,9 5,54194644 0,180442018 0 DAATPYEGAVELHQR ICPL (N-term)

TRUE SLPI=protease inhibitor [S. lividans, 66,

,Peptide, 107 aa] gi|257239 448,5569089 78,5046729 24 4 0,05 0,02 4 29,12 11,04 307639867 5442 TRUE 1463 934,48364 467,745458 0,080684 2 160,8747221 22,67 1 62,3 0,024938637 40,09842238 1 RLSYER ICPL:13C(6) (N-term)

gi|257239 TRUE 2130 1111,515043 556,2611595 0,047081 2 209,7503221 84,76 1 25,4 0,033699475 29,67405258 0 NAGSASVFTF ICPL:13C(6) (N-term)

gi|257239 TRUE 795 1885,953885 629,3228123 0,108263 3 90,61491627 57,01 1 16,7 0,030079773 33,24493206 0 EYAPVVVTVDGVWQGR ICPL:13C(6)

(N-term)

gi|257239 TRUE 794 1885,953885 943,4805805 0,108439 2 90,59768267 112,1 1 56,1 0,030079773 33,24493206 0 EYAPVVVTVDGVWQGR ICPL:13C(6) (N-term)

gi|257239 TRUE 3296 2579,243635 860,4193957 0,006313 3 303,4737888 67,83 1 30,9 0,136479578 7,32710355 0 AVTLTCAPTASGTHPAAAAACAELR ICPL:13C(6) (N-term)

TRUE aconitate hydratase

[S. coelicolor A3(2)] gi|21224335 431,457904 9,402654867 10 8 1,78 0,09 8 0,57 0,03 23342858 260,3 TRUE 4795 831,414777 831,414777 -0,003777 1 429,022549 25,37 1 12,6 2,167335568 0,461396018 0 YQFLR ICPL (N-term)

gi|21224335 TRUE 6495 1074,630567 1074,630567 -0,057167 1 581,9492635 25,53 1 2,7 2,077624515 0,481318926 0 VLLENLLR ICPL (N-term)

gi|21224335 TRUE 5222 1074,630567 537,8189215 0,065757 2 460,2976658 48,91 1 15,8 1,559082878 0,641402721 0 VLLENLLR ICPL (N-term)

gi|21224335 TRUE 6488 1074,630567 537,8189215 0,133957 2 581,6625136 65,26 1 80,2 2,077624515 0,481318926 0 VLLENLLR ICPL (N-term)

gi|21224335 TRUE 3704 1199,613401 600,3103385 0,113523 2 327,9198053 35,41 1 7,4 1,974316195 0,506504481 0 AGLTPYLDK ICPL:13C(6) (K); ICPL:13C(6) (N-term)

gi|21224335 TRUE 6338 1237,66876 619,338018 0,254564 2 570,434072 63,55 1 52,9 1,558685261 0,641566341 0 NGGILQYVLR ICPL (N-term)

gi|21224335 TRUE 1772 1404,606611 702,8069435 0,084113 2 182,6055307 51,28 1 35,4 1,786274857 0,55982426 0 IDTPGEADYYR ICPL (N-term)

gi|21224335 TRUE 3584 1748,812589 874,9099325 0,056335 2 320,5827717 73,34 1 7,4 1,73413249 0,576657208 0 STLQVGDESYEIFR ICPL (N-term)

gi|21224335 TRUE 3855 1797,844251 899,4257635 -0,039527 2 337,8435717 100,94 1 21,5 1,40936635 0,709538723 0 DALGVDQDGNPVFLK ICPL (K); ICPL

(N-term)

TRUE GroEL1

+[S. coelicolor] gi|406598 426,8969089 14,44444444 8 6 1,09 0,22 6 1,14 0,23 11957463 184,4 TRUE 3542 1039,520704 520,26399 -0,09238 2 318,0456634 42,9 1 12,6 0,964185393 1,037144938 0 VLEGNLGK ICPL (K); ICPL (N-term)

gi|406598 TRUE 3482 1128,587537 564,7974065 0,071387 2 314,7499136 24,88 1 18,8 0,552410824 1,810246934 0 GTFNAVAVK ICPL:13C(6) (K); ICPL:13C(6) (N-term)

gi|406598 TRUE 1999 1226,605161 613,8062185 0,209363 2 199,6636554 74,62 1 26,1 0,917028228 1,090478972 0 VGAATEVELK ICPL (K); ICPL (N-term)

gi|406598 TRUE 1996 1238,645429 619,8263525 0,174895 2 199,3443722 65,6 1 21,3 0,917028228 1,090478972 0 VGAATEVELK ICPL:13C(6) (K); ICPL:13C(6) (N-term)

gi|406598 TRUE 1622 1279,627714 640,317495 0,06801 2 172,1891968 86,77 1 24,7 1,74231849 0,573947878 0 TGDEATGVAVVR ICPL (N-term)

gi|406598 TRUE 4920 1637,828199 819,4177375 0,052725 2 438,0980829 107,14 1 48,4 1,793553411 0,557552395 0 FGAPTITNDGVTIAR ICPL (N-term)

gi|406598 TRUE 3623 1679,770002 840,388639 -0,011278 2 323,1846224 45,52 1 7,3 0,575849614 1,736564506 0 GYLSPYFVTDQER ICPL (N-term)

TRUE substrate binding protein

[S. coelicolor A3(2)] gi|21220385 420,3788992 15,57017544 9 7 2,96 0,39 7 0,38 0,05 31873127 226,6 TRUE 5033 1016,519987 508,7636315 0,064337 2 446,1979826 50,14 1 43,7 3,922899692 0,254913477 0 FYVDLVR ICPL (N-term)

gi|21220385 TRUE 3362 1142,526533 571,7669045 0,127791 2 307,5073386 30,63 1 19,8 2,009191567 0,49771262 0 LTADHFTK ICPL (K); ICPL (N-term)

gi|21220385 TRUE 404 1145,474546 573,240911 0,099178 2 57,5211744 53,53 1 32,9 2,048552811 0,488149485 0 ESLTADDGK ICPL (K); ICPL (N-term)

gi|21220385 TRUE 2027 1213,516004 607,26164 0,06932 2 201,6977221 43,17 1 27,8 2,558502643 0,390853612 0 LDSPEWEK ICPL (K); ICPL (N-term)

gi|21220385 TRUE 3577 1254,615323 627,8112995 0,269601 2 320,3285216 78,55 1 22,5 2,040814833 0,490000359 0 IGYAPAPVEK ICPL (K); ICPL (N-term)

gi|21220385 TRUE 4881 1306,674955 653,8411155 0,157369 2 435,0001661 92,06 1 37,6 5,049111446 0,19805465 0 VSQEISAAIAGR ICPL (N-term)

gi|21220385 TRUE 3587 1507,753965 754,3806205 0,081159 2 320,8618634 54,3 1 16,8 3,147153633 0,317747437 0 VNFTVLPENDVR ICPL (N-term)

TRUE hypothetical protein SCO2368

[S. coelicolor A3(2)] gi|21220836 375,6369089 31,41361257 19 8 0,89 0,03 8 1,14 0,04 72759200 741,8 TRUE 3139 725,372907 725,372907 0,044593 1 293,9720384 24,75 1 28,9 0,989856308 1,010247641 0 NAFIR ICPL (N-term)

gi|21220836 TRUE 3005 1054,506467 527,7568715 0,040657 2 285,3747802 54,61 1 27,5 0,761150825 1,313800061 0 QQSFGQVR ICPL (N-term)

gi|21220836 TRUE 2996 1060,526609 530,7669425 0,080315 2 285,292505 41,84 1 21,8 0,761150825 1,313800061 0 QQSFGQVR ICPL:13C(6) (N-term)

gi|21220836 TRUE 3814 1124,537107 1124,537107 0,115493 1 335,4669552 51,74 1 10,1 0,861259387 1,161090392 0 GIAQDFGVNV ICPL (N-term)

gi|21220836 TRUE 3810 1124,537107 562,7721915 0,150217 2 335,178297 33,2 1 62,7 0,861259387 1,161090392 0 GIAQDFGVNV ICPL (N-term)

gi|21220836 TRUE 1680 1361,680768 681,344022 0,072756 2 176,3198549 51,07 1 8,5 0,832484625 1,201223386 0 VVNQAGEAEIAR ICPL (N-term)

gi|21220836 TRUE 1674 1361,680768 681,344022 0,196356 2 175,7016138 95,51 1 61,6 0,832484625 1,201223386 0 VVNQAGEAEIAR ICPL (N-term)

gi|21220836 TRUE 1663 1367,70091 684,354093 0,056214 2 175,2151136 46,1 1 88,2 0,832484625 1,201223386 0 VVNQAGEAEIARnnICPL:13C(6) (N-term)

gi|21220836 TRUE 3847 1493,763441 747,3853585 0,100483 2 337,5048554 55,86 1 99,9 0,911348105 1,097275557 0 INLATVPADIEK ICPL (K); ICPL (N-term)

gi|21220836 TRUE 880 1627,81146 814,409368 0,022464 2 96,99779946 73,11 1 8,3 0,934719554 1,069839607 0 IVFPVSIYDAENR ICPL (N-term)

gi|21220836 TRUE 881 1627,81146 814,409368 0,054664 2 96,84890827 95,69 1 25 0,934719554 1,069839607 0 IVFPVSIYDAENR ICPL (N-term)

gi|21220836 TRUE 5173 1627,81146 814,409368 0,117864 2 456,6961495 71,27 1 135 0,788367055 1,268444684 0 IVFPVSIYDAENR ICPL (N-term)

gi|21220836 TRUE 5174 1627,81146 814,409368 0,117864 2 456,7119325 78,99 1 58,2 0,788367055 1,268444684 0 IVFPVSIYDAENR ICPL (N-term)

gi|21220836 TRUE 5230 1633,831602 817,419439 0,169722 2 460,7841079 44,28 1 16,3 1,006600992 0,993442295 0 IVFPVSIYDAENR ICPL:13C(6) (N-term)

TRUE secreted protein

[S. coelicolor A3(2)] gi|21220348 361,1969089 22,68656716 10 5 0,196 0,009 5 5,17 0,23 47903242 279 TRUE 3555 797,423078 797,423078 0,006722 1 318,9988805 22,67 1 17,4 0,166191746 6,017146017 0 LDLSK ICPL:13C(6) (K); ICPL:13C(6)

(N-term)

gi|21220348 TRUE 3635 1198,610227 599,8087515 0,178297 2 324,0510133 75,77 1 16,1 0,206064931 4,852839311 0 TAAATALELK ICPL (K); ICPL (N-term)

gi|21220348 TRUE 3476 1414,725187 707,8662315 0,100737 2 314,4330634 44,42 1 43 0,191332483 5,226504051 0 VTLTTGGTTDV ICPL:13C(6) (K); ICPL:13C(6) (N-term)

gi|21220348 TRUE 5298 1914,052244 957,52976 -0,01992 2 466,1594327 76,96 1 39,4 0,225603411 4,432557099 0 QPVADANLLGPVTVLGK ICPL:13C(6) (K); ICPL:13C(6) (N-term)

gi|21220348 TRUE 3702 2237,150403 746,3883183 0,155645 3 327,8779557 60,38 1 13 0,188665804 5,300377584 0 VSVNPGDLNVAEVDGTVTLAR ICPL:13C(6) (N-term)

TRUE membrane protein

[S. coelicolor A3(2)] gi|21220739 346,0769089 9,647495362 5 5 9,94 2,78 5 0,14 0,04 37729571 189,1 TRUE 2968 1016,527173 508,7672245 0,084951 2 283,143864 65,12 1 7,3 8,469166678 0,118075371 0 LVSASAAHR ICPL (N-term)

gi|21220739 TRUE 3586 1184,594617 592,8009465 0,176507 2 320,8479552 70,4 1 67,3 10,91341453 0,091630351 0 FEVTLTDVR ICPL (N-term)

gi|21220739 TRUE 1877 1257,589857 629,2985665 0,150267 2 190,8804384 35,16 1 40,9 3,610493698 0,276970432 1 KGDDVPLAK 2 ICPL (K); ICPL (N-term)

gi|21220739 TRUE 2220 1454,716169 727,8617225 0,016955 2 215,7975717 97,6 1 44,3 20,37493861 0,049079902 0 TEGVVLADLEAK ICPL (K); ICPL (N-term)

gi|21220739 TRUE 3787 1512,711774 756,859525 0,03135 2 333,812872 80,32 1 29,3 6,246023111 0,160101873 0 GEIADGTLGWGVK ICPL (K); ICPL

(N-term)

TRUE secreted esterase

[S. coelicolor A3(2)] gi|21221495 336,7969089 10,90651558 11 5 0,46 0,07 5 2,4 0,36 39293116 418,4 TRUE 3101 1163,616727 582,3120015 0,123997 2 291,5620554 41,48 1 32,8 0,258449989 3,869220514 1 TDRDLVLAR ICPL (N-term)

gi|21221495 TRUE 5006 1786,944608 893,975942 -0,018484 2 444,5332247 96,52 1 23,4 0,703997346 1,4204599 0 LDNIAGGNTLTPGAVLR ICPL (N-term)

gi|21221495 TRUE 5004 1792,96475 896,986013 0,058974 2 444,3669325 114,88 1 47,5 0,703997346 1,4204599 0 LDNIAGGNTLTPGAVLR ICPL:13C(6)

(N-term)

gi|21221495 TRUE 1859 1821,807203 911,4072395 -0,024479 2 189,4881557 62,25 1 48,1 0,497609343 2,009608568 0 TDDDAICAGDAGGPLLR ICPL (N-term)

gi|21221495 TRUE 1863 1821,807203 911,4072395 -0,007479 2 189,6210554 81,76 1 23,3 0,497609343 2,009608568 0 TDDDAICAGDAGGPLLR ICPL (N-term)

gi|21221495 TRUE 5168 2185,186323 729,0669583 0,107825 3 456,4076413 37,73 1 83,9 0,449902419 2,222704208 0 TTAVLGPAAGTTVEVVELVPR ICPL

(N-term)

gi|21221495 TRUE 873 2191,206464 731,073672 0,007884 3 96,3400576 32,21 1 19,9 0,399210238 2,504945777 0 TTAVLGPAAGTTVEVVELVPR ICPL:13C(6) (N-term)

TRUE hypothetical protein SCO3767

[S. coelicolor A3(2)] gi|21222178 321,1307266 37,08609272 10 6 0,53 0,07 6 2,07 0,27 40844752 387 TRUE 5325 1180,63606 590,821668 0,094264 2 467,750741 69,14 1 20,6 0,471614013 2,120378046 0 VGILQEIAK ICPL (K); ICPL (N-term)

gi|21222178 TRUE 5342 1180,63606 590,821668 0,139064 2 468,8995746 66,51 1 30,9 0,457612202 2,185256413 0 VGILQEIAK ICPL (K); ICPL (N-term)

gi|21222178 TRUE 5234 1191,587202 596,297239 0,101122 2 461,4717245 79,69 1 12,9 0,366640716 2,72746576 0 LTADFAFGK ICPL:13C(6) (K); ICPL:13C(6) (N-term)

gi|21222178 TRUE 5248 1191,587202 596,297239 0,274522 2 462,1585159 63,38 1 16,3 0,81777675 1,222827624 0 LTADFAFGK ICPL:13C(6) (K); ICPL:13C(6) (N-term)

gi|21222178 TRUE 5322 1192,676328 596,841802 0,124996 2 467,5939741 56,05 1 30,1 0,471614013 2,120378046 0 VGILQEIAK ICPL:13C(6) (K); ICPL:13C(6) (N-term)

gi|21222178 TRUE 5339 1192,676328 596,841802 0,150796 2 468,7636077 67,07 1 148,2 0,457612202 2,185256413 0 VGILQEIAK ICPL:13C(6) (K); ICPL:13C(6) (N-term)

gi|21222178 TRUE 1745 1272,604612 636,805944 0,118512 2 180,6364224 39,38 1 35,7 0,713558701 1,401426398 1 RFEENLDK ICPL:13C(6) (K); ICPL:13C(6) (N-term)

gi|21222178 TRUE 5114 2510,309324 837,441292 0,075924 3 452,0878493 116,66 1 41,1 0,366573748 2,727964033 1 VAQLISTNEVLQNFPADDLRR ICPL:13C(6) (N-term)

TRUE secreted protein

[S. coelicolor A3(2)] gi|21220483 313,9519903 7,370184255 7 4 2,33 0,23 4 0,45 0,04 40648753 266,9 TRUE 3841 979,524717 490,2659965 0,039007 2 337,0199717 52,58 1 41,1 2,378365915 0,420456749 0 GLLELPK ICPL (K); ICPL (N-term)

gi|21220483 TRUE 3840 991,564985 496,2861305 0,013739 2 337,0078469 52,68 1 42,9 2,378365915 0,420456749 0 GLLELPK ICPL:13C(6) (K); ICPL:13C(6) (N-term)

gi|21220483 TRUE 3582 1102,520383 551,7638295 0,141941 2 320,5688298 64,07 1 37,9 3,014942797 0,331681251 0 TVPGLDYK ICPL (K); ICPL (N-term)

gi|21220483 TRUE 3596 1604,759077 802,8831765 0,067247 2 321,8172133 91,51 1 34,1 1,754726019 0,569889538 0 LYADDAEGTYILR ICPL (N-term)

gi|21220483 TRUE 3599 1604,759077 802,8831765 0,086247 2 321,8232389 34,37 1 23,6 1,754726019 0,569889538 0 LYADDAEGTYILR ICPL (N-term)

gi|21220483 TRUE 3913 1868,833768 934,920522 -0,023044 2 342,1182384 110,13 1 61,2 2,157511087 0,463497039 0 FTVLGDTDATFTGGEK ICPL (K); ICPL

(N-term)

gi|21220483 TRUE 3914 1880,874036 940,940656 -0,008512 2 342,13208 113,69 1 26,1 2,157511087 0,463497039 0 FTVLGDTDATFTGGEK ICPL:13C(6) (K); ICPL:13C(6) (N-term)

TRUE co-chaperonin GroES

[S. coelicolor A3(2)] gi|21223140 312,25 74,50980392 9 6 0,39 0,04 6 2,73 0,27 38935452 334,4 TRUE 3970 1096,567319 548,7872975 0,101405 2 345,7677637 53,73 1 39,4 0,26848074 3,724661959 0 DVLAIVEK ICPL (K); ICPL (N-term)

gi|21223140 TRUE 3966 1108,607587 1108,607587 -0,037187 1 345,5815968 56,16 1 63,5 0,26848074 3,724661959 0 DVLAIVEK ICPL:13C(6) (K); ICPL:13C(6) (N-term)

gi|21223140 TRUE 3426 1262,693036 631,850156 0,109488 2 311,5304384 38,74 1 20,3 0,560931811 1,782747887 0 VAIKPLEDR ICPL:13C(6) (K); ICPL:13C(6) (N-term)

gi|21223140 TRUE 3380 1757,937208 879,472242 0,100316 2 309,061905 65,59 1 28,5 0,315417313 3,170403019 0 EKPQEGVVLAVGPGR ICPL:13C(6) (K); ICPL:13C(6) (N-term)

gi|21223140 TRUE 3388 1757,937208 586,6505867 0,14344 3 309,2531717 35,68 1 31,9 0,315417313 3,170403019 0 EKPQEGVVLAVGPGR ICPL:13C(6) (K); ICPL:13C(6) (N-term)

gi|21223140 TRUE 963 2544,280805 848,765119 -0,134757 3 102,2865499 67,46 1 15,3 0,374555349 2,66983238 1 FEDGNRLPLDVSVGDVVLYSK ICPL:13C(6) (K); ICPL:13C(6) (N-term)

gi|21223140 TRUE 5250 2544,280805 848,765119 -0,044757 3 462,5116327 74,61 1 43,9 0,361088214 2,769406371 1 FEDGNRLPLDVSVGDVVLYSK ICPL:13C(6) (K); ICPL:13C(6) (N-term)

gi|21223140 TRUE 3975 2588,36453 863,459694 0,054618 3 346,1366133 77,15 1 64,1 0,442575283 2,259502593 0 IVVQPLDAEQTTASGLVIPDTAK ICPL:13C(6) (K); ICPL:13C(6) (N-term)

TRUE secreted protein

[S. coelicolor A3(2)] gi|21221339 302,1338177 13,87665198 7 5 1,44 0,06 5 0,7 0,03 5298632 188,9 TRUE 4699 913,452634 457,229955 0,06349 2 421,4610413 39,33 1 50,2 1,447166453 0,691005515 0 LTFGGSAR ICPL (N-term)

gi|21221339 TRUE 270 1121,522138 561,264707 0,172186 2 46,07210827 57,5 1 40,5 1,604322523 0,623316064 0 QEVNEEIR ICPL (N-term)

gi|21221339 TRUE 1566 1236,558683 618,7829795 0,077441 2 168,2567221 57,41 1 6,5 1,251774037 0,798866225 0 QTSYLADGDR ICPL:13C(6) (N-term)

gi|21221339 TRUE 2946 1359,676404 680,34184 0,08772 2 281,6937386 46,17 1 27,6 1,580036548 0,632896752 0 NVVHTSVGGTGAR ICPL (N-term)

gi|21221339 TRUE 3981 1394,62634 697,816808 0,167784 2 346,5836298 85,54 1 39,7 1,330724061 0,751470593 0 VFDTVVDFDK ICPL (K); ICPL (N-term)

TRUE BldKB

[S. coelicolor] gi|1532202 296,2694679 8,637873754 4 4 1,56 0,24 4 0,7 0,11 13093334 136,6 TRUE 6432 1498,780134 749,893705 0,07479 2 576,9821632 95,69 1 63,3 2,038110102 0,490650627 0 VFQIFGSNVGGIR ICPL (N-term)

gi|1532202 TRUE 3634 1528,727796 764,867536 0,083128 2 323,9151802 80,03 1 18,3 1,382044113 0,723565905 0 IQQITDTAEATK ICPL (K); ICPL (N-term)

gi|1532202 TRUE 5336 1686,837354 843,922315 0,02537 2 468,4784157 69,43 1 31,5 1,84937815 0,540722296 0 DAITLALPSDSVFK ICPL (K); ICPL (N-term)

gi|1532202 TRUE 1914 1702,770734 851,889005 -0,03201 2 193,4908635 63,5 1 23,5 0,934547403 1,07003668 0 HLPDNVLETPDDK ICPL (K); ICPL (N-term)

TRUE ribosome recycling factor

[S. coelicolor A3(2)] gi|21223982 255,2884727 19,45945946 9 5 0,45 0,11 5 2,97 0,76 21096732 241,2 TRUE 2153 907,440702 907,440702 -0,033802 1 211,404505 37,11 1 18,8 0,308866678 3,237642874 0 EAELLEV ICPL (N-term)

gi|21223982 TRUE 2166 913,460844 913,460844 -0,020344 1 212,6023557 31,93 1 22,8 0,748288846 1,336382342 0 EAELLEV ICPL:13C(6) (N-term)

gi|21223982 TRUE 2149 913,460844 913,460844 0,011356 1 211,0487307 32,21 1 46 0,308866678 3,237642874 0 EAELLEV ICPL:13C(6) (N-term)

gi|21223982 TRUE 1759 932,456769 466,7320225 0,043155 2 181,6037221 54,81 1 4,1 0,255797479 3,909342674 0 EDFAAIR ICPL:13C(6) (N-term)

gi|21223982 TRUE 1907 1289,656309 645,3317925 0,105215 2 193,0070971 38,9 1 21,4 0,716341949 1,395981349 1 HKEAELLEV ICPL:13C(6) (K); ICPL:13C(6) (N-term)

gi|21223982 TRUE 3549 1331,657331 666,3323035 0,161993 2 318,5844469 46,55 1 38,5 0,207180189 4,826716333 0 VTFPELTEER ICPL:13C(6) (N-term)

TRUE glycine betaine transport system permease protein

[S. coelicolor A3(2)] gi|21220116 254,8 4,47761194 4 4 4,09 0,23 4 0,25 0,01 19062626 97,7 TRUE 1990 991,415586 496,211431 0,084938 2 198,9181637 35,82 1 5 4,634295213 0,215782542 0 SDLFDGK ICPL (K); ICPL (N-term)

gi|21220116 TRUE 3757 1256,579328 628,793302 0,111796 2 331,7956634 55,84 1 24,9 3,85871838 0,259153403 0 DINSLEDLK ICPL (K); ICPL (N-term)

gi|21220116 TRUE 3718 1455,711393 728,3593345 0,158931 2 329,1152053 82,19 1 40,7 3,488108002 0,286688371 0 QLTSLEAEINK ICPL (K); ICPL (N-term)

gi|21220116 TRUE 3752 1538,727415 769,8673455 0,028709 2 331,512272 80,95 1 27,1 4,377307943 0,228450914 0 TWLEANPGVVDK ICPL (K); ICPL (N-term)

TRUE metallopeptidase

[S. coelicolor A3(2)] gi|21225030 247,85 8,102766798 4 4 3,04 0,74 4 0,41 0,1 4196424 106,9 TRUE 4757 1016,588743 508,7980095 0,075781 2 426,1776749 61,16 1 40,6 3,589555256 0,278586044 0 GAPLTVVVR ICPL (N-term)

gi|21225030 TRUE 4882 1031,599622 516,303449 0,042502 2 435,2356823 61,32 1 11 2,365991572 0,422655774 0 GALAVQVLR ICPL (N-term)

gi|21225030 TRUE 4870 1149,626218 575,316747 0,069906 2 434,2154407 70,74 1 44 4,717335593 0,21198407 0 LEGTATILAR ICPL (N-term)

gi|21225030 TRUE 4038 1632,809553 816,9084145 0,171371 2 349,8839968 54,63 1 11,3 1,391816003 0,718485776 0 NAIGDDAFFTVLK ICPL:13C(6) (K); ICPL:13C(6) (N-term)

TRUE hypothetical protein SCO3324

[S. coelicolor A3(2)] gi|21221755 242,9509952 9,307875895 5 4 0,15 0,01 4 7,16 0,71 10259622 111 TRUE 3258 943,472761 472,2400185 0,127763 2 301,0657136 33,03 1 19,4 0,159512392 6,269105416 0 ETWGALR ICPL:13C(6) (N-term)

gi|21221755 TRUE 3395 1120,572858 560,790067 0,125866 2 309,6689888 63,58 1 43,6 0,168782878 5,924771572 0 AYLDLASTR ICPL:13C(6) (N-term)

gi|21221755 TRUE 1752 1170,548109 585,7776925 0,124415 2 181,1269221 75,81 1 13,2 0,1495774 6,685501945 0 LGLADGNDER ICPL:13C(6) (N-term)

gi|21221755 TRUE 1656 1506,727872 753,867574 0,052652 2 174,4006139 74,53 1 33,6 0,102038792 9,800194405 0 SGPLDHESLGEVR ICPL:13C(6) (N-term)

gi|21221755 TRUE 1655 1506,727872 502,9141413 0,133376 3 174,2851552 49 1 1,2 0,102038792 9,800194405 0 SGPLDHESLGEVR ICPL:13C(6) (N-term)

TRUE cytochrome c oxidase subunit II

[S. coelicolor A3(2)] gi|21220633 241,7438177 15,04702194 13 6 3,89 0,49 6 0,28 0,04 32347892 422,4 TRUE 4791 827,441007 827,441007 0,022193 1 428,7695991 27,29 1 34,3 5,598541179 0,178617959 0 FVLTSR ICPL (N-term)

gi|21220633 TRUE 4789 827,441007 414,2241415 0,079317 2 428,7554077 37,06 1 50,8 5,598541179 0,178617959 0 FVLTSR ICPL (N-term)

gi|21220633 TRUE 3512 961,441404 961,441404 0,011196 1 316,5939973 31,78 1 19,3 3,294725118 0,303515457 0 EGTFLGK ICPL (K); ICPL (N-term)

gi|21220633 TRUE 3507 961,441404 481,22434 0,07972 2 316,2924304 36,99 1 30,5 3,294725118 0,303515457 0 EGTFLGK ICPL (K); ICPL (N-term)

gi|21220633 TRUE 1736 975,489393 488,2483345 0,056531 2 180,1384469 45,28 1 91,8 4,601776662 0,217307374 0 ELAGIPDR ICPL (N-term)

gi|21220633 TRUE 5013 977,454938 489,231107 0,064986 2 445,0031911 31,36 1 39 4,388706882 0,227857551 0 MLFNVK ICPL (K); ICPL (N-term); Oxidation (M)

gi|21220633 TRUE 1561 1408,57324 704,790258 0,041684 2 167,9013301 58,43 1 3,9 3,187096136 0,313765245 0 CAELCGVDHSR ICPL (N-term)

gi|21220633 TRUE 3329 1454,669874 727,838575 0,11385 2 305,5291386 31,62 1 45,2 2,24019482 0,446389748 0 YEQHLQDLAK ICPL (K); ICPL (N-term)

gi|21220633 TRUE 3332 1466,710142 733,858709 0,088582 2 305,8839136 27,78 1 26 2,24019482 0,446389748 0 YEQHLQDLAK ICPL:13C(6) (K); ICPL:13C(6) (N-term)

TRUE type II citrate synthase

[S. coelicolor A3(2)] gi|21221189 240,1309952 10,25641026 6 4 2,05 0,29 4 0,53 0,07 28482205 205,3 TRUE 3252 1169,594938 585,301107 0,127786 2 300,7031973 31,76 1 2,6 2,872338018 0,34814844 0 QIYTGVVER ICPL (N-term)

gi|21221189 TRUE 1818 1256,554212 628,780744 -0,025288 2 186,5099808 60,2 1 51,2 1,906544628 0,524509096 0 DAGGDVDSFIR ICPL (N-term)

gi|21221189 TRUE 3987 1326,657575 663,8324255 0,201749 2 347,0143888 47,67 1 74,6 2,083250033 0,480019193 0 SDELLDIALK ICPL (K); ICPL (N-term)

gi|21221189 TRUE 3988 1338,697843 669,8525595 0,113481 2 347,0298053 36,49 1 33,9 2,083250033 0,480019193 0 SDELLDIALK ICPL:13C(6) (K); ICPL:13C(6) (N-term)

gi|21221189 TRUE 3706 1583,806364 792,40682 0,05956 2 328,3988133 25,44 1 16,1 1,319145904 0,758066259 0 SAITYLDGEAGILR ICPL (N-term)

gi|21221189 TRUE 3703 1583,806364 792,40682 0,08876 2 327,9638805 104,5 1 26,9 1,319145904 0,758066259 0 SAITYLDGEAGILR ICPL (N-term)

TRUE 30S ribosomal protein S1

[S. coelicolor A3(2)] gi|21220480 237,3609952 8,366533865 5 4 0,53 0,07 4 2,02 0,27 18297283 584,2 TRUE 3564 1077,557477 539,2823765 0,106647 2 319,5407056 41,7 1 17,2 0,458983622 2,178726979 0 VIDIDLER ICPL (N-term)

gi|21220480 TRUE 3558 1083,577619 542,2924475 0,130505 2 319,2413386 59,84 1 40 0,458983622 2,178726979 0 VIDIDLER ICPL:13C(6) (N-term)

gi|21220480 TRUE 6448 1110,609479 555,8083775 0,068245 2 578,3820886 54,55 1 479,7 0,586219044 1,705847004 0 LVPFGAFVR ICPL (N-term)

gi|21220480 TRUE 3242 1385,736341 693,3718085 0,039183 2 300,1675637 36,97 1 29,7 0,355539598 2,812626236 0 THQIGQVVPGK ICPL:13C(6) (K); ICPL:13C(6) (N-term)

gi|21220480 TRUE 3941 1763,827543 882,4174095 -0,000619 2 343,9203637 90 1 17,6 0,727079487 1,375365443 0 YFNDGDIVDGVIVK ICPL (K); ICPL

(N-term)

TRUE ORF3

[S. coelicolor A3(2)] gi|565055 221,69 21,4953271 3 3 0,19 0,02 3 5,54 0,57 19754800 103,4 TRUE 3397 1244,636564 622,82192 0,16216 2 309,8195888 59,2 1 33,3 0,220512485 4,534890613 0 GFDVSVPASVR ICPL:13C(6) (N-term)

gi|565055 TRUE 1740 1344,648588 672,827932 0,181136 2 180,323464 102,9 1 25,1 0,193557129 5,16643331 0 LTGGSGLGDSDVR ICPL:13C(6) (N-term)

gi|565055 TRUE 5258 2623,240644 875,0850653 -0,053596 3 463,0488327 59,59 1 45 0,144597812 6,915733971 0 ATYDPDPITAFAEYNQIVNDVR ICPL:13C(6) (N-term)

TRUE fructose-bisphosphate aldolase

[S. coelicolor A3(2)] gi|21222064 219,06 13,11953353 4 4 0,95 0,12 4 1,11 0,14 14416890 99,6 TRUE 3041 1145,55203 573,279653 0,138894 2 287,9447216 45,46 1 5,6 1,16483797 0,858488499 0 VVEACGHLR ICPL (N-term)

gi|21222064 TRUE 1836 1182,582807 591,7950415 0,193117 2 187,9147386 49,85 1 35,3 0,786481889 1,271485096 0 ELNEGIASK ICPL:13C(6) (K); ICPL:13C(6) (N-term)

gi|21222064 TRUE 3659 1226,605161 613,8062185 0,169163 2 325,549872 77,95 1 46,5 1,187256763 0,842277788 0 TVEALGLGEK ICPL (K); ICPL (N-term)

gi|21222064 TRUE 5014 2170,09271 724,035754 0,317838 3 444,9877079 45,8 1 12,2 0,673242213 1,485349523 1 DKLDGYVRPLIAVSEER ICPL (K); ICPL

(N-term)

TRUE trigger factor

[S. coelicolor A3(2)] gi|21221077 215,7188992 11,96581197 5 4 1,8 0,85 4 1,05 0,49 11595509 150,9 TRUE 373 1317,559324 659,2833 0,0778 2 55,031816 65,23 1 47,6 1,809597656 0,552609027 0 TAEEFETETR ICPL (N-term)

gi|21221077 TRUE 4046 1401,704893 701,3560845 0,181231 2 350,4174805 49,28 1 17,1 3,523926474 0,283774366 0 TQFVLDELVK ICPL (K); ICPL (N-term)

gi|21221077 TRUE 3960 1635,801282 818,404279 0,077842 2 345,2232138 47,27 1 25,1 1,173599745 0,852079258 0 GAVLEEAVNDALPK ICPL (K); ICPL

(N-term)

gi|21221077 TRUE 3802 1722,873594 861,940435 0,04153 2 334,7719221 37,5 1 11,5 0,393742666 2,539729843 0 AAADGDVLTLDLQAK ICPL:13C(6) (K); ICPL:13C(6) (N-term)

TRUE secreted esterase

[S. coelicolor A3(2)] gi|21224890 209,95 3,80952381 8 5 0,6 0,15 5 2,16 0,53 19910502 461 TRUE 3391 922,545209 461,7762425 0,008515 2 309,3659466 34,63 1 18,2 0,875547749 1,142142163 0 VVEIVPR ICPL:13C(6) (N-term)

gi|21224890 TRUE 4855 922,545209 461,7762425 0,022915 2 433,0013826 40,64 1 28,7 0,278811248 3,586655879 0 VVEIVPR ICPL:13C(6) (N-term)

gi|21224890 TRUE 4614 1072,626172 536,816724 0,085152 2 415,0144823 31,14 1 101,8 0,91567609 1,092089234 1 RVVEIVPR ICPL (N-term)

gi|21224890 TRUE 4615 1072,626172 536,816724 0,120952 2 415,0968909 46,11 1 121,3 0,91567609 1,092089234 1 RVVEIVPR ICPL (N-term)

gi|21224890 TRUE 1543 1149,601087 575,3041815 0,147437 2 166,7102384 32,03 1 23,3 0,296919058 3,367921236 1 TDRDVVLAR ICPL (N-term)

gi|21224890 TRUE 1539 1155,621229 578,3142525 0,042095 2 166,363464 39,04 1 87,9 0,296919058 3,367921236 1 TDRDVVLAR ICPL:13C(6) (N-term)

gi|21224890 TRUE 1544 1155,621229 385,8785937 0,078319 3 166,6341803 30,61 1 21,2 0,296919058 3,367921236 1 TDRDVVLAR ICPL:13C(6) (N-term)

gi|21224890 TRUE 4041 1512,704404 756,85584 0,11932 2 350,137705 84,16 1 58,6 0,623507669 1,603829511 0 VDDLASWIDEK ICPL:13C(6) (K); ICPL:13C(6) (N-term)

TRUE superoxide dismutase

[S. coelicolor A3(2)] gi|21219516 203,39 20,46511628 4 3 1,48 0,12 3 0,69 0,05 21608956 176,7 TRUE 3436 1337,684797 669,3460365 0,127527 2 312,0422138 30,35 1 36,9 1,34459685 0,743717345 1 ERGDSLLLKP ICPL (K); ICPL (N-term)

gi|21219516 TRUE 3432 1349,725065 675,3661705 0,125059 2 312,0863472 26,96 1 33,5 1,34459685 0,743717345 1 ERGDSLLLKP ICPL:13C(6) (K); ICPL:13C(6) (N-term)

gi|21219516 TRUE 1854 1492,702619 746,8549475 0,055705 2 189,4980133 100,51 1 86,8 1,779055831 0,562095907 0 GANDTLEQLAEAR ICPL (N-term)

gi|21219516 TRUE 5011 2197,067243 733,027265 0,123705 3 444,8072077 72,53 1 19,5 1,327916763 0,753059249 0 AAATTQGSGWGVLAYEPLSGR

ICPL (N-term)

TRUE superoxide dismutase [Fe-Zn]

[S. coelicolor A3(2)] gi|21221090 199,6369089 19,71830986 3 3 1,73 0,18 3 0,6 0,06 15410359 136,9 TRUE 3734 947,519636 947,519636 -0,001336 1 330,3679306 29,12 1 30,6 2,068766146 0,483379913 0 TNTLLLAP ICPL (N-term)

gi|21221090 TRUE 1854 1492,702619 746,8549475 0,055705 2 189,4980133 100,51 1 86,8 1,779055831 0,562095907 0 GANDTLEQLAEAR ICPL (N-term)

gi|21221090 TRUE 5011 2197,067243 733,027265 0,123705 3 444,8072077 72,53 1 19,5 1,327916763 0,753059249 0 AAATTQGSGWGVLAYEPLSGR ICPL

(N-term)

TRUE aminopeptidase N

[S. coelicolor A3(2)] gi|21221100 198,967904 4,317386231 5 3 0,99 0,19 3 1,13 0,22 15970922 183,6 TRUE 6376 1111,662214 556,334745 0,14011 2 572,9652219 45,7 1 51,6 1,019992451 0,980399413 0 IALPGLLPGR ICPL (N-term)

gi|21221100 TRUE 6374 1111,662214 556,334745 0,14351 2 572,8746134 37,48 1 80,5 1,019992451 0,980399413 0 IALPGLLPGR ICPL (N-term)

gi|21221100 TRUE 3829 1473,700849 737,3540625 0,161875 2 336,3204554 100,93 1 18 0,604469652 1,654342773 0 IAIGAYDLDGAGK ICPL (K); ICPL (N-term)

gi|21221100 TRUE 3824 1485,741117 743,3741965 0,152407 2 336,1470218 108,43 1 22,9 0,604469652 1,654342773 0 IAIGAYDLDGAGK ICPL:13C(6) (K); ICPL:13C(6) (N-term)

gi|21221100 TRUE 5349 1571,800687 786,4039815 0,121437 2 469,6700663 51,36 1 10,6 1,340805899 0,745820107 0 LSDLLGALEETSGR ICPL:13C(6) (N-term)

TRUE Clp-family ATP-binding protease

[S. coelicolor A3(2)] gi|21221802 197,7176355 4,637336504 3 2 1,33 0,3 2 0,83 0,19 9813707 109,8 TRUE 1977 1264,616773 632,8120245 0,155951 2 198,1455973 61,29 1 24 1,73109021 0,577670646 0 AIDLIDEAGSR ICPL (N-term)

gi|21221802 TRUE 6591 1671,84892 836,428098 -0,048596 2 588,7870555 125,32 1 27,6 0,922264711 1,084287394 0 QQVIQLLSGYQGK ICPL (K); ICPL (N-term)

TRUE hypothetical protein SCO2271

[S. coelicolor A3(2)] gi|21220740 197,32 8,385744235 3 3 4,15 0,45 3 0,25 0,03 18210814 89,6 TRUE 3856 941,451566 471,229421 0,029358 2 338,1931386 34,26 1 31,6 3,167151854 0,315741097 0 LDWGIK ICPL (K); ICPL (N-term)

gi|21220740 TRUE 4966 1594,753644 797,88046 -0,06332 2 441,2917911 82,39 1 39 4,923312396 0,203115285 0 SSFQTYVTGPVAK ICPL (K); ICPL (N-term)

gi|21220740 TRUE 3585 2046,025052 682,679868 0,052596 3 320,7245301 80,67 1 19 4,343592903 0,230224154 0 GGGGTVALDNLPATLTAEGAR ICPL

(N-term)

TRUE DNA-directed RNA polymerase subunit alpha

[S. coelicolor A3(2)] gi|21223108 195,8669089 14,11764706 5 3 1,08 0,23 3 1,04 0,22 14578944 169,5 TRUE 6581 1223,678266 612,342771 0,183458 2 588,1020806 62,63 1 84,7 1,390323434 0,719257099 0 TLVELFGLAR ICPL (N-term)

gi|21223108 TRUE 5378 1494,787733 747,8975045 0,037591 2 471,4017575 41,4 1 11,2 0,625794083 1,597969728 0 EDVTDLILNIK ICPL:13C(6) (K); ICPL:13C(6) (N-term)

gi|21223108 TRUE 6343 1590,884962 795,946119 0,037362 2 570,4913632 36,53 1 54,2 1,217603548 0,821285386 0 TLLSSIPGAAVTSIR ICPL (N-term)

TRUE hypothetical protein SCO3967

[S. coelicolor A3(2)] gi|21222371 191,64 22,13438735 5 4 1,61 0,15 4 0,64 0,06 11567001 74 TRUE 3669 1165,531293 583,2692845 0,111031 2 325,939097 54,81 1 26,2 1,2407445 0,805967707 0 GGYAVVDFK ICPL (K); ICPL (N-term)

gi|21222371 TRUE 3671 1177,571561 589,2894185 0,077163 2 326,0424885 42,73 1 14,7 1,2407445 0,805967707 0 GGYAVVDFK ICPL:13C(6) (K); ICPL:13C(6) (N-term)

gi|21222371 TRUE 3503 1234,552747 617,7800115 0,271577 2 315,986872 28,8 1 6,9 1,436550856 0,696111799 0 GVEPGYFQK ICPL (K); ICPL (N-term)

gi|21222371 TRUE 5400 2156,069883 1078,53858 -0,213359 2 472,2666327 83,12 1 9 1,961046286 0,50993187 0 FPVSIGALPEDADQLVFK ICPL (K); ICPL (N-term)

gi|21222371 TRUE 3921 2486,187406 829,4006527 0,146242 3 342,7873888 24,91 1 17,2 1,785658437 0,560017515 0 WIEVPQEGQEEPETPAPVLK ICPL (K); ICPL (N-term)

TRUE alpha-ketoglutarate decarboxylase

[S. coelicolor A3(2)] gi|21223647 184,4586307 4,402515723 5 3 2,16 0,32 3 0,49 0,07 12627413 93,6 TRUE 4788 989,483915 495,2455955 0,071809 2 428,5676914 40,72 1 17 2,454942967 0,407341439 0 SYAQIFR ICPL (N-term)

gi|21223647 TRUE 6494 1220,674573 610,8409245 0,101351 2 582,3295968 31,89 2 13,3 1,465684888 0,682274893 1 RGLTDTAIIR ICPL (N-term)

gi|21223647 TRUE 5035 2393,245939 798,4201637 0,121909 3 446,3549661 80,56 1 17,7 2,546171418 0,392746534 0 VSLVANPSHLEAVDPVLEGVSR ICPL

(N-term)

TRUE secreted protein

[S. coelicolor A3(2)] gi|21222858 183,11 8,333333333 4 3 4,14 0,9 3 0,28 0,06 2590118 115,9 TRUE 3057 879,468275 440,2377755 0,003449 2 288,7135056 42,97 1 15,3 5,892774693 0,169699344 0 ATLTNVR ICPL (N-term)

gi|21222858 TRUE 1668 1190,558866 595,783071 0,122458 2 175,2690304 29,46 1 19,4 2,426865872 0,412054087 0 LYDPPNNPR ICPL (N-term)

gi|21222858 TRUE 1943 1728,786375 864,8968255 0,010149 2 195,6781552 86,42 1 34,2 4,079678262 0,245117369 0 GASDIGYLTDEHIK ICPL (K); ICPL (N-term)

TRUE Rieske iron-sulfur protein

[S. coelicolor A3(2)] gi|21220626 179,74 7,932011331 11 3 1,14 0,44 3 1,27 0,49 25493982 431,7 TRUE 4801 908,502195 454,7547355 -0,004071 2 429,7532663 57,17 1 25,4 0,4006982 2,495643857 0 AALMIIR ICPL (N-term); Oxidation (M)

gi|21220626 TRUE 4803 908,502195 454,7547355 0,042529 2 429,8954413 29,78 1 87,5 0,4006982 2,495643857 0 AALMIIR ICPL (N-term); Oxidation (M)

gi|21220626 TRUE 1945 1011,478162 506,242719 0,054562 2 195,8077221 36,2 1 38 1,477753633 0,676702786 0 LEPDSIK ICPL (K); ICPL (N-term)

gi|21220626 TRUE 1944 1023,51843 512,262853 0,106294 2 195,6446971 39 1 62,4 1,477753633 0,676702786 0 LEPDSIK ICPL:13C(6) (K); ICPL:13C(6) (N-term)

gi|21220626 TRUE 6196 1580,869596 527,6280493 0,088352 3 559,8596304 35,72 1 13,1 1,424301498 0,702098539 0 VIFGPAGHALPQLR ICPL (N-term)

gi|21220626 TRUE 6194 1580,869596 790,938436 0,124528 2 559,5679888 45,14 1 76,6 1,424301498 0,702098539 0 VIFGPAGHALPQLR ICPL (N-term)

TRUE triosephosphate isomerase

[S. coelicolor A3(2)] gi|21220430 178,9169089 9,302325581 4 3 0,68 0,08 3 1,54 0,18 13798053 1 22,5 TRUE 1984 1283,630536 642,318906 0,147588 2 198,7578304 38,34 1 30,2 0,504414578 1,982496233 0 SVQTLVDGDK ICPL:13C(6) (K); ICPL:13C(6) (N-term)

gi|21220430 TRUE 3931 1589,748167 795,3777215 0,118957 2 343,4785802 32,06 1 32,7 0,709461485 1,409519785 0 LAELYSQELADK ICPL (K); ICPL (N-term)

gi|21220430 TRUE 3937 1601,788434 801,397855 -0,09211 2 343,5321802 74,23 1 35,2 0,709461485 1,409519785 0 LAELYSQELADK ICPL:13C(6) (K); ICPL:13C(6) (N-term)

gi|21220430 TRUE 3716 1635,851148 818,429212 0,047576 2 328,8253381 68,87 1 24,4 0,820640868 1,218559834 1 SVQTLVDGDKLK 2 ICPL:13C(6) (K); ICPL:13C(6) (N-term)

TRUE 50S ribosomal protein L1

[S. coelicolor A3(2)] gi|21223031 175,6238177 10,37344398 2 2 1,99 0,28 2 0,52 0,07 2863273 27,4 TRUE 2170 1274,626279 637,8167775 0,058245 2 212,5421301 82,44 1 21,7 2,370131123 0,421917585 0 NLLVEEDPAAV ICPL (N-term)

gi|21223031 TRUE 5321 1694,874753 847,9410145 0,062571 2 467,4295826 98,23 1 5,7 1,603318511 0,62370639 0 LVENYGAALEEILR ICPL (N-term)

TRUE hypothetical protein SCO4584

[S. coelicolor A3(2)] gi|21222967 174,6 6,904761905 4 3 2,41 0,44 3 0,45 0,08 7457544 128,4 TRUE 1960 1117,494886 559,251081 0,134838 2 196,8107056 36,21 1 34 1,504146645 0,664828794 0 DFADQALK ICPL (K); ICPL (N-term)

gi|21222967 TRUE 1959 1129,535154 565,271215 0,08997 2 196,8653387 54,05 1 30,7 1,504146645 0,664828794 0 DFADQALK ICPL:13C(6) (K); ICPL:13C(6) (N-term)

gi|21222967 TRUE 3680 1240,620801 620,8140385 0,182323 2 326,5998885 57 1 24,2 2,67510788 0,3738167 0 VTAEQLIEK ICPL (K); ICPL (N-term)

gi|21222967 TRUE 4836 1417,743391 709,3753335 0,087333 2 431,7789325 63,55 1 39,5 3,035059425 0,32948284 0 LTELVSGSHTLR ICPL (N-term)

TRUE lipoprotein

[S. coelicolor A3(2)] gi|21223259 171,457904 14,94252874 4 3 2,55 0,67 3 0,47 0,13 7655301 84,3 TRUE 3605 969,50398 485,255628 0,054544 2 321,8884469 32,49 1 18,4 1,565932534 0,638597116 0 IISGEIK ICPL (K); ICPL (N-term)

gi|21223259 TRUE 2180 1501,695753 751,3515145 0,052571 2 213,5429722 52,26 1 10,5 4,25183836 0,235192384 0 FADDAELQAAIK ICPL (K); ICPL (N-term)

gi|21223259 TRUE 5373 2490,19354 830,7360307 0,284608 3 471,0844914 72,52 1 10,6 1,868174662 0,535281856 0 NVADLVFNEQEASYLAGVAAAK ICPL (K); ICPL (N-term)

TRUE transcriptional regulator

[S. coelicolor A3(2)] gi|21221840 169,8409952 7,90513834 6 2 0,42 0,02 2 2,37 0,13 31261433 339,4 TRUE 3402 1047,558134 524,282705 0,05859 2 310,1483637 72,28 1 88,7 0,455448161 2,195639558 0 DAILANAVR ICPL (N-term)

gi|21221840 TRUE 3403 1053,578275 1053,578275 -0,045475 1 310,18268 38,04 1 24,4 0,455448161 2,195639558 0 DAILANAVR ICPL:13C(6) (N-term)

gi|21221840 TRUE 3401 1053,578275 527,2927755 0,046649 2 309,93328 62 1 61,7 0,455448161 2,195639558 0 DAILANAVR ICPL:13C(6) (N-term)

gi|21221840 TRUE 1781 1294,590955 647,7991155 0,129969 2 183,2675472 81,04 1 40,7 0,391527502 2,554098994 0 IQDEADELTR ICPL (N-term)

gi|21221840 TRUE 1778 1300,611097 650,8091865 0,107427 2 183,1496053 73,58 1 88,7 0,391527502 2,554098994 0 IQDEADELTR ICPL:13C(6) (N-term)

TRUE secreted protein

[S. coelicolor A3(2)] gi|21223053 169,2569089 7,300509338 6 4 1,62 0,21 4 0,66 0,09 21643123 181,4 TRUE 3358 1072,57855 536,792913 0,060774 2 307,3495301 28,78 1 20,4 1,560247062 0,640924136 0 SVLLPGPER ICPL (N-term)

gi|21223053 TRUE 3361 1375,685224 688,34625 0,1161 2 307,9187968 27,76 1 29,1 1,105923715 0,9042215 0 TLPDTSLPVGDR ICPL (N-term)

gi|21223053 TRUE 3383 1375,685224 688,34625 0,2219 2 308,9754549 26,77 1 23,9 2,285633935 0,43751538 0 TLPDTSLPVGDR ICPL (N-term)

gi|21223053 TRUE 1534 1476,617216 738,812246 0,012108 2 165,8632389 49,61 1 16,9 1,528737499 0,65413454 0 QTGNLDCSGYTR ICPL (N-term)

TRUE secreted protein

[S. coelicolor A3(2)] gi|21224891 168,74 16,07142857 6 4 0,99 0,04 4 1,01 0,04 17893998 190,8 TRUE 208 980,443204 490,72524 0,11612 2 40,36375786 41,51 1 12,2 0,961294823 1,040263586 0 GVGESVDGR ICPL (N-term)

gi|21224891 TRUE 3222 1099,589445 550,2983605 0,040279 2 299,1468805 40,3 1 33,6 0,87364235 1,144633156 0 EHVLVEIR ICPL (N-term)

gi|21224891 TRUE 3225 1099,589445 550,2983605 0,192679 2 299,1409386 47,04 1 28,8 0,87364235 1,144633156 0 EHVLVEIR ICPL (N-term)

gi|21224891 TRUE 5225 1350,741575 675,8744255 0,188549 2 460,6652909 61,76 1 23,3 1,032524502 0,968500019 0 ILAEQNIVLK ICPL (K); ICPL (N-term)

gi|21224891 TRUE 5226 1362,781843 681,8945595 0,135481 2 460,6799575 69,14 1 34,6 1,032524502 0,968500019 0 ILAEQNIVLK ICPL:13C(6) (K); ICPL:13C(6) (N-term)

gi|21224891 TRUE 5212 1362,781843 681,8945595 0,176081 2 459,5998242 80,19 1 58,3 1,10779031 0,902697912 0 ILAEQNIVLK ICPL:13C(6) (K); ICPL:13C(6) (N-term)

TRUE carboxyl transferase

[S. coelicolor A3(2)] gi|21223890 166,93 6,451612903 4 3 5,55 0,27 3 0,181 0,009 5643630 161,6 TRUE 6359 819,508665 410,2579705 0,029059 2 571,6705472 34,5 1 46,4 5,689868887 0,175750974 0 ISLILR ICPL (N-term)

gi|21223890 TRUE 6361 819,508665 819,508665 0,052735 1 571,751272 30,85 1 25,8 5,689868887 0,175750974 0 ISLILR ICPL (N-term)

gi|21223890 TRUE 6443 1435,769178 718,388227 0,106346 2 578,0186139 31,52 1 60,5 6,032443146 0,165770315 0 YLLSLLPQNNR ICPL (N-term)

gi|21223890 TRUE 6436 1870,944608 624,31972 0,30534 3 577,4360806 100,91 1 28,9 4,937078867 0,202548922 0 IQEGVSALAGYGGIFQR ICPL (N-term)

TRUE phosphoglycerate kinase

[S. coelicolor A3(2)] gi|21220431 164,07 7,444168734 3 2 0,49 0,04 2 2,08 0,15 4090107 87 TRUE 3973 1553,788465 777,3978705 0,032059 2 346,012672 101,64 1 45,8 0,535940562 1,865878554 0 TIDELLSEGVAGK ICPL:13C(6) (K); ICPL:13C(6) (N-term)

gi|21220431 TRUE 1969 1877,887571 939,4474235 -0,096647 2 197,5054053 62,43 1 25,7 0,436218164 2,292430905 0 ADLNVPLDGTTITDDGR ICPL (N-term)

gi|21220431 TRUE 1970 1883,907712 628,6407547 0,058636 3 197,6072053 28,21 1 15,5 0,436218164 2,292430905 0 ADLNVPLDGTTITDDGR ICPL:13C(6) (N-term)

TRUE substrate binding protein

[S. coelicolor A3(2)] gi|21224757 162,88 5,904059041 3 3 4,37 0,91 3 0,26 0,05 6061620 63 TRUE 5084 1009,546522 505,276899 0,136402 2 449,7383746 43,2 1 19,9 5,198699489 0,1923558 0 IFDGLLAR ICPL (N-term)

gi|21224757 TRUE 1766 1236,585477 618,7963765 0,123047 2 182,3911808 61,44 1 33,4 5,329897604 0,187620865 0 SELDAVENVR ICPL (N-term)

gi|21224757 TRUE 3317 1476,744138 738,875707 0,080386 2 304,534297 58,24 1 9,7 2,49939384 0,400097009 0 AVTLPSAGQVTGDR ICPL (N-term)

TRUE regulatory protein

[S. coelicolor A3(2)] gi|21225529 156,62 6,374501992 3 3 5,88 2,55 3 0,27 0,12 6500887 75,4 TRUE 2089 1233,542234 617,274755 0,21829 2 206,5339803 71,28 1 40,7 7,550491056 0,132441717 0 AEDLFVSDK ICPL (K); ICPL (N-term)

gi|21225529 TRUE 3574 1322,605146 661,806211 0,207578 2 320,5363552 32,12 1 24,1 1,823261706 0,548467615 0 LTAEYAGYPK ICPL (K); ICPL (N-term)

gi|21225529 TRUE 3288 1446,733549 723,8704125 0,060775 2 302,7443472 53,22 1 10,6 7,52955365 0,132809997 0 LAQSGSGEQVLPR ICPL (N-term)

TRUE malate dehydrogenase

[S. coelicolor A3(2)] gi|21223204 153,19 9,118541033 3 3 0,7 0,07 3 1,48 0,14 5578430 98,8 TRUE 5354 1219,712369 610,3598225 0,134955 2 470,0174829 59,53 1 22,9 0,792333909 1,262094162 0 LLEITPALK ICPL:13C(6) (K); ICPL:13C(6) (N-term)

gi|21223204 TRUE 328 1312,584272 656,795774 0,099452 2 50,99539146 60,93 1 56,1 0,546473267 1,829915679 0 NAAETVNDEK ICPL:13C(6) (K); ICPL:13C(6) (N-term)

gi|21223204 TRUE 1705 1404,658109 702,8326925 0,121015 2 178,0217802 32,73 1 19,8 0,746914047 1,338842138 0 AINDHAADDIK ICPL:13C(6) (K); ICPL:13C(6) (N-term)

TRUE 30S ribosomal protein S3

[S. coelicolor A3(2)] gi|21223088 153,0469089 16,24548736 3 3 2,47 0,23 3 0,42 0,04 4174504 58,4 TRUE 3834 1104,536039 552,7716575 0,175685 2 336,7577472 53,68 1 27,1 2,051610236 0,487422017 0 LGITTDFK ICPL (K); ICPL (N-term)

gi|21223088 TRUE 368 1619,71832 810,362798 0,030804 2 54,187816 46,43 1 25,5 3,000467272 0,333281422 0 AEAPAAAAPAESTGTEA ICPL (N-term)

gi|21223088 TRUE 5246 2205,078184 735,6975787 0,262564 3 462,1563581 55,46 1 5,8 2,344909374 0,426455713 0 SPETDAQLVAQAVAEQLSSR ICPL (N-term)

TRUE glutamine synthetase I

[S. coelicolor A3(2)] gi|21220671 149,57 7,249466951 2 2 2,03 0,06 2 0,49 0,01 3056727 147,2 TRUE 6457 1893,985746 947,496511 -0,063822 2 579,258855 64,89 1 53,1 2,113597558 0,473126966 0 LVPGFEAPVNLVYSQR ICPL (N-term)

gi|21220671 TRUE 6051 2130,051526 710,6886927 0,216622 3 548,8353222 84,68 1 94,1 1,940170633 0,515418584 0 HAPSLLAFTNPTVNSYHR ICPL (N-term)

TRUE F0F1 ATP synthase subunit beta

[S. coelicolor A3(2)] gi|21223733 147,8069089 4,811715481 2 2 1,57 0,22 2 0,66 0,09 8707943 49,9 TRUE 5401 1370,735456 685,871366 0,008268 2 472,4286578 69,59 1 31,3 1,881461552 0,531501693 0 VIDLLTPYVK ICPL (K); ICPL (N-term)

gi|21223733 TRUE 6596 1546,77223 773,889753 0,156094 2 589,0787387 80,74 1 18,6 1,266890324 0,78933431 0 VALAGLTMAEYFR ICPL (N-term)

TRUE NLP/P60 family protein

[S. coelicolor A3(2)] gi|21222944 144,83 11,19133574 9 3 8,58 0,39 3 0,117 0,005 30438074 1523,8 TRUE 3079 869,520322 435,263799 0,016402 2 290,0714048 22,25 1 27,8 0 QVGISLPR

gi|21222944 TRUE 4729 974,541776 487,774526 -0,059252 2 424,065749 65,47 1 1238,9 8,431380435 0,11860454 0 QVGISLPR ICPL (N-term)

gi|21222944 TRUE 4734 974,541776 974,541776 -0,056976 1 424,1082994 27,58 1 25,4 8,431380435 0,11860454 0 QVGISLPR ICPL (N-term)

gi|21222944 TRUE 851 2530,134351 844,0496343 -0,073903 3 95,00164107 79,36 1 39,6 9,478253495 0,105504669 0 AYVSGATGPSAYDCSGLVQAAFK

ICPL (K); ICPL (N-term)

gi|21222944 TRUE 5144 2530,134351 844,0496343 0,037697 3 454,4439826 64,88 1 41,3 7,827121029 0,127760897 0 AYVSGATGPSAYDCSGLVQAAFK

ICPL (K); ICPL (N-term)

TRUE 50S ribosomal protein L7/L12

[S. coelicolor A3(2)] gi|21223035 141,4069089 20,47244094 5 2 0,85 0,08 2 1,19 0,11 26698390 172,6 TRUE 3733 1082,591932 541,799604 0,040992 2 330,6817472 42,22 1 49,1 0,963837913 1,037518846 0 ELTSLGLK ICPL:13C(6) (K); ICPL:13C(6) (N-term)

gi|21223035 TRUE 3754 1695,83769 848,422483 0,044034 2 331,7117888 77,65 1 43 0,740667152 1,350134128 0 DLVDGAPKPVLEK 2 ICPL (K); ICPL

(N-term)

gi|21223035 TRUE 3751 1713,898084 571,9708787 0,105364 3 331,725105 44,25 1 35,8 0,740667152 1,350134128 0 DLVDGAPKPVLEK 2 ICPL:13C(6) (K); ICPL:13C(6) (N-term)

TRUE F0F1 ATP synthase subunit alpha

[S. coelicolor A3(2)] gi|21223731 139,8069089 7,75047259 4 3 1,45 0,22 3 0,74 0,11 5803013 83,6 TRUE 4859 953,48393 477,245603 0,039394 2 433,2216327 41,07 1 26 1,417626203 0,705404568 0 VDLAQFR ICPL (N-term)

gi|21223731 TRUE 4898 1177,599988 589,303632 0,081336 2 436,4348829 43,58 1 25,3 1,907843036 0,524152135 0 ELLEYLHR ICPL (N-term)

gi|21223731 TRUE 2053 1681,847059 841,4271675 -0,004935 2 203,4243141 33,07 1 12,8 1,008298313 0,991769983 0 EEVGTVTLAGDGIAK ICPL:13C(6) (K); ICPL:13C(6) (N-term)

TRUE aldehyde dehydrogenase

[S. coelicolor A3(2)] gi|21223286 138,2707266 9,556313993 2 2 1,88 0,68 2 0,67 0,24 5443588 38,6 TRUE 3539 1552,775373 776,8913245 0,095351 2 318,0019717 67,25 1 16,5 2,714534702 0,36838726 1 ELEIAAADNLKR ICPL (K); ICPL (N-term)

gi|21223286 TRUE 4837 1695,776166 848,391721 -0,053442 2 431,8141074 78,59 1 22,1 1,039038407 0,962428332 0 TVWHPTGALGASGSSY ICPL (N-term)

TRUE secreted protein

[S. coelicolor A3(2)] gi|21220861 137,07 10,07194245 3 3 2,97 0,52 3 0,37 0,07 4366938 67,1 TRUE 4668 1090,615583 545,8114295 0,055941 2 419,1004658 32,6 1 20,5 4,053001239 0,246730741 0 ALRPYLPR ICPL (N-term)

gi|21220861 TRUE 4849 1122,594221 561,8007485 0,061103 2 432,5763661 65,94 1 33,4 2,894070906 0,345534036 0 VETAVFPVR ICPL (N-term)

gi|21220861 TRUE 5172 2512,267805 838,094119 -0,039057 3 456,5995831 38,53 1 13,2 1,947575436 0,51345893 0 ISNPSGAVALALDGTVIDTPDGPAR ICPL

(N-term)

TRUE gamma-glutamyltranspeptidase (putative secreted protein)

[S. coelicolor A3(2)] gi|21224715 136,687904 3,648424544 2 2 1,93 0,2 2 0,53 0,06 5076351 33,4 TRUE 1751 1192,595655 596,8014655 0,066869 2 181,0847802 80,38 1 15,2 1,649554965 0,606224116 0 DASAAGVEVLR ICPL (N-term)

gi|21224715 TRUE 3645 1411,66406 706,335668 0,190864 2 324,5853472 62,83 1 18,2 2,216862451 0,451087978 0 AELEALGHSFK ICPL (K); ICPL (N-term)

TRUE lipoprotein

[S. coelicolor A3(2)] gi|21222720 134,87 19,48051948 5 3 1,38 0,39 3 0,89 0,25 7164441 192,6 TRUE 1852 1251,604276 626,305776 0,101848 2 188,9799307 30 1 22,4 1,997341427 0,500665528 0 NLSPDEINK ICPL:13C(6) (K); ICPL:13C(6) (N-term)

gi|21222720 TRUE 2151 1286,568799 643,7880375 0,097925 2 211,2035637 71,56 1 53,8 1,448529414 0,690355329 0 FGVDPDIADK ICPL (K); ICPL (N-term)

gi|21222720 TRUE 2169 1298,609067 649,8081715 0,106457 2 212,7728469 57,58 1 16 0,668486471 1,495916587 0 FGVDPDIADK ICPL:13C(6) (K); ICPL:13C(6) (N-term)

TRUE succinyl-CoA synthetase subunit alpha

[S. coelicolor A3(2)] gi|21223187 134,27 9,523809524 3 3 0,76 0,11 3 1,41 0,21 2299277 41,3 TRUE 5022 838,486021 419,7466485 -0,020697 2 445,3841495 37,83 1 15,4 0,914985467 1,092913534 0 IGLVSK ICPL:13C(6) (K); ICPL:13C(6) (N-term)

gi|21223187 TRUE 1904 1109,566434 555,286855 0,01789 2 192,916089 57,24 1 17,6 0,508911562 1,964977953 0 EALEAAGVK ICPL:13C(6) (K); ICPL:13C(6) (N-term)

gi|21223187 TRUE 5141 1582,830289 791,9187825 0,046035 2 454,2547495 39,2 1 8,3 0,83986226 1,190671432 0 TGANVSVIFVPEK ICPL:13C(6) (K); ICPL:13C(6) (N-term)

TRUE aldehyde dehydrogenase

[S. coelicolor A3(2)] gi|21223287 131,9169089 3,958333333 2 2 1,83 0,02 2 0,546 0,006 2742823 39,5 TRUE 3812 1040,541105 520,7741905 0,130219 2 335,1505056 50,39 1 14,3 1,802212908 0,554873398 0 LTLELGGK ICPL (K); ICPL (N-term)

gi|21223287 TRUE 4893 1205,627317 603,3172965 0,086007 2 435,9380077 84,05 1 25,2 1,858838273 0,537970416 0 GVVNIITGDGR ICPL (N-term)

TRUE phosphomannomutase

[S. coelicolor A3(2)] gi|21223290 129,95 6,921675774 3 3 0,49 0,04 3 2,1 0,17 2004449 35,6 TRUE 1946 858,429899 858,429899 0,004301 1 195,8437301 35,1 1 9,8 0,426822836 2,342892451 0 DLSTAAGI ICPL:13C(6) (N-term)

gi|21223290 TRUE 1906 1470,659116 735,833196 0,169408 2 192,7596805 57,21 1 16,8 0,444165879 2,251411121 0 AWLAEDPDADTR ICPL:13C(6) (N-term)

gi|21223290 TRUE 6162 1907,960981 636,658511 0,010167 3 557,2347803 37,64 1 9 0,588455019 1,699365234 0 GAHGTFAESIVSSSLLAR ICPL (N-term)

TRUE phosphocarrier protein HPr

[S. coelicolor A3(2)] gi|21224185 128,67 27,95698925 3 2 0,42 0,16 2 3,08 1,18 18456974 54,9 TRUE 3533 1421,782606 711,394941 0,163518 2 317,6959136 60,21 1 13,9 0,222250642 4,499424567 0 AATATGVPVTIAK ICPL:13C(6) (K); ICPL:13C(6) (N-term)

gi|21224185 TRUE 2476 1503,757688 752,382482 0,003036 2 230,0641973 45,12 1 17,9 0,613382816 1,630303252 0 LVAEGLEELPETV ICPL (N-term)

gi|21224185 TRUE 2473 1509,77783 755,392553 -0,163106 2 229,7870219 68,46 1 23,1 0,613382816 1,630303252 0 LVAEGLEELPETV ICPL:13C(6) (N-term)

TRUE peptidyl-prolyl cis-trans isomerase

[S. coelicolor A3(2)] gi|21225776 126,16 18,18181818 3 2 0,7676 0,0003 2 1,3028 0,0005 15911496 93,2 TRUE 3756 1333,625028 667,316152 0,206096 2 331,7529968 61,21 1 22,8 0,768007312 1,302070937 0 FADENFQLK ICPL:13C(6) (K); ICPL:13C(6) (N-term)

gi|21225776 TRUE 3877 2422,119764 808,044772 -0,003816 3 339,8559301 53,53 1 28,4 0,767192469 1,303453879 1 VVEGTDVVDKIEGYGSSPAGK 2 ICPL (K); ICPL (N-term)

gi|21225776 TRUE 3873 2440,180158 814,0649033 -0,05011 3 339,6955221 64,95 1 42 0,767192469 1,303453879 1 VVEGTDVVDKIEGYGSSPAGK

2 ICPL:13C(6) (K); ICPL:13C(6) (N-term)

TRUE oxidoreductase (secreted protein)

[S. coelicolor A3(2)] gi|21225017 125,6 6,034482759 2 2 2,57 0,17 2 0,39 0,03 3419769 95,9 TRUE 6415 985,525403 493,2663395 0,124721 2 575,9657723 57,96 1 72,8 2,807988134 0,356126861 0 GVFALAFR ICPL (N-term)

gi|21225017 TRUE 1890 1478,691022 739,849149 0,067302 2 191,6114384 67,64 1 23,1 2,32389124 0,430312737 0 DVSPPGAGELEFR ICPL (N-term)

TRUE elongation factor G

[S. coelicolor A3(2)] gi|21223042 123,37 2,824858757 2 2 0,84 0,24 2 1,4 0,4 2048588 53,4 TRUE 2101 1208,634839 604,8210575 0,112885 2 207,3343808 62,84 1 34,1 0,521533514 1,91742232 0 NVAEEIIAK ICPL:13C(6) (K); ICPL:13C(6) (N-term)

gi|21223042 TRUE 5048 1271,710645 636,3589605 0,126679 2 447,2639074 60,53 1 19,3 1,146032327 0,872575735 0 GVQPLLDAVVR ICPL (N-term)

TRUE hypothetical protein SCO2999

[S. coelicolor A3(2)] gi|32141188 119,7748129 0,907441016 2 2 2,26 0,72 2 0,53 0,17 1799955 71,6 TRUE 6005 875,48863 438,247953 0,080694 2 545,1590971 43,42 1 50,3 3,167730167 0,315683454 0 LHFVVR ICPL (N-term)

gi|32141188 TRUE 6613 1193,667676 597,337476 0,158848 2 590,3089472 85,4 1 21,3 1,338918949 0,746871198 0 LLINLFEAR ICPL (N-term)

TRUE ketol-acid reductoisomerase

[S. coelicolor A3(2)] gi|21223870 119,6 7,831325301 2 2 0,685 0,007 2 1,46 0,02 3668295 25,1 TRUE 3884 1513,772428 757,389852 0,061496 2 340,3085888 79,48 1 21,8 0,695721974 1,43735578 0 VLAEIQDGTFAK ICPL:13C(6) (K); ICPL:13C(6) (N-term)

gi|21223870 TRUE 4933 1636,786664 546,267072 0,241284 3 438,7662743 40,12 1 3,3 0,674674231 1,482196821 0 DGDALFFGHGLNIR ICPL (N-term)

TRUE aspartate aminotransferase

[S. coelicolor A3(2)] gi|21223027 115,84 8,578431373 3 2 0,87 0,06 2 1,16 0,08 9645711 42,2 TRUE 3379 1220,626951 610,8171135 0,126573 2 308,712105 79,94 1 14,9 0,960773691 1,040827834 0 VTVEQLEAAR ICPL (N-term)

gi|21223027 TRUE 3376 1220,626951 610,8171135 0,134173 2 308,855464 55,63 1 21,8 0,960773691 1,040827834 0 VTVEQLEAAR ICPL (N-term)

gi|21223027 TRUE 5201 2668,325315 890,113289 -0,137967 3 458,7159906 35,9 1 5,5 0,787333108 1,270110439 0 VVLFVSPSNPTGAVYGEAETEAIGR

ICPL (N-term)

TRUE RecName: Full=30S ribosomal

protein S2 gi|6226896 112,3469089 6,451612903 2 2 3,98 0,1 2 0,252 0,006 780553 24,8 TRUE 4931 934,535612 467,771444 0,097312 2 438,6358994 57,84 1 15,9 4,113708657 0,243089651 0 ELLVLSR ICPL (N-term)

gi|6226896 TRUE 836 1402,768857 701,8880665 -0,024733 2 93,58639147 57,03 1 8,9 3,840517733 0,26038156 0 VIADAVAEGLIAR ICPL (N-term)

TRUE secreted protein

[S. coelicolor A3(2)] gi|21222370 111,61 14,35185185 2 2 1,26 0,02 2 0,8 0,01 6422491 25,7 TRUE 3452 1253,616086 627,311681 0,208638 2 313,0814885 47,13 1 16,4 1,224657711 0,816554692 0 VVFVSTDPER ICPL (N-term)

gi|21222370 TRUE 853 2263,135343 755,049965 0,014505 3 95,25589119 64,48 1 9,3 1,290120018 0,775121683 0 GIDPQIVGLTGEFDTIQAGARICPL (N-term)

TRUE spore-associated protein precursor

[S. coelicolor A3(2)] gi|21218951 110,33 16,88311688 2 2 0,41 0,12 2 2,9 0,88 3750017 30,7 TRUE 3208 1274,616392 637,811834 0,070332 2 298,1561552 38,15 1 19,7 0,567019443 1,763607955 0 SYAGPVYVSAR ICPL (N-term)

gi|21218951 TRUE 1695 1836,730695 918,8689855 -0,099371 2 177,1357386 72,18 1 11 0,248777449 4,019656948 0 NIESGADQYDEGDYR ICPL (N-term)

TRUE carboxypeptidase

[S. coelicolor A3(2)] gi|21224458 108,2 3,32594235 2 1 5,26 0 1 0,19 0 2118255 36 TRUE 6213 1522,822385 761,9148305 0,015739 2 561,1909467 94,82 1 2 5,261344406 0,19006549 0 TGTLTGAIALSGVTR ICPL (N-term)

gi|21224458 TRUE 6218 1522,822385 761,9148305 0,110939 2 561,4587387 108,2 1 34 5,261344406 0,19006549 0 TGTLTGAIALSGVTR ICPL (N-term)

TRUE hypothetical protein SCO3899

[S. coelicolor A3(2)] gi|21222306 105,4309952 6,666666667 3 2 1,45 0,14 2 0,7 0,07 6439976 46,4 TRUE 2955 1174,596342 587,801809 0,131982 2 282,1278885 51,15 1 7,9 1,653695758 0,604706153 0 SQVGATITHR ICPL (N-term)

gi|21222306 TRUE 3883 1670,806074 835,906675 0,01865 2 340,1508634 58,28 1 26 1,250012338 0,799992104 0 FTEAGVPIVGDDIK ICPL (K); ICPL (N-term)

gi|21222306 TRUE 3888 1682,846342 841,926809 -0,032418 2 340,3937637 44,26 1 12,5 1,250012338 0,799992104 0 FTEAGVPIVGDDIK ICPL:13C(6) (K); ICPL:13C(6) (N-term)

TRUE aminopeptidase

[S. coelicolor A3(2)] gi|21221092 101,54 2,761104442 2 2 0,92 0,04 2 1,09 0,04 1457242 52,3 TRUE 2007 1391,680112 696,343694 0,128012 2 200,4529803 52,47 1 32 0,872340629 1,146341196 0 IEGLDVDQELR ICPL (N-term)

gi|21221092 TRUE 1964 1441,623014 721,315145 0,14771 2 197,2898389 49,07 1 20,3 0,972884179 1,027871581 0 DLDPSEVFDGSR ICPL (N-term)

TRUE secreted protein

[S. coelicolor A3(2)] gi|21223402 101,067904 12,60504202 3 2 1,02 0,07 2 0,99 0,07 5610316 63,5 TRUE 3201 1094,566785 547,7870305 0,068339 2 297,6125216 45,3 1 43,1 0,913839768 1,094283742 0 TNPATQIK ICPL:13C(6) (K); ICPL:13C(6) (N-term)

gi|21223402 TRUE 3373 1262,525861 631,7665685 -0,073737 2 308,261472 36,02 1 11,3 1,124103793 0,889597568 0 WGLSYMDNR ICPL (N-term); Oxidation (M)

TRUE serine hydroxymethyltransferase

[S. coelicolor A3(2)] gi|21223213 95,08 4,365904366 2 2 0,73 0,1 2 1,42 0,2 2934893 43,6 TRUE 3976 1182,623243 591,8152595 0,164481 2 346,3128885 52,72 1 19,9 0,589982211 1,694966358 0 VEVPFLEK ICPL:13C(6) (K); ICPL:13C(6) (N-term)

gi|21223213 TRUE 1838 1476,707731 738,8575035 0,045593 2 187,8711466 42,36 1 23,7 0,878454945 1,13836231 0 VADAIGQEVADQR ICPL (N-term)

TRUE glutamate binding protein [

S. coelicolor A3(2)] gi|21224122 93,17099517 7,194244604 2 2 1,05 0,02 2 0,96 0,02 8270703 26,2 TRUE 3479 1199,548002 600,277639 0,138322 2 314,5939136 45,99 1 15,4 1,078362436 0,927332005 0 FDQPGLGQK ICPL (K); ICPL (N-term)

gi|21224122 TRUE 3838 1591,706327 796,3568015 0,129997 2 336,8231221 51,18 1 10,8 1,016248347 0,98401144 0 LGYTEDQIEWK ICPL (K); ICPL (N-term)

TRUE branched chain amino acid binding protein

[S. coelicolor A3(2)] gi|21220490 92,84 2,870813397 2 1 0,98 0 1 1,02 0 14501712 73,2 TRUE 3722 1539,638669 770,3229725 0,141255 2 329,2320554 84,05 1 36,8 0,981196968 1,019163361 0 VSFDEFGDATNK ICPL (K); ICPL (N-term)

gi|21220490 TRUE 3719 1551,678937 776,3431065 0,091187 2 329,1049888 92,84 1 36,4 0,981196968 1,019163361 0 VSFDEFGDATNK ICPL:13C(6) (K); ICPL:13C(6) (N-term)

TRUE hydrolytic protein

[S. coelicolor A3(2)] gi|21222651 92,35 3,966597077 2 2 2,28 0,15 2 0,44 0,03 7003307 46 TRUE 3363 933,515211 467,2612435 0,068513 2 307,7861466 44,73 1 38 2,488865886 0,401789428 0 LQLEAVR ICPL (N-term)

gi|21222651 TRUE 3610 1534,688245 767,8477605 0,086079 2 322,5100634 47,62 1 8 2,066246784 0,483969295 0 EAVSPMEGFTLK ICPL (K); ICPL (N-term); Oxidation (M)

TRUE zinc protease

[S. coelicolor A3(2)] gi|21224181 90,95 2,444444444 2 1 0,83 0 1 1,21 0 2195481 24,6 TRUE 6298 1180,647306 590,827291 0,166018 2 567,5051216 90,95 1 17,8 0,826137409 1,210452388 0 TAVAAGFGLLR ICPL (N-term)

gi|21224181 TRUE 6296 1180,647306 590,827291 0,190618 2 567,2841803 59,54 1 6,8 0,826137409 1,210452388 0 TAVAAGFGLLR ICPL (N-term)

TRUE secreted protease

[S. coelicolor A3(2)] gi|21221367 89,92690887 1,408450704 3 2 1,61 0,06 2 0,62 0,03 10659063 87,1 TRUE 5285 1419,694303 710,3507895 0,053221 2 464,7971741 92,45 1 21,7 1,703643359 0,586977312 0 LVYPLDAYAGK ICPL (K); ICPL (N-term)

gi|21221367 TRUE 5296 1419,694303 710,3507895 0,069021 2 465,9071074 61,41 1 23,8 1,520601567 0,657634466 0 LVYPLDAYAGK ICPL (K); ICPL (N-term)

gi|21221367 TRUE 5284 1431,734571 716,3709235 0,146753 2 465,120941 90,61 1 41,6 1,703643359 0,586977312 0 LVYPLDAYAGK ICPL:13C(6) (K); ICPL:13C(6) (N-term)

TRUE superoxide dismutase

[S. coelicolor A3(2)] gi|21223621 88,62 12,21374046 2 2 0,56 0,23 2 2,38 0,97 8927390 163 TRUE 3928 1045,518049 523,2626625 0,136875 2 343,0263888 36,9 1 25,1 0,826962112 1,209245243 0 IFWETK ICPL:13C(6) (K); ICPL:13C(6) (N-term)

gi|21223621 TRUE 3868 1371,698179 686,3527275 0,132545 2 339,1213632 51,72 1 137,9 0,284007213 3,521037332 0 ALDYIAQIDK ICPL:13C(6) (K); ICPL:13C(6) (N-term)

TRUE hypothetical protein SCO1074

[S. coelicolor A3(2)] gi|21219589 87,27 4,4345898 2 2 1,5 1,54 2 2,05 2,09 9690334 30,7 TRUE 4877 1253,673246 627,340261 0,131878 2 434,512933 60,1 1 11,5 2,475853749 0,403901079 0 LSVAEAHFLR ICPL:13C(6) (N-term)

gi|21219589 TRUE 3494 1292,600172 646,803724 0,173352 2 315,3208554 27,17 1 19,2 0,296412882 3,37367254 0 SVADFDQFPR ICPL:13C(6) (N-term)

TRUE hypothetical protein SCO4253

[S. coelicolor A3(2)] gi|21222648 86,38 2,434456929 2 2 2,86 1,01 2 0,44 0,15 1595763 59,9 TRUE 2092 1414,612104 707,80969 0,09502 2 206,6966554 86,38 1 44,2 4,104388961 0,243641626 0 LAQFSSGGGELDE ICPL (N-term)

gi|21222648 TRUE 2124 1414,612104 707,80969 0,09962 2 208,9758139 47,16 1 15,7 1,596619406 0,62632334 0 LAQFSSGGGELDE ICPL (N-term)

TRUE nucleoside diphosphate kinase

[S. coelicolor A3(2)] gi|2125898 85,67 17,51824818 2 2 2,68 0,84 2 0,44 0,14 10367040 32,3 TRUE 6318 940,50395 470,755613 0,056974 2 568,9362134 37,34 1 29,9 1,613652195 0,61971223 1 IFFPGRV ICPL (N-term)

gi|2125898 TRUE 3457 1688,906156 844,956716 -0,102632 2 313,2272634 48,33 1 2,4 3,742709959 0,267186079 0 ALAGPTDPIAAAPGSIR ICPL:13C(6)

(N-term)

TRUE hypothetical protein SCO4175

[S. coelicolor A3(2)] gi|21222572 83,31690887 20 2 2 0,3 0,16 2 5,23 2,8 966140 45,7 TRUE 6039 1148,699415 574,8533455 0,181709 2 547,881047 29,66 1 43,8 0,12235904 8,172669568 0 VLLRPLGGGR ICPL:13C(6) (N-term)

gi|21222572 TRUE 4863 1810,917798 604,3107833 0,11705 3 433,4634413 56,18 1 1,9 0,469793457 2,12859499 0 AAFDHSAVTDANVLLR ICPL:13C(6)

(N-term)

TRUE electron transfer flavoprotein, alpha subunit

[S. coelicolor A3(2)] gi|21219596 75,64 9,375 2 2 3,88 1,2 2 0,31 0,1 1910647 34,1 TRUE 6449 1464,82089 732,914083 0,102034 2 578,5569307 43,27 1 26,2 5,406675267 0,184956549 0 KPTLELLTLAR ICPL (K); ICPL (N-term)

gi|21219596 TRUE 6211 2089,082487 697,0323463 0,065861 3 561,0447222 32,37 1 7,9 2,338959112 0,427540608 0 SVSPQLYIASGISGAIQHR ICPL (N-term)

TRUE acyl carrier protein [

S. coelicolor A3(2)] gi|21220856 72,66 17,07317073 3 2 0,683 0,004 2 1,464 0,008 1772240 109,4 TRUE 1792 964,452283 482,7297795 0,062641 2 184,2033717 29,76 1 44,1 0,688075469 1,453328951 0 YILDHQA ICPL (N-term)

gi|21220856 TRUE 1796 964,452283 482,7297795 0,089041 2 184,5372805 31,84 1 25,9 0,688075469 1,453328951 0 YILDHQA ICPL (N-term)

gi|21220856 TRUE 1888 1023,482068 512,244672 0,039856 2 191,6277552 40,82 1 39,4 0,67777149 1,475423523 0 IPDDDVK ICPL:13C(6) (K); ICPL:13C(6) (N-term)

TRUE purine nucleoside phosphorylase

[S. coelicolor A3(2)] gi|21223291 70,29 6,934306569 4 2 1,44 0,08 2 0,7 0,04 3458571 86 TRUE 6294 880,503934 440,755605 0,10819 2 567,1213222 41,18 1 37,9 1,328636021 0,75265158 0 ALVFLGR ICPL (N-term)

gi|21223291 TRUE 4867 1379,709989 690,3586325 0,163135 2 433,7728823 29,11 1 13,8 1,544930655 0,647278243 0 TIILTNGCGGLR ICPL (N-term)

________________________________________________________________________________________________________________________________________________________________________________________________________________________________________________

Extracellular proteins of *S. coelicolor* M145 were labelled with C12 and those of *S. coelicolor* M28 were labelled with C13 at 24h of growth.
