# Supplementary material for: A Novel Two-Component System Involved in the Transition to Secondary Metabolism in Streptomyces coelicolor
Source: PLoS One. 2012 Feb 9;7(2):e31760. doi: 10.1371/journal.pone.0031760 (PMC3276577; doi:10.1371/journal.pone.0031760)
Supplement: Table S4 — ICPL analysis of extracellular proteins of S. coelicolor M28 labelled with C12. Extracellular proteins of S. coelicolor M145 were labelled with C13 and those of S. coelicolor M28 were labelled with C12 at 24 h of growth. (DOC) [file pone.0031760.s005.doc]

**Table S4. ICPL analysis of extracellular proteins of *S. coelicolor* M28 labelled with C12**

**_________________________________________________________________________________________________________________________________________________________________________________________________________________________________________________**

Protein List: D:\Daniel Rozas ICPL_junio_baf.d\BTDataExchange_1\ProteinList.WARPResult

WARP-LC Method: D:\Methods\WarpLCMethods\ICPL_NCBI trypsin streptomyces_C12C13.WarpLCMethod

BioTools Method: ICPL_NCBI_trypsin_streptomyces_C12C13

Computer Name: MSMSANALYSIS

Rel. Protein Name and Species Accession Score SC [%] # Pept. # (L/H) Avg. Avg. (SD (L/H)) # (H/L) Avg. Avg. (SD (H/L)) Abs. Inten. S/N

TRUE secreted protein [S. coelicolor A3(2)] gi|21224522 2579,23002 39,53287197 46 30 7,89 0,75 30 0,16 0,02 183759704 2030,4

TRUE nucleotidase [S. coelicolor A3(2)] gi|21220497 1087,577904 29,06976744 32 18 0,65 0,05 18 1,69 0,13 115130699 1043,4

TRUE succinyl-CoA synthetase subunit beta [S. coelicolor A3(2)] gi|21223186 1004,785808 36,54822335 26 15 1,01 0,06 15 1,03 0,06 53023045 787,1

TRUE esterase [S. coelicolor A3(2)] gi|21224437 987,0924484 32,5 29 16 1,38 0,1 16 0,79 0,06 119323838 1307,7

TRUE secreted protein [S. coelicolor A3(2)] gi|21218846 911,0338177 37,64705882 20 14 15,29 4,53 14 0,15 0,04 74420550 979,3

TRUE molecular chaperone DnaK [S. coelicolor A3(2)] gi|32141213 862,6837121 28,3171521 17 14 2,13 0,14 14 0,5 0,03 48044733 516,4

TRUE secreted esterase [S. coelicolor A3(2)] gi|21221495 848,2817218 24,64589235 18 12 1,21 0,07 12 0,86 0,05 74985216 704,9

TRUE oligopeptide-binding lipoprotein [S. coelicolor A3(2)] gi|21223834 706,0177984 23 21 14 1,32 0,09 14 0,81 0,06 69752691 802,2

TRUE chaperonin GroEL [S. coelicolor A3(2)] gi|21222689 702,9183671 23,65988909 19 11 2,26 0,28 11 0,52 0,06 44968045 667,1

TRUE hypothetical protein SCO4584 [S. coelicolor A3(2)] gi|21222967 644,4496258 34,28571429 19 14 0,23 0,03 14 5,06 0,58 79193059 756,1

TRUE secreted protein [S. coelicolor A3(2)] gi|21220594 592,717904 11,55885472 12 8 1,03 0,12 8 1,08 0,13 19857282 419,9

TRUE SLPI=protease inhibitor [S. lividans, 66, Peptide, 107 aa] gi|257239 533,82 99,06542056 38 13 21,13 5,6 13 0,09 0,02 406309668 3064,6

TRUE secreted protein [S. coelicolor A3(2)] gi|21224521 517,38 31,11111111 18 9 9,36 0,96 9 0,12 0,01 103479339 950,4

TRUE tellurium resistance protein [S. coelicolor A3(2)] gi|21222670 515,09 45,02617801 16 11 2,05 0,15 11 0,52 0,04 151283619 1245,6

TRUE dihydrolipoamide dehydrogenase [S. coelicolor A3(2)] gi|21220654 506,8807266 16,25514403 11 6 2,24 0,31 6 0,5 0,07 28055469 360,8

TRUE secreted esterase [S. coelicolor A3(2)] gi|21224523 494,1738177 10,90174966 10 7 5,54 0,91 7 0,21 0,04 28596241 444,9

TRUE secreted hydrolase [S. coelicolor A3(2)] gi|21224438 481,0469089 22,28796844 11 9 1,4 0,11 9 0,76 0,06 47228947 297

TRUE phosphomannomutase [S. coelicolor A3(2)] gi|21223290 480,4169089 14,38979964 10 7 1,59 0,16 7 0,67 0,07 33051825 219,4

TRUE hypothetical protein SCO2368 [S. coelicolor A3(2)] gi|21220836 465,67 43,45549738 17 9 1,17 0,09 9 0,91 0,07 143260807 1197,2

TRUE tellurium resistance protein [S. coelicolor A3(2)] gi|32141118 459,7969089 47,64397906 21 9 0,87 0,04 9 1,17 0,06 60485151 948,9

TRUE transcriptional regulator [S. coelicolor A3(2)] gi|21221840 435,2809952 32,80632411 13 8 1,33 0,15 8 0,82 0,09 61497422 553,6

TRUE secreted protein [S. coelicolor A3(2)] gi|21220348 431,8969089 20,29850746 10 7 7,45 2,1 7 0,21 0,06 26214133 455,3

TRUE secreted 5'-nucleotidase [S. coelicolor A3(2)] gi|21222550 423,4932856 14,54248366 9 7 1,31 0,12 7 0,81 0,07 16319444 230,6

TRUE isocitrate dehydrogenase [S. coelicolor A3(2)] gi|21225286 417,4853816 11,23139378 8 6 2,23 0,14 6 0,46 0,03 18774179 27,9

TRUE hypothetical protein SCO3767 [S. coelicolor A3(2)] gi|21222178 411,8069089 47,01986755 13 9 2,04 0,19 9 0,53 0,05 28556860 448,8

TRUE secreted protein [S. coelicolor A3(2)] gi|21220483 388,8119903 14,23785595 12 7 1,17 0,08 7 0,88 0,06 18180826 310,1

TRUE co-chaperonin GroES [S. coelicolor A3(2)] gi|21223140 374,91 72,54901961 17 8 2,31 0,2 8 0,46 0,04 35961193 558

TRUE type II citrate synthase [S. coelicolor A3(2)] gi|21221189 363,8209952 13,28671329 8 5 0,68 0,02 5 1,48 0,05 23463383 276,1

TRUE secreted esterase [S. coelicolor A3(2)] gi|21224890 362,14 8,843537415 8 6 1,69 0,14 6 0,61 0,05 17508233 308,1

TRUE secreted tripeptidylaminopeptidase [S. coelicolor A3(2)] gi|21219739 338,2469089 15,34195933 8 6 0,31 0,03 6 3,41 0,29 19271280 216,2

TRUE GroEL1 [S. coelicolor] gi|406598 322,442717 13,88888889 9 6 3,34 0,46 6 0,33 0,05 9918523 200,3

TRUE 4-aminobutyrate aminotransferase

[S. coelicolor A3(2)] gi|21224026 321,5169089 9,684684685 4 3 0,36 0,06 3 3,02 0,51 11153792 116,6

TRUE elongation factor Ts [S. coelicolor A3(2)] gi|21223980 315,062559 21,94244604 8 5 0,44 0,05 5 2,42 0,27 22738425 282,4

TRUE triosephosphate isomerase [S. coelicolor A3(2)] gi|21220430 300,9617218 19,37984496 5 4 0,92 0,12 4 1,16 0,16 7667642 92,5

TRUE succinyl-CoA synthetase subunit alpha [S. coelicolor A3(2)] gi|21223187 280,51 18,36734694 11 6 0,97 0,16 6 1,21 0,21 15571114 277,6

TRUE BldKB [S. coelicolor] gi|1532202 279,89 9,800664452 5 4 0,7 0,09 4 1,51 0,19 3741359 112,7

TRUE elongation factor gi|1091582 274,96 15,11335013 5 4 3,18 0,57 4 0,36 0,06 9534525 217,5

TRUE secreted tripeptidyl aminopeptidase [S. coelicolor A3(2)] gi|21221213 271,8515638 14,34599156 7 4 0,22 0,04 4 5,19 1,02 13279783 208,4

TRUE malate dehydrogenase [S. coelicolor A3(2)] gi|21223204 270,9369089 16,71732523 6 6 1,12 0,12 6 0,95 0,1 25764442 255,6

TRUE secreted protease [S. coelicolor A3(2)] gi|21221367 264,872717 5,377720871 11 7 0,27 0,02 7 3,88 0,31 30191890 548,2

TRUE aconitate hydratase [S. coelicolor A3(2)] gi|21224335 264,3738177 4,756637168 5 3 1,04 0,14 3 1,01 0,13 9494441 123,6

TRUE secreted protein [S. coelicolor A3(2)] gi|21224891 260,2 24,4047619 7 7 2,13 0,39 7 0,58 0,11 4751273 125,2

TRUE ribosome recycling factor [S. coelicolor A3(2)] gi|21223982 243,0909952 25,94594595 5 3 2,3 0,37 3 0,47 0,08 9873915 146,5

TRUE secreted protein [S. coelicolor A3(2)] gi|21221339 241,77 9,471365639 5 4 1,29 0,14 4 0,81 0,09 6296381 311,3

TRUE secreted protein [S. coelicolor A3(2)] gi|21223402 238,3269089 18,06722689 7 5 1,37 0,1 5 0,75 0,06 32053289 210,5

TRUE secreted protein [S. coelicolor A3(2)] gi|21224075 237,73 16,98113208 4 3 2,22 0,19 3 0,46 0,04 7024383 104

TRUE transaldolase [S. coelicolor A3(2)] gi|21220422 235,9538177 10,48387097 5 3 0,5 0,04 3 2,05 0,15 11285724 157,6

TRUE ORF3 [S. coelicolor A3(2)] gi|565055 235,42 27,10280374 4 4 12,05 1,44 4 0,09 0,01 12042173 214,8

TRUE cytochrome c oxidase subunit II [S. coelicolor A3(2)] gi|21220633 215,23 13,1661442 6 4 2,36 0,22 4 0,44 0,04 15915124 211,5

TRUE glutamate binding protein [S. coelicolor A3(2)] gi|21224122 207,2909952 13,30935252 5 3 2,23 0,28 3 0,47 0,06 22872845 86,4

TRUE DNA polymerase III subunit beta [S. coelicolor A3(2)] gi|21222286 197,127904 22,60638298 5 5 2,31 0,12 5 0,44 0,02 4799719 107,4

TRUE hypothetical protein SCO3324 [S. coelicolor A3(2)] gi|21221755 195,5 6,92124105 4 3 10,38 1,6 3 0,1 0,02 12912251 150,2

TRUE superoxide dismutase [S. coelicolor A3(2)] gi|21219516 189,43 20,46511628 7 4 0,85 0,1 4 1,24 0,14 46837800 220,3

TRUE NLP/P60 family protein [S. coelicolor A3(2)] gi|21222944 188,63 13,71841155 11 5 0,25 0,02 5 4,06 0,32 58838034 624,1

TRUE hypothetical protein SCO2367 [S. coelicolor A3(2)] gi|21220835 188,1369089 12,56544503 2 2 1,17 0,13 2 0,88 0,09 7003286 64,1

TRUE 50S ribosomal protein L7/L12 [S. coelicolor A3(2)] gi|21223035 185,55 23,62204724 6 5 2,37 0,27 5 0,45 0,05 7435334 145,6

TRUE bifunctional protein (methylenetetrahydrofolate

dehydrogenase and methenyltetrahydrofolate cyclohydrolase)

[S. coelicolor A3(2)] gi|21223201 183,5 10,21126761 2 2 1,7 0,04 2 0,59 0,01 6574389 51,2

TRUE serine/threonine protein kinase [S. coelicolor A3(2)] gi|32141292 182,81 4,361617764 3 3 0,24 0,1 3 6,58 2,85 15180180 108,2

TRUE phosphoglycerate kinase [S. coelicolor A3(2)] gi|21220431 178,3109952 15,13647643 4 3 1,16 0,07 3 0,87 0,05 6061545 72,2

TRUE RNA polymerase alpha subunit [S. coelicolor A3(2)] gi|1524340 172,4984727 10,32448378 4 1 2,31 0 1 0,43 0 5901180 107,7

TRUE carboxypeptidase [S. coelicolor A3(2)] gi|21224458 168,9 5,321507761 2 2 0,38 0,16 2 3,57 1,52 4389916 77,5

TRUE lipoprotein [S. coelicolor A3(2)] gi|21222720 164,12 13,63636364 4 4 3,65 0,77 4 0,32 0,07 6165196 149,7

TRUE thioredoxin [S. coelicolor A3(2)] gi|21223779 162,34 7,547169811 5 4 2,05 0,23 4 0,51 0,06 5648806 126,1

TRUE branched chain amino acid binding protein [S. coelicolor A3(2)] gi|21220490 156,92 4,545454545 4 4 2,53 0,46 4 0,45 0,08 2563695 72,7

TRUE nucleoside diphosphate kinase [S. coelicolor A3(2)] gi|21221070 156,7009952 27,00729927 5 3 2,44 0,61 3 0,49 0,12 28991776 131,5

TRUE secreted hydrolase [S. coelicolor A3(2)] gi|21220452 154,26 10,38062284 3 3 1,77 0,08 3 0,57 0,03 8923693 28,8

TRUE peptidyl-prolyl cis-trans isomerase [S. coelicolor A3(2)] gi|21225776 154,14 16,36363636 2 2 1,23 0,53 2 1,12 0,49 25372475 70,6

TRUE aminopeptidase N [S. coelicolor A3(2)] gi|21221100 154,0815638 3,967327888 3 3 1,37 0,19 3 0,77 0,11 2611393 41,9

TRUE superoxide dismutase [S. coelicolor A3(2)] gi|21223621 150,18 22,13740458 9 7 2,37 0,24 7 0,45 0,05 16000198 236,9

TRUE 30S ribosomal protein S1 [S. coelicolor A3(2)] gi|21220480 149,0209952 5,378486056 5 3 4,4 0,64 3 0,24 0,04 20217073 1406,7

TRUE metallopeptidase [S. coelicolor A3(2)] gi|21225030 148,19 4,545454545 4 2 1,13 0,16 2 0,92 0,13 5672238 166,3

TRUE NLP/P60 family secreted protein [S. coelicolor A3(2)] gi|21223174 143,8463767 9,547738693 4 3 1,94 0,1 3 0,52 0,03 4062569 69,1

TRUE secreted protein [S. coelicolor A3(2)] gi|21220087 137,08 5,314009662 2 2 6,36 1,89 2 0,18 0,05 4592062 72,5

TRUE hydrolase [S. coelicolor A3(2)] gi|21222125 134,05 7,913669065 5 3 1,04 0,03 3 0,96 0,03 9122639 125,9

TRUE Rieske iron-sulfur protein [S. coelicolor A3(2)] gi|21220626 130,69 5,949008499 6 4 9,69 2,17 4 0,12 0,03 60275086 549,4

TRUE hypothetical protein SCO3899 [S. coelicolor A3(2)] gi|21222306 127,7309952 8,055555556 2 2 3,62 0,74 2 0,3 0,06 5901918 55,9

TRUE phosphoglyceromutase [S. coelicolor A3(2)] gi|21222605 125,43 7,114624506 3 2 0,35 0,13 2 3,65 1,34 3033606 148

TRUE phosphocarrier protein HPr [S. coelicolor A3(2)] gi|21224185 118,86 27,95698925 2 2 1,76 0,41 2 0,63 0,15 1981532 28,2

TRUE hypothetical protein SCO1384 [S. coelicolor A3(2)] gi|21219888 118,5669089 7,490636704 2 2 1,18 0,18 2 0,88 0,13 3229616 34,8

TRUE secreted protein [S. coelicolor A3(2)] gi|21221536 118,32 10,6557377 3 3 0,52 0,09 3 2,08 0,36 1968133 78,7

TRUE D-alanyl-D-alanine dipeptidase (putative secreted protein)

[S. coelicolor A3(2)] gi|21219900 117,32 6,569343066 2 2 0,79 0,02 2 1,27 0,04 1097254 38,9

TRUE serine hydroxymethyltransferase [S. coelicolor A3(2)] gi|21223213 117,2569089 4,98960499 2 2 2,42 1,04 2 0,57 0,24 1836250 49,5

TRUE fructose-bisphosphate aldolase [S. coelicolor A3(2)] gi|21222064 115,0769089 7,871720117 2 2 1,15 0,27 2 0,96 0,22 3568284 39

TRUE membrane protein [S. coelicolor A3(2)] gi|21223676 114,8409952 12,21122112 3 2 0,25 0,01 2 4,04 0,18 5971224 89,2

TRUE lipoprotein [S. coelicolor A3(2)] gi|21223308 114,097904 9,661835749 3 2 0,63 0,37 2 2,65 1,54 7679110 79,3

TRUE secreted alkaline phosphatase [S. coelicolor A3(2)] gi|32141154 113,8 3,266787659 2 2 0,43 0,12 2 2,69 0,76 8898931 93

TRUE large secreted protein [S. coelicolor A3(2)] gi|21220395 112,14 3,588907015 2 2 0,35 0,14 2 3,79 1,51 6518716 48,4

TRUE 50S ribosomal protein L3 [S. coelicolor A3(2)] gi|21223082 107,1909952 10,28037383 4 2 3,23 1,45 2 0,43 0,19 1986368 60,5

TRUE hypothetical protein SCO5725 [S. coelicolor A3(2)] gi|21224072 100,12 20,38834951 2 2 0,32 0,03 2 3,21 0,31 2523774 85,2

TRUE purine nucleoside phosphorylase [S. coelicolor A3(2)] gi|21223291 99,89 6,934306569 5 2 0,58 0,03 2 1,73 0,1 17782567 135,9

TRUE aldose 1-epimerase [S. coelicolor A3(2)] gi|21220874 95,99 6,284153005 2 2 4,97 1,4 2 0,23 0,07 2508775 64,7

TRUE 50S ribosomal protein L17 [S. coelicolor A3(2)] gi|21223109 95,73099517 8,928571429 2 1 7,65 0 1 0,13 0 630802 31,7

TRUE oxidoreductase (secreted protein) [S. coelicolor A3(2)] gi|21225017 95,38 8,045977011 3 2 6,51 1,31 2 0,17 0,03 3180636 92

TRUE hypothetical protein SCO2093 [S. coelicolor A3(2)] gi|21220573 94,55 11,37724551 2 2 0,26 0,02 2 3,88 0,31 2013275 21,3

TRUE F0F1 ATP synthase subunit beta [S. coelicolor A3(2)] gi|21223733 94,21690887 3,138075314 2 2 3,2 0,27 2 0,32 0,03 9375799 77,9

TRUE spore-associated protein precursor [S. coelicolor A3(2)] gi|21218951 93,81 11,68831169 2 2 2,16 0,15 2 0,47 0,03 3402201 92,9

TRUE secreted protein [S. coelicolor A3(2)] gi|21222370 88,36 16,66666667 2 2 3,64 0,02 2 0,275 0,002 11972660 89,6

TRUE 50S ribosomal protein L14 [S. coelicolor A3(2)] gi|21223092 88,00381774 18,03278689 2 2 2,96 1,24 2 0,46 0,19 1809391 53,8

TRUE glycine cleavage system H protein [S. coelicolor A3(2)] gi|21223828 81,02 21,6 2 2 0,83 0,04 2 1,21 0,06 962732 33,6

TRUE hypothetical protein SCO1074 [S. coelicolor A3(2)] gi|21219589 79,39 5,764966741 2 2 1,28 0,16 2 0,8 0,1 9641671 38,9

TRUE polypeptide deformylase [S. coelicolor A3(2)] gi|21223589 78,2 11,57407407 3 2 0,783 0,006 2 1,28 0,01 1617358 86,2

TRUE translation initiation factor IF-2 [S. coelicolor A3(2)] gi|32141270 76,46381774 1,74249758 2 2 1,02 0,91 2 2,55 2,29 1661656 37,9

TRUE hypothetical protein SCO3659 [S. coelicolor A3(2)] gi|21222074 74,45 17,97752809 2 2 0,58 0,18 2 2,04 0,63 2699395 51,6

TRUE cystathionine gamma-synthase [S. coelicolor A3(2)] gi|21223331 74,31 9,067357513 2 2 0,66 0,19 2 1,76 0,51 11510919 59,4

TRUE secreted protein [S. coelicolor A3(2)] gi|21220689 73,62 5,189620758 2 2 1,09 0,21 2 0,99 0,19 2307141 57,8

TRUE hypothetical protein SCO6176 [S. coelicolor A3(2)] gi|21224501 61,86099517 9,782608696 2 2 1,67 0,22 2 0,62 0,08 695631 59,7

Protein List: D:\Daniel Rozas ICPL_junio_baf.d\BTDataExchange_1\ProteinList.WARPResult

WARP-LC Method: D:\Methods\WarpLCMethods\ICPL_NCBI trypsin streptomyces_C12C13.WarpLCMethod

BioTools Method: ICPL_NCBI_trypsin_streptomyces_C12C13

Computer Name: MSMSANALYSIS

Proteins: Peptides:

Rel. Protein Name

and Species Accession Score SC [%] # Pept. # (L/H) Avg. Avg. (SD (L/H)) # (H/L) Avg. Avg. (SD (H/L)) Abs. Inten. S/N Rel. Cmpd MH+ (calc) [Da] m/z (calc) _ m [Da] z RT [min] Ions Sc. Rank S/N L/H H/L Miss Sequence Variable Modification

TRUE secreted

protein

[S. coelicolor

A3(2) gi|21224522 2579,23002 39,53287197 46 30 7,89 0,75 30 0,16 0,02 183759704 2030,4 TRUE 3360 876,504987 438,7561315 -0,112263 2 293,0308272 39,99 1 79,1 4,874432772 0,205152075 1 RVDIL ICPL(N-term)

gi|21224522 TRUE 3683 885,425352 443,216314 -0,154228 2 313,2469691 45,99 1 28,7 3,370358597 0,296704333 0 AVTAWK ICPL (K); ICPL (N-term)

gi|21224522 TRUE 2119 1023,489408 512,248342 -0,119084 2 188,6268685 49,15 1 28,5 11,34662839 0,088131907 0 ILGDNPGK ICPL (K); ICPL (N-term)

gi|21224522 TRUE 2022 1050,521421 525,7643485 -0,146097 2 182,9949682 62,17 1 18 5,3939142 0,185394124 0 TAELQDLR ICPL (N-term)

gi|21224522 TRUE 5025 1196,609205 598,8082405 -0,114881 2 423,691229 70,13 1 20,1 3,888721976 0,257153894 0 TVLGAMALSGR ICPL (N-term); Oxidation M

gi|21224522 TRUE 5089 1412,691678 471,5687433 -0,13963 3 427,3101874 47,49 1 25,5 17,27390264 0,057890798 0 FNTLSIQNSAGR ICPL (N-term)

gi|21224522 TRUE 5086 1412,691678 706,849477 -0,121754 2 427,1559293 98,57 1 112 17,27390264 0,057890798 0 FNTLSIQNSAGR ICPL (N-term)

gi|21224522 TRUE 2198 1449,696775 725,3520255 -0,075051 2 193,8663517 106,12 1 33 7,115756095 0,140533204 0 EAALTAAAEAEAAR ICPL (N-term)

gi|21224522 TRUE 1607 1459,703626 487,2393927 -0,175778 3 155,030485 50,8 1 37,6 3,729289955 0,268147559 0 TAVQASSAAHNAAR ICPL (N-term)

gi|21224522 TRUE 1604 1459,703626 730,355451 -0,170902 2 154,853877 95,99 1 90,3 3,729289955 0,268147559 0 TAVQASSAAHNAAR ICPL (N-term)

gi|21224522 TRUE 1870 1545,729169 515,9145737 -0,125321 3 172,6489517 39,31 1 32,2 5,843905038 0,171118455 0 LAHDTEQSLAEAR ICPL (N-term)

gi|21224522 TRUE 1868 1545,729169 773,3682225 -0,123245 2 172,6742679 79,85 1 87,8 5,843905038 0,171118455 0 LAHDTEQSLAEAR ICPL (N-term)

gi|21224522 TRUE 5080 1549,77168 775,389478 -0,175156 2 426,7807794 135,86 1 87,9 8,804564384 0,113577453 0 AQSLANTAASAAAAAR ICPL (N-term)

gi|21224522 TRUE 5078 1549,77168 517,2620773 -0,170032 3 426,7245623 88,92 1 63,2 8,804564384 0,113577453 0 AQSLANTAASAAAAAR ICPL (N-term)

gi|21224522 TRUE 5175 1611,787351 806,3973135 -0,162427 2 432,6317207 92,68 1 29,3 5,382055957 0,185802602 0 SAFAATAAAQAAATAGR ICPL (N-term)

gi|21224522 TRUE 4160 1654,807111 827,9071935 -0,125787 2 344,2948939 96,41 1 26,5 10,50131734 0,095226148 0 GVSQILEDASQGLK ICPL (K); ICPL (N-term)

gi|21224522 TRUE 4085 1654,785947 827,8966115 -0,103823 2 339,6685355 116,03 1 31,3 4,173571798 0,239602922 0 AWAADSVAAAELAAK ICPL( K); ICPL

(N-term)

gi|21224522 TRUE 1103 1694,88603 847,946653 -0,017706 2 103,6459499 83,91 1 47,1 13,46643814 0,074258686 0 AAVNLISTTIGTWSR ICPL (N-term)

gi|21224522 TRUE 1873 1713,782667 857,3949715 -0,186343 2 172,8144765 82,27 1 48,2 5,348124219 0,186981446 1 NAATEADRLDAQTK ICPL (K); ICPL

(N-term)

gi|21224522 TRUE 3797 1733,860532 867,433904 0,030192 2 320,4388693 104,08 1 94,8 13,97170075 0,071573248 0 NFLTTGIHEAAALDR ICPL (N-term)

gi|21224522 TRUE 6499 1767,888456 589,9676693 -0,209008 3 539,8840501 47,75 1 10,7 5,147570477 0,194266403 1 RSAFAATAAAQAAATAGR ICPL (N-term)

gi|21224522 TRUE 1968 1800,81468 600,9430773 -0,126232 3 179,0393181 41,91 1 19,5 8,953052603 0,111693748 0 AEAAYASGDTASALANGR ICPL (N-term)

gi|21224522 TRUE 2558 1892,923597 631,6460497 -0,000449 3 219,0383767 67,21 1 16,7 4,761795693 0,21000481 0 DEETATVDQLVAIVER ICPL (N-term)

gi|21224522 TRUE 5823 1897,969419 633,3279903 -0,211171 3 470,8065623 90,36 1 32,9 11,00490217 0,090868595 0 VEVFTILANASPEVAK ICPL (K); ICPL

(N-term)

gi|21224522 TRUE 7232 1897,969419 949,4883475 -0,147695 2 590,7697173 76,88 1 43,9 4,564383317 0,219087647 0 VEVFTILANASPEVAK ICPL (K); ICPL

(N-term)

gi|21224522 TRUE 5822 1897,969419 949,4883475 -0,109095 2 470,6671794 99,01 1 103,2 11,00490217 0,090868595 0 VEVFTILANASPEVAK ICPL (K); ICPL

(N-term)

gi|21224522 TRUE 4221 2029,986524 1015,4969 -0,118 2 348,7264272 146,19 1 138,5 18,80660529 0,053172807 0 ALAGSATDIDGFLAEGLAK ICPL (K); ICPL

(N-term)

gi|21224522 TRUE 2355 2096,988264 699,6676053 -0,122716 3 203,5726685 75,62 1 29,8 5,164255322 0,193638761 1 EAALTAAAEAEAAREDVAK ICPL (K); ICPL (N-term)

gi|21224522 TRUE 3930 2120,061811 707,3587877 0,020737 3 328,8492859 88,09 1 20,1 5,506546176 0,181602037 1 ARDEETATVDQLVAIVER ICPL (N-term)

gi|21224522 TRUE 3816 2165,004636 722,3397293 -0,031588 3 321,6856853 110,08 1 112 9,656719208 0,103554839 0 DAAQAALDDGSPSALHAF ICPL (N-term)

gi|21224522 TRUE 1098 2474,274549 825,4297003 -0,036001 3 103,1699749 79,46 1 24,1 6,37165325 0,156945138 0 AAQALAAAQTATQAAAAAGISAAATAR

ICPL (N-term)

gi|21224522 TRUE 7258 2681,218778 894,41111 -0,31773 3 592,1752751 105,41 1 8,7 5,345694383 0,187066437 0 GMATASAAALEAGEEAVAAFLDGGFK

ICPL (K); ICPL (N-term); Oxidation (M)

gi|21224522 TRUE 5833 2681,218778 894,41111 -0,05793 3 472,1509623 110,5 1 33,4 15,94424577 0,062718552 0 GMATASAAALEAGEEAVAAFLDGGFK

ICPL (K); ICPL (N-term); Oxidation (M)

gi|21224522 TRUE 7196 2918,416654 973,4770687 -0,199406 3 588,709617 95,41 1 34,8 8,695139139 0,115006785 0 NAAQTALSDGTSDALSVFLLDTQFTAR

ICPL (N-term)

gi|21224522 TRUE 2652 3634,673902 909,4239325 -0,25293 4 226,0019687 31,05 1 25 3,728875931 0,268177332 0 QDDSETALAVANVSTEAVANAAADAIENSDPQSVR ICPL (N-term)

TRUE nucleotidase

[S. coelicolor A3(2)] gi|21220497 1087,577904 29,06976744 32 18 0,65 0,05 18 1,69 0,13 115130699 1043,4 TRUE 2311 738,345731 738,345731 -0,033731 1 200,6252434 30,42 1 36,1 0,551700244 1,812578499 0 DGVPVF ICPL (N-term)

gi|21220497 TRUE 5120 976,557416 488,782346 -0,176892 2 429,4968786 44,43 1 11,8 0,734503364 1,361464153 0 LLTGAQLR ICPL (N-term)

gi|21220497 TRUE 5122 976,557416 488,782346 -0,164492 2 429,2358791 54,6 1 56,2 0,734503364 1,361464153 0 LLTGAQLR ICPL (N-term)

gi|21220497 TRUE 6639 976,557416 488,782346 -0,124892 2 549,5781919 48,21 1 20,1 0,714520654 1,399539669 0 LLTGAQLR ICPL (N-term)

gi|21220497 TRUE 6638 982,577558 491,792417 -0,231034 2 549,4440922 57,94 1 5,5 0,714520654 1,399539669 0 LLTGAQLR ICPL:13C(6) (N-term)

gi|21220497 TRUE 5124 982,577558 982,577558 -0,157758 1 429,5694957 42,11 1 34 0,734503364 1,361464153 0 LLTGAQLR ICPL:13C(6) (N-term)

gi|21220497 TRUE 3229 1029,605115 515,3061955 -0,163591 2 284,1300107 44,73 1 77,6 0 ISTLVNQVR

gi|21220497 TRUE 5217 1134,626569 567,8169225 -0,126245 2 435,1300455 38,74 1 106,8 0,633698578 1,578037312 0 ISTLVNQVR ICPL (N-term)

gi|21220497 TRUE 702 1140,646711 570,8269935 0,005013 2 76,10268267 30,58 2 16,8 0,394924773 2,532127806 0 ISTLVNQVR ICPL:13C(6) (N-term)

gi|21220497 TRUE 1964 1253,579694 627,293485 -0,09157 2 178,8611186 78,62 1 9,7 0,588605618 1,698930436 0 TAAGAPVDVDK ICPL (K); ICPL (N-term)

gi|21220497 TRUE 4226 1308,683408 654,845342 0,027516 2 349,0672608 73,67 1 18,4 1,082868288 0,923473345 0 DAPIIDLITK ICPL (K); ICPL (N-term)

gi|21220497 TRUE 2182 1354,627332 677,817304 -0,055008 2 192,7421762 59,34 1 48,5 0,666740268 1,499834415 0 ELWSESTEIR ICPL (N-term)

gi|21220497 TRUE 2180 1354,627332 677,817304 -0,053008 2 192,5303101 38,11 1 83,8 0,666740268 1,499834415 0 ELWSESTEIR ICPL (N-term)

gi|21220497 TRUE 2201 1360,647474 680,827375 -0,06115 2 193,9910349 56,7 1 3,5 0,275969873 3,623583942 0 ELWSESTEIR ICPL:13C(6) (N-term)

gi|21220497 TRUE 2013 1363,685209 682,3462425 -0,167485 2 182,4941266 52,7 1 38,5 0,760887629 1,314254512 0 TSEIPAGDVTIR ICPL (N-term)

gi|21220497 TRUE 2011 1363,685209 682,3462425 -0,164685 2 182,6200679 34,16 1 62,3 0,760887629 1,314254512 0 TSEIPAGDVTIR ICPL (N-term)

gi|21220497 TRUE 3777 1373,612043 687,3096595 -0,112519 2 319,489344 38,2 1 33,2 0,799682124 1,250496878 0 DFASADWSLTR ICPL (N-term)

gi|21220497 TRUE 3778 1373,612043 687,3096595 -0,098519 2 319,146952 68,64 1 29,3 0,799682124 1,250496878 0 DFASADWSLTR ICPL (N-term)

gi|21220497 TRUE 4053 1483,72157 742,364423 -0,091046 2 337,0063941 61,35 1 25 0,568926974 1,757694828 0 LAFPGLEEQAAK ICPL (K); ICPL (N-term)

gi|21220497 TRUE 4082 1495,761838 748,384557 -0,076914 2 339,0344944 48,09 1 25,7 0,695092325 1,438657808 0 LAFPGLEEQAAK ICPL:13C(6) (K); ICPL:13C(6) (N-term)

gi|21220497 TRUE 1849 1713,74629 857,376783 -0,157566 2 171,4377266 56,81 1 6,3 0,740223202 1,350943874 0 DAEYTDAAGNAQGLGR ICPL (N-term)

gi|21220497 TRUE 1851 1713,74629 857,376783 -0,116566 2 171,3687933 124,45 1 26,4 0,740223202 1,350943874 0 DAEYTDAAGNAQGLGR ICPL (N-term)

gi|21220497 TRUE 4230 1722,923551 861,9654135 -0,085827 2 349,2895275 75,23 1 51,3 0,362907418 2,755523726 1 YKDAPIIDLITK 2 ICPL:13C(6) (K); ICPL:13C(6) (N-term)

gi|21220497 TRUE 5826 1876,995588 939,001432 -0,181264 2 470,8605287 117,4 1 35,3 0,790716967 1,264675024 0 VAVLGLTNPGIAIWDK ICPL (K); ICPL

(N-term)

gi|21220497 TRUE 5595 2389,212949 1195,110113 -0,272225 2 457,1487373 99,47 1 15,9 0,568214194 1,759899718 0 EALAGTEYASLPVLAQASPFSR ICPL:13C(6) (N-term)

gi|21220497 TRUE 4211 2639,306669 880,440407 -0,134721 3 348,0854357 77,09 1 22,4 0,7860237 1,27222627 0 NTLLVDAGDTIQGTPLTYYYAK ICPL:13C(6) (K); ICPL:13C(6) (N-term)

TRUE succinyl-CoA synthetase subunit beta

[S. coelicolor A3(2)] gi|21223186 1004,785808 36,54822335 26 15 1,01 0,06 15 1,03 0,06 53023045 787,1 TRUE 3551 972,497632 486,752454 -0,108508 2 305,4638773 44,55 1 24,5 0,924273464 1,081930878 0 NLNYVK ICPL:13C(6) (K); ICPL:13C(6)

(N-term)

gi|21223186 TRUE 2291 1086,489072 543,748174 -0,119748 2 199,4373015 61,15 1 37,1 1,073918792 0,931169105 0 FPAEVADK ICPL (K); ICPL (N-term)

gi|21223186 TRUE 2287 1098,52934 549,768308 -0,132816 2 199,1813346 48,75 1 33,1 1,073918792 0,931169105 0 FPAEVADK ICPL:13C(6) (K); ICPL:13C(6)

(N-term)

gi|21223186 TRUE 5805 1132,546201 566,7767385 -0,034877 2 469,7267373 51,03 1 34,9 1,713136517 0,583724642 0 LWDTFIK ICPL (K); ICPL (N-term)

gi|21223186 TRUE 3622 1149,641492 383,885348 -0,144144 3 309,489344 34,1 1 18,4 0,641933944 1,557792682 0 VEKPLVVR ICPL (K); ICPL (N-term)

gi|21223186 TRUE 3620 1161,68176 387,8987707 -0,183212 3 309,4575941 38,93 1 10,4 0,641933944 1,557792682 0 VEKPLVVR ICPL:13C(6) (K); ICPL:13C(6)

(N-term)

gi|21223186 TRUE 1866 1163,543943 582,2756095 -0,118019 2 172,4139602 74,37 1 43,1 1,120747727 0,892261458 0 LDGNNAELGR ICPL (N-term)

gi|21223186 TRUE 1865 1163,543943 582,2756095 -0,099619 2 172,3478434 48,32 1 35,6 1,120747727 0,892261458 0 LDGNNAELGR ICPL (N-term)

gi|21223186 TRUE 3513 1174,6042 587,805738 -0,151276 2 302,8958027 63,14 1 40,3 0,963143779 1,038266582 0 AAELAHAAAK ICPL:13C(6) (K); ICPL:13C(6) (N-term)

gi|21223186 TRUE 1912 1178,579984 589,79363 -0,06186 2 175,4231101 92,07 1 48,6 0,981600462 1,018744426 0 LAASADEAVAR ICPL (N-term)

gi|21223186 TRUE 1909 1184,600126 592,803701 -0,157402 2 175,1526269 89,67 1 23,7 0,981600462 1,018744426 0 LAASADEAVAR ICPL:13C(6) (N-term)

gi|21223186 TRUE 2118 1248,640974 624,824125 -0,09385 2 188,4139517 94,59 1 29,9 0,79176719 1,262997524 0 AAANPLEAAAK ICPL:13C(6) (K); ICPL:13C(6) (N-term)

gi|21223186 TRUE 2069 1270,569837 635,7885565 -0,109513 2 185,4866013 64,81 1 17,1 1,309482822 0,763660266 0 VSLDDNAEFR ICPL (N-term)

gi|21223186 TRUE 2408 1382,695066 691,851171 -0,019942 2 207,5175517 78,12 1 45,7 0,953341819 1,048941712 0 VVSGDVIALDGK ICPL (K); ICPL

(N-term)

gi|21223186 TRUE 2423 1394,735334 697,871305 -0,00861 2 208,6578183 98,94 1 14,9 0,849448907 1,177233842 0 VVSGDVIALDGK ICPL:13C(6) (K); ICPL:13C(6) (N-term)

gi|21223186 TRUE 3504 1481,785917 741,3965965 -0,201393 2 302,5676603 61,53 1 39,5 0,876875946 1,14041217 0 ILTDANHPLVQR ICPL (N-term)

gi|21223186 TRUE 3505 1481,785917 494,6001563 -0,144169 3 302,5596773 31,95 1 18,7 0,876875946 1,14041217 0 ILTDANHPLVQR ICPL (N-term)

gi|21223186 TRUE 2354 1565,748212 783,377744 -0,126488 2 203,5594685 67,89 1 50,9 1,150413787 0,869252447 0 TPIDAIDGVTPEK ICPL (K); ICPL

(N-term)

gi|21223186 TRUE 2388 1565,748212 783,377744 -0,098888 2 206,3695431 63,71 1 24,2 1,019395908 0,980973135 0 TPIDAIDGVTICPL (K); ICPL (N-term)

gi|21223186 TRUE 2352 1577,78848 789,397878 -0,113356 2 203,6520434 71,13 1 45,7 1,150413787 0,869252447 0 TPIDAIDGVTPEK ICPL:13C(6)(K);ICPL:13C(6) (N-term)

gi|21223186 TRUE 2167 1993,99777 997,502523 -0,148446 2 191,6545101 70,05 1 32,7 0,869228536 1,150445433 0 HDVPVLAGEVIDTPEAAR ICPL (N-term)

gi|21223186 TRUE 2169 1993,99777 997,502523 -0,124046 2 191,7377186 123,52 1 21,2 0,869228536 1,150445433 0 HDVPVLAGEVIDTPEAAR ICPL (N-term)

gi|21223186 TRUE 2165 1993,99777 665,3374407 -0,107222 3 191,9100098 61,3 1 45,5 0,869228536 1,150445433 0 HDVPVLAGEVIDTPEAAR ICPL (N-term)

TRUE esterase

[S. coelicolor A3(2)] gi|21224437 987,0924484 32,5 29 16 1,38 0,1 16 0,79 0,06 119323838 1307,7 TRUE 3593 791,440992 396,224134 -0,143668 2 308,0747941 41,48 1 21,9 1,404903817 0,7117925 0 ADLLVR ICPL (N-term)

gi|21224437 TRUE 1637 948,453367 474,7303215 -0,144243 2 157,4492685 49,8 1 62,1 1,080630275 0,925385882 0 GDTGGPALR ICPL (N-term)

gi|21224437 TRUE 1636 954,473508 477,740392 -0,151784 2 157,0233762 68,81 1 22,9 1,080630275 0,925385882 0 GDTGGPALR ICPL:13C(6) (N-term)

gi|21224437 TRUE 2136 1084,505796 542,756536 -0,091272 2 189,6561602 48,68 1 12 2,172025575 0,460399735 0 FIDGSDLGR ICPL (N-term)

gi|21224437 TRUE 3424 1112,51193 556,759603 -0,095206 2 297,3313024 100,77 1 39,7 1,543056549 0,648064389 0 NLGDSFAAGR ICPL (N-term)

gi|21224437 TRUE 781 1187,678281 594,3427785 -0,099757 2 81,6715744 63,82 1 26,7 2,164189329 0,462066782 0 TLGIVEVVPR ICPL (N-term)

gi|21224437 TRUE 5307 1187,678281 594,3427785 -0,028357 2 440,3179954 73,04 1 65,8 0,968424368 1,032605161 0 TLGIVEVVPR ICPL (N-term)

gi|21224437 TRUE 5291 1187,678281 594,3427785 -0,027757 2 439,8571623 42,52 1 21 0,968424368 1,032605161 0 TLGIVEVVPR ICPL (N-term)

gi|21224437 TRUE 5310 1187,678281 1187,678281 -0,015281 1 440,4497287 45,35 1 50,5 0,968424368 1,032605161 0 TLGIVEVVPR ICPL (N-term)

gi|21224437 TRUE 5309 1193,698423 1193,698423 -0,076223 1 440,4615538 48,53 1 54,7 0,968424368 1,032605161 0 TLGIVEVVPR ICPL:13C(6) (N-term)

gi|21224437 TRUE 1971 1215,611646 608,309461 -0,064122 2 179,5480519 37,26 1 9,3 1,015975261 0,984275935 0 LHIGEGDTLR ICPL (N-term)

gi|21224437 TRUE 1973 1215,611646 608,309461 -0,055322 2 179,4769847 58,33 1 72,9 1,015975261 0,984275935 0 LHIGEGDTLR ICPL (N-term)

gi|21224437 TRUE 2361 1332,585508 666,796392 -0,025184 2 204,0972434 57,55 1 56,2 2,118311534 0,472074095 0 LYFADVDGDGR ICPL (N-term)

gi|21224437 TRUE 2130 1415,752897 708,3800865 -0,069173 2 189,376885 70,12 1 76,3 1,155579104 0,86536698 0 LAEPVTTVEPVR ICPL (N-term)

gi|21224437 TRUE 2129 1415,752897 708,3800865 -0,066173 2 189,5112183 68,81 1 91,8 1,155579104 0,86536698 0 LAEPVTTVEPVR ICPL (N-term)

gi|21224437 TRUE 3918 1422,664823 711,8360495 -0,161099 2 327,8428192 36,82 1 8 1,283745224 0,778970766 0 ADGLADWVTDVR ICPL (N-term)

gi|21224437 TRUE 3902 1422,664823 711,8360495 -0,100899 2 327,2639355 31,77 1 33,8 1,283745224 0,778970766 0 ADGLADWVTDVR ICPL (N-term)

gi|21224437 TRUE 3911 1422,664823 711,8360495 -0,051499 2 327,4861104 100,78 1 74,2 1,283745224 0,778970766 0 ADGLADWVTDVR ICPL (N-term)

gi|21224437 TRUE 3928 1422,664823 711,8360495 -0,031699 2 328,5157525 85,21 1 41,9 1,318743432 0,758297616 0 ADGLADWVTDVR ICPL (N-term)

gi|21224437 TRUE 1811 1487,723722 744,365499 -0,153398 2 168,921285 55,13 1 5,7 1,069920781 0,934648637 0 GTGGEVELAAVHSR ICPL (N-term)

gi|21224437 TRUE 1947 1619,65434 810,330808 -0,093816 2 177,8160679 95,17 1 44,3 1,154856614 0,865908363 0 SWQGGCFGETETR ICPL (N-term)

gi|21224437 TRUE 3675 1819,839826 910,423551 -0,163902 2 312,7667605 70,09 1 11,8 1,005651053 0,994380702 0 TEWVPDQLHTGEFR ICPL (N-term)

gi|21224437 TRUE 3677 1819,839826 910,423551 -0,120702 2 312,9200443 32,65 1 3,4 1,005651053 0,994380702 0 TEWVPDQLHTGEFR ICPL (N-term)

gi|21224437 TRUE 5709 1904,986463 952,9968695 -0,046139 2 464,0414461 48,14 1 246,8 0,949648299 1,05302142 0 LAADAPVAAETLLGAGFGR ICPL (N-term)

gi|21224437 TRUE 2560 2196,935667 1098,971472 -0,032743 2 219,2565351 105,4 1 52 1,705551775 0,586320518 0 FVDGSDLGSLEFGDATGDGK ICPL (K); ICPL (N-term)

TRUE secreted protein

[S. coelicolor A3(2)] gi|21218846 911,0338177 37,64705882 20 14 15,29 4,53 14 0,15 0,04 74420550 979,3 TRUE 692 866,419523 433,7133995 -0,149799 2 75,3981744 25,4 1 40,9 8,120946308 0,123138359 0 FYIYR ICPL (N-term)

gi|21218846 TRUE 1832 909,410108 455,208692 -0,170784 2 170,2535517 34,87 1 29,8 5,982519065 0,167153667 0 DVPGSPK ICPL (K); ICPL (N-term)

gi|21218846 TRUE 2003 1075,484312 538,245794 -0,047988 2 181,5978599 59,17 1 61,5 18,78209802 0,053242188 0 YADGEFIR ICPL (N-term)

gi|21218846 TRUE 3457 1285,497602 643,252439 -0,151478 2 299,4699269 56,82 1 15,4 2,94236028 0,339863207 0 YTGSMMGYDR ICPL (N-term)

gi|21218846 TRUE 2469 1313,600843 1313,600843 -0,135043 1 212,2781095 32,22 1 8,2 27,23614578 0,036715915 0 QDGSGTFDLIGV ICPL (N-term)

gi|21218846 TRUE 2085 1408,583753 704,7955145 -0,120029 2 186,4240269 74,09 1 38,8 7,53716209 0,132675931 0 AALDCSAEAYK ICPL (K); ICPL (N-term)

gi|21218846 TRUE 3359 1446,643033 723,8251545 -0,158109 2 293,043536 77,5 1 106,6 4,911723715 0,203594513 0 VTGTYGSLNCNR ICPL (N-term)

gi|21218846 TRUE 3745 1485,679059 743,3431675 -0,110135 2 316,9993355 95,2 1 63,6 28,50864536 0,035077079 0 AQALLDCGSAAAK ICPL (K); ICPL (N-term)

gi|21218846 TRUE 3780 1485,679059 743,3431675 -0,071335 2 319,3508021 32,14 1 16,8 3,970175295 0,251878047 0 AQALLDCGSAAAK ICPL (K); ICPL (N-term)

gi|21218846 TRUE 3785 1530,722333 765,8648045 -0,095209 2 319,6656944 83,35 1 46,8 22,60696749 0,04423415 0 TTEVSSAITHFK ICPL (K); ICPL (N-term)

gi|21218846 TRUE 1693 1591,761152 796,384214 -0,194228 2 161,0166183 80,5 1 33 6,45299636 0,15496677 0 AGAAAPTAQQKPDR ICPL (K); ICPL (N-term)

gi|21218846 TRUE 3399 1679,759458 840,383367 -0,168734 2 295,6194272 93,61 1 31 28,19807859 0,035463409 1 KVTGTYGSLNCNR ICPL (K); ICPL (N-term)

gi|21218846 TRUE 6732 1903,956159 952,4817175 -0,224435 2 556,423617 70,56 1 48,9 39,87847182 0,025076187 0 LRPSSIFSAHLDNWR ICPL (N-term)

gi|21218846 TRUE 5597 2464,088041 822,0341977 -0,350693 3 457,3201042 55,71 1 8 2,764198825 0,361768477 0 ESNSVTWNLTQPGYYGFYK ICPL (K); ICPL (N-term)

TRUE molecular chaperone DnaK

[S. coelicolor A3(2)] gi|32141213 862,6837121 28,3171521 17 14 2,13 0,14 14 0,5 0,03 48044733 516,4 TRUE 1635 1007,490476 504,248876 -0,167952 2 157,0396514 38,84 1 56,1 2,827637843 0,353652078 0 QAVTNVDR ICPL (N-term)

gi|32141213 TRUE 4088 1045,535306 523,271291 -0,078382 2 339,3229024 52,77 1 6,8 2,383921149 0,419476962 0 VVDYLVK ICPL (K); ICPL (N-term)

gi|32141213 TRUE 2084 1233,546094 617,276685 -0,03537 2 186,2651517 65,04 1 23 2,295110117 0,435708942 0 DAESYLGEK ICPL:13C(6) (K); ICPL:13C(6) (N-term)

gi|32141213 TRUE 3763 1346,706251 673,8567635 -0,080127 2 318,1325024 90,1 1 51,1 2,446857218 0,408687517 0 EAGEIAGLNVLR ICPL (N-term)

gi|32141213 TRUE 1978 1353,683591 677,3454335 -0,165667 2 179,7106429 41,53 1 8,7 1,327485716 0,753303774 1 LKGEDTAEIR ICPL:13C(6) (K); ICPL:13C(6) (N-term)

gi|32141213 TRUE 3500 1496,691709 499,5687537 -0,148661 3 302,1028192 41,57 1 35,1 2,497181263 0,400451507 0 QFQSGHGVDLAK ICPL (K); ICPL (N-term)

gi|32141213 TRUE 3499 1508,731977 754,8696265 -0,103653 2 302,0379776 54,9 1 27,2 2,497181263 0,400451507 0 QFQSGHGVDLAK ICPL:13C(6) (K); ICPL:13C(6) (N-term)

gi|32141213 TRUE 4018 1653,823087 827,4151815 -0,042963 2 334,1537355 95,13 1 50,2 1,96577485 0,508705257 0 AQFQQLTSDLLER ICPL (N-term)

gi|32141213 TRUE 1992 1658,784772 829,896024 -0,172248 2 180,7411597 65,71 1 18,8 1,162414786 0,860278114 0 NQGEQLVYQTEK ICPL:13C(6) (K); ICPL:13C(6) (N-term)

gi|32141213 TRUE 5775 1844,896604 922,95194 -0,09128 2 468,2487538 76,98 1 79,7 2,614538752 0,382476641 0 DFNPQQISAFVLQK ICPL (K); ICPL (N-term)

gi|32141213 TRUE 7191 1844,896604 922,95194 -0,05068 2 588,3595002 52,22 1 22,7 2,403786474 0,416010328 0 DFNPQQISAFVLQK ICPL (K); ICPL (N-term)

gi|32141213 TRUE 3969 1985,960325 662,6582923 -0,167077 3 331,4879189 34,64 1 11,6 1,874651409 0,533432506 0 VTDAVITVPAYFNDAER ICPL (N-term)

gi|32141213 TRUE 5802 2131,118223 1066,06275 -0,094299 2 469,8529538 151,09 1 53,3 1,891566795 0,528662272 0 GVNPDEVVAIGAALQAGVLK ICPL (K); ICPL (N-term)

gi|32141213 TRUE 1205 2131,118223 711,0442583 -0,076375 3 109,9423413 71,71 1 17 2,144000462 0,46641781 0 GVNPDEVVAIGAALQAGVLK ICPL (K); ICPL (N-term)

gi|32141213 TRUE 3867 2171,109128 724,37456 0,00852 3 324,8365275 56,79 1 19,7 1,907813878 0,524160146 0 DAGIQLSEIDHVVLVGGSTR ICPL (N-term)

TRUE secreted esterase

[S. coelicolor A3(2)] gi|21221495 848,2817218 24,64589235 18 12 1,21 0,07 12 0,86 0,05 74985216 704,9 TRUE 1833 1163,616727 582,3120015 -0,085603 2 170,3365767 40,58 1 45,7 1,199600984 0,83361052 1 TDRDLVLAR ICPL (N-term)

gi|21221495 TRUE 1810 1237,580717 619,2939965 -0,064993 2 168,9464599 67,75 1 63,7 1,03013062 0,97075068 0 AEDSLVSNAAR ICPL (N-term)

gi|21221495 TRUE 3610 1440,69061 480,9017207 -0,136562 3 308,9146608 34,7 1 14,1 1,079966178 0,925954924 1 TKEEWAPLTR ICPL (K); ICPL (N-term)

gi|21221495 TRUE 3604 1440,69061 720,848943 -0,132886 2 308,6686277 58,31 1 26,6 1,079966178 0,925954924 1 TKEEWAPLTR ICPL (K); ICPL (N-term)

gi|21221495 TRUE 3606 1452,730878 484,9151433 -0,12103 3 308,766152 26,52 1 18,7 1,079966178 0,925954924 1 TKEEWAPLTR ICPL:13C(6) (K); ICPL:13C(6) (N-term)

gi|21221495 TRUE 1919 1651,745908 826,376592 -0,166384 2 175,7531346 100,85 1 38,3 1,046424922 0,955634732 0 DAQGASQWAAGTEVR ICPL (N-term)

gi|21221495 TRUE 1918 1651,745908 826,376592 -0,158784 2 175,5227853 47,07 1 31,9 1,046424922 0,955634732 0 DAQGASQWAAGTEVR ICPL (N-term)

gi|21221495 TRUE 5009 1704,845243 852,9262595 -0,167319 2 422,8350706 61 1 73,5 0,801768303 1,24724312 0 TLWSTGTAGHLGATAR ICPL (N-term)

gi|21221495 TRUE 5010 1704,845243 852,9262595 -0,166119 2 422,7347954 110,96 1 52,6 0,801768303 1,24724312 0 TLWSTGTAGHLGATAR ICPL (N-term)

gi|21221495 TRUE 6547 1704,845243 568,953265 -0,130695 3 543,1119418 33,62 1 12,1 1,002543562 0,997462891 0 TLWSTGTAGHLGATAR ICPL (N-term)

gi|21221495 TRUE 832 1786,944608 893,975942 -0,220484 2 84,68804959 25,14 1 8,4 1,128310015 0,886281241 0 LDNIAGGNTLTPGAVLR ICPL (N-term)

gi|21221495 TRUE 5364 1786,944608 893,975942 -0,148884 2 443,5436791 117,87 1 126,9 1,36879721 0,730568409 0 LDNIAGGNTLTPGAVLR ICPL (N-term)

gi|21221495 TRUE 6850 1786,944608 893,975942 -0,141484 2 564,5572165 89,75 1 18,8 1,573820234 0,635396584 0 LDNIAGGNTLTPGAVLR ICPL (N-term)

gi|21221495 TRUE 2100 1821,807203 607,940585 -0,154455 3 187,414085 33,92 1 43,5 1,434929472 0,696898363 0 TDDDAICAGDAGGPLLR ICPL (N-term)

gi|21221495 TRUE 2105 1821,807203 911,4072395 -0,137879 2 187,5765682 105,97 1 33,7 1,434929472 0,696898363 0 TDDDAICAGDAGGPLLR ICPL (N-term)

gi|21221495 TRUE 5588 2185,186323 1093,0968 -0,241399 2 456,6545789 114,18 1 64,8 1,421830186 0,70331887 0 TTAVLGPAAGTTVEVVELVPR ICPL (N-term)

gi|21221495 TRUE 5681 3115,609907 1039,208153 -0,388959 3 462,3681207 74,8 1 12,9 1,41962896 0,704409411 0 LASPVAGTTPVPFATTAPAPGEELTVVGFGR ICPL (N-term)

TRUE chaperonin GroEL

[S. coelicolor A3(2)] gi|21222689 702,9183671 23,65988909 19 11 2,26 0,28 11 0,52 0,06 44968045 667,1 TRUE 1766 824,368574 412,687925 -0,12265 2 165,8093682 31,35 1 44,2 3,70570785 0,269853977 0 APGFGDR ICPL (N-term)

gi|21222689 TRUE 3829 939,493421 470,2503485 -0,011097 2 322,3318939 47,6 1 5,9 1,902251965 0,525692715 0 IGAELVK ICPL (K); ICPL (N-term)

gi|21222689 TRUE 4067 1064,577466 532,792371 -0,114742 2 338,0204272 53,43 1 28 1,477057371 0,677021773 0 LALEAPLK ICPL (K); ICPL (N-term)

gi|21222689 TRUE 2720 1150,614255 575,8107655 -0,038531 2 229,7844935 55,42 1 36,7 2,89132112 0,345862655 0 DLLPLLEK ICPL (K); ICPL (N-term)

gi|21222689 TRUE 2382 1474,63722 737,822248 -0,135096 2 205,5179597 47,86 1 17,6 1,332706212 0,750352922 0 EIELEDPYEK ICPL (K); ICPL (N-term)

gi|21222689 TRUE 4179 1609,822004 805,41464 -0,04688 2 345,5970859 63,9 1 47 2,625154216 0,380930002 0 AVEAVSAALLEQAK ICPL (K); ICPL (N-term)

gi|21222689 TRUE 2631 1609,822004 805,41464 0,05052 2 224,7508429 77,69 1 25,8 1,184549917 0,844202499 0 AVEAVSAALLEQAK ICPL (K); ICPL (N-term)

gi|21222689 TRUE 2303 1698,796934 849,902105 -0,18381 2 200,332093 96,39 1 31,6 2,223111757 0,449819941 0 LELTGDEATGANAVK ICPL (K); ICPL (N-term)

gi|21222689 TRUE 1893 1923,867902 962,437589 -0,180978 2 174,0470679 109,65 1 34,8 2,78123764 0,35955216 0 DETTIVDGAGSADQVQGR ICPL (N-term)

gi|21222689 TRUE 1892 1923,867902 641,960818 -0,180954 3 174,0768679 80,03 1 37,3 2,78123764 0,35955216 0 DETTIVDGAGSADQVQGR ICPL (N-term)

gi|21222689 TRUE 1204 2553,298368 851,7709733 -0,46222 3 110,0252165 98,29 1 39,3 3,37197517 0,296562089 0 AAVEEGIVAGGGVALLQASQVFEK ICPL (K); ICPL

(N-term)

gi|21222689 TRUE 5859 2553,298368 851,7709733 -0,38122 3 473,577629 52,26 1 2,4 1,264400406 0,790888705 0 AAVEEGIVAGGGVALLQASQVFEK ICPL (K); ICPL

(N-term)

gi|21222689 TRUE 1209 2553,298368 1277,152822 -0,198644 2 110,1458245 76,5 1 29,6 3,37197517 0,296562089 0 AAVEEGIVAGGGVALLQASQVFEK ICPL (K); ICPL

(N-term)

TRUE hypothetical protein SCO4584

[S. coelicolor A3(2)] gi|21222967 644,4496258 34,28571429 19 14 0,23 0,03 14 5,06 0,58 79193059 756,1 TRUE 2254 906,512235 906,512235 -0,159535 1 197,3501682 25,28 1 2,6 0,327722214 3,051364717 0 LVVEPK ICPL:13C(6) (K); ICPL:13C(6) (N-term)

gi|21222967 TRUE 2246 906,512235 453,7597555 -0,115711 2 196,6281431 35,48 1 39,5 0,327722214 3,051364717 0 LVVEPK ICPL:13C(6) (K); ICPL:13C(6) (N-term)

gi|21222967 TRUE 3549 1030,539518 515,773397 -0,141794 2 305,5298277 58,41 1 13,4 0,190538568 5,248281293 0 VYAGAVTK I CPL:13C(6) (K); ICPL:13C(6) (N-term)

gi|21222967 TRUE 2183 1129,535154 565,271215 -0,07163 2 192,5559181 45,98 1 36 0,267098222 3,743941059 0 DFADQALK ICPL:13C(6) (K); ICPL:13C(6) (N-term)

gi|21222967 TRUE 3773 1236,572309 618,7897925 -0,103985 2 319,0735275 34,87 1 4,3 0,257834337 3,878459367 0 GDFGSGTVFK ICPL:13C(6) (K); ICPL:13C(6) (N-term)

gi|21222967 TRUE 2347 1252,661069 626,8341725 -0,002945 2 203,1409602 69,55 1 32,6 0,217962371 4,587947899 0 VTAEQLIEK ICPL:13C(6) (K); ICPL:13C(6) (N-term)

gi|21222967 TRUE 1858 1300,65875 650,833013 -0,110426 2 172,2631431 40,47 1 25,2 0,16079125 6,219243911 0 SDVQQLSGTVR ICPL:13C(6) (N-term)

gi|21222967 TRUE 4714 1312,721937 656,8646065 -0,123013 2 402,4830205 33,6 1 13,8 0 LTELVSGSHTLR

gi|21222967 TRUE 4066 1380,719663 690,8634695 -0,010139 2 338,0734021 85,52 1 36,7 0,237525323 4,210077424 0 LVNALITDDGK ICPL:13C(6) (K); ICPL:13C(6) (N-term)

gi|21222967 TRUE 5172 1417,743391 709,3753335 -0,043467 2 432,1915127 73,54 1 69,1 0,145567231 6,869677953 0 LTELVSGSHTLR ICPL (N-term)

gi|21222967 TRUE 5173 1423,763532 475,2593613 -0,154984 3 432,2992706 58,47 1 48,5 0,145567231 6,869677953 0 LTELVSGSHTLR ICPL:13C(6) (N-term)

gi|21222967 TRUE 5169 1423,763532 712,385404 -0,128208 2 432,4164125 62,04 1 161,5 0,145567231 6,869677953 0 LTELVSGSHTLR ICPL:13C(6) (N-term)

gi|21222967 TRUE 6684 1423,763532 712,385404 -0,064808 2 553,2232752 73,02 1 90,4 0,103267609 9,683578533 0 LTELVSGSHTLR ICPL:13C(6) (N-term)

gi|21222967 TRUE 669 1423,763532 712,385404 -0,052808 2 73,65158293 30,62 1 34,3 0,106420865 9,396653592 0 LTELVSGSHTLR ICPL:13C(6) (N-term)

gi|21222967 TRUE 1979 1445,709852 723,358564 -0,168728 2 180,1135597 52,71 1 25,4 0,346496877 2,886028899 0 DVPGQDVPATPK ICPL:13C(6) (K); ICPL:13C(6) (N-term)

gi|21222967 TRUE 1131 2060,052671 1030,529974 -0,068147 2 105,8706832 36,16 1 12,1 0,443363943 2,255483372 0 FTLTPASGGAAVVDVGFTK ICPL:13C(6) (K); ICPL:13C(6) (N-term)

gi|21222967 TRUE 1769 2182,982679 728,3324103 -0,248131 3 166,1779101 67,79 1 61,4 0,198070458 5,048708476 0 SNEVYHSTAADGAERPEK ICPL:13C(6) (K); ICPL:13C(6) (N-term)

gi|21222967 TRUE 4104 2445,153211 815,7225877 -0,210563 3 340,7557355 60,02 1 32,1 0,258814771 3,863767106 0 VSFAKPDASTFDFTPPEGAK 2 ICPL:13C(6) (K); ICPL:13C(6) (N-term)

TRUE secreted protein

[S. coelicolor A3(2)] gi|21220594 592,717904 11,55885472 12 8 1,03 0,12 8 1,08 0,13 19857282 419,9 TRUE 3445 1001,525861 501,2665685 -0,121537 2 298,6134187 44,47 1 9,3 0,9534932 1,048775177 0 GAQLFAQR ICPL:13C(6) (N-term)

gi|21220594 TRUE 3307 1168,574537 584,7909065 -0,120013 2 289,7107771 67,6 1 89,9 1,137188872 0,879361401 0 AGASSVPVYGR ICPL (N-term)

gi|21220594 TRUE 3306 1168,574537 584,7909065 -0,120013 2 289,698144 39,83 1 117,9 1,137188872 0,879361401 0 AGASSVPVYGR ICPL (N-term)

gi|21220594 TRUE 1815 1250,533704 625,77049 -0,04698 2 169,1566765 59,16 1 14,7 0,987891394 1,012257022 0 NEAWHAANK ICPL (K); ICPL (N-term)

gi|21220594 TRUE 4183 1406,616407 703,8118415 -0,096083 2 346,0096939 34,76 1 22,3 0,51092051 1,957251627 0 DWTAIWYGGK ICPL (K); ICPL (N-term)

gi|21220594 TRUE 2218 1444,752622 722,879949 -0,078298 2 194,7976765 71,11 1 14,9 1,481348713 0,675060498 0 VLAGGEYVVAEAR ICPL:13C(6) (N-term)

gi|21220594 TRUE 5399 1562,717512 781,862394 -0,089188 2 445,5947623 46,53 1 28,7 1,053154201 0,949528568 1 RDWTAIWYGGK ICPL (K); ICPL (N-term)

gi|21220594 TRUE 2193 2150,070417 717,3616563 -0,121969 3 193,2574599 36,78 1 22,4 0,720545377 1,387837647 0 EHILGHDEVPGVLDGNVK ICPL:13C(6) (K); ICPL:13C(6) (N-term)

gi|21220594 TRUE 2366 2253,017347 751,6772997 -0,185399 3 204,9064354 68,08 1 17,9 1,37790016 0,725741987 0 DGSWYTEPQYESSAALVK ICPL:13C(6) (K); ICPL:13C(6) (N-term)

TRUE SLPI=protease inhibitor [S. lividans, 66,

Peptide, 107 aa] gi|257239 533,82 99,06542056 38 13 21,13 5,6 13 0,09 0,02 406309668 3064,6 TRUE 6899 1105,494901 553,2510885 -0,234577 2 567,9434672 51,6 1 6,7 9,194561021 0,10875995 0 NAGSASVFTF ICPL (N-term)

gi|257239 TRUE 6870 1105,494901 553,2510885 -0,156377 2 566,0566586 89,24 1 16,9 30,97781735 0,032281164 0 NAGSASVFTF ICPL (N-term)

gi|257239 TRUE 4035 1105,494901 553,2510885 -0,146177 2 335,6368107 64,68 1 28,5 6,521856101 0,153330583 0 NAGSASVFTF ICPL (N-term)

gi|257239 TRUE 3899 1105,494901 553,2510885 -0,136577 2 326,8378277 49,61 1 10,9 40,03657705 0,02497716 0 NAGSASVFTF ICPL (N-term)

gi|257239 TRUE 3908 1105,494901 1105,494901 -0,122801 1 327,2526939 53,29 1 79,7 40,03657705 0,02497716 0 NAGSASVFTF ICPL (N-term)

gi|257239 TRUE 3921 1105,494901 553,2510885 -0,120577 2 328,1488027 91,02 1 128,3 40,03657705 0,02497716 0 NAGSASVFTF ICPL (N-term)

gi|257239 TRUE 3926 1105,494901 1105,494901 -0,117801 1 328,3933605 50,37 1 97,1 40,03657705 0,02497716 0 NAGSASVFTF ICPL (N-term)

gi|257239 TRUE 3996 1879,933744 940,47051 -0,15042 2 332,7690939 108,54 1 167,9 29,49598869 0,033902915 0 EYAPVVVTVDGVWQGR ICPL (N-term)

gi|257239 TRUE 937 1879,933744 940,47051 -0,12862 2 91,36120799 77,52 1 26,8 5,16762877 0,193512352 0 EYAPVVVTVDGVWQGR ICPL (N-term)

gi|257239 TRUE 3977 1879,933744 940,47051 -0,12562 2 331,6118107 103,76 1 92,1 24,5679396 0,040703454 0 EYAPVVVTVDGVWQGR ICPL (N-term)

gi|257239 TRUE 2454 1879,933744 940,47051 -0,11702 2 211,4498429 111,28 1 42,2 12,14785773 0,082319041 0 EYAPVVVTVDGVWQGR ICPL (N-term)

gi|257239 TRUE 1944 2063,890928 1032,449102 -0,233404 2 177,4026599 121,78 1 98,6 31,61144112 0,031634116 0 AAHGDPSALAAEDSVMCTR ICPL (N-term)

gi|257239 TRUE 1813 2079,885847 1040,446562 -0,300523 2 169,2158685 81,31 1 36,6 44,24654497 0,022600635 0 AAHGDPSALAAEDSVMCTR ICPL (N-term); Oxidation (M)

gi|257239 TRUE 5283 2357,22479 786,413114 -0,180942 3 439,0173623 60,84 1 36,2 10,39101732 0,096236968 0 YAPSALVLTVGHGESAATAAPLR ICPL (N-term)

gi|257239 TRUE 6534 2573,223493 858,4126817 -0,337145 3 542,2365919 41,76 1 51,4 15,16946742 0,065921892 0 AVTLTCAPTASGTHPAAAAACAELR ICPL (N-term)

gi|257239 TRUE 2030 2573,223493 858,4126817 -0,232445 3 183,4226434 45,3 1 16,5 3,325231371 0,300730953 0 AVTLTCAPTASGTHPAAAAACAELR ICPL (N-term)

TRUE secreted protein

[S. coelicolor A3(2)] gi|21224521 517,38 31,11111111 18 9 9,36 0,96 9 0,12 0,01 103479339 950,4 TRUE 2082 934,394117 467,7006965 -0,126393 2 186,3032765 44,77 1 57,3 8,457733036 0,118234992 0 SFDVEK ICPL (K); ICPL (N-term)

gi|21224521 TRUE 2087 934,394117 934,394117 -0,123317 1 186,5367266 38,87 1 4,8 8,457733036 0,118234992 0 SFDVEK ICPL (K); ICPL (N-term)

gi|21224521 TRUE 2107 934,394117 467,7006965 -0,117393 2 187,7627767 41,57 1 19,1 6,677044905 0,149766853 0 SFDVEK ICPL (K); ICPL (N-term)

gi|21224521 TRUE 2060 934,394117 467,7006965 -0,113593 2 185,0710183 39,43 1 73,2 12,0890367 0,082719577 0 SFDVEK ICPL (K); ICPL (N-term)

gi|21224521 TRUE 3990 1081,56764 541,287458 -0,078116 2 332,4193941 71,36 1 31,1 10,418608 0,095982112 0 DFALLEIR ICPL (N-term)

gi|21224521 TRUE 3991 1081,56764 1081,56764 -0,05024 1 332,5028272 41,6 1 13,1 10,418608 0,095982112 0 DFALLEIR ICPL (N-term)

gi|21224521 TRUE 1969 1394,615766 465,5434393 -0,118318 3 179,6303682 55,11 1 70,3 8,787010009 0,113804354 0 GDGHIVLADCK ICPL (K); ICPL (N-term)

gi|21224521 TRUE 1965 1394,615766 697,811521 -0,104842 2 179,0642935 50,12 1 93,3 8,787010009 0,113804354 0 GDGHIVLADCK ICPL (K); ICPL (N-term)

gi|21224521 TRUE 1853 1550,716871 775,8620735 -0,177747 2 171,5475853 88,33 1 84,9 10,22645717 0,097785576 1 RGDGHIVLADCK ICPL (K); ICPL (N-term)

gi|21224521 TRUE 1852 1550,716871 517,577141 -0,177723 3 171,5309767 62,08 1 106,1 10,22645717 0,097785576 1 RGDGHIVLADCK ICPL (K); ICPL (N-term)

gi|21224521 TRUE 2266 1751,798337 876,4028065 -0,128613 2 197,8506098 102,98 1 98,7 11,70245179 0,085452179 0 NLWTAVGQTADEEGR ICPL (N-term)

gi|21224521 TRUE 2284 1751,798337 876,4028065 -0,094813 2 199,0201933 109,7 1 118,6 10,95241158 0,091304093 0 NLWTAVGQTADEEGR ICPL (N-term)

gi|21224521 TRUE 2285 1751,798337 584,6042963 -0,094789 3 199,0324685 64,64 1 51,8 10,95241158 0,091304093 0 NLWTAVGQTADEEGR ICPL (N-term)

gi|21224521 TRUE 4127 2709,326688 903,7804133 -0,10744 3 342,2037525 90,86 1 41 4,448624898 0,224788563 1 NLWTAVGQTADEEGRDFALLEIR ICPL (N-term)

TRUE tellurium resistance protein

[S. coelicolor A3(2)] gi|21222670 515,09 45,02617801 16 11 2,05 0,15 11 0,52 0,04 151283619 1245,6 TRUE 1724 741,367811 371,1875435 -0,139287 2 162,8969602 28,38 1 29,7 2,701718555 0,370134779 0 NAYIR ICPL (N-term)

gi|21222670 TRUE 1552 935,469358 468,238317 -0,152634 2 151,2384103 31,6 1 241,6 0 SQNFGQVR

gi|21222670 TRUE 1697 1040,490812 520,749044 -0,134088 2 161,5639431 50,86 1 4,2 2,182136699 0,458266432 0 SQNFGQVR ICPL (N-term)

gi|21222670 TRUE 3522 1317,690931 659,3491035 -0,120607 2 303,566336 92,39 1 80,7 2,405438148 0,415724678 0 ILNQAGGAEIAR ICPL (N-term)

gi|21222670 TRUE 7025 1627,81146 814,409368 -0,111336 2 576,9773173 114 1 114,4 1,674780958 0,597093008 0 IVFPVSIYDAENR ICPL (N-term)

gi|21222670 TRUE 5586 1627,81146 543,2753373 -0,079312 3 456,5797458 62,93 1 15,7 1,298423902 0,770164504 0 IVFPVSIYDAENR ICPL (N-term)

gi|21222670 TRUE 5583 1627,81146 814,409368 -0,055336 2 456,4949703 109,98 1 418,9 1,298423902 0,770164504 0 IVFPVSIYDAENR ICPL (N-term)

gi|21222670 TRUE 1017 1627,81146 814,409368 0,041464 2 96,84701599 66,17 1 38,6 1,660951567 0,602064515 0 IVFPVSIYDAENR ICPL (N-term)

gi|21222670 TRUE 5582 1633,831602 817,419439 -0,116678 2 456,2154375 96 1 55,1 1,298423902 0,770164504 0 IVFPVSIYDAENR ICPL:13C(6) (N-term)

gi|21222670 TRUE 1677 1672,767393 836,8873345 -0,187869 2 159,8079762 84,21 1 77,9 1,894156915 0,527939366 0 QTPDNTIVHTGDNR ICPL (N-term)

gi|21222670 TRUE 6911 1797,801984 599,9388453 -0,155536 3 568,9319754 39,05 1 10,3 1,454288263 0,687621585 0 VYSDAHFVFFNNK ICPL (K); ICPL (N-term)

gi|21222670 TRUE 5441 1797,801984 899,40463 -0,12026 2 448,1272039 57,35 1 51,2 1,955158118 0,511467584 0 VYSDAHFVFFNNK ICPL (K); ICPL (N-term)

gi|21222670 TRUE 5779 2129,029828 1065,018552 -0,121704 2 468,4314455 87,78 1 32,4 2,976424177 0,335973618 0 AVGQGYASGLTGIAQDFGVNV ICPL (N-term)

gi|21222670 TRUE 7192 2129,029828 710,3481267 -0,10178 3 588,4720586 72,71 1 35 2,311973041 0,432530995 0 AVGQGYASGLTGIAQDFGVNV ICPL (N-term)

TRUE dihydrolipoamide dehydrogenase

[S. coelicolor A3(2)] gi|21220654 506,8807266 16,25514403 11 6 2,24 0,31 6 0,5 0,07 28055469 360,8 TRUE 5799 1311,61569 656,311483 -0,112566 2 469,4653709 59,75 1 67,5 3,827694431 0,261253874 0 FNLGTFFQK ICPL (K); ICPL (N-term)

gi|21220654 TRUE 5801 1311,61569 1311,61569 -0,09389 1 469,5706455 44,52 1 33 3,827694431 0,261253874 0 FNLGTFFQK ICPL (K); ICPL (N-term)

gi|21220654 TRUE 2017 1322,637556 661,822416 -0,087032 2 182,9963266 71,97 1 44,9 2,508634037 0,398623309 0 TVPVDYDGVPR ICPL (N-term)

gi|21220654 TRUE 2077 1375,66406 688,335668 -0,149136 2 186,0326685 58,63 1 26,4 1,282090116 0,779976374 1 KITYIEGEGR ICPL (K); ICPL (N-term)

gi|21220654 TRUE 3566 1445,738294 482,584282 -0,150546 3 306,4432107 56,12 1 15,7 2,120751737 0,471530912 0 IISSDHALTLDR ICPL (N-term)

gi|21220654 TRUE 3561 1445,738294 723,372785 -0,06537 2 306,2093355 92,07 1 38 2,120751737 0,471530912 0 IISSDHALTLDR ICPL (N-term)

gi|21220654 TRUE 1932 1471,728788 736,368032 -0,172064 2 176,7489597 84,12 1 41,3 1,943865779 0,514438811 0 ALLHAGEVADQSR ICPL (N-term)

gi|21220654 TRUE 1937 1471,728788 491,24778 -0,14934 3 176,699477 58,29 1 13,7 1,943865779 0,514438811 0 ALLHAGEVADQSR ICPL (N-term)

gi|21220654 TRUE 2566 1575,768949 788,3881125 0,004375 2 219,8837682 78,57 1 29,9 1,777413879 0,562615163 0 SFGSEVTVIEGLK ICPL (K); ICPL (N-term)

TRUE secreted esterase

[S. coelicolor A3(2)] gi|21224523 494,1738177 10,90174966 10 7 5,54 0,91 7 0,21 0,04 28596241 444,9 TRUE 1691 778,420606 389,713941 -0,115082 2 160,8654434 50,2 1 49,7 4,747642377 0,210630861 0 TGGIVAR ICPL (N-term)

gi|21224523 TRUE 1694 778,420606 778,420606 -0,072106 1 161,0687181 36,97 1 28,9 4,747642377 0,210630861 0 TGGIVAR ICPL (N-term)

gi|21224523 TRUE 3697 916,525068 458,766172 -0,124744 2 313,9382939 44,68 1 35,4 7,477729822 0,133730427 0 VVELVPR ICPL (N-term)

gi|21224523 TRUE 1797 1122,553815 561,7805455 -0,142091 2 167,9922018 89,19 1 68,4 6,887750278 0,145185287 0 ADLTGTGGAVR ICPL (N-term)

gi|21224523 TRUE 1740 1149,601087 383,8718797 -0,177839 3 164,2231266 38,43 1 6,7 2,245623642 0,445310595 1 TDRDVVLAR ICPL (N-term)

gi|21224523 TRUE 1736 1149,601087 575,3041815 -0,147963 2 163,9149186 40,28 1 93,8 2,245623642 0,445310595 1 TDRDVVLAR ICPL (N-term)

gi|21224523 TRUE 3727 1149,641492 575,324384 -0,049968 2 315,7200107 62,49 1 33,6 7,547677156 0,132491094 1 KVVELVPR ICPL (K); ICPL (N-term)

gi|21224523 TRUE 1898 1752,728208 876,867742 -0,200884 2 174,4594935 125,36 1 62,3 5,808540033 0,172160301 0 SYQGGCYGIDAAETR ICPL (N-term)

gi|21224523 TRUE 5536 3242,716764 1081,577105 -0,409216 3 453,5708039 87,02 1 33 3,628894685 0,275566002 0 LNRPVTNVTPLALATAAPTAGEELTLAGYGR

ICPL (N-term)

TRUE secreted hydrolase

[S. coelicolor A3(2)] gi|21224438 481,0469089 22,28796844 11 9 1,4 0,11 9 0,76 0,06 47228947 297 TRUE 3712 825,425352 413,216314 -0,090228 2 314,9837691 29,98 1 1,3 1,052052853 0,950522587 0 LDFAVR ICPL (N-term)

gi|21224438 TRUE 3565 1043,515623 522,2614495 -0,114499 2 306,4672277 51,41 1 27,5 1,211935655 0,825126314 0 LTIGTDYR ICPL (N-term)

gi|21224438 TRUE 1765 1213,559614 607,283445 -0,04249 2 165,7798098 56,18 1 27 1,401557552 0,713491928 0 ADSGVANGYVR ICPL (N-term)

gi|21224438 TRUE 1903 1317,654584 659,33093 -0,07446 2 174,7393597 65,84 1 42,3 1,494709645 0,669026258 0 DGAPQLVGVNSR ICPL (N-term)

gi|21224438 TRUE 1901 1323,674726 662,341001 -0,013202 2 174,5727517 66,82 1 24,4 1,494709645 0,669026258 0 DGAPQLVGVNSR ICPL:13C(6) (N-term)

gi|21224438 TRUE 3563 1393,722257 697,3647665 -0,114733 2 306,3344107 73,66 1 62,8 0,903889534 1,10632988 0 EVVELVPHPNR ICPL (N-term)

gi|21224438 TRUE 3562 1393,722257 697,3647665 -0,031133 2 306,4909104 26 1 10,4 0,903889534 1,10632988 0 EVVELVPHPNR ICPL (N-term)

gi|21224438 TRUE 3543 1467,701536 734,354406 -0,160212 2 304,7902357 33,48 1 33,4 1,977315462 0,505736196 1 TKDEWVPVQR ICPL (K); ICPL (N-term)

gi|21224438 TRUE 5666 2702,430067 901,4815397 -0,149519 3 461,602821 70,67 1 19,5 1,421005223 0,703727181 0 AVPDIAPVPLAAGAPDPGAPLTAVGFGR ICPL (N-term)

gi|21224438 TRUE 3722 2725,179487 909,0646797 -0,223139 3 315,587736 101,37 1 29,5 1,268882825 0,788094835 0 STQGGCFGSDATSTAAVAAASDADFVR ICPL (N-term)

gi|21224438 TRUE 2226 2725,179487 909,0646797 -0,182639 3 195,3409597 83,38 1 18,9 1,851643322 0,540060814 0 STQGGCFGSDATSTAAVAAASDADFVR ICPL (N-term)

TRUE phosphomannomutase

[S. coelicolor A3(2)] gi|21223290 480,4169089 14,38979964 10 7 1,59 0,16 7 0,67 0,07 33051825 219,4 TRUE 5457 1082,562894 541,785085 -0,07837 2 449,0184541 87,77 1 28,2 1,574273527 0,635213629 0 DTLLAAFAR ICPL (N-term)

gi|21223290 TRUE 5460 1082,562894 1082,562894 -0,070694 1 449,1265458 49,74 1 22,4 1,574273527 0,635213629 0 DTLLAAFAR ICPL (N-term)

gi|21223290 TRUE 5458 1088,583036 1088,583036 -0,063936 1 449,0300791 52,65 1 35,5 1,574273527 0,635213629 0 DTLLAAFAR ICPL:13C(6) (N-term)

gi|21223290 TRUE 4150 1155,604429 578,3058525 -0,074105 2 343,5082192 65,21 1 12,7 1,076552006 0,928891493 0 ATDLLTALK ICPL (K); ICPL (N-term)

gi|21223290 TRUE 5505 1158,594175 579,8007255 -0,065451 2 452,1513293 89,16 1 14,5 1,327876468 0,753082101 0 AAAGLAAYLK ICPL (K); ICPL (N-term)

gi|21223290 TRUE 5511 1170,634443 585,8208595 -0,063119 2 452,0766455 55,98 1 18,9 1,327876468 0,753082101 0 AAAGLAAYLK ICPL:13C(6) (K); ICPL:13C(6) (N-term)

gi|21223290 TRUE 3423 1390,751646 695,879461 -0,201722 2 297,1310272 43,34 1 15,4 2,428698552 0,411743153 0 VVVRPSGTEPK ICPL:13C(6) (K); ICPL:13C(6) (N-term)

gi|21223290 TRUE 2159 1464,638975 732,8231255 -0,019651 2 191,2186098 28,68 2 17,4 1,921541656 0,520415468 0 AWLAEDPDADTR ICPL (N-term)

gi|21223290 TRUE 5533 1530,773282 765,890279 0,032042 2 453,3981959 57,99 1 19,6 1,571308985 0,636412068 0 VEDLSLIAAAMHR ICPL (N-term)

gi|21223290 TRUE 5325 1656,870389 828,9388325 0,017135 2 441,2823458 110,79 1 34,8 1,217248848 0,821524705 0 GDEVGALLATHLVTR ICPL (N-term)

TRUE hypothetical protein SCO2368

[S. coelicolor A3(2)] gi|21220836 465,67 43,45549738 17 9 1,17 0,09 9 0,91 0,07 143260807 1197,2 TRUE 3365 725,372907 363,1900915 -0,123183 2 293,5010944 32,27 1 92,6 1,02241879 0,978072792 0 NAFIR ICPL (N-term)

gi|21220836 TRUE 3363 731,393049 731,393049 -0,080549 1 293,3398773 29,2 1 82,9 1,02241879 0,978072792 0 NAFIR ICPL:13C(6) (N-term)

gi|21220836 TRUE 1719 1054,506467 527,7568715 -0,120543 2 162,668885 58,06 1 47,3 0,943424855 1,059967834 0 QQSFGQVR ICPL (N-term)

gi|21220836 TRUE 1718 1054,506467 527,7568715 -0,113343 2 162,9575517 34,01 1 62,1 0,943424855 1,059967834 0 QQSFGQVR ICPL (N-term)

gi|21220836 TRUE 3435 1197,601072 599,304174 -0,107948 2 298,4639525 89,31 1 116,9 1,116377373 0,89575445 0 AIGQGYASGLR ICPL (N-term)

gi|21220836 TRUE 3433 1197,601072 599,304174 -0,062948 2 297,9080773 78,86 1 5,3 1,116377373 0,89575445 0 AIGQGYASGLR ICPL (N-term)

gi|21220836 TRUE 1880 1361,680768 681,344022 -0,097044 2 173,3233847 86,85 1 52,3 0,836294464 1,195751069 0 VVNQAGEAEIAR ICPL (N-term)

gi|21220836 TRUE 1876 1361,680768 681,344022 -0,058044 2 173,0553095 55,09 1 4,1 0,836294464 1,195751069 0 VVNQAGEAEIAR ICPL (N-term)

gi|21220836 TRUE 7025 1627,81146 814,409368 -0,111336 2 576,9773173 114 1 114,4 1,674780958 0,597093008 0 IVFPVSIYDAENR ICPL (N-term)

gi|21220836 TRUE 5586 1627,81146 543,2753373 -0,079312 3 456,5797458 62,93 1 15,7 1,298423902 0,770164504 0 IVFPVSIYDAENR ICPL (N-term)

gi|21220836 TRUE 5583 1627,81146 814,409368 -0,055336 2 456,4949703 109,98 1 418,9 1,298423902 0,770164504 0 IVFPVSIYDAENR ICPL (N-term)

gi|21220836 TRUE 1017 1627,81146 814,409368 0,041464 2 96,84701599 66,17 1 38,6 1,660951567 0,602064515 0 IVFPVSIYDAENR ICPL (N-term)

gi|21220836 TRUE 5582 1633,831602 817,419439 -0,116678 2 456,2154375 96 1 55,1 1,298423902 0,770164504 0 IVFPVSIYDAENR ICPL:13C(6) (N-term)

gi|21220836 TRUE 5587 1844,915739 922,9615075 -0,194615 2 456,4590626 57,21 1 26,8 1,009207514 0,990876491 0 VASDAHFIFFNNLK ICPL:13C(6) (K); ICPL:13C(6) (N-term)

gi|21220836 TRUE 2607 2408,069395 803,3613157 0,104353 3 223,3791181 27,97 1 16,8 0,954489579 1,047680375 0 YDLSEDASTETAMVFGELYR ICPL:13C(6) (N-term)

TRUE tellurium resistance protein

[S. coelicolor A3(2)] gi|32141118 459,7969089 47,64397906 21 9 0,87 0,04 9 1,17 0,06 60485151 948,9 TRUE 3365 725,372907 363,1900915 -0,123183 2 293,5010944 32,27 1 92,6 1,02241879 0,978072792 0 NAFIR ICPL (N-term)

gi|32141118 TRUE 3363 731,393049 731,393049 -0,080549 1 293,3398773 29,2 1 82,9 1,02241879 0,978072792 0 NAFIR ICPL:13C(6) (N-term)

gi|32141118 TRUE 1708 983,469358 492,238317 -0,189034 2 162,0703346 64,76 1 78,4 1,002645349 0,99736163 0 GQSFGQVR ICPL (N-term)

gi|32141118 TRUE 1707 983,469358 492,238317 -0,141034 2 162,2365351 29,09 1 81 1,002645349 0,99736163 0 GQSFGQVR ICPL (N-term)

gi|32141118 TRUE 1955 1281,716123 641,3616995 -0,078399 2 178,4450018 49 1 33 0 VNLVAVPAEVDR

gi|32141118 TRUE 1530 1355,702573 678,3549245 -0,158249 2 149,7233186 42,33 1 44,8 0 VVNQANNQELAR

gi|32141118 TRUE 3788 1386,737577 693,8724265 -0,035053 2 320,2076523 63,97 1 88,1 1,001203462 0,998797985 0 VNLVAVPAEVDR ICPL (N-term)

gi|32141118 TRUE 1780 1460,724027 730,8656515 -0,120903 2 167,2323346 70,56 1 80 0,716838427 1,3950145 0 VVNQANNQELAR ICPL (N-term)

gi|32141118 TRUE 1776 1460,724027 730,8656515 -0,104503 2 167,0320098 39,65 1 114,4 0,716838427 1,3950145 0 VVNQANNQELAR ICPL (N-term)

gi|32141118 TRUE 3787 1601,80705 801,407163 -0,155326 2 319,8687691 76,35 1 8,5 0,768878445 1,300595701 0 IVFPVSIHDAENR ICPL (N-term)

gi|32141118 TRUE 2280 1601,80705 801,407163 -0,070926 2 198,6671517 71,01 1 15,7 0,841117855 1,188894034 0 IVFPVSIHDAENR ICPL (N-term)

gi|32141118 TRUE 2282 1601,80705 801,407163 -0,049726 2 198,7591767 74,94 1 21,8 0,841117855 1,188894034 0 IVFPVSIHDAENR ICPL (N-term)

gi|32141118 TRUE 3774 1607,827192 804,417234 -0,126668 2 318,7632939 106,09 1 6,1 0,768878445 1,300595701 0 IVFPVSIHDAENR ICPL:13C(6) (N-term)

gi|32141118 TRUE 4081 2368,0827 790,0324173 -0,145252 3 338,957344 74,8 1 37,5 0,84762656 1,179764824 0 TTTGTDYDLDASALLLDTSGK ICPL (K); ICPL (N-term)

gi|32141118 TRUE 2544 2368,0827 790,0324173 -0,135352 3 218,0802263 48,98 1 22,1 0,689810231 1,449674063 0 TTTGTDYDLDASALLLDTSGK ICPL (K); ICPL (N-term)

gi|32141118 TRUE 4086 2380,122968 1190,565122 -0,324844 2 339,1865024 96,7 1 19,9 0,84762656 1,179764824 0 TTTGTDYDLDASALLLDTSGK ICPL:13C(6) (K); ICPL:13C(6) (N-term)

gi|32141118 TRUE 2542 2380,122968 794,04584 -0,25332 3 218,2743597 42,56 1 27,7 0,689810231 1,449674063 0 TTTGTDYDLDASALLLDTSGK ICPL:13C(6) (K); ICPL:13C(6) (N-term)

gi|32141118 TRUE 4079 2380,122968 794,04584 -0,17532 3 338,8341189 48,27 1 27,4 0,84762656 1,179764824 0 TTTGTDYDLDASALLLDTSGK ICPL:13C(6) (K); ICPL:13C(6) (N-term)

gi|32141118 TRUE 2607 2408,069395 803,3613157 0,104353 3 223,3791181 27,97 1 16,8 0,954489579 1,047680375 0 YDLSEDASTETAMVFGELYR ICPL:13C(6) (N-term)

TRUE transcriptional regulator

[S. coelicolor A3(2)] gi|21221840 435,2809952 32,80632411 13 8 1,33 0,15 8 0,82 0,09 61497422 553,6 TRUE 3630 1047,558134 524,282705 -0,07421 2 310,1016939 70,14 1 136,6 1,715648906 0,582869838 0 DAILANAVR ICPL (N-term)

gi|21221840 TRUE 3631 1053,578275 1053,578275 -0,112475 1 310,1273771 33,06 1 17,4 1,715648906 0,582869838 0 DAILANAVR ICPL:13C(6) (N-term)

gi|21221840 TRUE 1902 1087,459165 544,2332205 -0,131641 2 174,7273431 54,84 1 100 1,348299625 0,741674908 0 WGNTDAYR ICPL (N-term)

gi|21221840 TRUE 1929 1450,69206 725,849668 -0,109736 2 176,2463186 51,6 1 50,6 1,012309074 0,987840597 1 IQDEADELTRR ICPL (N-term)

gi|21221840 TRUE 6775 1700,821287 567,6119463 -0,197239 3 559,720609 77,33 1 32,3 0,93346584 1,071276481 0 NIDAAKPGLAAYMR ICPL (K); ICPL (N-term)

gi|21221840 TRUE 5278 1700,821287 567,6119463 -0,166039 3 438,6659543 77,3 1 54,4 1,244701417 0,803405529 0 NIDAAKPGLAAYMR ICPL (K); ICPL (N-term)

gi|21221840 TRUE 6770 1712,861555 571,625369 -0,157707 3 559,386825 59,2 1 40,7 0,93346584 1,071276481 0 NIDAAKPGLAAYMR ICPL:13C(6) (K); ICPL:13C(6) (N-term)

gi|21221840 TRUE 5277 1712,861555 571,625369 -0,116607 3 438,6547959 34,21 1 24 1,244701417 0,803405529 0 NIDAAKPGLAAYMR ICPL:13C(6) (K); ICPL:13C(6) (N-term)

gi|21221840 TRUE 5095 1716,816205 572,9435857 -0,221957 3 427,7058045 67,01 1 12,3 0,837576923 1,193920192 0 NIDAAKPGLAAYMR ICPL (K); ICPL (N-term); Oxidation (M)

gi|21221840 TRUE 2588 2225,929822 1113,468549 -0,261298 2 221,8514263 84,28 1 23,7 2,21979339 0,450492377 0 FEVFGDFDPDQYEEEVR ICPL (N-term)

gi|21221840 TRUE 2392 2610,08711 870,700554 -0,249162 3 206,3297015 92,47 1 16,2 1,371520146 0,729117981 0 FVALMDAGEPADSEGAMDAAEDHR ICPL (N-term)

TRUE secreted protein

[S. coelicolor A3(2)] gi|21220348 431,8969089 20,29850746 10 7 7,45 2,1 7 0,21 0,06 26214133 455,3 TRUE 1599 1026,485044 513,74616 -0,15832 2 154,4979682 83,67 1 48,2 2,960667056 0,337761721 0 SEGSTTLAR ICPL (N-term)

gi|21220348 TRUE 3820 1198,610227 1198,610227 -0,144527 1 321,7954773 67,42 1 10,2 17,05393858 0,058637481 0 TAAATALELK ICPL (K); ICPL (N-term)

gi|21220348 TRUE 3813 1198,610227 599,8087515 -0,075503 2 321,5285024 84,88 1 58,1 17,05393858 0,058637481 0 TAAATALELK ICPL (K); ICPL (N-term)

gi|21220348 TRUE 3840 1198,610227 599,8087515 -0,031103 2 323,5120939 65,64 1 27,7 4,528053498 0,220845447 0 TAAATALELK ICPL (K); ICPL (N-term)

gi|21220348 TRUE 2058 1488,696485 744,8518805 -0,190161 2 184,9487181 60,26 1 21,3 2,137821077 0,467765993 1 LDLSKEETTSR ICPL (K); ICPL (N-term)

gi|21220348 TRUE 7096 1902,011976 951,509626 -0,194252 2 582,1976586 89,5 1 59,7 6,97217155 0,143427337 0 QPVADANLLGPVTVLGK ICPL (K); ICPL (N-term)

gi|21220348 TRUE 1088 1902,011976 951,509626 -0,081052 2 102,4899579 56,47 1 27,3 9,382730427 0,106578784 0 QPVADANLLGPVTVLGK ICPL (K); ICPL (N-term)

gi|21220348 TRUE 3915 2231,130262 744,3816047 0,020986 3 327,6200192 59,82 1 29,6 8,554903801 0,116892022 0 VSVNPGDLNVAEVDGTVTLAR ICPL (N-term)

TRUE secreted 5'-nucleotidase

[S. coelicolor A3(2)] gi|21222550 423,4932856 14,54248366 9 7 1,31 0,12 7 0,81 0,07 16319444 230,6 TRUE 2290 1433,621931 717,3146035 -0,077807 2 199,4230269 64,09 1 24,5 1,471193725 0,679720137 0 FEDEVETINK ICPL (K); ICPL (N-term)

gi|21222550 TRUE 3624 1467,722639 734,3649575 -0,039915 2 309,618144 27,11 1 26,7 1,107199718 0,903179421 0 LNGAAIDPTATYR ICPL (N-term)

gi|21222550 TRUE 4099 1558,790006 779,898641 -0,105682 2 340,1736773 67,93 1 43,8 1,096267348 0,912186249 0 DYTGAQLIQVLK ICPL (K); ICPL (N-term)

gi|21222550 TRUE 4100 1570,830274 785,918775 -0,13295 2 340,2792853 82,53 1 27,1 1,096267348 0,912186249 0 DYTGAQLIQVLK ICPL:13C(6) (K); ICPL:13C(6)

(N-term)

gi|21222550 TRUE 833 1720,865293 574,2932817 -0,103445 3 84,81790773 36,44 1 2 1,857118665 0,538468553 0 TIDAGGVEYLATHLR ICPL (N-term)

gi|21222550 TRUE 6843 1726,885435 576,2999957 0,150013 3 564,1221418 62,1 1 23,8 1,315349948 0,760253955 0 TIDAGGVEYLATHLR ICPL:13C(6) (N-term)

gi|21222550 TRUE 2497 1848,84387 924,925573 -0,032346 2 214,3706429 109,39 1 32,8 1,471893087 0,679397171 0 GADYPYLAANVLDEK ICPL (K); ICPL (N-term)

gi|21222550 TRUE 2496 1860,884138 930,945707 -0,038414 2 214,2368765 95,48 1 36 1,471893087 0,679397171 0 GADYPYLAANVLDEK ICPL:13C(6) (K); ICPL:13C(6) (N-term)

gi|21222550 TRUE 3850 2462,121885 821,3788123 -0,138337 3 324,0876357 95,7 1 13,9 0,858874016 1,164315117 0 VASNSFLAGGGDGFTTLGEGTNER ICPL (N-term)

TRUE isocitrate dehydrogenase

[S. coelicolor A3(2)] gi|21225286 417,4853816 11,23139378 8 6 2,23 0,14 6 0,46 0,03 18774179 227,9 TRUE 1998 1236,62187 618,814573 -0,044546 2 181,2084519 56,21 1 21,5 1,70323308 0,587118705 0 SAVIAEDGTLR ICPL (N-term)

gi|21225286 TRUE 3645 1326,668851 663,8380635 -0,081727 2 310,8612693 32,17 1 26,4 2,394327007 0,417653895 0 TFEIPTTGTVR ICPL (N-term)

gi|21225286 TRUE 5188 1443,77432 722,390798 -0,205996 2 433,3146375 84,97 1 41,7 2,56919978 0,389226252 0 VSDPIIFGHVVR ICPL (N-term)

gi|21225286 TRUE 2411 1638,754697 819,8809865 -0,092373 2 207,6821762 70,82 1 45,1 2,190206122 0,456578032 0 ATGDPAVFWLDEGR ICPL (N-term)

gi|21225286 TRUE 1108 2251,135328 1126,071302 -0,206804 2 104,0251909 95,72 1 34,6 2,607631897 0,38348971 0 LVDAAGNAVLEQTVSAGDIFR ICPL (N-term)

gi|21225286 TRUE 5698 2251,135328 751,04996 0,01962 3 463,6636455 83,7 1 45,5 1,908368693 0,524007758 0 LVDAAGNAVLEQTVSAGDIFR ICPL (N-term)

TRUE hypothetical protein SCO3767

[S. coelicolor A3(2)] gi|21222178 411,8069089 47,01986755 13 9 2,04 0,19 9 0,53 0,05 28556860 448,8 TRUE 4207 1180,63606 590,821668 -0,087936 2 348,1009941 69,99 1 78 2,572649446 0,388704338 0 VGILQEIAK ICPL (K); ICPL (N-term)

gi|21222178 TRUE 4194 1180,63606 1180,63606 -0,08756 1 347,3753861 48,63 1 40,1 3,44071386 0,290637362 0 VGILQEIAK ICPL (K); ICPL (N-term)

gi|21222178 TRUE 4208 1192,676328 596,841802 -0,147204 2 347,9000357 69,11 1 29,1 2,572649446 0,388704338 0 VGILQEIAK ICPL:13C(6) (K); ICPL:13C(6) (N-term)

gi|21222178 TRUE 4189 1192,676328 596,841802 -0,141804 2 346,6911104 66,87 1 89 2,252739257 0,443904015 0 VGILQEIAK ICPL:13C(6) (K); ICPL:13C(6) (N-term)

gi|21222178 TRUE 4195 1192,676328 1192,676328 -0,090928 1 347,3022944 43,26 1 38,5 2,572649446 0,388704338 0 VGILQEIAK ICPL:13C(6) (K); ICPL:13C(6) (N-term)

gi|21222178 TRUE 2649 1192,676328 596,841802 0,050196 2 225,7367767 63,32 1 24,3 1,77329417 0,563922229 0 VGILQEIAK ICPL:13C(6) (K); ICPL:13C(6) (N-term)

gi|21222178 TRUE 1960 1260,564344 630,78581 -0,05562 2 178,6402434 53,54 1 37,5 1,853206184 0,539605365 1 RFEENLDK ICPL (K); ICPL (N-term)

gi|21222178 TRUE 2485 1894,831602 947,919439 -0,161078 2 213,5888183 50 1 21 1,52221729 0,656936435 0 EACYALDLPPHEFDL ICPL (N-term)

gi|21222178 TRUE 5680 2348,188077 1174,597677 0,094447 2 462,3217874 104,58 1 30,1 2,074918623 0,481946612 0 VAQLISTNEVLQNFPADDLR ICPL (N-term)

gi|21222178 TRUE 5522 2504,289182 626,8277525 -0,31701 4 452,952429 52,07 1 14,8 1,392152401 0,718312161 1 VAQLISTNEVLQNFPADDLRR ICPL (N-term)

gi|21222178 TRUE 979 2504,289182 835,434578 -0,137634 3 93,71986667 63,88 1 6,9 1,490940394 0,670717625 1 VAQLISTNEVLQNFPADDLRR ICPL (N-term)

TRUE secreted protein

[S. coelicolor A3(2)] gi|21220483 388,8119903 14,23785595 12 7 1,17 0,08 7 0,88 0,06 18180826 310,1 TRUE 4065 979,524717 490,2659965 -0,128993 2 337,7227691 31,12 1 14,4 1,143714914 0,874343761 0 GLLELPK ICPL (K); ICPL (N-term)

gi|21220483 TRUE 4037 979,524717 490,2659965 -0,091793 2 335,7882192 26,25 2 16,6 1,62985658 0,613550917 0 GLLELPK ICPL (K); ICPL (N-term)

gi|21220483 TRUE 4075 991,564985 496,2861305 -0,164461 2 338,4963109 39,22 1 20 1,242380384 0,804906462 0 GLLELPK ICPL:13C(6) (K); ICPL:13C(6) (N-term)

gi|21220483 TRUE 4059 991,564985 496,2861305 -0,012061 2 337,5797771 32,26 1 26 1,143714914 0,874343761 0 GLLELPK ICPL:13C(6) (K); ICPL:13C(6) (N-term)

gi|21220483 TRUE 3753 1114,560651 557,7839635 -0,159927 2 317,5850688 50,91 1 30,3 1,011771511 0,988365445 0 TVPGLDYK ICPL:13C(6) (K); ICPL:13C(6) (N-term)

gi|21220483 TRUE 2316 1604,759077 802,8831765 -0,121953 2 200,9118354 77,07 1 29,8 0,892419854 1,120548804 0 LYADDAEGTYILR ICPL (N-term)

gi|21220483 TRUE 2315 1604,759077 802,8831765 -0,015553 2 201,0454269 42,71 1 90,8 0,892419854 1,120548804 0 LYADDAEGTYILR ICPL (N-term)

gi|21220483 TRUE 2164 1741,792844 581,269132 0,005904 3 191,3911847 54,79 1 13,6 1,32002366 0,757562179 1 NSWDLADQADLKR ICPL (K); ICPL (N-term)

gi|21220483 TRUE 2511 3102,388471 1034,801008 0,564277 3 215,5773933 46,78 1 29 0,987128229 1,013039613 0 VADALTSTGETEPTCTPGGEFADPIEVKI CPL (K);ICPL (N-term)

TRUE co-chaperonin GroES

[S. coelicolor A3(2)] gi|21223140 374,91 72,54901961 17 8 2,31 0,2 8 0,46 0,04 35961193 558 TRUE 1862 963,420667 482,2139715 -0,146943 2 172,2986183 37,33 1 33,1 2,931161677 0,341161666 0 YGGTEVK ICPL (K); ICPL (N-term)

gi|21223140 TRUE 4142 1096,567319 548,7872975 -0,115995 2 343,2352773 59,17 1 55,3 2,925190436 0,341858085 0 DVLAIVEK ICPL (K); ICPL (N-term)

gi|21223140 TRUE 4177 1096,567319 1096,567319 -0,115019 1 345,6281109 56,4 1 31,1 2,450867584 0,408018779 0 DVLAIVEK ICPL (K); ICPL (N-term)

gi|21223140 TRUE 4162 1096,567319 1096,567319 -0,113519 1 344,4729275 56,54 1 33,7 2,450867584 0,408018779 0 DVLAIVEK ICPL (K); ICPL (N-term)

gi|21223140 TRUE 4181 1096,567319 548,7872975 -0,108395 2 345,7242688 59,55 1 48,1 2,450867584 0,408018779 0 DVLAIVEK ICPL (K); ICPL (N-term)

gi|21223140 TRUE 4143 1096,567319 1096,567319 -0,096319 1 343,2515024 53,15 1 32,1 2,925190436 0,341858085 0 DVLAIVEK ICPL (K); ICPL (N-term)

gi|21223140 TRUE 4166 1108,607587 1108,607587 -0,145587 1 344,7616107 56,03 1 2,8 2,450867584 0,408018779 0 DVLAIVEK ICPL:13C(6) (K); ICPL:13C(6) (N-term)

gi|21223140 TRUE 4144 1108,607587 554,8074315 -0,131263 2 343,4806608 56,02 1 23,6 2,925190436 0,341858085 0 DVLAIVEK ICPL:13C(6) (K); ICPL:13C(6) (N-term)

gi|21223140 TRUE 4169 1108,607587 554,8074315 -0,064263 2 345,4439856 55,63 1 9,3 2,450867584 0,408018779 0 DVLAIVEK ICPL:13C(6) (K); ICPL:13C(6) (N-term)

gi|21223140 TRUE 3658 1250,652769 417,5557737 -0,190121 3 311,586544 35,24 1 17,5 2,844959069 0,351498906 0 VAIKPLEDR ICPL (K); ICPL (N-term)

gi|21223140 TRUE 3735 1518,722288 759,864782 -0,008564 2 316,5094272 80,02 1 18,4 2,038680569 0,490513333 0 YNGEEYLVLSAR ICPL (N-term)

gi|21223140 TRUE 3600 1745,89694 873,452108 -0,125816 2 308,4716773 58,33 1 17,8 1,505819906 0,664090039 0 EKPQEGVVLAVGPGR ICPL (K); ICPL (N-term)

gi|21223140 TRUE 3596 1745,89694 582,637164 -0,109692 3 308,3806523 71,51 1 41,5 1,505819906 0,664090039 0 EKPQEGVVLAVGPGR ICPL (K); ICPL (N-term)

gi|21223140 TRUE 2598 2576,324262 859,4462713 -0,122314 3 222,4883266 81,71 1 19,2 1,664503519 0,600779745 0 IVVQPLDAEQTTASGLVIPDTAK ICPL (K); ICPL (N-term)

gi|21223140 TRUE 4147 2576,324262 859,4462713 -0,071914 3 343,5368773 91,26 1 99,9 2,101968865 0,47574444 0 IVVQPLDAEQTTASGLVIPDTAK ICPL (K); ICPL (N-term)

gi|21223140 TRUE 4145 2588,36453 863,459694 -0,047682 3 343,6209189 56,71 1 49,6 2,101968865 0,47574444 0 IVVQPLDAEQTTASGLVIPDTAK ICPL:13C(6) (K); ICPL:13C(6) (N-term)

TRUE type II citrate synthase

[S. coelicolor A3(2)] gi|21221189 363,8209952 13,28671329 8 5 0,68 0,02 5 1,48 0,05 23463383 276,1 TRUE 4841 938,430128 469,718702 -0,182604 2 411,6941207 30,41 1 33,6 0,75046874 1,332500538 0 LMGFGHR ICPL (N-term); Oxidation (M)

gi|21221189 TRUE 5649 1332,702527 666,8549015 -0,076203 2 460,6735874 70,99 1 39,5 0,682528168 1,465140997 0 LPTIAAYAYK ICPL:13C(6) (K); ICPL:13C(6) (N-term)

gi|21221189 TRUE 5365 1362,680051 681,8436635 -0,042927 2 443,6719874 83,9 1 43,5 0,597821747 1,672739415 0 AAAHDVLSALGK ICPL (K); ICPL (N-term)

gi|21221189 TRUE 5363 1374,720319 687,8637975 -0,052995 2 443,3639042 65,27 1 61,5 0,597821747 1,672739415 0 AAAHDVLSALGK ICPL:13C(6) (K); ICPL:13C(6) (N-term)

gi|21221189 TRUE 3937 1589,826505 795,4168905 -0,135981 2 329,0078357 96,6 1 28,6 0,676819958 1,477497801 0 SAITYLDGEAGILR ICPL:13C(6) (N-term)

gi|21221189 TRUE 2171 1785,807828 595,9407933 -0,15508 3 192,1502599 39,44 1 26,9 0,695177425 1,438481694 0 LEEHALSDDYFVSR ICPL (N-term)

gi|21221189 TRUE 2179 1785,807828 893,407552 -0,060304 2 192,2378765 68,64 1 13,9 0,695177425 1,438481694 0 LEEHALSDDYFVSR ICPL (N-term)

gi|21221189 TRUE 2176 1791,82797 896,417623 -0,061646 2 192,0859933 85,92 1 28,6 0,695177425 1,438481694 0 LEEHALSDDYFVSR ICPL:13C(6) (N-term)

TRUE secreted esterase

[S. coelicolor A3(2)] gi|21224890 362,14 8,843537415 8 6 1,69 0,14 6 0,61 0,05 17508233 308,1 TRUE 3258 893,48393 447,245603 -0,221206 2 286,6150192 66,64 1 49 1,531096172 0,653126837 0 ATTAVIGR ICPL (N-term)

gi|21224890 TRUE 3619 916,525068 458,766172 -0,130944 2 309,6028192 41,22 1 33,1 1,580667968 0,632643933 0 VVEIVPR ICPL (N-term)

gi|21224890 TRUE 3408 1072,626172 536,816724 -0,128248 2 296,2642523 56,35 1 55,2 1,220947729 0,819035882 1 RVVEIVPR ICPL (N-term)

gi|21224890 TRUE 1740 1149,601087 383,8718797 -0,177839 3 164,2231266 38,43 1 6,7 2,245623642 0,445310595 1 TDRDVVLAR ICPL (N-term)

gi|21224890 TRUE 1736 1149,601087 575,3041815 -0,147963 2 163,9149186 40,28 1 93,8 2,245623642 0,445310595 1 TDRDVVLAR ICPL (N-term)

gi|21224890 TRUE 4214 1512,704404 756,85584 -0,04228 2 348,2889605 79,89 1 30,3 1,980454644 0,504934563 0 VDDLASWIDEK ICPL:13C(6) (K); ICPL:13C(6) (N-term)

gi|21224890 TRUE 6905 3070,489866 1024,168139 -0,348418 3 568,4789002 74,3 1 4,6 1,61051047 0,620921142 0 LVGLNSQSFQGGCLGVDETQTSTAGVIAR ICPL (N-term)

TRUE secreted tripeptidylaminopeptidase

[S. coelicolor A3(2)] gi|21219739 338,2469089 15,34195933 8 6 0,31 0,03 6 3,41 0,29 192712802 16,2 TRUE 2016 841,450971 421,2291235 -0,223047 2 182,6381767 34,54 1 9,2 0,345691983 2,892748601 0 DLDVIR ICPL:13C(6) (N-term)

gi|21219739 TRUE 5350 1412,789609 706,8984425 -0,077085 2 442,6792541 65,24 1 20,6 0,232566619 4,299843218 0 GLPPVLIVQSER ICPL (N-term)

gi|21219739 TRUE 6831 1418,809751 709,9085135 -0,137027 2 563,4147088 68,25 1 21,9 0,353663801 2,827544114 0 GLPPVLIVQSER ICPL:13C(6) (N-term)

gi|21219739 TRUE 5343 1418,809751 709,9085135 -0,096627 2 442,3328375 67,67 1 13,1 0,232566619 4,299843218 0 GLPPVLIVQSER ICPL:13C(6) (N-term)

gi|21219739 TRUE 1721 1432,673353 716,8403145 -0,159229 2 162,8430434 60,96 1 43,3 0,384111922 2,603407868 0 DVTCAPHATPRP ICPL:13C(6) (N-term)

gi|21219739 TRUE 3581 1724,881025 862,9441505 -0,159501 2 307,4753355 98,55 1 23,4 0,287835114 3,474211284 0 QGALIYNPGGPGGSGLR ICPL:13C(6) (N-term)

gi|21219739 TRUE 3437 1992,9288 664,9811173 -0,165352 3 298,1976272 27,73 1 56,8 0,229215638 4,362704078 0 DAGSHGVTGLVNPCINDR ICPL:13C(6) (N-term)

TRUE GroEL1

[S. coelicolor] gi|406598 322,442717 13,88888889 9 6 3,34 0,46 6 0,33 0,05 9918523 200,3 TRUE 1766 824,368574 412,687925 -0,12265 2 165,8093682 31,35 1 44,2 3,70570785 0,269853977 0 APGFGDR ICPL (N-term)

gi|406598 TRUE 3644 1116,547269 558,7772725 -0,047545 2 311,2075024 32,99 1 18,3 2,969806342 0,336722293 0 GTFNAVAVK ICPL (K); ICPL (N-term)

gi|406598 TRUE 5852 1633,92381 817,465543 -0,086686 2 473,2409042 79,6 1 33,3 4,669453793 0,21415781 0 ISSIADLLPLLEK ICPL:13C(6) (K); ICPL:13C(6) (N-term)

gi|406598 TRUE 3838 1679,770002 840,388639 -0,115478 2 322,8184773 77,94 1 19,1 4,389189025 0,227832521 0 GYLSPYFVTDQER ICPL (N-term)

gi|406598 TRUE 3857 1679,770002 840,388639 -0,053278 2 324,1932027 45,01 1 22,5 1,832475355 0,545709931 0 GYLSPYFVTDQER ICPL (N-term)

gi|406598 TRUE 5492 2193,114606 731,7097193 -0,221158 3 451,2799789 48,27 1 23,9 2,440250597 0,409793978 0 TNDIAGDGTTTATVLAQALVR ICPL (N-term)

TRUE 4-aminobutyrate aminotransferase

[S. coelicolor A3(2)] gi|21224026 321,5169089 9,684684685 4 3 0,36 0,06 3 3,02 0,51 11153792 116,6 TRUE 5311 1379,77371 690,390493 -0,096986 2 440,5578461 87,94 1 46,9 0,416048999 2,403563046 0 GIAGGLPLSAVTGR ICPL:13C(6) (N-term)

gi|21224026 TRUE 6947 1877,027982 939,017629 -0,128658 2 571,342617 126,6 1 13 0,428979062 2,331116104 0 TAVVAQGVGSTLPVFVAR ICPL (N-term)

gi|21224026 TRUE 6719 2039,149229 680,387927 -0,199281 3 555,4711754 72,23 1 47 0,229625289 4,354921028 1 RTAVVAQGVGSTLPVFVAR ICPL:13C(6) (N-term)

TRUE elongation factor Ts

[S. coelicolor A3(2)] gi|21223980 315,062559 21,94244604 8 5 0,44 0,05 5 2,42 0,27 22738425 282,4 TRUE 5194 1419,716779 710,3620275 -0,087855 2 433,726221 29,96 1 21 0,483155947 2,069725121 0 GVAQHIAAFAPK ICPL (K); ICPL (N-term)

gi|21223980 TRUE 5184 1431,757047 716,3821615 -0,166723 2 433,0244375 60,46 1 42,7 0,483155947 2,069725121 0 GVAQHIAAFAPK ICPL:13C(6) (K); ICPL:13C(6) (N-term)

gi|21223980 TRUE 5577 1708,84416 854,925718 -0,047236 2 456,0153959 106,9 1 42 0,380693988 2,626781699 0 FQNVATAIAEHVAK ICPL (K); ICPL (N-term)

gi|21223980 TRUE 5572 1720,884427 860,9458515 -0,069103 2 455,8365122 135,5 1 95,8 0,380693988 2,626781699 0 FQNVATAIAEHVAK ICPL:13C(6) (K); ICPL:13C(6) (N-term)

gi|21223980 TRUE 4235 2004,991239 1002,999258 -0,140115 2 349,608752 52,87 1 22,1 0,500894889 1,996426839 0 AAPADLEALLASEIEPGK ICPL (K); ICPL (N-term)

gi|21223980 TRUE 2709 2017,031507 673,015353 0,034041 3 229,2055351 71,79 1 17 0,553052765 1,808145739 0 AAPADLEALLASEIEPGK ICPL:13C(6) (K); ICPL:13C(6) (N-term)

gi|21223980 TRUE 2187 2228,102064 743,3722053 -0,220516 3 192,9748765 57,17 1 16 0,276181403 3,620808605 2 YLSKEDVPAEVVESERR ICPL:13C(6) (K); ICPL:13C(6) (N-term)

TRUE triosephosphate isomerase

[S. coelicolor A3(2)] gi|21220430 300,9617218 19,37984496 5 4 0,92 0,12 4 1,16 0,16 7667642 92,5 TRUE 4123 1589,748167 795,3777215 0,006157 2 341,7716272 45,97 1 34,6 1,003698989 0,996314643 0 LAELYSQELADK ICPL (K); ICPL (N-term)

gi|21220430 TRUE 4121 1601,788434 801,397855 0,00169 2 341,7716939 85,37 1 20,8 1,003698989 0,996314643 0 LAELYSQELADK ICPL:13C(6) (K); ICPL:13C(6) (N-term)

gi|21220430 TRUE 3903 1617,790754 809,399015 -0,10023 2 326,8073691 69,32 1 14 0,924082325 1,082154666 1 SVQTLVDGDKLK 2 ICPL (K); ICPL (N-term)

gi|21220430 TRUE 4062 1856,95796 928,982618 -0,073836 2 337,4877024 85,35 1 12 0,580661005 1,722175232 1 LAELYSQELADKVR ICPL:13C(6) (K); ICPL:13C(6) (N-term)

gi|21220430 TRUE 2722 2727,330045 909,7815323 -0,236497 3 229,9963015 72,49 1 11,1 1,176133347 0,850243727 0 DLAAEQAESVVIAYEPVWAIGTGK ICPL (K); ICPL

(N-term)

TRUE succinyl-CoA synthetase subunit alpha

[S. coelicolor A3(2)] gi|21223187 280,51 18,36734694 11 6 0,97 0,16 6 1,21 0,21 15571114 277,6 TRUE 5566 954,483182 477,745229 -0,173058 2 455,5477373 27,79 1 28,7 1,593438785 0,627573528 0 AIWLNK ICPL (K); ICPL (N-term)

gi|21223187 TRUE 5514 954,483182 477,745229 -0,146658 2 452,5466205 28,02 1 29,9 1,202320574 0,831724934 0 AIWLNK ICPL (K); ICPL (N-term)

gi|21223187 TRUE 5513 966,52345 483,765363 -0,114126 2 452,6673207 25,3 1 28,2 1,202320574 0,831724934 0 AIWLNK ICPL:13C(6) (K); ICPL:13C(6) (N-term)

gi|21223187 TRUE 5575 966,52345 483,765363 -0,113326 2 455,807229 26,79 1 9,2 1,593438785 0,627573528 0 AIWLNK ICPL:13C(6) (K); ICPL:13C(6) (N-term)

gi|21223187 TRUE 5519 966,52345 966,52345 -0,11115 1 452,5015037 26,89 1 13,3 1,202320574 0,831724934 0 AIWLNK ICPL:13C(6) (K); ICPL:13C(6) (N-term)

gi|21223187 TRUE 2135 1109,566434 555,286855 -0,10711 2 189,3365517 46,91 1 15,2 1,021645583 0,978813022 0 EALEAAGVK ICPL:13C(6) (K); ICPL:13C(6) (N-term)

gi|21223187 TRUE 3814 1348,702985 674,8551305 -0,025261 2 321,487152 58,53 1 14,5 0,452349838 2,210678365 1 KEALEAAGVK 2 ICPL:13C(6) (K); ICPL:13C(6) (N-term)

gi|21223187 TRUE 3390 1940,891935 647,6354957 -0,093587 3 295,1440608 69,31 1 12,1 0,796349117 1,255730657 0 TMGHAGAIVSGSSGTAQAK ICPL (K); ICPL (N-term)

gi|21223187 TRUE 5560 2267,162992 1134,085134 -0,268268 2 455,0854877 77,74 1 13,7 0,724380472 1,380490002 0 NVTKPVVGYVAGFTAPEGK 2 ICPL:13C(6) (K); ICPL:13C(6) (N-term)

gi|21223187 TRUE 5554 2267,162992 756,3925147 -0,042044 3 454,7482791 62,33 1 78,3 0,724380472 1,380490002 0 NVTKPVVGYVAGFTAPEGK 2 ICPL:13C(6) (K); ICPL:13C(6) (N-term)

TRUE BldKB

[S. coelicolor] gi|1532202 279,89 9,800664452 5 4 0,7 0,09 4 1,51 0,19 3741359 112,7 TRUE 3885 1134,565732 567,786504 -0,033008 2 325,6555771 39,38 1 21,7 0,488678323 2,046335909 0 TWTYTLK ICPL:13C(6) (K); ICPL:13C(6) (N-term)

gi|1532202 TRUE 6659 1393,75868 697,382978 -0,163756 2 551,3559754 77,6 1 37,4 0 VFQIFGSNVGGIR

gi|1532202 TRUE 3623 1494,73187 747,869573 -0,124946 2 309,6603189 65,7 1 31,9 0,888012166 1,126110698 0 SLVYAYANTPER ICPL:13C(6) (N-term)

gi|1532202 TRUE 4184 1698,877622 849,942449 -0,122298 2 346,3052352 25,62 1 13,4 0,840106107 1,190325831 0 DAITLALPSDSVFK ICPL:13C(6) (K); ICPL:13C(6) (N-term)

gi|1532202 TRUE 2363 1747,800092 874,403684 -0,235568 2 204,3364765 71,59 1 8,3 0,594272322 1,682730227 0 EIDSATWYEQVGK ICPL:13C(6) (K); ICPL:13C(6) (N-term)

TRUE elongation factor

gi|1091582 274,96 15,11335013 5 4 3,18 0,57 4 0,36 0,06 9534525 217,5 TRUE 3326 942,515546 471,761411 -0,232222 2 290,9491109 35,67 1 78,8 2,807870071 0,356141835 0 EHVLLAR ICPL (N-term)

gi|1091582 TRUE 4020 1801,944257 901,4757665 -0,066733 2 334,7139525 109,65 1 23,2 1,753230624 0,570375618 0 LLDEGQAGENVGLLLR ICPL (N-term)

gi|1091582 TRUE 2654 2146,001523 1073,5044 -0,227799 2 226,0742599 88,09 1 59,7 3,626994358 0,275710382 0 ELLSEYEFPGDDVPVVK ICPL (K); ICPL (N-term)

gi|1091582 TRUE 2656 2158,041791 1079,524534 -0,242467 2 226,1806349 50,95 1 37,6 3,626994358 0,275710382 0 ELLSEYEFPGDDVPVVK ICPL:13C(6) (K); ICPL:13C(6) (N-term)

gi|1091582 TRUE 2406 2471,114973 824,3765083 -0,106225 3 207,3029597 41,55 1 18,2 4,487456251 0,222843398 0 VLHDAYPDINEASAFDQIDK ICPL (K); ICPL (N-term)

TRUE secreted tripeptidyl aminopeptidase

[S. coelicolor A3(2)] gi|21221213 271,8515638 14,34599156 7 4 0,22 0,04 4 5,19 1,02 13279783 208,4 TRUE 3278 991,472761 496,2400185 -0,148437 2 288,1042688 36,03 1 45,4 0,297543419 3,360854031 0 YGYQPPR ICPL:13C(6) (N-term)

gi|21221213 TRUE 3736 1550,744184 775,87573 -0,12446 2 316,6504859 64,91 1 16 0,110183815 9,075743114 0 LDIWQAASDQHR ICPL:13C(6) (N-term)

gi|21221213 TRUE 3498 1953,987256 652,0006027 -0,170108 3 302,0219776 42,38 1 21,8 0,223695195 4,470368703 0 VQENPSAARPLATFDAR ICPL:13C(6) (N-term)

gi|21221213 TRUE 5015 2455,173261 819,0626043 -0,336313 3 423,2189293 64,86 1 37 0,249109062 4,014305995 0 DVNRPTVFYTGGYNVSTNPSR ICPL:13C(6) (N-term)

TRUE malate dehydrogenase

[S. coelicolor A3(2)] gi|21223204 270,9369089 16,71732523 6 6 1,12 0,12 6 0,95 0,1 25764442 255,6 TRUE 3241 1061,585004 531,29614 -0,23608 2 285,063744 47,05 1 51,2 0,935212575 1,069275614 1 RGAAIIEAR ICPL (N-term)

gi|21223204 TRUE 5780 1207,672101 1207,672101 -0,077401 1 468,4439874 55,85 1 54,2 1,278607972 0,782100551 0 LLEITPALK ICPL (K); ICPL (N-term)

gi|21223204 TRUE 1913 1404,658109 702,8326925 -0,188785 2 175,3489511 32,65 1 16,8 0,889415282 1,124334178 0 AINDHAADDIK ICPL:13C(6) (K); ICPL:13C(6) (N-term)

gi|21223204 TRUE 4040 1577,832212 789,419744 -0,083888 2 336,2197189 48,84 1 26,4 0,801160119 1,248189939 0 IASGQLLGADVPVK ICPL (K); ICPL (N-term)

gi|21223204 TRUE 4027 1589,87248 795,439878 -0,099356 2 335,2392859 48,87 1 16,5 1,128242567 0,886334224 0 IASGQLLGADVPVK ICPL:13C(6) (K); ICPL:13C(6) (N-term)

gi|21223204 TRUE 4244 1599,784193 800,3957345 -0,152469 2 350,1751024 89,04 1 90,5 1,727130123 0,578995171 0 WLADEFIPTVAK ICPL (K); ICPL (N-term)

TRUE secreted protease

[S. coelicolor A3(2)] gi|21221367 264,872717 5,377720871 11 7 0,27 0,02 7 3,88 0,31 30191890 548,2 TRUE 3817 1230,619199 615,8132375 -0,090275 2 321,6383691 32,83 1 11,7 0,220282975 4,539615474 0 LGLAEYNTK ICPL:13C(6) (K); ICPL:13C(6) (N-term)

gi|21221367 TRUE 2115 1305,597104 653,30219 -0,07358 2 188,1517517 57,44 1 15,7 0,380076273 2,631050848 0 YQTDGGLAMK ICPL:13C(6) (K); ICPL:13C(6) (N-term)

gi|21221367 TRUE 4171 1431,734571 716,3709235 -0,130647 2 345,1436192 87,27 1 143,8 0,227179944 4,401797008 0 LVYPLDAYAGK ICPL:13C(6) (K); ICPL:13C(6) (N-term)

gi|21221367 TRUE 4154 1431,734571 716,3709235 -0,067447 2 343,9801611 87,46 1 99,8 0,21694243 4,609517833 0 LVYPLDAYAGK ICPL:13C(6) (K); ICPL:13C(6) (N-term)

gi|21221367 TRUE 4137 1431,734571 716,3709235 -0,055847 2 342,7965189 85,09 1 50,3 0,226801247 4,409146842 0 LVYPLDAYAGK ICPL:13C(6) (K); ICPL:13C(6) (N-term)

gi|21221367 TRUE 2590 1431,734571 716,3709235 0,004153 2 221,988277 79,63 1 32,8 0,282200626 3,543578245 0 LVYPLDAYAGK ICPL:13C(6) (K); ICPL:13C(6) (N-term)

gi|21221367 TRUE 2064 1530,695463 510,9033383 -0,166915 3 185,1782935 38,28 1 27 0,331399101 3,017509692 0 EYAQYYIAENR ICPL:13C(6) (N-term)

gi|21221367 TRUE 2061 1530,695463 765,8513695 -0,165339 2 185,0828765 54,38 1 46,5 0,331399101 3,017509692 0 EYAQYYIAENR ICPL:13C(6) (N-term)

TRUE aconitate hydratase

[S. coelicolor A3(2)] gi|21224335 264,3738177 4,756637168 5 3 1,04 0,14 3 1,01 0,13 9494441 123,6 TRUE 5672 1074,630567 1074,630567 -0,043667 1 461,9421037 33,41 1 29,6 1,261866794 0,792476674 0 VLLENLLR ICPL (N-term)

gi|21224335 TRUE 5676 1074,630567 537,8189215 -0,028443 2 462,1435874 56,28 1 34,4 1,261866794 0,792476674 0 VLLENLLR ICPL (N-term)

gi|21224335 TRUE 2428 1307,645765 654,3265205 -0,117641 2 208,9932183 65,92 1 13,6 0,740398086 1,350624778 0 EQGLWLDPK ICPL:13C(6) (K); ICPL:13C(6) (N-term)

gi|21224335 TRUE 2477 1809,884519 905,4458975 -0,164395 2 212,7798434 98,15 1 22,8 1,116805429 0,89541112 0 DALGVDQDGNPVFLK ICPL:13C(6) (K); ICPL:13C(6) (N-term)

TRUE secreted protein

[S. coelicolor A3(2)] gi|21224891 260,2 24,4047619 7 7 2,13 0,39 7 0,58 0,11 4751273 125,2 TRUE 3471 1099,589445 550,2983605 -0,093121 2 300,3420443 47,57 1 10,9 4,388090151 0,227889575 0 EHVLVEIR ICPL (N-term)

gi|21224891 TRUE 4093 1350,741575 675,8744255 0,055149 2 339,6654773 66,44 1 11,5 1,015184564 0,985042558 0 ILAEQNIVLK ICPL (K); ICPL (N-term)

gi|21224891 TRUE 4073 1350,741575 675,8744255 0,062949 2 338,4145275 69,36 1 28,1 1,337658812 0,747574786 0 ILAEQNIVLK ICPL (K); ICPL (N-term)

gi|21224891 TRUE 963 2417,166654 806,3937353 -0,324106 3 92,85445812 58,43 1 23 3,01557351 0,33161188 0 GDGHIVLADCAGGDNLLQFLAR ICPL (N-term)

gi|21224891 TRUE 6961 2417,166654 806,3937353 -0,186406 3 572,5255333 75,83 1 8,2 1,555939144 0,642698658 0 GDGHIVLADCAGGDNLLQFLAR ICPL (N-term)

gi|21224891 TRUE 6845 2573,267759 644,0723968 -0,088387 4 564,2233589 67,44 1 23,6 1,782553341 0,56099303 1 RGDGHIVLADCAGGDNLLQFLAR ICPL (N-term)

gi|21224891 TRUE 825 2573,267759 858,427437 0,039489 3 84,32797493 60,02 1 19,9 1,90701855 0,524378748 1 RGDGHIVLADCAGGDNLLQFLAR ICPL (N-term)

TRUE ribosome recycling factor

[S. coelicolor A3(2)] gi|21223982 243,0909952 25,94594595 5 3 2,3 0,37 3 0,47 0,08 9873915 146,5 TRUE 1984 926,436628 463,721952 -0,170904 2 180,2944349 53,36 1 11,8 2,38623229 0,419070685 0 EDFAAIR ICPL (N-term)

gi|21223982 TRUE 2190 1277,616041 639,3116585 -0,099717 2 193,0332013 45,06 1 22,5 1,529914325 0,653631372 1 HKEAELLEV ICPL (K); ICPL (N-term)

gi|21223982 TRUE 2629 1644,746107 822,8766915 -0,058983 2 224,5753933 66,89 1 43,5 2,968621577 0,336856677 0 MIEETLLEAEEK ICPL (K); ICPL (N-term)

TRUE secreted protein

[S. coelicolor A3(2)] gi|21221339 241,77 9,471365639 5 4 1,29 0,14 4 0,81 0,09 6296381 311,3 TRUE 3491 913,452634 457,229955 -0,14511 2 301,5129024 39,79 1 10,5 1,498492254 0,66733745 0 LTFGGSAR ICPL (N-term)

gi|21221339 TRUE 1748 1230,538541 615,7729085 -0,114217 2 164,5256434 65,28 1 13,3 1,630804944 0,613194118 0 QTSYLADGDR ICPL (N-term)

gi|21221339 TRUE 3171 1359,676404 680,34184 -0,17908 2 280,2867691 46,45 1 101,7 1,0298496 0,971015573 0 NVVHTSVGGTGAR ICPL (N-term)

gi|21221339 TRUE 3172 1359,676404 680,34184 -0,16088 2 280,0752192 51,05 1 152,1 1,0298496 0,971015573 0 NVVHTSVGGTGAR ICPL (N-term)

gi|21221339 TRUE 3472 1399,660047 700,3336615 -0,091923 2 300,5540523 85,65 1 33,7 0,992665505 1,007388687 0 YSVVNEGISGNR ICPL (N-term)

TRUE secreted protein

[S. coelicolor A3(2)] gi|21223402 238,3269089 18,06722689 7 5 1,37 0,1 5 0,75 0,06 32053289 210,5 TRUE 1888 1082,526517 541,7668965 -0,165393 2 173,7452765 47,6 1 44,3 1,09600615 0,912403639 0 TNPATQIK ICPL (K); ICPL (N-term)

gi|21223402 TRUE 1885 1094,566785 547,7870305 -0,183261 2 173,9178354 53,38 1 31,1 1,09600615 0,912403639 0 TNPATQIK ICPL:13C(6) (K); ICPL:13C(6) (N-term)

gi|21223402 TRUE 1895 1101,474805 551,2410405 -0,135481 2 174,3506349 37,18 1 8,5 1,135477855 0,880686484 0 YASAGADWR ICPL (N-term)

gi|21223402 TRUE 1896 1101,474805 551,2410405 -0,113681 2 174,3172599 59,91 1 51,3 1,135477855 0,880686484 0 YASAGADWR ICPL (N-term)

gi|21223402 TRUE 3760 1246,530942 623,769109 -0,024018 2 318,2083109 59,12 1 28,9 1,576204868 0,634435295 0 WGLSYMDNR ICPL (N-term)

gi|21223402 TRUE 1089 2263,907911 1132,457594 -0,250587 2 102,5913243 39,95 1 8,4 1,642340461 0,608887148 0 YGSPCDAWAFWQANHWY ICPL (N-term)

gi|21223402 TRUE 5668 2263,907911 755,3074877 -0,043463 3 461,8461122 68,44 1 38 1,414737498 0,70684491 0 YGSPCDAWAFWQANHWY ICPL (N-term)

TRUE secreted protein

[S. coelicolor A3(2)] gi|21224075 237,73 16,98113208 4 3 2,22 0,19 3 0,46 0,04 7024383 104 TRUE 3261 1271,660306 424,558286 -0,170058 3 286,5924608 56,67 1 24,3 1,787116625 0,559560572 1 RQDLAHLASR ICPL (N-term)

gi|21224075 TRUE 3263 1271,660306 636,333791 -0,158782 2 286,7534523 33,53 1 43,1 1,787116625 0,559560572 1 RQDLAHLASR ICPL (N-term)

gi|21224075 TRUE 4223 1623,711454 812,359365 -0,04053 2 348,8274939 88,13 1 19,6 2,53753564 0,394083135 0 FDVGTEAFVWDK ICPL (K); ICPL (N-term)

gi|21224075 TRUE 3856 1702,770734 851,889005 -0,05081 2 323,9616523 92,93 1 17 2,321596306 0,430738108 0 LVSPDSSFTSEWSR ICPL (N-term)

TRUE transaldolase

[S. coelicolor A3(2)] gi|21220422 235,9538177 10,48387097 5 3 0,5 0,04 3 2,05 0,15 11285724 157,6 TRUE 5060 1464,778516 732,892896 -0,120192 2 425,7463458 62,74 1 27,1 0,507359268 1,970989915 0 QRPLWASTGVK ICPL:13C(6) (K); ICPL:13C(6) (N-term)

gi|21220422 TRUE 5059 1464,778516 488,9310227 -0,120068 3 425,7596039 46,7 1 42,4 0,507359268 1,970989915 0 QRPLWASTGVK ICPL:13C(6) (K); ICPL:13C(6) (N-term)

gi|21220422 TRUE 4087 1766,802015 883,9046455 -0,033491 2 339,4288773 48,08 1 45,1 0,564547229 1,771330988 0 LAYQAYEEVFGTDR ICPL (N-term)

gi|21220422 TRUE 4092 1766,802015 883,9046455 0,003709 2 339,6345024 92,68 1 23,6 0,564547229 1,771330988 0 LAYQAYEEVFGTDR ICPL (N-term)

gi|21220422 TRUE 5829 1827,956586 914,481931 -0,154662 2 471,5400375 85,58 1 19,4 0,414469954 2,412720128 0 LGISYDEVVQLLEK ICPL:13C(6) (K); ICPL:13C(6) (N-term)

TRUE ORF3

[S. coelicolor A3(2)] gi|565055 235,42 27,10280374 4 4 12,05 1,44 4 0,09 0,01 12042173 214,8 TRUE 1950 1338,628446 669,817861 -0,055922 2 177,9455015 110,04 1 66 13,64986322 0,073260807 0 LTGGSGLGDSDVR ICPL (N-term)

gi|565055 TRUE 1970 1338,628446 669,817861 0,020078 2 179,5625602 38,88 1 46,8 12,01460111 0,08323206 0 LTGGSGLGDSDVR ICPL (N-term)

gi|565055 TRUE 4148 2617,220502 873,0783513 -0,215854 3 343,5503605 58,3 1 33,7 14,49335817 0,068997122 0 ATYDPDPITAFAEYNQIVNDVR ICPL (N-term)

gi|565055 TRUE 5611 2675,183759 892,399437 -0,152211 3 458,2064205 67,08 1 68,3 7,92601528 0,126166802 0 TTGPLNFVSGGSGYEDPGFYWYR ICPL (N-term)

TRUE cytochrome c oxidase subunit II

[S. coelicolor A3(2)] gi|21220633 215,23 13,1661442 6 4 2,36 0,22 4 0,44 0,04 15915124 211,5 TRUE 5099 827,441007 414,2241415 -0,168683 2 427,9319127 44,48 1 55,2 3,040401924 0,328903883 0 FVLTSR ICPL (N-term)

gi|21220633 TRUE 5101 827,441007 827,441007 -0,088707 1 427,9443207 39,19 1 28,9 3,040401924 0,328903883 0 FVLTSR ICPL (N-term)

gi|21220633 TRUE 1951 975,489393 488,2483345 -0,107869 2 178,1486599 43,2 1 50,9 2,178816084 0,458964851 0 ELAGIPDR ICPL (N-term)

gi|21220633 TRUE 2049 1454,669874 727,838575 -0,17235 2 184,4046765 53,31 1 41,6 2,370265909 0,421893593 0 YEQHLQDLAK ICPL (K); ICPL (N-term)

gi|21220633 TRUE 2050 1466,710142 733,858709 -0,128218 2 184,4331933 41,37 1 20,2 2,370265909 0,421893593 0 YEQHLQDLAK ICPL:13C(6) (K); ICPL:13C(6) (N-term)

gi|21220633 TRUE 3511 2187,025388 729,67998 -0,12444 3 302,9397691 74,24 1 14,7 1,835254146 0,544883662 1 KGQTGYVPAGIAQTSHEK 2 ICPL (K); ICPL (N-term)

TRUE glutamate binding protein

[S. coelicolor A3(2)] gi|21224122 207,2909952 13,30935252 5 3 2,23 0,28 3 0,47 0,06 22872845 86,4 TRUE 2052 1235,511594 618,259435 -0,01827 2 184,6906519 49,5 1 3,9 1,626107487 0,614965498 0 DNFGPANYK ICPL (K); ICPL (N-term)

gi|21224122 TRUE 2483 1591,706327 796,3568015 -0,050403 2 213,4314935 66,57 1 26,5 2,399436396 0,416764538 0 LGYTEDQIEWK ICPL (K); ICPL (N-term)

gi|21224122 TRUE 2486 1603,746595 802,3769355 -0,019471 2 213,3615437 46,21 1 15 2,399436396 0,416764538 0 LGYTEDQIEWK ICPL:13C(6) (K); ICPL:13C(6) (N-term)

gi|21224122 TRUE 5512 2032,065061 1016,536169 -0,156137 2 452,2017623 95,22 1 24,9 2,665130903 0,375216091 0 VDFAGPYLLAHQDVLIR ICPL (N-term)

gi|21224122 TRUE 5508 2032,065061 678,0265377 -0,027713 3 452,2643207 46,82 1 16,1 2,665130903 0,375216091 0 VDFAGPYLLAHQDVLIR ICPL (N-term)

TRUE DNA polymerase III subunit beta

[S. coelicolor A3(2)] gi|21222286 197,127904 22,60638298 5 5 2,31 0,12 5 0,44 0,02 4799719 107,4 TRUE 5334 996,533506 498,770391 -0,169582 2 441,6439709 27,19 1 23,3 2,574306915 0,388454071 0 YLIMPVR ICPL (N-term)

gi|21222286 TRUE 3721 1290,668851 645,8380635 -0,133727 2 315,4095856 43,54 1 27,1 2,168929734 0,461056891 0 DDTLPVLTGVR ICPL (N-term)

gi|21222286 TRUE 3473 1728,902738 576,97243 -0,17169 3 300,5384773 45,29 1 27,2 2,679555598 0,373196212 0 ALPNRPVEISTDGVR ICPL (N-term)

gi|21222286 TRUE 2627 2539,231061 847,081871 -0,106113 3 224,3319351 45,75 1 16,3 2,089026437 0,478691884 0 LSFEQGVLILEAGSSDDAQAVER ICPL (N-term)

gi|21222286 TRUE 1141 2808,416257 936,8102697 -0,163109 3 106,4386992 41,88 1 13,5 2,030695183 0,492442198 0 ALTSGDQVILALSGSGAGEGLIGFEGAGR ICPL (N-term)

TRUE hypothetical protein SCO3324

[S. coelicolor A3(2)] gi|21221755 195,5 6,92124105 4 3 10,38 1,6 3 0,1 0,02 12912251 150,2 TRUE 1982 937,452619 469,2299475 -0,136095 2 180,1419933 51,47 1 51,7 6,925481673 0,144394289 0 ETWGALR ICPL (N-term)

gi|21221755 TRUE 3614 1114,552717 557,7799965 -0,133793 2 309,260336 71,03 1 34,8 12,16492793 0,082203528 0 AYLDLASTR ICPL (N-term)

gi|21221755 TRUE 1875 1500,707731 500,9074277 -0,146183 3 173,0346098 64,82 1 36 11,98391758 0,083445167 0 SGPLDHESLGEVR ICPL (N-term)

gi|21221755 TRUE 1874 1500,707731 750,8575035 -0,129807 2 172,9412599 73 1 27,7 11,98391758 0,083445167 0 SGPLDHESLGEVR ICPL (N-term)

TRUE superoxide dismutase

[S. coelicolor A3(2)] gi|21219516 189,43 20,46511628 7 4 0,85 0,1 4 1,24 0,14 46837800 220,3 TRUE 3665 1349,725065 675,3661705 -0,147541 2 312,0547941 27,38 1 28,6 0,624925875 1,600189782 1 ERGDSLLLKP ICPL:13C(6) (K); ICPL:13C(6) (N-term)

gi|21219516 TRUE 2109 1492,702619 746,8549475 -0,114095 2 187,8556098 85,84 1 78,5 1,167536725 0,856504107 0 GANDTLEQLAEAR ICPL (N-term)

gi|21219516 TRUE 2108 1492,702619 746,8549475 -0,113295 2 188,1353431 45,87 1 6,7 1,167536725 0,856504107 0 GANDTLEQLAEAR ICPL (N-term)

gi|21219516 TRUE 2111 1492,702619 498,239057 -0,088671 3 188,0443597 65,09 1 3,6 1,167536725 0,856504107 0 GANDTLEQLAEAR ICPL (N-term)

gi|21219516 TRUE 5370 2197,067243 733,027265 -0,111795 3 443,9777623 76,21 1 24,4 0,772977253 1,293699131 0 AAATTQGSGWGVLAYEPLSGR ICPL (N-term)

gi|21219516 TRUE 6847 2203,087385 735,033979 -0,237237 3 564,585984 38,25 1 52,7 0,835645693 1,196679416 0 AAATTQGSGWGVLAYEPLSGR ICPL:13C(6) (N-term)

TRUE NLP/P60 family protein

[S. coelicolor A3(2)] gi|21222944 188,63 13,71841155 11 5 0,25 0,02 5 4,06 0,32 58838034 624,1 TRUE 1954 969,471479 485,2393775 -0,110955 2 178,191085 44,69 1 20,7 0,265487382 3,766657352 0 TDLAEAK ICPL:13C(6) (K); ICPL:13C(6) (N-term)

gi|21222944 TRUE 3527 974,541776 487,774526 -0,118652 2 303,9479861 66,18 1 28,7 0,244517399 4,089688528 0 QVGISLPR ICPL (N-term)

gi|21222944 TRUE 3528 974,541776 974,541776 0,011324 1 303,975136 42,79 1 45,5 0,244517399 4,089688528 0 QVGISLPR ICPL (N-term)

gi|21222944 TRUE 3523 980,561918 490,784597 -0,174994 2 303,6916523 57,25 1 140,4 0,244517399 4,089688528 0 QVGISLPR ICPL:13C(6) (N-term)

gi|21222944 TRUE 3526 980,561918 980,561918 -0,060018 1 303,8163691 25,9 1 139,8 0,244517399 4,089688528 0 QVGISLPR ICPL:13C(6) (N-term)

gi|21222944 TRUE 3999 2530,134351 844,0496343 -0,115003 3 333,0286272 66,02 1 20,9 0,277635523 3,601844572 0 AYVSGATGPSAYDCSGLVQAAFK ICPL (K); ICPL

(N-term)

gi|21222944 TRUE 4014 2542,174619 848,063057 -0,206571 3 333,9203861 77,76 1 57,7 0,301386319 3,318000648 0 AYVSGATGPSAYDCSGLVQAAFK ICPL:13C(6) (K); ICPL:13C(6) (N-term)

gi|21222944 TRUE 4028 2542,174619 848,063057 -0,177171 3 335,1008272 68,1 1 39,3 0,181148764 5,520324725 0 AYVSGATGPSAYDCSGLVQAAFK ICPL:13C(6) (K); ICPL:13C(6) (N-term)

gi|21222944 TRUE 3997 2542,174619 848,063057 -0,119271 3 332,7867605 75,84 1 40,6 0,277635523 3,601844572 0 AYVSGATGPSAYDCSGLVQAAFK ICPL:13C(6) (K); ICPL:13C(6) (N-term)

TRUE hypothetical protein SCO2367

[S. coelicolor A3(2)] gi|21220835 188,1369089 12,56544503 2 2 1,17 0,13 2 0,88 0,09 7003286 64,1 TRUE 3356 1183,585432 592,296354 -0,113308 2 292,9066859 89,46 1 17,2 0,992244439 1,00781618 0 AVGQGYASGLR ICPL (N-term)

gi|21220835 TRUE 5199 1529,749555 765,3784155 -0,072831 2 434,0231207 101,2 1 46,9 1,342326029 0,744975497 0 GQTFGQVSNAFIR ICPL (N-term)

TRUE 50S ribosomal protein L7/L12

[S. coelicolor A3(2)] gi|21223035 185,55 23,62204724 6 5 2,37 0,27 5 0,45 0,05 7435334 145,6 TRUE 1845 1027,484327 514,2458015 -0,169203 2 171,0711767 55,34 1 9,2 1,977770849 0,505619749 0 GAGASVEVK ICPL (K); ICPL (N-term)

gi|21223035 TRUE 3923 1070,551664 535,77947 -0,08314 2 328,1852773 52 1 29,7 2,11328883 0,473196085 0 ELTSLGLK ICPL (K); ICPL (N-term)

gi|21223035 TRUE 2433 1695,83769 565,9507473 -0,243142 3 209,3768434 52,53 1 18,2 2,337309596 0,427842337 0 DLVDGAPKPVLEK 2 ICPL (K); ICPL (N-term)

gi|21223035 TRUE 2429 1695,83769 848,422483 -0,143166 2 209,5068765 78,21 1 40,3 2,337309596 0,427842337 0 DLVDGAPKPVLEK 2 ICPL (K); ICPL (N-term)

gi|21223035 TRUE 2448 1695,83769 565,9507473 -0,067342 3 210,9274349 48,36 1 19,5 3,676586613 0,271991416 0 DLVDGAPKPVLEK 2 ICPL (K); ICPL (N-term)

gi|21223035 TRUE 2414 1695,83769 848,422483 -0,049366 2 207,9603346 74,98 1 28,7 1,75556278 0,569617909 0 DLVDGAPKPVLEK 2 ICPL (K); ICPL (N-term)

TRUE bifunctional protein (methylenetetrahydrofolate dehydrogenase and methenyltetrahydrofolate cyclohydrolase)

[S. coelicolor A3(2)] gi|21223201 183,5 10,21126761 2 2 1,7 0,04 2 0,59 0,01 6574389 51,2 TRUE 5783 1516,81178 758,909528 0,001744 2 468,6097458 81,44 1 40,8 1,752377151 0,570653412 0 AQLLVNVVEAAER ICPL (N-term)

gi|21223201 TRUE 2386 1860,933744 930,97051 -0,01902 2 205,700085 102,06 1 10,4 1,647386533 0,60702208 0 ELPATATQEEIEAVVR ICPL (N-term)

TRUE serine/threonine protein kinase

[S. coelicolor A3(2)] gi|32141292 182,81 4,361617764 3 3 0,24 0,1 3 6,58 2,85 15180180 108,2 TRUE 5103 1426,695798 713,851537 -0,086474 2 428,1896039 77,84 1 54,7 0,473729033 2,110911366 0 SLVGGAWGEHFR ICPL:13C(6) (N-term)

gi|32141292 TRUE 5102 1901,923612 634,6460547 -0,166964 3 428,0717709 64,07 1 25,6 0,147690794 6,770902707 0 HDATLYTAGAGWSTLAR ICPL:13C(6) (N-term)

gi|32141292 TRUE 5473 2935,448652 979,1544013 -0,362704 3 450,1645207 40,9 1 27,9 0,09797791 10,20638223 0 VTYAFKPGVPTSVGVIPGDGTTAYCR ICPL:13C(6) (K); ICPL:13C(6) (N-term)

TRUE phosphoglycerate kinase

[S. coelicolor A3(2)] gi|21220431 178,3109952 15,13647643 4 3 1,16 0,07 3 0,87 0,05 6061545 72,2 TRUE 5127 1352,751234 676,879255 -0,08911 2 429,6475122 65,01 1 30,1 1,228229259 0,814180246 0 RPYVVALGGAK ICPL:13C(6) (K); ICPL:13C(6) (N-term)

gi|21220431 TRUE 3634 1516,75427 758,880773 -0,144946 2 310,2652773 41,33 1 22,8 1,261988504 0,792400245 0 GAPDPAFSLAPAAAR ICPL (N-term)

gi|21220431 TRUE 4000 2404,225568 802,08004 0,09468 3 333,0948939 49,33 1 7,9 0,996449775 1,003562874 0 AVAQALVDAPGFTVVGGGDSAAAVR ICPL (N-term)

TRUE RNA polymerase alpha subunit

[S. coelicolor A3(2)] gi|1524340 172,4984727 10,32448378 4 1 2,31 0 1 0,43 0 5901180 107,7 TRUE 4118 1387,719068 694,363172 -0,032544 2 341,451152 26,57 2 17 1 AKLAGMGLALK 2 ICPL (K); ICPL (N-term)

gi|1524340 TRUE 5713 1540,804594 770,905935 -0,04727 2 464,5186541 62,94 1 32,7 2,310635782 0,432781318 0 IPVDSIYSPVLK ICPL (K); ICPL (N-term)

TRUE carboxypeptidase

[S. coelicolor A3(2)] gi|21224458 168,9 5,321507761 2 2 0,38 0,16 2 3,57 1,52 4389916 77,5 TRUE 2151 1385,700254 693,353765 -0,11533 2 190,5690263 85,13 1 42,5 0,187415859 5,335727765 0 TLTGEDVAELAR ICPL:13C(6) (N-term)

gi|21224458 TRUE 3723 1457,732603 729,3699395 -0,094879 2 316,0166277 83,77 1 35 0,570100702 1,754076073 0 TSDNNIAETLLR ICPL:13C(6) (N-term)

TRUE lipoprotein

[S. coelicolor A3(2)] gi|21222720 164,12 13,63636364 4 4 3,65 0,77 4 0,32 0,07 6165196 149,7 TRUE 2402 1286,568799 643,7880375 -0,107675 2 207,0443351 77,92 1 49,5 5,048495819 0,198078801 0 FGVDPDIADK ICPL (K); ICPL (N-term)

gi|21222720 TRUE 2422 1286,568799 643,7880375 -0,093075 2 208,8480434 65,94 1 27,4 2,799502723 0,357206297 0 FGVDPDIADK ICPL (K); ICPL (N-term)

gi|21222720 TRUE 2445 1286,568799 643,7880375 -0,082675 2 210,4885847 74,66 1 13,1 4,776750959 0,209347318 0 FGVDPDIADK ICPL (K); ICPL (N-term)

gi|21222720 TRUE 1856 1367,643751 684,3255135 -0,128827 2 171,8942685 86,2 1 59,7 1,881904992 0,531376453 0 SITVDQDETVR ICPL (N-term)

TRUE thioredoxin

[S. coelicolor A3(2)] gi|21223779 162,34 7,547169811 5 4 2,05 0,23 4 0,51 0,06 5648806 126,1 TRUE 5699 801,429365 401,2183205 -0,220441 2 463,6781287 37,48 1 29,3 2,346712434 0,426128053 0 FLLAK ICPL (K); ICPL (N-term)

gi|21223779 TRUE 5716 801,429365 401,2183205 -0,154841 2 464,6353959 37,26 1 13,7 2,555344387 0,391336684 0 FLLAK ICPL (K); ICPL (N-term)

gi|21223779 TRUE 3483 1046,562894 523,785085 -0,12937 2 301,1538107 47,2 1 28 1,439065424 0,694895439 0 QLSPVLER ICPL (N-term)

gi|21223779 TRUE 5422 1316,732068 658,869672 -0,027344 2 446,9712626 77,66 1 27,1 1,84258537 0,542715695 0 LGLAQAELLQR ICPL (N-term)

TRUE branched chain amino acid binding protein

[S. coelicolor A3(2)] gi|21220490 156,92 4,545454545 4 4 2,53 0,46 4 0,45 0,08 2563695 72,7 TRUE 2517 1045,498929 523,2531025 -0,141805 2 216,1061767 56,06 1 4,1 2,079930154 0,480785375 0 VFVIDDK ICPL (K); ICPL (N-term)

gi|21220490 TRUE 2504 1045,498929 523,2531025 -0,123405 2 215,0211767 52,68 1 19 3,114879882 0,321039667 0 VFVIDDK ICPL (K); ICPL (N-term)

gi|21220490 TRUE 2372 1539,638669 770,3229725 -0,104745 2 204,858693 100,86 1 29,5 3,472322246 0,287991704 0 VSFDEFGDATNK ICPL (K); ICPL (N-term)

gi|21220490 TRUE 2416 1539,638669 770,3229725 -0,058545 2 208,1420183 56,89 1 20,1 1,439693654 0,694592212 0 VSFDEFGDATNK ICPL (K); ICPL (N-term)

TRUE nucleoside diphosphate kinase

[S. coelicolor A3(2)] gi|21221070 156,7009952 27,00729927 5 3 2,44 0,61 3 0,49 0,12 28991776 131,5 TRUE 5427 1362,716428 681,861852 -0,200904 2 447,2684205 59,84 1 16,1 4,009707001 0,249394781 0 AGWQITALELR ICPL (N-term)

gi|21221070 TRUE 5424 1362,716428 681,861852 0,018696 2 447,0857789 70,03 1 31,6 4,009707001 0,249394781 0 AGWQITALELR ICPL (N-term)

gi|21221070 TRUE 5415 1420,794691 710,9009835 -0,032367 2 446,7082711 56,65 1 32,4 1,785838061 0,559961187 0 SLVLLKPDAVR ICPL (K); ICPL (N-term)

gi|21221070 TRUE 5416 1432,834958 716,921117 -0,106634 2 446,7199709 43,44 1 35,8 1,785838061 0,559961187 0 SLVLLKPDAVR ICPL:13C(6) (K); ICPL:13C(6) (N-term)

gi|21221070 TRUE 2023 2010,882795 670,9657823 -0,174547 3 183,0743266 34,02 1 15,6 1,556255479 0,642568019 0 TLDTETLEQHYGEHK ICPL (K); ICPL (N-term)

TRUE secreted hydrolase

[S. coelicolor A3(2)] gi|21220452 154,26 10,38062284 3 3 1,77 0,08 3 0,57 0,03 8923693 28,8 TRUE 4252 938,534543 938,534543 -0,057943 1 350,5327936 44,01 1 13,5 1,704620021 0,586641004 0 LLLELK ICPL (K); ICPL (N-term)

gi|21220452 TRUE 5134 1346,685148 673,846212 -0,105024 2 430,1415122 39,09 1 2,4 1,624066515 0,615738328 0 VPTLEQYVHR ICPL (N-term)

gi|21220452 TRUE 2122 1657,818006 829,412641 -0,151482 2 188,6372183 71,16 1 12,9 1,973301523 0,506764926 0 DGELVVLHDESLAR ICPL (N-term)

TRUE peptidyl-prolyl cis-trans isomerase

[S. coelicolor A3(2)] gi|21225776 154,14 16,36363636 2 2 1,23 0,53 2 1,12 0,49 25372475 70,6 TRUE 3509 896,441358 448,724317 -0,172634 2 302,7091856 47,64 1 30,7 0,593573792 1,684710499 0 HVVFGK ICPL (K); ICPL (N-term)

gi|21225776 TRUE 5386 2412,115598 804,71005 -0,00825 3 444,7531538 106,5 1 39,9 1,843958814 0,542311462 0 NFLELATGEHGFGYAGSPFHR ICPL (N-term)

TRUE aminopeptidase N

[S. coelicolor A3(2)] gi|21221100 154,0815638 3,967327888 3 3 1,37 0,19 3 0,77 0,11 2611393 41,9 TRUE 952 1111,662214 556,334745 -0,02049 2 92,18214133 51,24 1 1,9 1,828401728 0,546925758 0 IALPGLLPGR ICPL (N-term)

gi|21221100 TRUE 5128 1302,658918 651,833097 -0,100394 2 429,7515703 41,87 1 25,8 1,252855688 0,798176526 0 WTEATLAHLR ICPL (N-term)

gi|21221100 TRUE 5788 1565,780546 783,393911 -0,045022 2 468,8931207 66,83 1 14,2 1,039129741 0,962343738 0 LSDLLGALEETSGR ICPL (N-term)

TRUE superoxide dismutase

[S. coelicolor A3(2)] gi|21223621 150,18 22,13740458 9 7 2,37 0,24 7 0,45 0,05 16000198 236,9 TRUE 2568 1033,477781 517,2425285 -0,135857 2 220,2462514 34,31 1 20,3 2,531417421 0,3950356 0 IFWETK ICPL (K); ICPL (N-term)

gi|21223621 TRUE 4106 1033,477781 517,2425285 -0,126257 2 340,8700187 30,19 1 54,3 2,074665898 0,48200532 0 IFWETK ICPL (K); ICPL (N-term)

gi|21223621 TRUE 4157 1033,477781 517,2425285 -0,104057 2 344,1520107 33,17 1 16,6 3,511568283 0,284773047 0 IFWETK ICPL (K); ICPL (N-term)

gi|21223621 TRUE 4126 1033,477781 517,2425285 -0,099857 2 342,4909691 33,75 1 36,4 2,154444256 0,464156822 0 IFWETK ICPL (K); ICPL (N-term)

gi|21223621 TRUE 4107 1045,518049 523,2626625 -0,060525 2 340,8151104 28,25 1 27,3 2,074665898 0,48200532 0 IFWETK ICPL:13C(6) (K); ICPL:13C(6) (N-term)

gi|21223621 TRUE 4097 1359,657911 680,3325935 -0,022187 2 340,4310859 48,08 1 18 2,453608297 0,407563017 0 ALDYIAQIDK ICPL (K); ICPL (N-term)

gi|21223621 TRUE 4054 1371,698179 686,3527275 -0,032055 2 336,9499355 51,36 1 18,1 2,486046198 0,40224514 0 ALDYIAQIDK ICPL:13C(6) (K); ICPL:13C(6) (N-term)

gi|21223621 TRUE 5484 1779,870038 890,438657 -0,149514 2 450,6893623 64,51 1 33,6 1,359796588 0,73540411 0 YPELHQLVNDTLK ICPL (K); ICPL (N-term)

TRUE 30S ribosomal protein S1

[S. coelicolor A3(2)] gi|21220480 149,0209952 5,378486056 5 3 4,4 0,64 3 0,24 0,04 20217073 1406,7 TRUE 7047 1110,609479 555,8083775 -0,267955 2 578,7038581 68,97 1 1193,8 5,901325671 0,169453451 0 LVPFGAFVR ICPL (N-term)

gi|21220480 TRUE 7048 1110,609479 1110,609479 -0,092179 1 578,7173168 31,18 1 143,8 5,901325671 0,169453451 0 LVPFGAFVR ICPL (N-term)

gi|21220480 TRUE 1046 1110,609479 1110,609479 -0,068679 1 99,11780746 26,25 1 36 4,066685847 0,24590048 0 LVPFGAFVR ICPL (N-term)

gi|21220480 TRUE 2141 1581,708066 791,357671 -0,091542 2 190,0499762 51,75 1 25,8 3,237003456 0,30892769 0 ATQEDPWQQFAR ICPL (N-term)

TRUE metallopeptidase

[S. coelicolor A3(2)] gi|21225030 148,19 4,545454545 4 2 1,13 0,16 2 0,92 0,13 5672238 166,3 TRUE 4199 1620,769285 810,8882805 -0,071361 2 347,683136 80,16 1 46,9 0,900627301 1,110337204 0 NAIGDDAFFTVLK ICPL (K); ICPL (N-term)

gi|21225030 TRUE 2672 1632,809553 816,9084145 -0,120629 2 227,1310098 47,02 1 21 1,353793572 0,738665052 0 NAIGDDAFFTVLK ICPL:13C(6) (K); ICPL:13C(6) (N-term)

gi|21225030 TRUE 4210 1632,809553 816,9084145 -0,067629 2 348,0056859 103,96 1 49,4 0,900627301 1,110337204 0 NAIGDDAFFTVLK ICPL:13C(6) (K); ICPL:13C(6) (N-term)

TRUE NLP/P60 family secreted protein

[S. coelicolor A3(2)] gi|21223174 143,8463767 9,547738693 4 3 1,94 0,1 3 0,52 0,03 4062569 69,1 TRUE 3391 889,489011 445,2481435 -0,146687 2 295,2335024 37,27 1 3,6 2,167501566 0,461360682 0 AAGVTLPR ICPL (N-term)

gi|21223174 TRUE 2543 1471,695066 736,351171 -0,033942 2 218,0991933 86,69 1 21 1,741520653 0,574210819 0 ELLSELTAEEK ICPL (K); ICPL (N-term)

gi|21223174 TRUE 2549 1483,735334 742,371305 -0,12221 2 218,362093 89,31 1 6,4 1,741520653 0,574210819 0 ELLSELTAEEK ICPL:13C(6) (K); ICPL:13C(6) (N-term)

gi|21223174 TRUE 4010 2253,034589 751,683047 -0,077541 3 333,8657691 32,17 1 38,1 1,917979841 0,521381914 0 EESIFYDGESSIYSVVRPA ICPL (N-term)

TRUE secreted protein

[S. coelicolor A3(2)] gi|21220087 137,08 5,314009662 2 2 6,36 1,89 2 0,18 0,05 4592062 72,5 TRUE 7029 1256,621122 628,814199 -0,115398 2 577,2906416 59,14 1 39,8 8,777060637 0,113933359 0 FGVSPFGVWR ICPL (N-term)

gi|21220087 TRUE 3855 1484,749174 742,878225 0,09995 2 324,0180021 77,94 1 32,7 3,925816332 0,254724092 0 AELIEHLDNAVR ICPL (N-term)

TRUE hydrolase

[S. coelicolor A3(2)] gi|21222125 134,05 7,913669065 5 3 1,04 0,03 3 0,96 0,03 9122639 125,9 TRUE 5178 928,467573 464,7374245 -0,219849 2 432,6957623 56,95 1 41,4 0,975234022 1,025394908 0 TFGFPVR ICPL (N-term)

gi|21222125 TRUE 5183 928,467573 464,7374245 -0,182849 2 432,9765874 26,03 1 5,3 0,975234022 1,025394908 0 TFGFPVR ICPL (N-term)

gi|21222125 TRUE 1601 929,458784 465,23303 -0,16666 2 154,4959266 33,78 1 1,5 1,111514734 0,899673184 0 DVVAAGHR ICPL (N-term)

gi|21222125 TRUE 5688 1093,582914 547,295095 -0,12959 2 462,7589458 43,32 1 27,1 1,04637456 0,955680727 0 QVLPWLK ICPL (K); ICPL (N-term)

gi|21222125 TRUE 5684 1105,623182 553,315229 -0,083858 2 462,6298045 40,18 1 50,6 1,04637456 0,955680727 0 QVLPWLK ICPL:13C(6) (K); ICPL:13C(6) (N-term)

TRUE Rieske iron-sulfur protein

[S. coelicolor A3(2)] gi|21220626 130,69 5,949008499 6 4 9,69 2,17 4 0,12 0,03 60275086 549,4 TRUE 2175 1011,478162 506,242719 -0,123038 2 192,3677682 39,88 1 33,3 4,797072578 0,208460469 0 LEPDSIK ICPL (K); ICPL (N-term)

gi|21220626 TRUE 6787 1580,869596 527,6280493 -0,269548 3 560,3549749 49,5 1 191,9 14,24325585 0,070208667 0 VIFGPAGHALPQLR ICPL (N-term)

gi|21220626 TRUE 6785 1580,869596 790,938436 -0,193672 2 560,7020832 83,98 1 230,8 14,24325585 0,070208667 0 VIFGPAGHALPQLR ICPL (N-term)

gi|21220626 TRUE 770 1580,869596 527,6280493 -0,133948 3 80,7118912 31,76 1 14,2 12,08176342 0,082769374 0 VIFGPAGHALPQLR ICPL (N-term)

gi|21220626 TRUE 769 1580,869596 790,938436 -0,133872 2 80,6946496 62,48 1 67,1 12,08176342 0,082769374 0 VIFGPAGHALPQLR ICPL (N-term)

gi|21220626 TRUE 5288 1580,869596 790,938436 -0,103472 2 439,3246541 90,81 1 12,1 7,433028123 0,134534672 0 VIFGPAGHALPQLR ICPL (N-term)

TRUE hypothetical protein SCO3899

[S. coelicolor A3(2)] gi|21222306 127,7309952 8,055555556 2 2 3,62 0,74 2 0,3 0,06 5901918 55,9 TRUE 2529 1670,806074 835,906675 -0,13615 2 216,7762183 46,87 1 18,9 4,614106893 0,216726665 0 FTEAGVPIVGDDIK ICPL (K); ICPL (N-term)

gi|21222306 TRUE 7245 1756,942106 878,974691 -0,193982 2 591,5553669 84,86 1 37 2,61775472 0,38200676 0 VAFVNALPVFIAGTK ICPL (K); ICPL (N-term)

TRUE phosphoglyceromutase

[S. coelicolor A3(2)] gi|21222605 125,43 7,114624506 3 2 0,35 0,13 2 3,65 1,34 3033606 148 TRUE 3270 838,449628 419,728452 -0,168704 2 287,2035611 26,53 2 28,4 0,189231733 5,284525927 0 GGELLK ICPL:13C(6) (K); ICPL:13C(6) (N-term)

gi|21222605 TRUE 4899 1342,722593 671,8649345 -0,090069 2 415,6764791 84,74 1 35,4 0,501826885 1,992719061 0 TVLVAAHGNSLR ICPL (N-term)

gi|21222605 TRUE 4896 1348,742734 674,875005 -0,16881 2 415,5021874 98,9 1 84,2 0,501826885 1,992719061 0 TVLVAAHGNSLR ICPL:13C(6) (N-term)

TRUE phosphocarrier protein HPr

[S. coelicolor A3(2)] gi|21224185 118,86 27,95698925 2 2 1,76 0,41 2 0,63 0,15 1981532 28,2 TRUE 3709 1421,782606 711,394941 -0,116282 2 314,7390357 46,61 1 15,3 1,210177846 0,826324828 0 AATATGVPVTIAK ICPL:13C(6) (K); ICPL:13C(6)

(N-term)

gi|21224185 TRUE 2675 1503,757688 752,382482 -0,197564 2 227,4131682 72,25 1 12,9 2,298955479 0,43498015 0 LVAEGLEELPETV ICPL (N-term)

TRUE hypothetical protein SCO1384

[S. coelicolor A3(2)] gi|21219888 118,5669089 7,490636704 2 2 1,18 0,18 2 0,88 0,13 3229616 34,8 TRUE 5261 1088,552335 544,7798055 -0,307011 2 437,9191042 37,48 1 20,8 0,935391743 1,069070802 0 LVFYASQR ICPL (N-term)

gi|21219888 TRUE 3981 1397,705945 699,3566105 0,014579 2 332,276952 83,61 1 14 1,430590705 0,699011951 0 FLLDGELTTAGR ICPL (N-term)

TRUE secreted protein

[S. coelicolor A3(2)] gi|21221536 118,32 10,6557377 3 3 0,52 0,09 3 2,08 0,36 1968133 78,7 TRUE 1952 894,487089 447,7471825 -0,121365 2 178,0553431 35,88 1 22,4 0,74093913 1,349638532 0 VLAGQGK ICPL:13C(6) (K); ICPL:13C(6) (N-term)

gi|21221536 TRUE 1990 1254,611295 627,8092855 -0,038571 2 180,7078685 48,58 1 44,2 0,467690853 2,138164545 0 SQQIQIAEK ICPL (K); ICPL (N-term)

gi|21221536 TRUE 1646 1277,631147 639,3192115 -0,180823 2 157,7873933 33,86 1 12,1 0,36581377 2,733631374 0 SAERPAAEPK ICPL:13C(6) (K); ICPL:13C(6) (N-term)

TRUE D-alanyl-D-alanine dipeptidase (putative secreted protein)

[S. coelicolor A3(2)] gi|21219900 117,32 6,569343066 2 2 0,79 0,02 2 1,27 0,04 1097254 38,9 TRUE 3284 954,488767 477,7480215 -0,108243 2 288,2352272 56,9 1 19 0,753286259 1,327516581 0 AVDHFVR ICPL:13C(6) (N-term)

gi|21219900 TRUE 3416 1403,679807 702,3435415 -0,048283 2 296,6888693 60,42 1 19,9 0,821470869 1,21732862 0 YVTAHNFVGER ICPL:13C(6) (N-term)

TRUE serine hydroxymethyltransferase

[S. coelicolor A3(2)] gi|21223213 117,2569089 4,98960499 2 2 2,42 1,04 2 0,57 0,24 1836250 49,5 TRUE 3297 1356,64433 678,825803 -0,158406 2 289,0519611 47,01 1 29,8 1,181545478 0,846349141 0 RPAFQDYAQR ICPL (N-term)

gi|21223213 TRUE 2305 1750,803083 875,9051795 -0,072759 2 200,5675351 72,77 1 19,7 3,617334493 0,276446649 0 QVNDLTDADWAELR ICPL (N-term)

TRUE fructose-bisphosphate aldolase

[S. coelicolor A3(2)] gi|21222064 115,0769089 7,871720117 2 2 1,15 0,27 2 0,96 0,22 3568284 39 TRUE 2331 1226,605161 613,8062185 -0,069837 2 202,1020434 69,56 1 27,3 1,504093424 0,664852318 0 TVEALGLGEK ICPL (K); ICPL (N-term)

gi|21222064 TRUE 3897 2182,132978 728,0491767 -0,06863 3 326,4750107 48,04 1 11,7 0,797025935 1,254664317 1 DKLDGYVRPLIAVSEER ICPL:13C(6) (K); ICPL:13C(6) (N-term)

TRUE membrane protein

[S. coelicolor A3(2)] gi|21223676 114,8409952 12,21122112 3 2 0,25 0,01 2 4,04 0,18 5971224 89,2 TRUE 4795 1031,566617 516,2869465 -0,160493 2 408,8943042 25,58 1 16,9 0 ICGAAGLLTR

gi|21223676 TRUE 3315 1355,727415 452,5806557 -0,283067 3 290,2159605 58,69 1 32,2 0,233064992 4,290648684 0 ALIVNHEVGHR ICPL:13C(6) (N-term)

gi|21223676 TRUE 3906 2012,959669 671,6580737 -0,055421 3 327,1306523 34,57 1 40,1 0,264278861 3,7838819 0 WVLGSPEFDGPIHEYR ICPL:13C(6) (N-term)

TRUE lipoprotein

[S. coelicolor A3(2)] gi|21223308 114,097904 9,661835749 3 2 0,63 0,37 2 2,65 1,54 7679110 79,3 TRUE 2112 1421,709821 711,3585485 -0,117697 2 187,973285 48,36 1 13,6 0,999204407 1,000796226 0 TIPISAGSPDNK ICPL:13C(6) (K); ICPL:13C(6) (N-term)

gi|21223308 TRUE 5132 2462,124937 821,3798297 -0,209789 3 430,1769458 42,42 1 32,6 0,237977925 4,202070429 0 GITVNSSSGQEVACHWFSTQR ICPL:13C(6) (N-term)

TRUE secreted alkaline phosphatase

[S. coelicolor A3(2)] gi|32141154 113,8 3,266787659 2 2 0,43 0,12 2 2,69 0,76 8898931 93 TRUE 5084 948,535688 474,771482 -0,161164 2 427,1026957 51,48 1 50,5 0,589353856 1,696773491 0 DLLAHLR ICPL:13C(6) (N-term)

gi|32141154 TRUE 5409 1407,678739 704,3430075 -0,087615 2 446,3551207 62,32 1 42,5 0,27216082 3,674298155 0 GLAPATDYWFR ICPL:13C(6) (N-term)

TRUE large secreted protein

[S. coelicolor A3(2)] gi|21220395 112,14 3,588907015 2 2 0,35 0,14 2 3,79 1,51 6518716 48,4 TRUE 5419 1385,740339 693,3738075 -0,128615 2 446,9307458 49,85 1 25,7 0,513830348 1,946167648 0 KPLAGEFVFR ICPL:13C(6) (K); ICPL:13C(6) (N-term)

gi|21220395 TRUE 5326 1584,799665 792,9034705 -0,103141 2 441,3193127 62,29 1 22,7 0,179190654 5,580648205 0 TVFVVANHFNSK ICPL:13C(6) (K); ICPL:13C(6) (N-term)

TRUE 50S ribosomal protein L3

[S. coelicolor A3(2)] gi|21223082 107,1909952 10,28037383 4 2 3,23 1,45 2 0,43 0,19 1986368 60,5 TRUE 5750 866,513425 433,7603505 -0,215101 2 466,8316871 31,59 1 14,9 4,873210651 0,205203524 0 GLLLIK ICPL (K); ICPL (N-term)

gi|21223082 TRUE 5747 866,513425 866,513425 0,029075 1 466,6401709 29,09 1 17,4 4,873210651 0,205203524 0 GLLLIK ICPL (K); ICPL (N-term)

gi|21223082 TRUE 3325 1145,606183 573,3067295 -0,159859 2 290,7723776 48,66 1 12,2 1,523666432 0,65631163 0 AGPNVVTQVR ICPL (N-term)

TRUE hypothetical protein SCO5725

[S. coelicolor A3(2)] gi|21224072 100,12 20,38834951 2 2 0,32 0,03 2 3,21 0,31 2523774 85,2 TRUE 1749 1048,515348 524,761312 -0,184824 2 164,7186098 51,71 1 21,3 0,274231355 3,646556022 0 TAYGEIQR ICPL:13C(6) (N-term)

gi|21224072 TRUE 2387 1581,773481 791,3903785 -0,137357 2 205,8935517 48,41 1 63,9 0,359880835 2,778697565 0 VAAVAETWQGEAK ICPL:13C(6) (K); ICPL:13C(6) (N-term)

TRUE purine nucleoside phosphorylase

[S. coelicolor A3(2)] gi|21223291 99,89 6,934306569 5 2 0,58 0,03 2 1,73 0,1 17782567 135,9 TRUE 5410 880,503934 440,755605 -0,13841 2 446,3058375 52,37 1 12,2 0,535066868 1,868925288 0 ALVFLGR ICPL (N-term)

gi|21223291 TRUE 5404 880,503934 880,503934 -0,107034 1 446,3093623 37,07 1 27 0,535066868 1,868925288 0 ALVFLGR ICPL (N-term)

gi|21223291 TRUE 5401 886,524076 443,765676 -0,212752 2 446,0192706 48,18 1 34,9 0,535066868 1,868925288 0 ALVFLGR ICPL:13C(6) (N-term)

gi|21223291 TRUE 5402 886,524076 886,524076 -0,052676 1 446,0308791 33,11 1 26,7 0,535066868 1,868925288 0 ALVFLGR ICPL:13C(6) (N-term)

gi|21223291 TRUE 5181 1385,730131 693,3687035 -0,142807 2 433,0476455 47,52 1 35,1 0,628752623 1,590450621 0 TIILTNGCGGLR ICPL:13C(6) (N-term)

TRUE aldose 1-epimerase

[S. coelicolor A3(2)] gi|21220874 95,99 6,284153005 2 2 4,97 1,4 2 0,23 0,07 2508775 64,7 TRUE 4990 1159,60069 580,303983 -0,131366 2 421,3735874 64,67 1 36,3 6,777139462 0,14755488 0 SPHFGALIGR ICPL (N-term)

gi|21220874 TRUE 3328 1583,82884 528,614464 -0,268392 3 290,9837856 31,32 1 28,4 3,141989207 0,318269712 0 GITDRPEHIATLR ICPL (N-term)

TRUE 50S ribosomal protein L17

[S. coelicolor A3(2)] gi|21223109 95,73099517 8,928571429 2 1 7,65 0 1 0,13 0 630802 31,7 TRUE 5803 1065,609098 533,308187 -0,051774 2 469,603029 68,97 1 12,8 7,654080493 0,130649266 0 LLLANLAK ICPL (K); ICPL (N-term)

TRUE oxidoreductase (secreted protein)

[S. coelicolor A3(2)] gi|21225017 95,38 8,045977011 3 2 6,51 1,31 2 0,17 0,03 3180636 92 TRUE 7016 985,525403 493,2663395 -0,253879 2 576,364192 59,95 1 42,6 8,272923474 0,120876254 0 GVFALAFR ICPL (N-term)

gi|21225017 TRUE 7014 985,525403 985,525403 -0,035503 1 576,2205168 33,11 1 25,2 8,272923474 0,120876254 0 GVFALAFR ICPL (N-term)

gi|21225017 TRUE 3304 2100,000562 700,6717047 -0,204614 3 289,4919189 35,43 1 24,2 4,743423508 0,210818199 0 DAAHGLAVGGDYRPGQASPR ICPL (N-term)

TRUE hypothetical protein SCO2093

[S. coelicolor A3(2)] gi|21220573 94,55 11,37724551 2 2 0,26 0,02 2 3,88 0,31 2013275 21,3 TRUE 3289 915,498975 458,2531255 -0,162251 2 288,5428021 56,89 1 8,3 0,2904968 3,442378715 0 SLTISQR ICPL:13C(6) (N-term)

gi|21220573 TRUE 3571 1525,774106 763,390691 -0,133582 2 306,8223109 37,66 1 13 0,231410091 4,321332722 0 TSPFLLHTEDVR ICPL:13C(6) (N-term)

TRUE F0F1 ATP synthase subunit beta

[S. coelicolor A3(2)] gi|21223733 94,21690887 3,138075314 2 2 3,2 0,27 2 0,32 0,03 9375799 77,9 TRUE 4893 813,415449 407,2113625 -0,160725 2 415,3626957 26,86 1 40 3,576437309 0,279607865 0 WPIHR ICPL (N-term)

gi|21223733 TRUE 5825 1370,735456 685,871366 -0,066732 2 470,7757543 69,88 1 37,9 2,815970969 0,355117297 0 VIDLLTPYVK ICPL (K); ICPL (N-term)

TRUE spore-associated protein precursor

[S. coelicolor A3(2)] gi|21218951 93,81 11,68831169 2 2 2,16 0,15 2 0,47 0,03 3402201 92,9 TRUE 1696 968,46182 484,734548 -0,123896 2 161,1729266 30,13 1 47 2,369254861 0,42207363 0 NCTVTIR ICPL (N-term)

gi|21218951 TRUE 3420 1274,616392 637,811834 -0,127668 2 296,8983771 63,68 1 45,9 1,945232911 0,514077257 0 SYAGPVYVSAR ICPL (N-term)

TRUE secreted protein

[S. coelicolor A3(2)] gi|21222370 88,36 16,66666667 2 2 3,64 0,02 2 0,275 0,002 11972660 89,6 TRUE 5234 1780,901716 594,3054227 -0,130268 3 436,1223127 43,75 1 43,6 3,607395109 0,277208337 0 IVSTHGTQVVAFSPK ICPL (K); ICPL (N-term)

gi|21222370 TRUE 4038 2263,135343 755,049965 -0,014295 3 335,9009024 44,61 1 46 3,67197953 0,272332673 0 GIDPQIVGLTGEFDTIQAGAR ICPL (N-term)

TRUE 50S ribosomal protein L14

[S. coelicolor A3(2)] gi|21223092 88,00381774 18,03278689 2 2 2,96 1,24 2 0,46 0,19 1809391 53,8 TRUE 4249 1059,608442 1059,608442 -0,108042 1 350,5690187 49 1 39,8 4,411307261 0,226690172 0 IISLAPEVL ICPL (N-term)

gi|21223092 TRUE 7122 1515,784208 758,395742 0,202716 2 584,0081754 44,05 1 14 1,474604305 0,678148027 0 YAGIGDVIVATVK ICPL (K); ICPL (N-term)

TRUE glycine cleavage system H protein

[S. coelicolor A3(2)] gi|21223828 81,02 21,6 2 2 0,83 0,04 2 1,21 0,06 962732 33,6 TRUE 1585 953,489469 477,2483725 -0,138145 2 153,4713602 39,9 1 17,2 0,894150696 1,118379714 0 SNPQQLR ICPL:13C(6) (N-term)

gi|21223828 TRUE 2691 2174,976407 1087,991842 -0,262483 2 228,0810183 41,12 1 16,4 0,770653435 1,297600133 0 ITDEPADLLSADEYTAFAGA ICPL (N-term)

TRUE hypothetical protein SCO1074

[S. coelicolor A3(2)] gi|21219589 79,39 5,764966741 2 2 1,28 0,16 2 0,8 0,1 9641671 38,9 TRUE 5219 1248,694669 624,8509725 -0,064545 2 435,1633954 28,22 1 34,4 1,061073879 0,942441445 0 VGLPTVTSTLR ICPL (N-term)

gi|21219589 TRUE 7006 1663,810804 555,2751187 -0,150656 3 575,6684581 51,17 1 4,5 1,507008519 0,663566255 0 SSNPVAETVASLMPR ICPL (N-term)

TRUE polypeptide deformylase

[S. coelicolor A3(2)] gi|21223589 78,2 11,57407407 3 2 0,783 0,006 2 1,28 0,01 1617358 86,2 TRUE 3179 1142,552381 571,7798285 -0,129057 2 280,7157941 28,87 1 39,7 0,774027767 1,291943316 0 HVGVVCNPR ICPL (N-term)

gi|21223589 TRUE 3180 1142,552381 571,7798285 -0,125457 2 280,9467856 28,39 2 21,2 0,774027767 1,291943316 0 HVGVVCNPR ICPL (N-term)

gi|21223589 TRUE 5037 1885,007902 629,0074847 -0,136754 3 424,4748957 49,33 1 25,3 0,792321284 1,262114272 0 GTSRPITVVGNPVLHK ICPL (K); ICPL (N-term)

TRUE translation initiation factor IF-2

[S. coelicolor A3(2)] gi|32141270 76,46381774 1,74249758 2 2 1,02 0,91 2 2,55 2,29 1661656 37,9 TRUE 5047 1082,652433 541,8298545 -0,144309 2 425,213021 29,55 1 21,1 0,237630519 4,208213677 2 NTKARLLR ICPL:13C(6) (N-term)

gi|32141270 TRUE 2261 1248,553129 624,7802025 -0,025805 2 197,459485 51,96 1 16,8 1,682662406 0,594296275 0 GASLTDFAEK ICPL (K); ICPL (N-term)

TRUE hypothetical protein SCO3659

[S. coelicolor A3(2)] gi|21222074 74,45 17,97752809 2 2 0,58 0,18 2 2,04 0,63 2699395 51,6 TRUE 1587 1491,661146 497,8918993 -0,143098 3 153,608085 30,15 1 38,1 0,351948577 2,841324174 0 VYSHTHDDGTVR ICPL (N-term)

gi|21222074 TRUE 3710 2675,259672 669,570375 0,4661 4 314,8209104 44,3 1 13,5 0,809048911 1,236019215 1 SHQIDEKPQLLESVDDWNRR ICPL (K); ICPL (N-term)

TRUE cystathionine gamma-synthase

[S. coelicolor A3(2)] gi|21223331 74,31 9,067357513 2 2 0,66 0,19 2 1,76 0,51 11510919 59,4 TRUE 2385 1554,785352 777,896314 0,051372 2 205,9070263 46,88 1 22,8 0,910551031 1,098236085 0 TALEENLAALEGGR ICPL:13C(6) (N-term)

gi|21223331 TRUE 5142 2403,220411 801,7449877 -0,181463 3 430,6468706 27,43 1 36,6 0,415185161 2,408563925 0 TLLRPGDHVVIPNDAYGGTFR ICPL (N-term)

TRUE secreted protein

[S. coelicolor A3(2)] gi|21220689 73,62 5,189620758 2 2 1,09 0,21 2 0,99 0,19 2307141 57,8 TRUE 6824 1289,631315 645,3192955 -0,174991 2 563,0884586 45,34 1 34,6 1,372352426 0,728675799 0 STYYWPVLR ICPL (N-term)

gi|21220689 TRUE 3704 2212,089429 738,0346603 -0,214681 3 314,4442603 28,28 1 23,2 0,801188822 1,248145223 1 QVTDKYPLCPEGSQVLR ICPL:13C(6) (K); ICPL:13C(6) (N-term)

TRUE hypothetical protein SCO6176

[S. coelicolor A3(2)] gi|21224501 61,86099517 9,782608696 2 2 1,67 0,22 2 0,62 0,08 695631 59,7 TRUE 1526 1133,533384 567,27033 -0,10606 2 149,2647351 25,1 1 42,3 1,365928999 0,732102474 0 SATNHEITR ICPL (N-term)

gi|21224501 TRUE 5602 2030,032499 677,349017 -0,185751 3 457,6094957 40,76 1 17,4 1,982261057 0,504474422 0 VSFAPIYSLDPVTAGEPR ICPL:13C(6) (N-term)

__________________________________________________________________________________________________________________________________________________________________________________________________________________________________________

Extracellular proteins of *S. coelicolor* M145 were labelled with C13 and those of *S. coelicolor* M28 were labelled with C12 at 24h of growth.
