# Supplementary material for: A Novel Two-Component System Involved in the Transition to Secondary Metabolism in Streptomyces coelicolor
Source: PLoS One. 2012 Feb 9;7(2):e31760. doi: 10.1371/journal.pone.0031760 (PMC3276577; doi:10.1371/journal.pone.0031760)
Supplement: Table S5 — Oligonucleotide primers used for gene transcript amplification. Oligonucleotide sequences start and terminate at their 5′ and 3′ ends, respectively. (DOC) [file pone.0031760.s006.doc]

Table S5. Oligonucleotide primers used for gene transcript amplification

**Gene**  **Forward primer Reverse primer**

SCO0297 CGGGCTACTACGGCTTCTAC CTTCCTCAAGGGTGGTGAA

SCO0762 (*sti1*) CGAGGACTCGGTGATGTG AAGGTGCGTTCGTAGGAGAG

SCO1230 ACTACCTCGGCGTCTCCTAC GGAAGAGGGTGCCGTAGAC

SCO1513 (*relA*) GCGAGAAGCAGGAGAAGAAG CTCCAGCTCCCACTTGATG

SCO1860 ACCGTCCTCGGCAAGAAG GGTTGACGGAGACCTTGAGT

SCO4561 CCTACGACTGCTCCGGTCTC TGATGCCGACCTGCTTGA

SCO5074 CAAGAAGTTCAAGGCCACCTA GTCGTTGACGATCTGGTTGT

SCO5087 AGGAGCTGTTCGGATTGAAG AGGTGAGCAGTTCCCAGAA

SCO5085 (*actII-4*) AGGCGCTGGAATCGTATC CCGTTGAGAATTTCCATGTG

SCO5785 (*degU*) CGACGAGCCGTTCATCTAC TCTTGGCGCTGATGAAGA

SCO5878 (redX) GTCGCACAGCAGGATCAC GCGCTTGAGCAGGAAGAG

SCO5881 (redZ) CTGACCAAGCCCGAGAATA CAGTTCTTCGACCGACGTT

SCO6197 ATGAGCACAACCAGTGTGAAC TGCGGGTAATTGAAGTCCTC

SCO6198 CCAGACCAAGGACTTCATCA ATGGTGGTGGAGATCAGGTT

SCO6199 CGGATCACCGACTTCAACT CGAGGTCCTGGGTGATCT

Oligonucleotide sequences start and terminate at their 5’ and 3’ ends, respectively.
